# Crystal Lattice Analysis for 2D Nanomorphology Prediction of Phase-Separated Materials

**DOI:** 10.1021/jacs.4c14964

**Published:** 2025-01-06

**Authors:** Tobias Schnitzer, Bart W. L. van den Bersselaar, Brigitte A. G. Lamers, Martin H. C. van Son, Stefan J. D. Maessen, Freek V. de Graaf, Bas F. M. de Waal, Nils Trapp, Ghislaine Vantomme, E. W. Meijer

**Affiliations:** † Institute for Complex Molecular Systems, Eindhoven University of Technology, 5600MB Eindhoven, The Netherlands; ‡ Institute of Organic Chemistry, Albert-Ludwigs University Freiburg, Albertstraße 21, 79110 Freiburg im Breisgau, Germany; § Small Molecule Crystallography Center, ETH Zürich, Vladimir-Prelog-Weg 3, 8093 Zürich, Switzerland

## Abstract

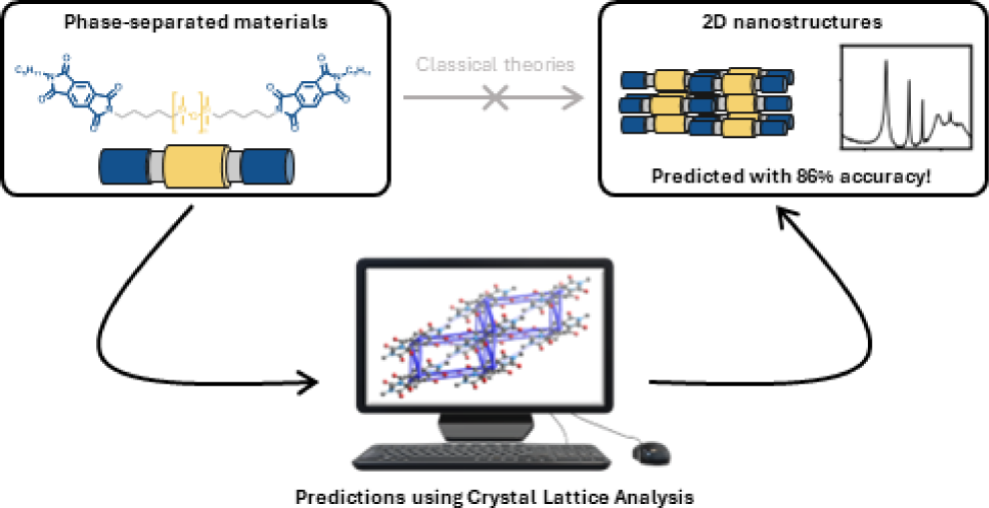

Spontaneous phase separation of materials is a powerful strategy to generate highly defined 2D nanomorphologies with novel properties and functions. Exemplary are such morphologies in block copolymers or amphiphilic systems, whose formation can be well predicted based on parameters such as volume fraction and shape factor. In contrast, the formation of 2D nanomorphologies is currently unpredictable in materials perfectly defined at the molecular level, in which crystallinity plays a significant role. Here, we introduce a crystal lattice analysis to predict a priori the formation of 2D nanomorphologies from the crystalline units in phase-separated soft materials. We show that the formation of lamellar morphologies, their domain spacings, and thermal transition temperatures of such materials can be predicted using a straightforward crystal lattice analysis workflow. We envision this approach to facilitate the design and discovery of new materials with 2D nanomorphologies that are essential for next-generation electronic applications.

## Introduction

The development of new materials with well-defined morphologies is time-consuming, yet essential for next-generation applications.^1^ The design of materials that spontaneously form highly ordered two-dimensional (2D) nanostructures is even more challenging.^2−4^ Typically, so-called lamellar morphologies are pursued because their sheet-like nanostructures are beneficial in many applications, ranging from single-layer membranes to optoelectronic materials and artificial skins.^5−8^ In recent decades, extensive research on block copolymers with low dispersity (*Đ*) and discrete block co-oligomers without dispersity (*Đ* < 1.00001) has enabled greater control over morphologies.^9−11^ To further increase the level of order and reduce the domain spacing, attention shifted to the combination of crystalline blocks with discrete amorphous oligodimethylsiloxane (*o*DMS, *Đ* = 1.00001) as this yielded phase-separated materials (block molecules) with an extraordinary level of organization.^12−14^ Despite careful molecular design, the morphologies of these crystalline-*o*DMS hybrid molecules often remain unpredictable^15−17^ when considering conventional parameters for block copolymers^18^ and liquid crystals,^19,20^ such as volume fraction (φ) and shape factor.

**Figure 1:**
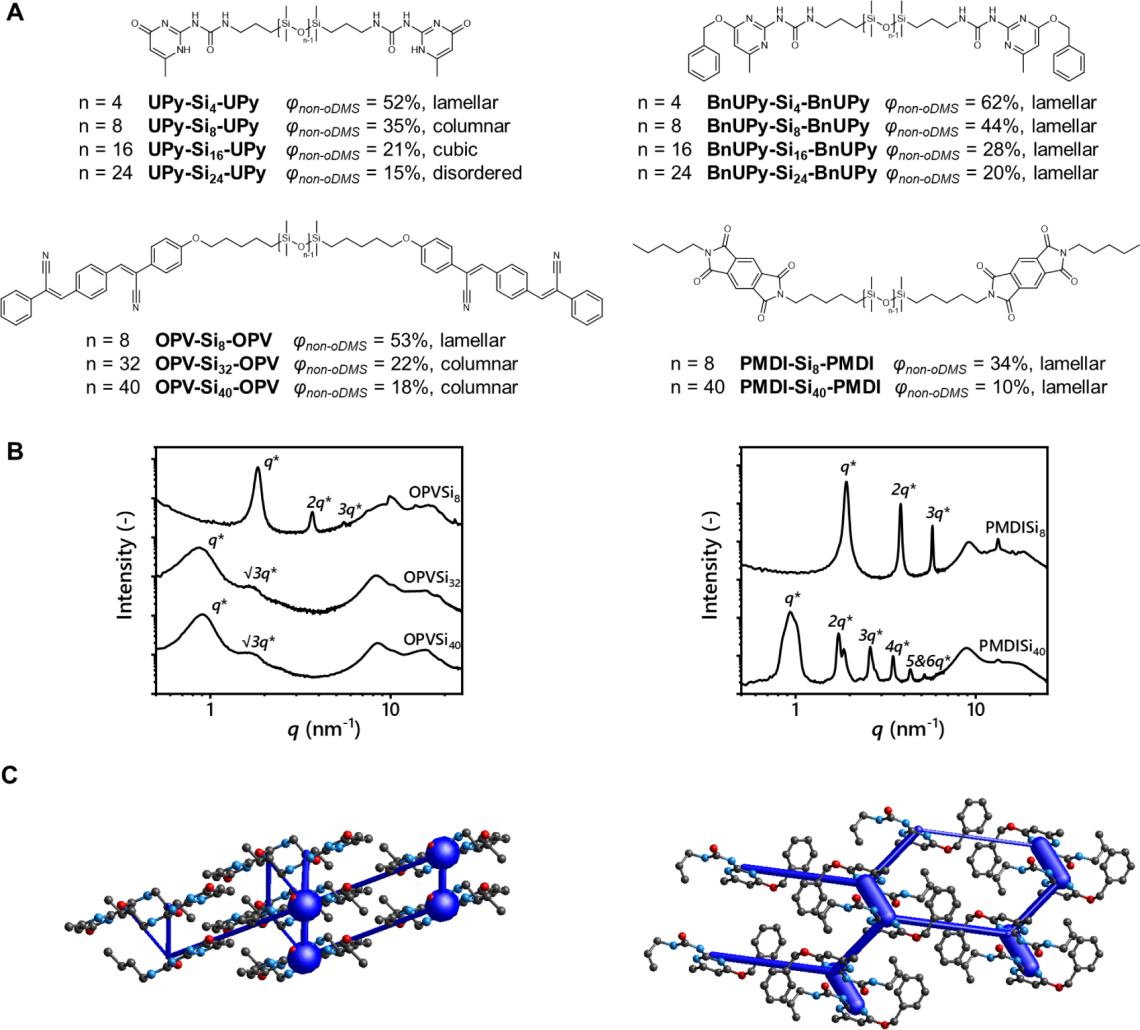
(A) Chemical structures and observed morphologies of **UPy-Si**_***n***_**-UPy** (*n* = 4, 8, 16, 24), **BnUPy-Si**_***n***_**-BnUPy** (*n* = 4, 8, 16, 24), **OPV-Si**_***n***_**-OPV** (*n* = 8, 32, 40), and **PMDI-Si**_***n***_**-PMDI** (*n* = 8, 40). (B) Medium/wide-angle X-ray scattering (MAXS/WAXS) patterns of **OPV-Si**_***n***_**-OPV** and **PMDI-Si**_***n***_**-PMDI**. Intensity is shown in arbitrary units, and graphs are stacked for visual clarity. (C) Crystal structures of **OPV** and **BnUPy** (CCDC identifiers: SAPNII01 and OLICOD). The width of the blue column of the energy framework is proportional to the total attractive energy between the connected molecules.

Exemplary of this uncertainty is the striking difference between a previously reported material based on ureidopyrimidinones (**UPy**) and its synthetic precursor, *O*-benzylated UPy (**BnUPy**) (Figure 1A).^21^ We incorporated **UPy** moieties into a block molecule to exploit the synergy between their strong tendency to dimerize and the phase separation induced by the *o*DMS.^22^ The morphologies of these materials comprising “soft” *o*DMS and “hard” crystalline segments were strictly determined by the respective volume fractions of the latter (φ_non-*o*DMS_), thereby matching the behavior of block copolymers (φ_non-*o*DMS_ = 15–52%, calculation of the volume fraction can be found in Supporting Information). In contrast, block molecules based on **BnUPy** moieties showed lamellar morphologies regardless of the volume fraction of the crystalline block (φ_non-*o*DMS_ = 20–62%). We also designed oligophenylvinylene (**OPV**) and pyromellitic diimide (**PMDI**)-based block molecule materials to exploit their functionality in an ordered structure (Figure 1A). Similar to the **UPy**-based materials, X-ray scattering experiments on the **OPV**-based block molecules revealed the formation of sheet-like structures only when an appropriate volume fraction was chosen (φ_non-*o*DMS_ = 53%, Figure 1B). Contrarily, phase separation into lamellae was consistently observed in the **PMDI**-based block molecules, regardless of the volume fractions (φ_non-*o*DMS_ = 10–34%). To elucidate the differences between these block molecules, we analyzed the crystal structures of the crystalline moieties using Crystal Explorer, which is a straightforward tool to perform energy decomposition analyses on crystal structures.^23^ Most importantly, the program can quantify the interactions among molecules in a crystal lattice. We found that the crystal packing of **UPy** and **BnUPy** differs significantly, with the latter inducing the formation of 2D structures via strong intramolecular interactions aligned within a layer (Figure 1C). In contrast, crystal lattice analysis of **UPy** revealed no such layer of strong interactions. Close inspection of the crystalline packing of **OPV** and **PMDI** revealed a similar phenomenon, where a clear directionality of the strong interactions can be found for **PMDI** but not for **OPV** (Figures S68 and S81).

We hypothesize that this different ordering in the crystal structure explains why a deviation from block copolymer theory was observed for **BnUPy**- and **PMDI**-based molecules. To formulate design rules to arrive at well-defined lamellar morphologies in these phase-separated materials, we introduce the concept of crystal lattice analysis (CLA). We established a workflow to support nanomorphology prediction of block molecules and the design of functional 2D materials. Subsequently, we applied this approach to a large library of discrete block molecules synthesized by our group to demonstrate the broad applicability of this approach.

## Results and Discussion

**Figure 2:**
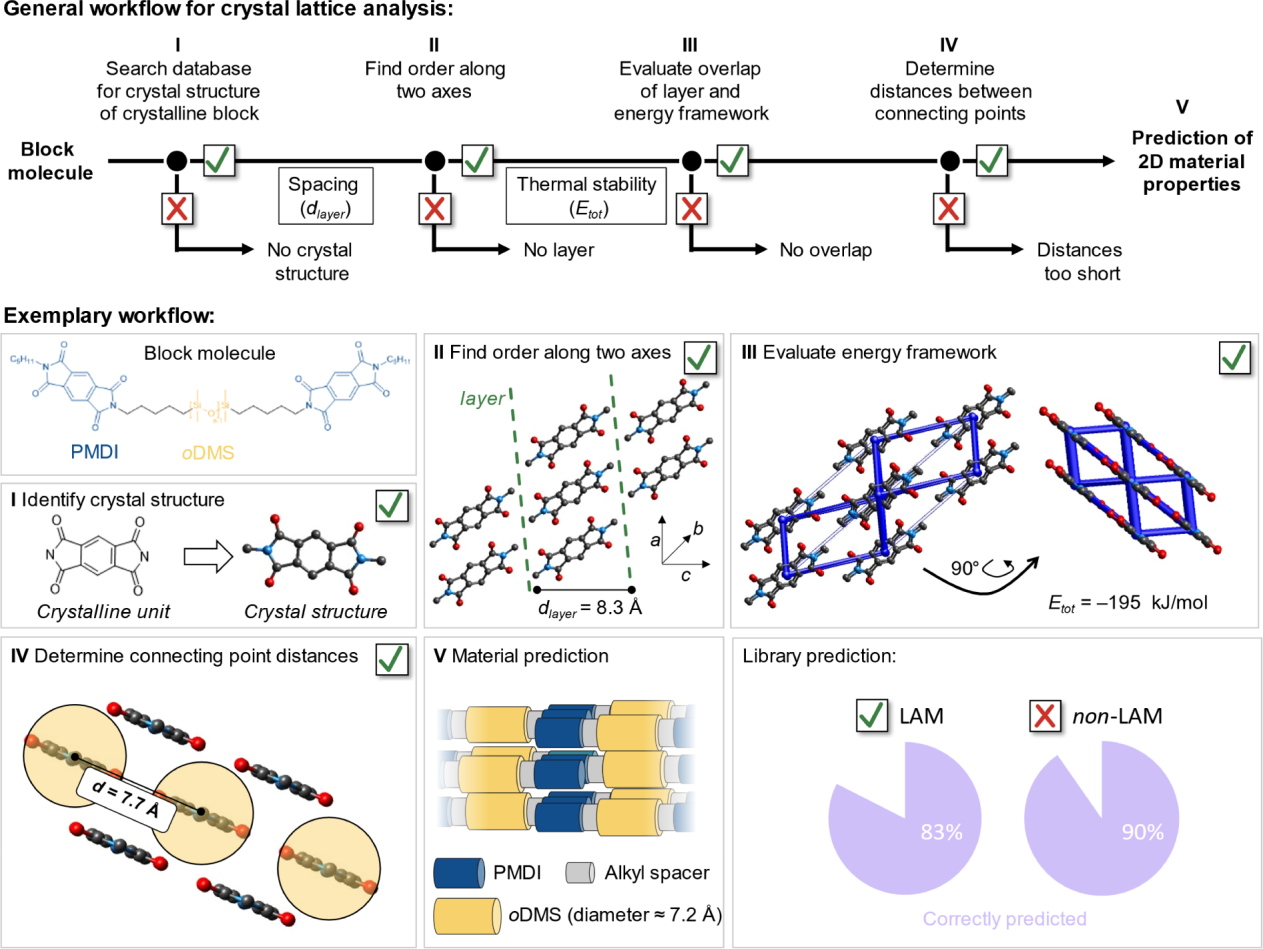
Crystal lattice analysis workflow. Top: stepwise analysis of the crystal lattice was performed using Crystal Explorer. Bottom: an exemplary workflow on a **PMDI**-based block molecule is shown. The blue column width of the energy framework is proportional to the sum of all of the interaction energies in the crystal structure.

A five-step workflow for the crystal lattice analysis (CLA) was designed to predict the presence of crystalline 2D morphologies and their properties in block molecules (Figure 2): (I) selection of an appropriate crystal structure of the crystalline part; (II) identification of the arrangement within the crystal lattice along two axes, forming the crystalline 2D domain of the lamellae (prediction of lamellar spacing via *d*_layer_); (III) quantification of the interactions within the crystal lattice using Crystal Explorer and calculation of the total interaction energy (*E*_tot_) of a molecule in the layer to its neighboring molecules [prediction of the endothermic transition temperature (*T*_endo_)]; (IV) comparison of the distance between connecting points and the theoretical diameter of *o*DMS chains (7.2 Å);^24^ (V) prediction of the formation of 2D morphologies. These five steps are all crucial to correctly predict the 2D material properties, while the order in which they are presented provides the most efficient workflow. To illustrate the workflow, the stepwise CLA of a **PMDI**-based block molecule is presented first. This block molecule consists of an *o*DMS chain of eight repeat units flanked by two **PMDI** units (Figure 2).

In step I, an appropriate crystal structure of the crystalline unit must be found. Note that different polymorphs of the same crystalline moiety can result in slightly different *E*_tot_ values (Figures S68–S70). Longer aliphatic chains in the crystal structure generally result in a higher *E*_tot_ in the energy analysis (step III, see Figures S72–S77). Thus, for consistency, we aim for structures with the shortest possible aliphatic terminus (e.g., methyl, methoxy, acetyl). In this case, we identified dimethyl **PMDI** as the appropriate structure for the CLA of the block molecule (CCDC identifier: RAGTIF).

Next, the arrangement of molecules along two axes in the unit cell is investigated by identifying the slip planes in the crystal unit in step II using Mercury software. The **PMDI** lattice indeed shows layers of molecules along both the *a*- and *b*-axis of the crystal with a layer width of *d*_layer_ = 8.3 Å. By considering lengths of 1.2 and 1.55 Å for a methylene and one dimethylsiloxane unit, respectively (for details, see Section S1), it was predicted that the lamellar domain (comprising one PMDI, ten methylene units, and eight dimethylsiloxane units) would have a domain spacing of *d*_pred_ = 33 Å. The energy analysis of the lattice using Crystal Explorer at the level of B3-LYP/6-31G(d,p) is evaluated in step III. To form lamellae, the high-energy intramolecular interactions must superimpose with the layer identified in the previous step. Moreover, this analysis allows for the determination of the energetic sum of interactions to adjacent molecules within the layer (*E*_tot_). The binding energy *E*_tot_ of a **PMDI** molecule in the crystalline layer was calculated as −195.4 kJ/mol. This energy serves as a measure to estimate the *T*_endo_ of the material (Figure 4A).

**Figure 3:**
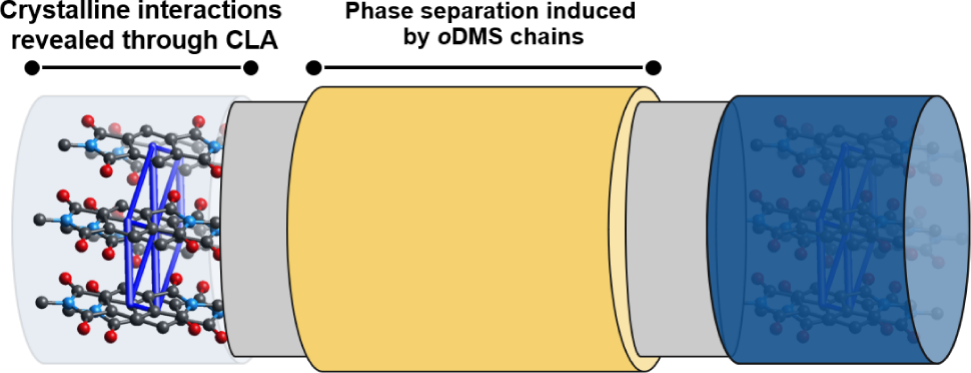
Representation of **PMDI-Si**_**8**_**-PMDI** showing the combination of crystalline interactions in the **PMDI**-block (blue), alkyl spacers (gray), and the phase-separation induced by the *o*DMS pendant (yellow).

In step IV, the relative positions of the connecting points between the crystalline units and *o*DMS chains are considered. The shortest distance between two connecting points should not be less than the diameter of an *o*DMS chain (7.2 Å). In the case of the **PMDI** molecule, the distance of 7.7 Å provided sufficient space for the *o*DMS chains (Figure 2), resulting in the siloxane side chains alternately pointing in- and out-of-plane of the **PMDI** layer. Finally, in step V, the formation of lamellae in **PMDI-Si**_**8**_**-PMDI** with a domain spacing of 33 Å was predicted. Figure 3 shows the strong intermolecular crystalline interactions present in the **PMDI** part (blue) of the molecule, while phase separation induced by the *o*DMS chains (in yellow) ensures segmentation of the crystalline blocks and the *o*DMS pendants. The molecule was then synthesized, and its morphology investigated using medium/wide-angle X-ray scattering (MAXS/WAXS). Indeed, highly ordered lamellae were found with an experimentally determined spacing (*d*_exp_) of 33 Å, which aligns perfectly with *d*_pred_ = 33 Å (Figure S9).

**Figure 4:**
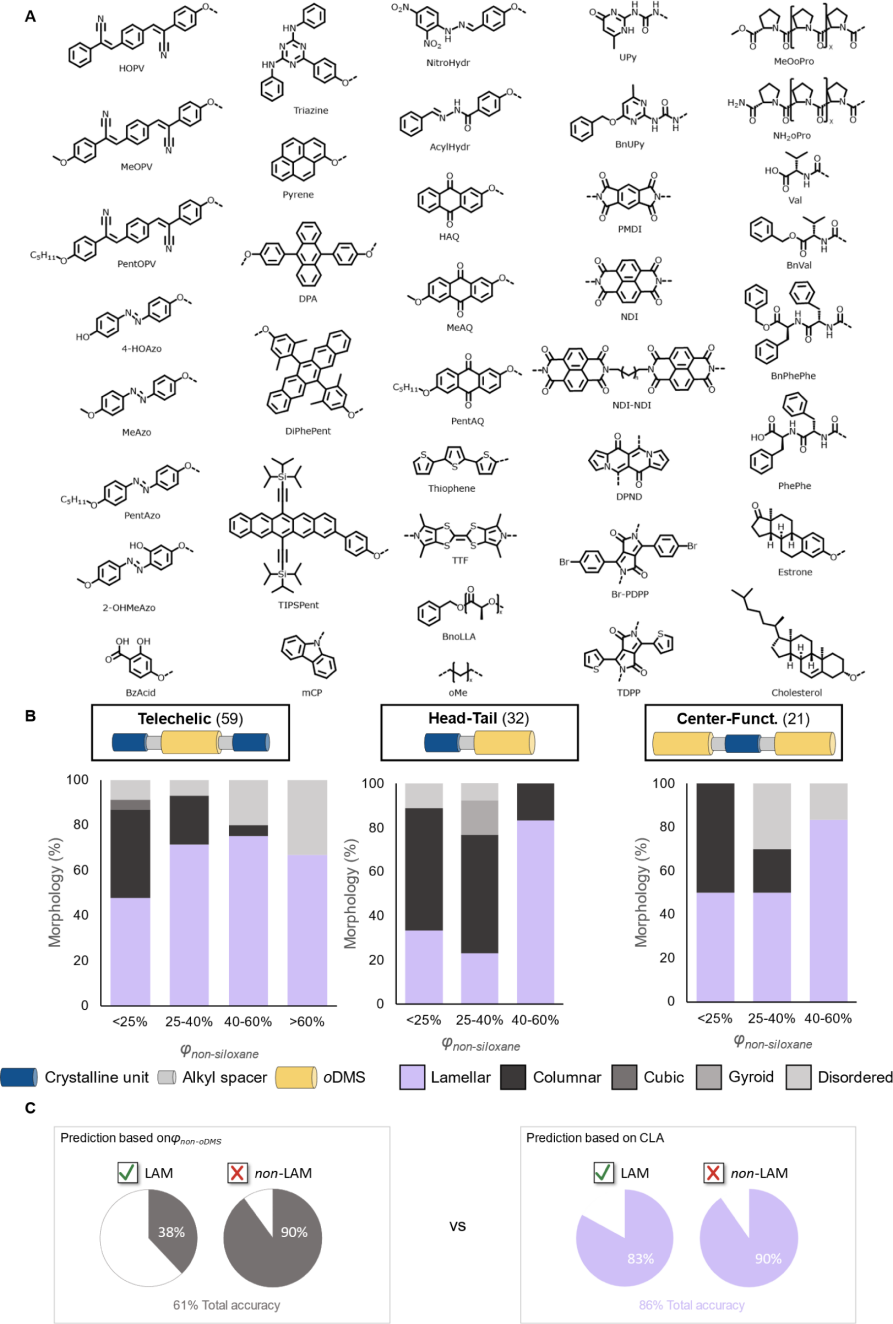
(A) Chemical structures of crystalline units from the various block molecules in the library. (B) Schematic representation of the three block molecule architectures and distribution of the morphologies for each architecture (lamellar: purple; columnar: black; cubic: dark gray; gyroid: gray; disordered: light gray). (C) Comparison of the prediction of the presence/absence of lamellae made using φ_non-*o*DMS_ versus predictions made using CLA.

Next, we used CLA to analyze all previously synthesized block molecules from our lab and evaluated their morphologies.^16,17,21,25,26^ This diverse library of block molecules comprised 39 different crystalline units and *o*DMS chains of various lengths (Figure 4A). The crystalline units are chiral/achiral, linear/cyclic, aliphatic/(hetero)aromatic and contain various functional groups (Figure 4A), providing a diverse mixture of different molecules that allows for generalization of our results. Three types of block molecule architectures are included in the library (Figure 4B), where the crystalline moiety is either at both ends (i.e., telechelic), at one end (i.e., head–tail), or in the center of the molecule (i.e., center-functionalized).^27^

Using our library, we investigated whether the observed morphologies in these materials deviated from what would be predicted based on the theory of phase separation in block copolymers.^28^ Block copolymers are expected to spontaneously phase separate into lamellae when the volume fractions of both blocks are approximately equal (φ_non-*o*DMS_ ∼ 40–60%). We determined φ_non-*o*DMS_ for all block molecules and correlated this to the morphology derived from MAXS/WAXS (Figures S2A–S20A). The data used for the CLA of all architectures are presented in Tables 1, S1, and S2. Block molecules with head–tail architectures mostly form columnar and lamellar structures (32 examples in Table S1). Since less than one-third of the structures had a reported crystal structure available, we herein focus on the telechelic and center-functionalized architectures. Intriguingly, the morphologies found in center-functionalized (21 examples, Table S2) and telechelic (59 examples, Table 1) architectures deviated significantly from block copolymer theory: 45 out of these 80 molecules form lamellae, independent of the φ_non-*o*DMS_. The growth of lamellae in telechelic materials can occur with siloxane chains pointing in alternating directions, as visualized in Figure 2, leading to covalent linkages between adjacent layers. This interconnection is absent in center-functionalized materials, where every distance between connecting points must be larger than 7.2 Å to accommodate the *o*DMS chains. Although the growth mechanisms of the lamellar domains are expected to differ as a result of the presence/absence of this interconnection, the 2D directionality in the crystalline unit dictates the eventual formation of lamellae. Remarkably, only around 40% of the center-functionalized and telechelic materials forming lamellae were correctly identified when only φ_non-*o*DMS_ was considered (Figure 4C). Therefore, we investigated these 80 block molecules further using CLA.

**Table 1:** Characteristics of Telechelic Block Molecules

Crystalline unit	# *o*DMS	φ_non-*o*DMS_^a^ (−)	1. Crystal structure^b^	2. Ordering in layer	3. Overlap energy framework	4. Intramol. distance (Å)^c^	5. Pred. *d*_lam_^d^ (Å)	Morph.^e^	*d*_exp_^f^ (Å)
2-HOMeAzo	16	0.38	EXEZUC	LAM	YES (−158.9 kJ/mol)	9.6	63	LAM	52
4-HOAzo	16	0.45	-	-	-	-	-	CYL	64
AcylHydr	40	0.15	RUJQOD	LAM	YES (−190.0 kJ/mol)	5.1	-	CYL	57
	8	0.47		LAM	YES (−190.0 kJ/mol)	5.1	-	LAM	35
BnOVal	8	0.49	-	-	-	-	-	Dis	-
BnPhePhe	8	0.63	-	-	-	-	-	LAM	33
BnUPy	24	0.18	OLICOD	LAM	YES (−255.2 kJ/mol)	7.8	51	LAM	48
	16	0.24		LAM	YES (−255.2 kJ/mol)	7.8	39	LAM	35
	8	0.39		LAM	YES (−255.2 kJ/mol)	7.8	27	LAM	22
	4	0.55		LAM	YES (−255.2 kJ/mol)	7.8	20	LAM	17
BzAcid	40	0.1	VAXYEA	LAM	-	-	-	Dis	-
	8	0.36		LAM	-	-	-	CYL	34
Cholesterol	40	0.23	BUGLEU	LAM	YES (−232.7 kJ/mol)	3.7	-	DIS	-
	8	0.6		LAM	YES (−232.7 kJ/mol)	3.7^g^	-	LAM	36
DPA	32	0.26	DPANTR	LAM	YES (−192.2 kJ/mol)	9.5	70	LAM	61
Estrone	8	0.5	MXESTO	LAM	NO	-	-	Dis	-
HAQ	32	0.2	ANTQUO	LAM	YES (−134.8 kJ/mol)	8.7^g^	67	CYL	59
	40	0.12		LAM	YES (−134.8 kJ/mol)	8.7^g^	79	CYL	88
	8	0.42		LAM	YES (−134.8 kJ/mol)	8.7	30	LAM	42
HOPV	32	0.21	REDHIR02	LAM	YES (−288.4 kJ/mol)	6.8	-	CYL	62
mCP	8	0.52	IFOREC	-	-	-	-	Dis	-
	4	0.67		-	-	-	-	Dis	-
MeAQ	32	0.2	ANTQUO	LAM	YES (−134.8 kJ/mol)	8.7^g^	56	CYL	57
MeAzo	16	0.29	AzPhen10	LAM	YES (−204.0 kJ/mol)	7.6	42	LAM	42
	16	0.38		LAM	YES (−204.0 kJ/mol)	7.6	57	LAM	51
	8	0.55		LAM	YES (−204.0 kJ/mol)	7.6	44	LAM	37
MeOPV	32	0.21	REDHIR02	LAM	YES (−288.4 kJ/mol)	6.8	-	CYL	68
NDI	24	0.17	DAHMUX	LAM	YES (−213.2 kJ/mol)	9.2	70	LAM	69
	16	0.26		LAM	YES (−213.2 kJ/mol)	9.2	58	LAM	57
	8	0.38		LAM	YES (−213.2 kJ/mol)	9.2	44	LAM	44
	8	0.49		LAM	YES (−213.2 kJ/mol)	9.5	30	LAM	31
	8	0.56		LAM	YES (−213.2 kJ/mol)	9.5	45	LAM	38
NDI-NDI	24	0.3		LAM	YES (−213.2 kJ/mol)	9.2	77	LAM	72
	16	0.42		LAM	YES (−213.2 kJ/mol)	9.2	65	LAM	63
	8	0.57		LAM	YES (−213.2 kJ/mol)	9.2	52	LAM	52
NitroHydr	40	0.15	YEFFAR	LAM	YES (−250.6 kJ/mol)	6.2	-	CYL	54
	24	0.23		LAM	YES (−250.6 kJ/mol)	6.2	-	CYL	46
	16	0.31		LAM	YES (−250.6 kJ/mol)	6.2	-	CYL	42
	8	0.47		LAM	YES (−250.6 kJ/mol)	6.2^g^	35	LAM	34
oMe	16	0.48	-	-	-	-	-	LAM	86
PentAQ	32	0.2	S.I. Six^h^	LAM	YES (−282.4 kJ/mol)	8.7	67	LAM	63
PentAzo	32	0.21	AzPhen10	LAM	YES (−204.0 kJ/mol)	7.6	67	LAM	67
PentOPV	32	0.24	REDHIR02	LAM	YES (−288.4 kJ/mol)	6.8^g^	79	LAM	72
PhePhe	8	0.53	METXEP	LAM	YES (−365.2 kJ/mol)	8.6	35	LAM	26
PMDI	40	0.09	RAGTIF	LAM	YES (−195.4 kJ/mol)	7.8	82	LAM	73
	8	0.33		LAM	YES (−195.4 kJ/mol)	7.8	33	LAM	33
Pyrene	40	0.13	S.I. Six^h^	LAM	YES (−262.8 kJ/mol)	7.4	86	LAM	79
	24	0.2		LAM	YES (−262.8 kJ/mol)	7.4	61	LAM	62
	8	0.43		LAM	YES (−262.8 kJ/mol)	7.4	36	LAM	33
Thiophene	8	0.44	EYUXEB	LAM	-i	-	34	LAM	34
	16	0.28		LAM	-i	-	47	LAM	44
TIPSPent	16	0.51	-	-	-	-	-	Dis	-
Triazine	8	0.5	-	-	-	-	-	LAM	38
	40	0.1		-	-	-	-	LAM	91
UPy	24	0.13	SOBLUQ	LAM	YES (−389.1 kJ/mol)	4.7	-	Dis	-
	16	0.18		LAM	YES (−389.1 kJ/mol)	4.7	-	CUB	48
	8	0.31		LAM	YES (−389.1 kJ/mol)	4.7	-	CYL	32
	4	0.46		LAM	YES (−389.1 kJ/mol)	4.7	-	LAM	22
Val	8	0.39	EWOTUF	LAM	YES (−212.2 kJ/mol)	6.7	-	Dis	-

a Volume fraction of the non-*o*DMS part of the molecule.

b Identifiers of the crystal structures were retrieved from the CCDC.

c Distance between the connection points of the *o*DMS chains.

d Predicted from the width of the crystalline layer and the length of the spacer ( = 1.2 Å, *d*_*o*DMS_ = 1.55 Å).

e Morphology determined from the pattern of the Bragg’s reflection peaks in SAXS patterns (LAM = lamellar, CYL = cylindrical, CUB = cubic, and Dis = disordered).

f Calculated using the formula *d*_exp_ = 2π/*q*.

g Measured distance contradicts the observed morphology.

h Crystal structure was determined in this work and is shown in Supporting Information 6.

i No energy calculation could be performed due to unresolved atoms in the crystal structure.

Two considerations allowed us to predict the formation of lamellar materials based on an existing crystal structure of the crystalline block: (a) does the crystal contain a layer that superimposes with the high-energy framework (step II/III)? and (b) Is the distance of connecting points larger than 7.2 Å (step IV)? Of the above set of 80 block molecules, 9 contained a crystalline unit for which no crystal structure was available. Of the remaining 71 block molecules, 40 formed lamellae and 31 did not (Tables 1 and S2). From those 40 lamellae-forming molecules, we correctly predicted 33 block molecules to form lamellae using CLA (83% correctly predicted versus 40% with the BCP theory). For the remaining seven block molecules, CLA predicts that no lamellae are formed, yet the material actually phase-separated into 2D nanostructures. Interestingly, more than half of those seven exceptions have a φ_non-*o*DMS_ of ∼40–60%, suggesting that these materials form phase-separated structures similar to block copolymers and that the formation of their 2D nanomorphology is not driven by crystallinity. Strikingly, CLA correctly predicted the lack of lamellae in 28 of 31 block molecules that do not form 2D morphologies (90%). In summary, CLA correctly predicts the presence—or absence—of lamellae in phase-separated molecules in 86% of the cases (61 out of total 71), compared to 60% accuracy that is achieved when the theory of phase separation in block copolymers is considered (43 out of total 71, Figure 4C). The remaining inaccuracy of CLA is explained by the aforementioned block molecules that form into 2D nanostructures following the BCP theory (φ_non-*o*DMS_ = 40–60%). As a result, the accuracy of CLA will increase further when block molecules with φ_non-*o*DMS_ ≠ 40–60% are targeted.

**Figure 5:**
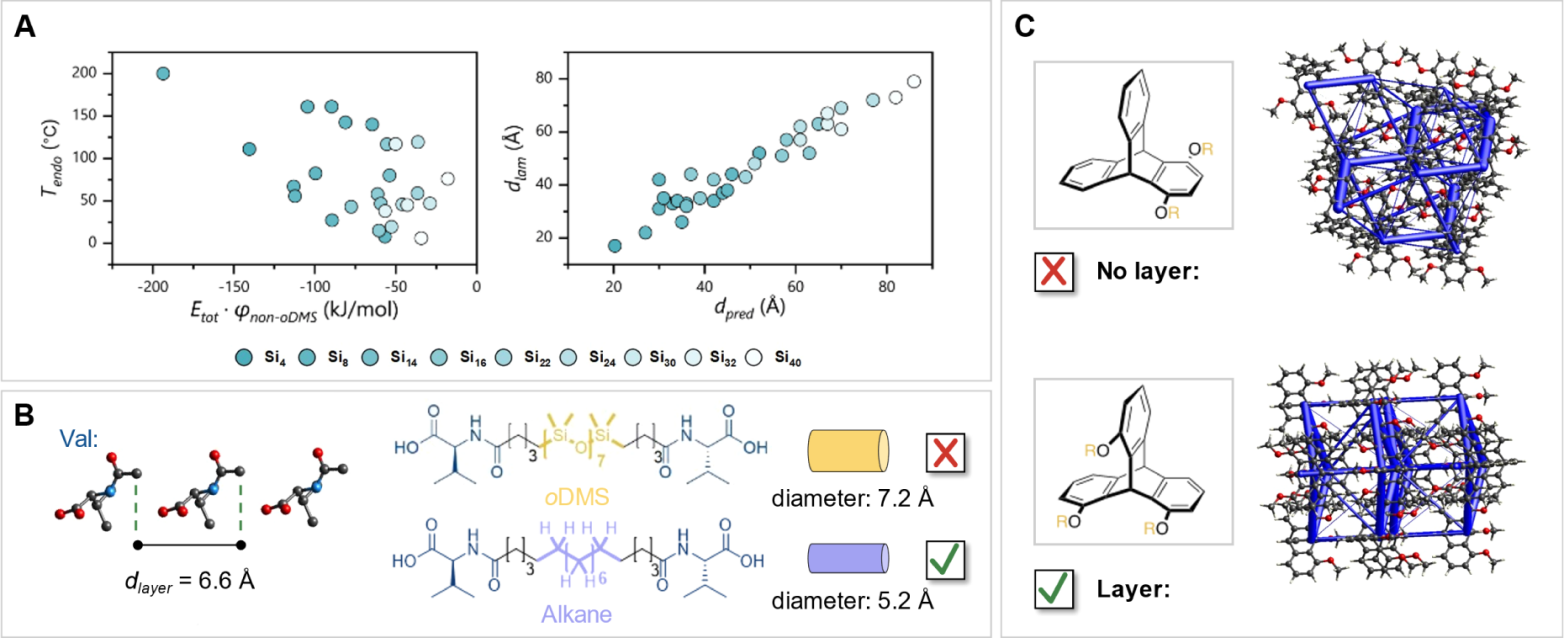
(A) Correlation of *T*_endo_ with *E*_tot_ · φ_non-*o*DMS_ (left, Figure S20A) and *d*_exp_ with *d*_pred_ (right, Figure S20B). (B) Measured intermolecular distance in valine (CCDC identifier: EWOTUF, left), chemical structure of **Val-Si**_**8**_**-Val** (right, top) and **Val-C**_**18**_**-Val** (right, bottom, Figure S19). (C) CLA of the crystal structures of triptycene derivatives that were shown to show the absence or presence of 2D nanostructures.^29,30^ Crystal structures were reproduced with permission from references (31) and (32). Copyright 2015, AAAS and 2007, Elsevier.

Characteristics of the crystalline unit of the materials, such as intermolecular distance and attractive energy, have been shown to be the main driving force for the formation of sheet-like nanostructures. Therefore, we hypothesize that the thermal stability of those ordered phases originates mainly from the strength of the interactions in the crystalline unit. As such, the endothermic transition temperature (*T*_endo_, determined by differential scanning calorimetry, Figures S2B–S20B) should depend on *E*_tot_. Moreover, the observed domain spacings (*d*_exp_) of the materials can be derived from the CLA. To account for different lengths of *o*DMS chains, *E*_tot_ was weighted by φ_non-*o*DMS_. A trend was found between *T*_endo_ and *E*_tot_ · φ_non-*o*DMS_, showing that the thermal stability of the 2D materials decreases with decreasing *E*_tot_ (Figure 5A, left). We then validated our aforementioned approach to predict *d*_exp_ from the results of the CLA. Indeed, *d*_pred_ showed a linear relationship with *d*_exp_ with an error of typically <10% (Figures 4A, right and S21 and Table S3).

Exemplary of the importance of sufficient space for the *o*DMS chains was a telechelic block molecule with *N*-acyl valine as a crystalline unit linked via a pentyl chain to the *o*DMS_8_ chain. This material phase separated into a disordered phase after thermal annealing (Figure S6). CLA predicted correctly that no lamellar material can be formed due to insufficient connecting point distances of 6.6 Å (Figure 5B). We hypothesized that the substitution of the *o*DMS chain (*d* = 7.2 Å) by an alkane chain (*d* = 5.2 Å) would allow the formation of a lamellar material. Thus, we prepared an alkane derivative of the block molecule in which the eight DMS units are replaced by an octyl chain. Indeed, the resulting material formed well-defined lamellae (*d*_exp_ = 48 Å, *d*_pred_ = 30 Å, Figures 5B and S20). This observation indicates the generalizability of the CLA-approach beyond *o*DMS-based materials.

Finally, we tested our CLA approach on a system from the Fukushima lab,^29,30^ in which it was shown that subtle changes in the structure of a telechelic block molecule lead to a morphology change from a lamellar to a disordered system. These block molecules consist of long disperse PDMS chains connected to triptycene units at their 1-position. A triptycene-PDMS material that has only an additional alkoxy group at the 4-position of the crystalline unit (1,4-tripycene) does not form an ordered material. In contrast, a block molecule equipped with a triptycene containing two additional alkoxy groups at the 8- and 13-position (1,8,13-triptycene) phases separates into lamellar structures. To explain this result, we performed CLA on crystal structures of methoxy-substituted 1,4-tripycene and 1,8,13-triptycene (Figure 4C). The absence of sheet-like nanostructures in the 1,4-triptycene block molecule was indeed explained with CLA, due to the absence of layers in the energy framework of the crystal lattice. In contrast, 1,8,13-triptycene showed clear layers in the crystal lattice overlapping with high-energy interactions, explaining the presence of 2D morphologies. The absence and presence of the energetically strong layers within the crystal lattice are thus key to understanding the unexpected properties of Fukushima’s triptycene-containing block molecules.

## Outlook and Conclusion

In conclusion, we herein introduced the crystal lattice analysis workflow to predict the presence and absence of lamellar nanostructures in phase-separated 2D materials. This work highlights the role of crystallinity in the formation of 2D morphologies in such block molecules. A detailed understanding of the magnitude and directionality of the crystalline interactions via crystal lattice analysis can shed light on the morphology and thermal stability of the resulting materials. Since block molecules contain *o*DMS chains in at least one dimension, the use of CLA for the prediction of 3D nanostructures is limited. The design of such complex morphologies requires different tools, such as the recently reported molecularly informed field theory approach.^33^ These in silico predictions of phase-separated materials allow the successful design of multidimensional nanostructures to omit classical trial-and-error-based approaches. Likewise, we envision the insights gained in this work to be powerful in the design of new materials consisting of crystalline moieties with amorphous linkers, which is essential for the development of next-generation (opto-)electronics. Furthermore, this research opens the door to high-throughput screenings of crystal structure databases to greatly accelerate the discovery of new functional phase-separating materials.

## Supporting Information Available

Additional experimental details, materials, and methods as well as MAXS/WAXS patterns and DSC spectra and a more detailed description of the crystal lattice analysis workflow (PDF)

## References

[ref1] Whitesides G. M.; Grzybowski B. Self-Assembly at All Scales. Science 2002, 295 (5564), 2418–2421. 10.1126/science.1070821. 11923529

[ref2] Kory M. J.; Wörle M.; Weber T.; Payamyar P.; Van De Poll S. W.; Dshemuchadse J.; Trapp N.; Schlüter A. D. Gram-Scale Synthesis of Two-Dimensional Polymer Crystals and Their Structure Analysis by X-Ray Diffraction. Nat. Chem 2014, 6 (9), 779–784. 10.1038/nchem.2007. 25143212

[ref3] Colson J. W.; Dichtel W. R. Rationally Synthesized Two-Dimensional Polymers. Nat. Chem 2013, 5 (6), 453–465. 10.1038/nchem.1628. 23695626

[ref4] Gupta A.; Sakthivel T.; Seal S. Recent Development in 2D Materials beyond Graphene. Prog. Mater. Sci 2015, 73, 44–126. 10.1016/j.pmatsci.2015.02.002.

[ref5] Mun J.; Ochiai Y.; Wang W.; Zheng Y.; Zheng Y. Q.; Wu H. C.; Matsuhisa N.; Higashihara T.; Tok J. B.-H.; Yun Y.; Bao Z. A Design Strategy for High Mobility Stretchable Polymer Semiconductors. Nat. Commun 2021, 12 (1), 3572 10.1038/s41467-021-23798-2. 34117254 PMC8196107

[ref6] Noriega R.; Rivnay J.; Vandewal K.; Koch F. P. V.; Stingelin N.; Smith P.; Toney M. F.; Salleo A. A General Relationship between Disorder, Aggregation and Charge Transport in Conjugated Polymers. Nat. Mater 2013, 12 (11), 1038–1044. 10.1038/nmat3722. 23913173

[ref7] Kato T. Self-Assembly of Phase-Segregated Liquid Crystal Structures. Science 2002, 295 (5564), 2414–2418. 10.1126/science.1070967-a. 11923528

[ref8] Oh J. Y.; Rondeau-Gagné S.; Chiu Y. C.; Chortos A.; Lissel F.; Wang G. J. N.; Schroeder B. C.; Kurosawa T.; Lopez J.; Katsumata T.; Xu J.; Zhu C.; Gu X.; Bae W. G.; Kim Y.; Jin L.; Chung J. W.; Tok J. B. H.; Bao Z. Intrinsically Stretchable and Healable Semiconducting Polymer for Organic Transistors. Nature 2016, 539 (7629), 411–415. 10.1038/nature20102. 27853213

[ref9] Zhang C.; Bates M. W.; Geng Z.; Levi A. E.; Vigil D.; Barbon S. M.; Loman T.; Delaney K. T.; Fredrickson G. H.; Bates C. M.; Whittaker A. K.; Hawker C. J. Rapid Generation of Block Copolymer Libraries Using Automated Chromatographic Separation. J. Am. Chem. Soc 2020, 142 (21), 9843–9849. 10.1021/jacs.0c04028. 32421319

[ref10] Isono T.; Komaki R.; Lee C.; Kawakami N.; Ree B. J.; Watanabe K.; Yoshida K.; Mamiya H.; Yamamoto T.; Borsali R.; Tajima K.; Satoh T. Rapid Access to Discrete and Monodisperse Block Co-Oligomers from Sugar and Terpenoid toward Ultrasmall Periodic Nanostructures. Commun. Chem 2020, 3 (1), 135 10.1038/s42004-020-00385-y. 36703322 PMC9814839

[ref11] Yang W.; Zhang W.; Luo L.; Lyu X.; Xiao A.; Shen Z.; Fan X. H. Ordered Structures and Sub-5 Nm Line Patterns from Rod–Coil Hybrids Containing Oligo(Dimethylsiloxane). Chem. Commun 2020, 56 (71), 10341–10344. 10.1039/D0CC04377J. 32760981

[ref12] Van Genabeek B.; De Waal B. F. M.; Gosens M. M. J.; Pitet L. M.; Palmans A. R. A.; Meijer E. W. Synthesis and Self-Assembly of Discrete Dimethylsiloxane-Lactic Acid Diblock Co-Oligomers: The Dononacontamer and Its Shorter Homologues. J. Am. Chem. Soc 2016, 138 (12), 4210–4218. 10.1021/jacs.6b00629. 26999049

[ref13] Van Genabeek B.; Lamers B. A. G.; De Waal B. F. M.; Van Son M. H. C.; Palmans A. R. A.; Meijer E. W. Amplifying (Im)Perfection: The Impact of Crystallinity in Discrete and Disperse Block Co-Oligomers. J. Am. Chem. Soc 2017, 139 (42), 14869–14872. 10.1021/jacs.7b08627. 28994585 PMC5677251

[ref14] Oschmann B.; Lawrence J.; Schulze M. W.; Ren J. M.; Anastasaki A.; Luo Y.; Nothling M. D.; Pester C. W.; Delaney K. T.; Connal L. A.; McGrath A. J.; Clark P. G.; Bates C. M.; Hawker C. J. Effects of Tailored Dispersity on the Self-Assembly of Dimethylsiloxane-Methyl Methacrylate Block Co-Oligomers. ACS Macro Lett 2017, 6 (7), 668–673. 10.1021/acsmacrolett.7b00262. 35650863

[ref15] van den Bersselaar B. W. L.; van de Ven A. P. A.; de Waal B. F. M.; Meskers S. C. J.; Eisenreich F.; Vantomme G. Stimuli-Responsive Nanostructured Viologen-Siloxane Materials for Controllable Conductivity. Adv. Mater 2024, 36 (23), 2312791 10.1002/adma.202312791. 38413048

[ref16] Zha R. H.; Vantomme G.; Berrocal J. A.; Gosens R.; De Waal B.; Meskers S.; Meijer E. W. Photoswitchable Nanomaterials Based on Hierarchically Organized Siloxane Oligomers. Adv. Funct. Mater 2018, 28 (1), 1703952 10.1002/adfm.201703952.

[ref17] Lamers B. A. G.; Graf R.; De Waal B. F. M.; Vantomme G.; Palmans A. R. A.; Meijer E. W. Polymorphism in the Assembly of Phase-Segregated Block Molecules: Pathway Control to 1D and 2D Nanostructures. J. Am. Chem. Soc 2019, 141 (38), 15456–15463. 10.1021/jacs.9b08733. 31483637 PMC6876923

[ref18] Bates F. S. Polymer-Polymer Phase Behavior. Science 1991, 251 (4996), 898–905. 10.1126/science.251.4996.898. 17847383

[ref19] Tschierske C. Liquid Crystal Engineering – New Complex Mesophase Structures and Their Relations to Polymer Morphologies, Nanoscale Patterning and Crystal Engineering. Chem. Soc. Rev 2007, 36 (12), 1930–1970. 10.1039/b615517k. 17982518

[ref20] Tschierske C. 6 Non-conventional soft matter. Annu. Rep. Prog. Chem., Sect. C: phys. Chem 2001, 97 (1), 191–267. 10.1039/b101114f.

[ref21] Zha R. H.; de Waal B.; Lutz M.; Teunissen A. J. P.; Meijer E. W. End Groups of Functionalized Siloxane Oligomers Direct Block Copolymeric or Liquid Crystalline Self-Assembly Behavior. J. Am. Chem. Soc 2016, 138, 5693 10.1021/jacs.6b02172. 27054381 PMC4858755

[ref22] Sijbesma R. P.; Beijer F. H.; Brunsveld L.; Folmer B. J. B.; Hirschberg J. H. K. K.; Lange R. F. M.; Lowe J. K. L.; Meijer E. W. Reversible Polymers Formed from Self-Complementary Monomers Using Quadruple Hydrogen Bonding. Science 1997, 278 (5343), 1601–1604. 10.1126/science.278.5343.1601. 9374454

[ref23] Spackman P. R.; Turner M. J.; Mckinnon J. J.; Wolff S. K.; Grimwood D. J.; Jayatilaka D.; Spackman M. A. CrystalExplorer: A Program for Hirshfeld Surface Analysis, Visualization and Quantitative Analysis of Molecular Crystals. J. Appl. Crystallogr 2021, 54, 1006–1011. 10.1107/S1600576721002910. 34188619 PMC8202033

[ref24] Kamatham N.; Ibraikulov O. A.; Durand P.; Wang J.; Boyron O.; Heinrich B.; Heiser T.; Lévêque P.; Leclerc N.; Méry S. On the Impact of Linear Siloxanated Side Chains on the Molecular Self-Assembling and Charge Transport Properties of Conjugated Polymers. Adv. Funct. Mater 2021, 31 (6), 2007734 10.1002/adfm.202007734.

[ref25] Lamers B. A. G.; van Son M. H. C.; de Graaf F. V.; van den Bersselaar B. W. L.; de Waal B. F. M.; Komatsu K.; Sato H.; Aida T.; Berrocal J. A.; Palmans A. R. A.; Vantomme G.; Meskers S. C. J.; Meijer E. W. Tuning the Donor–Acceptor Interactions in Phase-Segregated Block Molecules. Mater. Horizons 2022, 9 (1), 294–302. 10.1039/D1MH01141C. PMC8725796 34611679

[ref26] Berrocal J. A.; Zha R. H.; De Waal B. F. M.; Lugger J. A. M.; Lutz M.; Meijer E. W. Unraveling the Driving Forces in the Self-Assembly of Monodisperse Naphthalenediimide-Oligodimethylsiloxane Block Molecules. ACS Nano 2017, 11 (4), 3733–3741. 10.1021/acsnano.6b08380. 28380290 PMC5406784

[ref27] Cooper C. B.; Bao Z. Using Periodic Dynamic Polymers to Form Supramolecular Nanostructures. Acc. Mater. Res 2022, 3 (9), 948–959. 10.1021/accountsmr.2c00101.

[ref28] Leibler L. Theory of Microphase Separation in Block Copolymers. Macromolecules 1980, 13 (6), 1602–1617. 10.1021/ma60078a047.

[ref29] Ishiwari F.; Okabe G.; Ogiwara H.; Kajitani T.; Tokita M.; Takata M.; Fukushima T. Terminal Functionalization with a Triptycene Motif That Dramatically Changes the Structural and Physical Properties of an Amorphous Polymer. J. Am. Chem. Soc 2018, 140 (41), 13497–13502. 10.1021/jacs.8b09242. 30281289

[ref30] Chen Y.; Ishiwari F.; Fukui T.; Kajitani T.; Liu H.; Liang X.; Nakajima K.; Tokita M.; Fukushima T. Overcoming the Entropy of Polymer Chains by Making a Plane with Terminal Groups: A Thermoplastic PDMS with a Long-Range 1D Structural Order. Chem. Sci 2023, 14, 2431–2440. 10.1039/D2SC05491D. 36873840 PMC9977418

[ref31] Seiki N.; Shoji Y.; Kajitani T.; Ishiwari F.; Kosaka A.; Hikima T.; Takata M.; Someya T.; Fukushima T. Rational Synthesis of Organic Thin Films with Exceptional Long-Range Structural Integrity. Science 2015, 348 (6239), 1122–1126. 10.1126/science.aab1391. 26045433

[ref32] Yamamura K.; Yamane J.; Eda K.; Tajima F.; Yamada Y.; Hashimoto M. Non-Stoichiometric Quinhydrone-Type CT Complexes: Mixed Crystals of Triptycenequinone and 1,4-Dimethoxytriptycene with Characteristic Color Caused by Local CT Interaction. J. Mol. Struct 2007, 842 (1–3), 12–16. 10.1016/j.molstruc.2006.12.004.

[ref33] Li C.; Murphy E. A.; Skala S. J.; Delaney K. T.; Hawker C. J.; Shell M. S.; Fredrickson G. H. Accelerated Prediction of Phase Behavior for Block Copolymer Libraries Using a Molecularly Informed Field Theory. J. Am. Chem. Soc 2024, 146 (43), 29751–29758. 10.1021/jacs.4c11258. 39420443

