# Supplementary material for: Crystal Lattice Analysis for 2D Nanomorphology Prediction of Phase-Separated Materials
Source: J Am Chem Soc. 2025 Jan 6;147(2):1991–9. doi: 10.1021/jacs.4c14964 (PMC11744751; doi:10.1021/jacs.4c14964)
Supplement: Supplementary file 1 — ja4c14964_si_001.pdf [file ja4c14964_si_001.pdf]

Supporting Information

Crystal lattice analysis for 2D nanomorphology prediction of phase-separated materials

Tobias Schnitzer, [1,2,†] Bart W.L. van den Bersselaar,[1,†] Brigitte A.G. Lamers,[1,†] Martin H.C. van Son,[1] Stefan J.D. Maessen,[1] Freek V. de Graaf,[1] Bas F.M. de Waal,[1] Nils Trapp,[3] Ghislaine Vantomme, [1] E.W. Meijer[1*]

[1]Institute for Complex Molecular Systems, Eindhoven University of Technology; 5600MB, Eindhoven, The Netherlands. [2]Institute of Organic Chemistry, Albert-Ludwigs University Freiburg, Albertstraße 21, 79110 Freiburg im Breisgau, Germany. [3]Small Molecule Crystallography Center, ETH Zürich; Vladimir-Prelog-Weg 3, 8093 Zürich, Switzerland.

[†]These authors contributed equally to the work.

Table of Contents

1. **Materials and Methods** 2
2. **Synthetic procedures** 4
3. **SAXS and DSC characterization** 19
4. **NMR spectra** 41
5. **Crystal Lattice Analysis** 61
6. **Crystal Structure Determination** 88
7. **References** 90

1. Materials and Methods

All chemicals were purchased from commercial sources and used without further purification. The discrete length oligodimethylsiloxanes (*o*DMS) were synthesized according to literature procedure.[1] Dry solvents were obtained with an MBRAUN solvent purification system (MB-SPS). Oven-dried round bottom flasks (120 °C) or flame-dried Schlenk flasks were used for all reactions carried out under argon atmosphere. Reactions were followed by thin-layer chromatography (TLC) using 60-F_{254} silica gel plates from Merck and visualized by UV light at 254 nm or by staining with a ceric ammonium molybdate solution. Automated column chromatography was performed on a Grace Reveleris X2 automated column machine with Reveleris Silica Flash Cartridges.

Nuclear magnetic resonance (NMR) spectra were recorded on Bruker Avance III 400 MHz spectrometers (^{1}H using 400 MHz, ^{13}C using 100 MHz). Peak multiplicities are abbreviated as s: singlet, d: doublet, t: triplet, q: quartet, p: pentet, m: multiplet, dd: doublet of doublets, td: triplet of doublets, dt: doublet of triplets, dq: doublet of quartets, tt: triplet of triplets, ddd: doublet of doublet of doublets and ddt: doublet of doublet of triplets. Proton and carbon chemical shifts are reported in parts per million (ppm) downfield from tetramethylsilane (TMS) using the deuterated solvent resonance frequency as internal standard (7.26 ppm (^{1}H NMR) and 77.16 ppm (^{13}C NMR) for $CDCl_3$, 3.31 ppm (^{1}H NMR) and 49.00 ppm (^{13}C NMR) for Methanol-d_4 and 8.16 ppm (^{1}H NMR) and 162.62 ppm (^{13}C NMR) for DMF-d_7).

Matrix assisted laser desorption/ionization time of flight mass spectroscopy (MALDI-TOF-MS) spectra were acquired using a Bruker Autoflex Speed MALDI-TOF using γ-cyano-4-hydroxycinnamic acid (CHCA) or trans-2-[3-(4-tert-butylphenyl)-2-methyl-2-propenylidene]malononitrile (DCTB) as matrices. All samples were dissolved with a concentration of 1 mg mL^{-1} in chloroform or tetrahydrofuran.

Liquid-Chromatography Mass Spectroscopy (LC-MS) was measured on a device consisting of multiple components: Shimadzu SCL-10 A VP system controller with Shimadzu LC-10AD VP liquid chromatography pumps (with an Alltima C18 3 u (50 × 2.1 mm)) reversed-phase column and gradients of water, a Shimadzu DGU 20A3 prominence degasser, a Thermo Finnigan surveyor auto sampler, a Thermo Finnigan surveyor PDA detector and a Thermo Scientific LCW Fleet.

Differential scanning calorimetry (DSC) data were collected on a DSC Q2000 from TA instruments, calibrated with an indium standard. The samples (4-8 mg) were weighed directly into aluminium pans and hermetically sealed. The samples were initially heated to remove thermal history and then subjected to two cooling/heating cycles with a rate of 10 K min^{-1}. The data that is presented represents the second heating and/or cooling cycle. The thermal transition temperatures were assigned at the maximum of the transition.

Bulk small angle X-ray scattering (SAXS) was performed on a Ganesha instrument from SAXSLab. The flight tube and sample holder are all under vacuum in a single housing, with a GeniX-Cu ultra-low divergence X-ray generator. The source produces X-rays with a wavelength (λ) of 0.154 nm and a flux of 1 × 108 ph s^{-1}. Scattered X-rays were captured on a 2-dimensional Pilatus 300K detector with 487 × 619 pixel resolution. The sample-to-detector distance was 0.084 m (WAXS mode) or 0.48 m (MAXS mode). The measurement time was 300 seconds (WAXS mode) or 1200 seconds (MAXS mode). The instrument was calibrated with diffraction patterns from silver behenate. All room temperature measurements were performed after overnight cooling from their respective isotropic

melt. Domain spacings (d) are calculated using $d = 2\pi/q^*$, with q^* the principal scattering peak.

Volume fractions were estimated as the ratio between the *o*DMS volume and the core volume. For this, the bulk density of PDMS was taken as 0.95 g ml^{-1} which was converted to volume with the respective molecular weights of the eight lengths *o*DMS ($M_{w,CH3Si7H}$ = 519.1 g mol^{-1} gives $V_{CH3Si7H}$ ≈ 552 mL mol^{-1}; $M_{w,HSi8H}$ = 579.3 g mol^{-1} gives V_{HSi8H} ≈ 616 mL mol^{-1}; $M_{w,CH3Si11H}$ = 815.7 g mol^{-1} gives $V_{CH3Si11H}$ ≈ 868 mL mol^{-1}; $M_{w,CH3Si15H}$ = 1112.4 g mol^{-1} gives $V_{CH3Si15H}$ ≈ 1183 mL mol^{-1}; $M_{w,HSi16H}$ = 1172.5 g mol^{-1} gives V_{HSi16H} ≈ 1247 mL mol^{-1}; $M_{w,HSi24H}$ = 1765.7 g mol^{-1} gives V_{HSi24H} ≈ 1878 mL mol^{-1}; $M_{w,HSi32H}$ = 2359 g mol^{-1} gives V_{HSi32H} ≈ 2483 mL mol^{-1} and $M_{w,HSi40H}$ = 2952.2 g mol^{-1} gives V_{HSi40H} ≈ 3141 mL mol^{-1}).[2] The volume of the cores were calculated using the densities of the crystal structures of compounds similar to used cores. The crystal densities were estimated from the crystal structures by dividing the unit cell volume in Å^3 with the number of molecules in the unit cell followed by conversion to mL mol^{-1}. The alkyl chains were accounted for using V_{CH2} ≈ 16 mL mol^{-1} which was estimated by correlating the increase in molar volume for methyl-, ethyl-, propyl-, butyl- and pentyl- naphthalenediimide (Extended data Figure 1, CCDC identifiers: DHAMUX, BIYRIM, DAHLOQ and UNANAZ, RAGJIT).

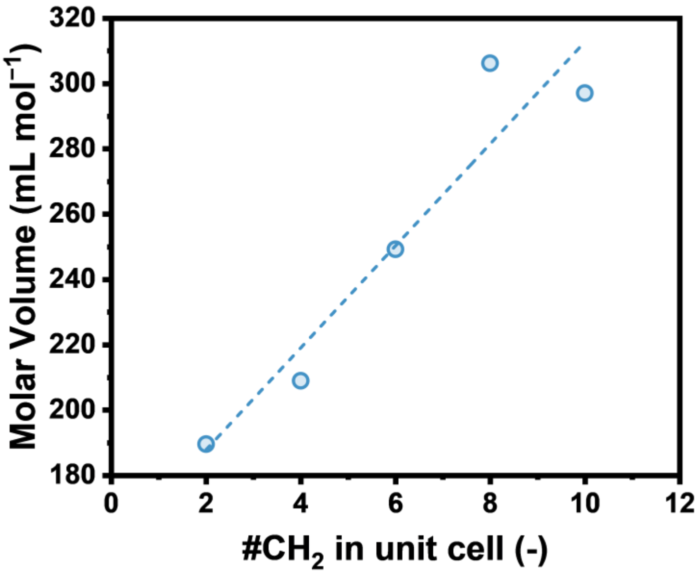

***Figure S1**: Molar volume of naphthalene diimide derivatives as a function of the number of CH_2 groups in their unit cell. From this curve, the slope (16 mL mol^{-1}) was used to account for the varying spacer length in calculating the volume fraction.*

2. Synthetic procedures

General method A for the hydrosilylation reaction

Under argon, *o*DMS$_n$ and the olefin terminated molecule were dissolved in dry DCM and stirred. Subsequently, Karstedt's catalyst (2 wt% in xylene) was added (1-2 drops) and the reaction was stirred until full conversion of the hydride which was monitored using ^{1}H NMR. Hereafter, DCM was removed *in vacuo* and the product was purified using automated column chromatography and dried overnight.

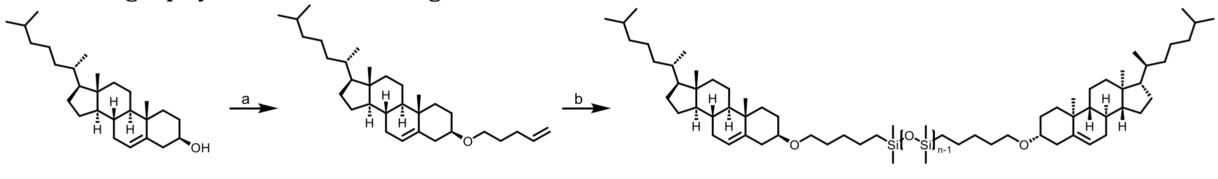

1

Chol-Si$_n$-Chol
n = 8, 40

Scheme S1: *Synthesis of* ***Chol-Si$_n$-Chol****. (a) 1-bromo-pent-4-ene, NaH, NH$_4$Cl, THF, 0 °C – 66 °C, O/N (52%). (b) oDMS$_8$ or oDMS$_{40}$, DCM, Karstedt's catalyst, RT, 2–3 h (20 – 43%).*

Pent-4-en-1-yloxy cholesterol ***(1)***

Cholesterol (0.998 g, 2.59 mmol) was dissolved in 10 mL dry THF and added dropwise to a 100 mL round-bottom flask containing a suspension of NaH (60 wt% in mineral oil) (0.630 g, 15.75 mmol, 6 eq) in 15 mL of dry THF cooled with an ice bath. The mixture was left to stir for 30 minutes, after which 5-bromopent-1-ene (0.45 mL, 4.35 mmol, 1.7 eq) was added. The mixture was left to reflux overnight while stirring and under argon atmosphere. The mixture was cooled down in an ice bath, slowly diluted with 30 mL of saturated NH$_4$Cl solution and transferred to a separatory funnel. The mixture was extracted 3 times with 40 mL of diethyl ether. The organic layers were combined and concentrated *in vacuo* until a yellow solid was obtained. Further purification was performed by automated flash column chromatography using heptane/EtOAc (90/10) as eluent, yielding the pure product after evaporation of the solvent as a wet white solid (0.322 g, 52%). ^{1}H NMR (400 MHz, CDCl$_3$) δ = 5.90 – 5.72 (m, 1H), 5.34 (d, J = 5.3 Hz, 1H), 5.08 – 4.91 (m, 2H), 3.47 (td, J = 6.6, 1.5 Hz, 2H), 3.20 – 3.05 (m, 1H), 2.43 – 0.80 (m, 50H), 0.68 (s, 3H). ^{13}C NMR (100 MHz, CDCl$_3$) δ = 141.14, 138.41, 121.43, 114.64, 79.02, 67.33, 56.81, 56.18, 50.24, 42.34, 39.81, 39.53, 39.23, 37.31, 37.11, 36.92, 36.21, 35.80, 31.97, 31.94, 31.92, 30.38, 30.05, 29.75, 29.71, 29.67, 29.38, 29.33, 28.51, 28.25, 28.03, 24.30, 23.84, 22.82, 22.71, 22.67, 22.57, 21.09, 19.40, 18.73, 14.13, 11.87.

Chol-Si$_8$-Chol (2)

Starting from compound **1** (0.131 g, 0.36 mmol, 2.2 eq) and *o*DMS$_8$ (0.095 g, 0.16 mmol), **Chol-Si$_8$-Chol** was obtained using general method A. The product was purified using automated column chromatography using heptane/DCM (70/30) as eluent. The pure product was obtained after evaporation of the solvent as an opaque wax (0.104 g, 43%). ^{1}H NMR (400 MHz, CDCl$_3$) δ = 5.34 (dt, J = 4.6, 1.9 Hz, 2H), 3.44 (td, J = 6.8, 1.5 Hz, 4H), 3.12 (tt, J = 11.3, 4.3 Hz, 2H), 2.36 (ddd, J = 13.3, 4.8, 2.1 Hz, 2H), 2.26 – 2.11 (m, 2H), 2.08 – 1.75 (m, 10H), 1.62 – 0.83 (m, 78H), 0.68 (s, 6H), 0.54 (dd, J = 9.9, 5.9 Hz, 4H), 0.12 – 0.01 (m, 48H). ^{13}C NMR (100 MHz, CDCl$_3$) δ = 141.20, 121.36, 78.94, 68.11, 56.80, 56.17, 50.23, 42.33, 39.81, 39.53, 39.23, 37.32, 36.92, 36.20, 35.79, 31.97, 31.91, 29.97, 28.50, 28.25, 28.02, 24.30, 23.84, 23.14, 22.83, 22.57, 21.08, 19.39, 18.73, 18.25, 11.87, 1.41, 1.19, 1.09. MS (MALDI-TOF): *m/z* calc. for $C_{80}H_{158}O_9Si_8$: 1487.01; found: 1510.96 Da [M+Na]$^+$ (2nd isotope peak).

Chol-Si$_{40}$-Chol (3)

Starting from compound **1** (0.092 g, 0.20 mmol, 2 eq) and *o*DMS$_{40}$ (0.295 g, 0.10 mmol), **Chol-Si$_{40}$-Chol** was obtained using general method A. The product was purified using automated column chromatography using heptane/DCM (70/30) as eluent. The pure product was obtained after evaporation of the solvent as a colorless oil (0.078 g, 20%). ^{1}H NMR (400 MHz, CDCl$_3$) δ = 5.34 (d, J = 5.7 Hz, 2H), 3.49 – 3.40 (m, 4H), 3.17 – 3.07 (m, 2H), 2.40 – 2.32 (m, 2H), 2.23 – 2.13

(m, 2H), 2.05 – 1.77 (m, 10H), 1.63 – 0.77 (m, 90H), 0.68 (s, 6H), 0.54 (t, J = 7.9 Hz, 4H), 0.10 – 0.04 (m, 240H). ^{13}C NMR (100 MHz, $CDCl_3$) δ = 141.20, 121.36, 78.94, 68.11, 56.81, 50.24, 42.33, 39.81, 37.32, 36.92, 36.20, 35.79, 31.97, 31.91, 29.97, 28.50, 28.24, 28.02, 24.30, 23.83, 23.14, 22.82, 22.57, 21.08, 19.39, 18.72, 18.25, 11.86, 1.41, 1.18, 1.04.

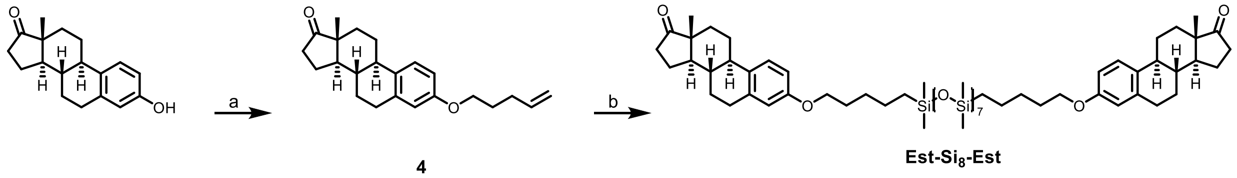

Scheme S2: *Synthesis of* ***Est-Si$_8$-Est****. (a) 1-bromo-pent-4-ene, K_2CO_3, ACN, 82 °C, O/N (81%). (b) oDMS$_8$, DCM, Karstedt's catalyst, RT, 2 h (81%).*

Pent-4-en-1-yloxy estrone ***(4)***

Estrone (0.802 g, 2.97 mmol) and K_2CO_3 (0.654 g, 4.73 mmol, 1.5 eq) were dissolved in 150 mL of anhydrous ACN in a 250 mL round-bottom flask. 5-Bromopent-1-ene (0.568 g, 3.81 mmol, 1.2 eq) was added to the mixture. The mixture was left to reflux overnight while stirring and under argon atmosphere. The mixture was cooled down to room temperature and concentrated *in vacuo*. The mixture was transferred to a separatory funnel and diluted with 20 mL saturated NH_4Cl solution. The mixture was extracted 4 times with 50 mL ethyl acetate. The organic layers were combined, dried over Na_2SO_4, filtered, and concentrated *in vacuo*. Further purification was performed by automated flash column chromatography using heptane/EtOAc (90/10) as eluent, yielding the pure product after evaporation of the solvent as a white solid (0.809 g, 81%). ^{1}H NMR (400 MHz, $CDCl_3$) δ = 7.19 (d, J = 8.6 Hz, 1H), 6.71 (d, J = 8.6 Hz, 1H), 6.65 (s, 1H), 5.85 (td, J = 16.9, 6.6 Hz, 1H), 5.13 – 4.94 (m, 2H), 3.94 (t, J = 6.4 Hz, 2H), 2.97 – 2.82 (m, 2H), 2.50 (dd, J = 18.8, 8.6 Hz, 1H), 2.39 (d, J = 9.8 Hz, 1H), 2.33 – 1.80 (m, 10H), 1.71 – 1.37 (m, 7H), 0.91 (s, 3H). ^{13}C NMR (100 MHz, $CDCl_3$) δ = 157.08, 137.90, 137.71, 131.92, 126.30, 115.12, 114.57, 112.14, 67.10, 50.43, 48.03, 44.01, 38.40, 35.89, 31.61, 30.15, 29.67, 28.52, 26.58, 25.94, 21.61, 13.87. MS (MALDI-TOF): *m/z* calc. for $C_{23}H_{30}O_2$ 338.22; found 338.38 Da [M]$^+$.

Est-Si$_8$-Est (5)

Starting from compound **4** (0.131 g, 0.39 mmol, 2.2 eq) and *o*DMS$_8$ (0.104 g, 0.18 mmol), **Est-Si$_8$-Est** was obtained using general method A. The product was purified using automated column chromatography using DCM as eluent. The pure product was obtained after evaporation of the solvent as a glassy solid (0.183 g, 81%). ^{1}H NMR (400 MHz, $CDCl_3$) δ = 7.19 (d, J = 8.5 Hz, 2H), 6.70 (d, J = 8.6 Hz, 2H), 6.64 (s, 2H), 3.92 (t, J = 6.6 Hz, 4H), 2.89 (d, J = 10.7 Hz, 4H), 2.50 (dd, J = 18.8, 8.6 Hz, 2H), 2.39 (d, J = 9.8 Hz, 2H), 2.29 – 1.92 (m, 10H), 1.82 – 1.71 (m, 4H), 1.68 – 1.29 (m, 22H), 0.91 (s, 6H), 0.65 – 0.50 (m, 4H), 0.08 (m, 48H). ^{13}C NMR (100 MHz, $CDCl_3$) δ = 157.17, 137.65, 131.79, 126.26, 114.54, 112.12, 67.91, 50.43, 48.02, 44.00, 38.41, 35.88, 31.61, 29.78, 29.66, 29.11, 26.59, 25.94, 23.08, 21.60, 18.22, 13.86, 1.20, 1.09, 0.20. MS (MALDI-TOF): *m/z* calc. for $C_{62}H_{110}O_{11}Si_8$ 1254.62; found 1277.63 Da [M+Na]$^+$.

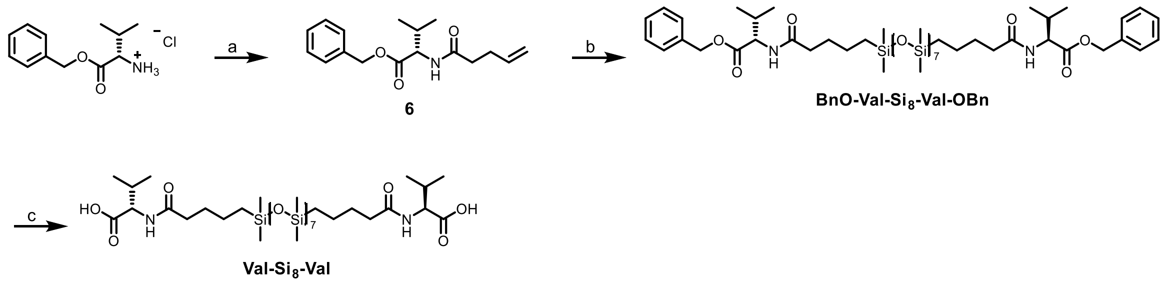

Scheme S3: *Synthesis of **Val-Si$_8$-Val**. (a) 4-Pentenoyl chloride, Et_3N, DCM, RT, 16 h (85%). (b) oDMS$_8$, DCM, Karstedt's catalyst, RT, 2 h (83%). (c) Pd/C, H_2, MeOH, O/N (82%).*

Benzyl pent-4-enoyl-L-valinate ***(6)***

Benzyl-L-valinate hydrochloride (0.515 g, 2.11 mmol) was dissolved in 20 mL of dry DCM in a 100 mL round-bottom flask. Triethylamine (0.900 mL, 6.46 mmol, 3 eq) was added to the solution and the mixture was left to stir for 30 minutes. 4-pentenoyl chloride (0.376 mL, 3.41 mmol, 1.2 eq) was added to the mixture and it was left to stir under argon atmosphere for 16 hours. The solvent was evaporated *in vacuo* until a yellow oil was obtained. The oil was dissolved in 50 mL of ethyl acetate and transferred to a separatory funnel. The organic layer was washed 3 times with 20 mL of water. The organic layer was concentrated *in vacuo* and dried over the weekend in a vacuum oven at 60°C, yielding the pure product as a yellow oil (0.518 g, 85%). ^1H NMR (400 MHz, $CDCl_3$) δ = 7.35 (s, 5H), 5.95 (d, J = 8.8 Hz, 1H), 5.89 – 5.74 (m, 1H), 5.17 (q, J = 12.2 Hz, 2H), 5.05 (dd, J = 26.3, 13.8 Hz, 2H), 4.64 (dd, J = 7.8, 4.2 Hz, 1H), 2.48 – 2.26 (m, 4H), 2.17 (q, J = 6.7 Hz, 1H), 0.88 (dd, J = 22.9, 6.9 Hz, 6H). ^{13}C NMR (100 MHz, $CDCl_3$) δ = 172.16, 172.04, 136.92, 135.29, 128.60, 128.47, 128.37, 115.71, 67.05, 56.87, 35.80, 31.35, 29.52, 18.97, 17.66, 1.02. MS (MALDI-TOF): *m/z* calc. for $C_{17}H_{23}NO_3$ 289.17; found 290.19 Da $[M+H]^+$, 312.17 Da $[M+Na]^+$, 328.15 $[M+K]^+$.

BnO-Val-Si$_8$-Val-OBn (7)

Starting from compound **6** (0.518 g, 1.79 mmol, 2.2 eq) and *o*DMS$_8$ (0.472 g, 0.81 mmol), **BnO-Val-Si$_8$-Val-OBn** was obtained using general method A. The product was purified using automated column chromatography using heptane/EtOAc (50/50) as eluent. The pure product was obtained after evaporation of the solvent as a colorless oil (0.785 g, 83%). ^1H NMR (400 MHz, $CDCl_3$) δ = 7.42 – 7.29 (m, 10H), 5.91 (d, J = 8.8 Hz, 2H), 5.24 – 5.09 (m, 4H), 4.64 (dd, J = 8.8, 4.7 Hz, 2H), 2.29 – 2.11 (m, 6H), 1.67 (m, J = 10.7, 7.1, 3.4 Hz, 4H), 1.44 – 1.30 (m, 4H), 0.88 (dd, J = 23.4, 6.9 Hz, 12H), 0.59 – 0.50 (m, 4H), 0.10 – 0.02 (m, 48H). ^{13}C NMR (100 MHz, $CDCl_3$) δ = 172.99, 172.12, 135.31, 128.60, 128.46, 128.36, 67.03, 56.75, 36.56, 31.39, 29.37, 23.03, 18.97, 18.04, 17.65, 1.19, 1.09, 0.14. MS (MALDI-TOF): *m/z* calc. for $C_{50}H_{96}N_2O_{13}Si_8$ 1156.51; found 1179.53 Da $[M+Na]^+$.

Val-Si$_8$-Val (8)

BnO-Val-Si$_8$-Val-OBn (7) (0.437 g, 0.38 mmol) was dissolved in 16 mL of methanol in a 50 mL round-bottom flask. The solution was bubbled with nitrogen gas for 15 minutes. Pd/C (10 wt%) (0.025 g) was added to the solution. The flask was equipped with a septum and a balloon containing hydrogen gas. The mixture was brought to a H_2 atmosphere and was stirred overnight. The mixture was filtered over celite, and the filtrate was concentrated under reduced pressure until an opaque wax was obtained. The opaque wax was dispersed in 20 mL of toluene and decanted 3 times. The pure product was obtained after drying as a colorless wax (0.304 g, 82%). ^1H NMR (400 MHz, Methanol-d_4) δ = 4.31 (d, J = 5.6 Hz, 2H), 2.28 (td, J = 7.3, 2.8 Hz, 4H), 2.16 (m, J = 6.7 Hz, 2H), 1.65 (m, J = 7.4, 5.4 Hz, 4H), 1.47 – 1.32 (m, 4H), 0.96 (dd, J = 6.8, 4.3 Hz, 12H), 0.68 – 0.52 (m, 4H), 0.11 – 0.04 (m, 48H). ^{13}C NMR (100 MHz, $CDCl_3$+ 2 drops of Methanol-d_4) δ = 174.01, 173.94, 57.13, 36.43, 31.02, 29.47, 23.06, 19.05, 18.07, 17.70, 1.18, 1.06, 0.11.

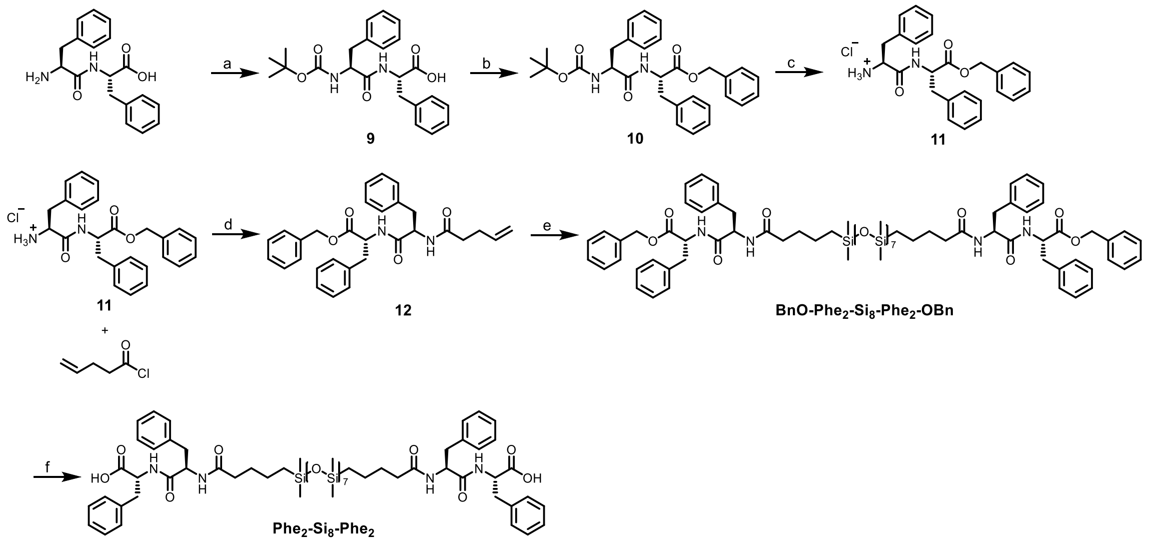

Scheme S4: *Synthesis of **Phe$_2$-Si$_8$-Phe$_2$**. (a) Boc$_2$O, Na$_2$CO$_3$, H$_2$O: Dioxane (1: 1), RT, 3 h (78%). (b) BzOH, HATU, dry DMF, RT, 3 h (75%). (c) 4 M HCl in Dioxane, RT, 3 h (99%). (d) 4-Pentenoyl chloride, Et$_3$N, DCM, RT, 16 h (63%). (e) oDMS$_8$, DCM, Umicore HS432, RT, 48 h (83%). (f) Pd/C, H$_2$, MeOH, O/N (23%).*

*(tert-butoxycarbonyl)-L-phenylalanyl-L-phenylalanine **(9)***

L-phenylalanyl-L-phenylalanine (1.030 g, 3.30 mmol), Boc$_2$O (1.085 g, 4.95 mmol, 1.5 eq) and Na$_2$CO$_3$ (1.109 g, 10.46 mmol, 3 eq) were dissolved in 60 mL of a 1: 1 mixture of water and 1,4-dioxane in a 250 mL round-bottom flask and stirred at room temperature for three hours. The mixture was transferred to a separatory funnel and extracted once with 40 mL ethyl acetate. The aqueous layer was acidified using concentrated HCl until pH 3 was obtained. The aqueous layer was extracted 4 times with 20 mL of ethyl acetate. The combined organic layers were dried over MgSO$_4$ and filtered. The pure product was obtained after evaporation of the solvent as a white solid (1.060 g, 78%). ^{1}H NMR (400 MHz, Methanol-*d$_4$*) δ = 7.32 – 7.13 (m, 10H), 4.75 – 4.54 (m, 1H), 4.28 (dd, *J* = 9.6, 5.0 Hz, 1H), 3.19 (dd, *J* = 13.9, 5.4 Hz, 1H), 3.09 – 2.93 (m, 2H), 2.72 (dd, *J* = 13.9, 9.6 Hz, 1H), 1.34 (s, 9H). ^{13}C NMR (100 MHz, Methanol-*d$_4$*) δ = 174.11, 157.46, 138.60, 138.09, 130.42, 130.31, 129.43, 129.33, 127.79, 127.60, 80.61, 57.28, 54.87, 39.15, 38.45, 28.63.

*Benzyl (tert-butoxycarbonyl)-L-phenylalanyl-L-phenylalaninate **(10)***

9 (1.060 g, 2.57 mmol) was dissolved in 10 mL of dry DMF in a 50 mL round-bottom flask. *N,N*-Diisopropylethylamine (0.7 mL, 4.02 mmol, 1.5 eq) was added to the solution. HATU (1.072 g, 2.82 mmol, 1.1 eq) was added to the mixture and it was left to stir for 15 minutes. Benzyl alcohol (0.3 mL, 2.89 mmol, 1.1 eq) was added to the mixture and it was left to stir overnight. The mixture was diluted with 50 mL water and transferred to a separatory funnel, where it was extracted 3 times with 40 mL ethyl acetate. The combined organic layers were washed 3 times with 60 mL water and once with 60 mL brine, respectively. The mixture was dried over Na$_2$SO$_4$, filtered and the solvent was evaporated *in vacuo*. Further purification was performed by automated flash column chromatography using heptane/EtOAc (80/20) as eluent, yielding the pure product after evaporation of the solvent as an opaque solid (0.971 g, 75%). ^{1}H NMR (400 MHz, CDCl$_3$) δ = 7.40 – 7.12 (m, 13H), 6.87 (ddd, *J* = 19.9, 7.2, 2.1 Hz, 2H), 6.33 (dd, *J* = 34.6, 7.7 Hz, 1H), 5.15 – 5.02 (m, 2H), 5.01 – 4.77 (m, 2H), 4.34 (s, 1H), 3.12 – 2.85 (m, 4H), 1.38 (d, *J* = 7.1 Hz, 9H). ^{13}C NMR (100 MHz, CDCl$_3$) δ = 170.91, 170.83, 170.77, 170.73, 136.62, 136.50, 135.49, 135.38, 135.04, 135.02, 129.36, 129.32, 129.29, 129.23, 128.67, 128.61, 128.60, 128.57, 128.55, 128.52, 127.10, 127.06, 126.98, 67.22, 53.32, 53.06, 37.95, 37.87, 30.94, 28.25. MS (MALDI-TOF): *m/z* calc. for $C_{30}H_{34}N_2O_5$ 502.25; found 525.26 Da $[M+Na]^+$

*Benzyl L-phenylalanyl-L-phenylalaninate hydrochloride **(11)***

10 (0.971 g, 1.93 mmol) was dissolved in 4 mL of 4 M HCl in 1,4-dioxane in a 50 mL round-bottom flask. The mixture was stirred for 3 hours after which the solvent was evaporated *in vacuo* until a yellow solid was obtained. The solid was dispersed in 20 mL diethyl ether, centrifuged, and

decanted for a total of 3 times, yielding the pure product after drying as a yellow solid (0.841 g, 99%). ^{1}H NMR (400 MHz, Methanol-d_4) δ = 7.40 – 7.02 (m, 15H), 5.22 – 5.05 (m, 2H), 4.76 (ddd, *J* = 9.0, 6.1, 2.7 Hz, 1H), 4.12 (ddd, *J* = 10.3, 8.3, 5.6 Hz, 1H), 3.26 – 2.75 (m, 4H). ^{13}C NMR (100 MHz, Methanol-d_4) δ = 171.06, 168.17, 136.35, 134.02, 133.85, 129.19, 129.14, 128.95, 128.87, 128.72, 128.70, 128.31, 128.26, 128.24, 128.21, 128.12, 128.10, 128.08, 127.46, 126.76, 126.65, 66.87, 66.86, 54.31, 54.02, 54.00, 37.15, 36.96.

Benzyl pent-4-enoyl-L-phenylalanyl-L-phenylalaninate ***(12)***

11 (0.321 g, 0.73 mmol) was dissolved in 20 mL of dry DCM in a 100 mL round-bottom flask. Triethylamine (0.300 mL, 2.15 mmol, 3 eq) was added to the solution and the mixture was left to stir for 30 minutes. 4-pentenoyl chloride (0.120 mL, 1.09 mmol, 1.5 eq) was added to the mixture and it was left to stir under argon atmosphere for 16 hours. The solvent was evaporated *in vacuo* until a yellow solid was obtained. Purification was performed by automated flash column chromatography using heptane/EtOAc (90/10) as eluent, yielding the pure product after evaporation of the solvent as a white solid (0.223 g, 63%). ^{1}H NMR (400 MHz, $CDCl_3$) δ = 7.44 – 6.78 (m, 15H), 6.25 (dd, *J* = 42.2, 7.8 Hz, 1H), 5.99 (dd, *J* = 15.5, 7.7 Hz, 1H), 5.71 (m, *J* = 16.8, 10.3, 8.3, 6.3 Hz, 1H), 5.17 – 4.89 (m, 4H), 4.80 (ddt, *J* = 20.9, 7.6, 6.0 Hz, 1H), 4.65 (dq, *J* = 14.1, 7.2 Hz, 1H), 3.13 – 2.85 (m, 4H), 2.36 – 2.13 (m, 4H). ^{13}C NMR (100 MHz, $CDCl_3$) δ = 172.16, 172.10, 170.81, 170.66, 170.49, 170.40, 136.70, 136.44, 136.35, 135.43, 135.36, 135.02, 129.32, 129.31, 129.23, 128.70, 128.65, 128.63, 128.62, 128.60, 128.58, 128.55, 128.53, 127.16, 127.09, 127.05, 115.73, 67.28, 67.25, 54.25, 54.12, 53.44, 53.14, 38.19, 38.09, 37.82, 37.79, 35.57, 35.55, 29.32, 29.30. MS (MALDI-TOF): *m/z* calc. for $C_{30}H_{32}N_2O_4$ 484.24; found 507.17 Da $[M+Na]^+$, 523.15 $[M+K]^+$.

BnO-Phe$_2$-Si$_8$-Phe$_2$-OBn (13)

Starting from compound **12** (0.219 g, 0.45 mmol, 1.9 eq) and *o*DMS$_8$ (0.135 g, 0.22 mmol) were dissolved in 6 mL of dry DCM in an oven dried 50 mL round-bottom flask. Umicore HS432 (30 mg) was added to the mixture, and it was left to stir under argon atmosphere over the weekend. The DCM was evaporated, and the product was purified using automated column chromatography using chloroform/diethyl ether (gradient 100/0 to 0/100) as eluent. The pure product was obtained after evaporation of the solvent as an opaque wax (0.290 g, 83%). ^{1}H NMR (400 MHz, $CDCl_3$) δ = 7.41 – 6.78 (m, 30H), 6.23 (dt, *J* = 40.2, 7.8 Hz, 2H), 6.06 – 5.84 (m, 2H), 5.14 – 5.01 (m, 4H), 4.86 – 4.71 (m, 2H), 4.71 – 4.56 (m, 2H), 3.10 – 2.83 (m, 8H), 2.17 – 2.01 (m, 4H), 1.62 – 1.46 (m, 4H), 1.29 (dq, *J* = 15.1, 7.4 Hz, 4H), 0.51 (dt, *J* = 12.6, 4.9 Hz, 4H), 0.06 (dd, *J* = 4.9, 2.1 Hz, 48H). ^{13}C NMR (100 MHz, $CDCl_3$) δ = 172.94, 172.90, 172.88, 170.79, 170.64, 170.55, 170.47, 170.45, 136.48, 136.40, 135.42, 135.38, 135.02, 129.30, 129.21, 128.66, 128.61, 128.59, 128.57, 128.55, 128.52, 128.49, 127.12, 127.06, 127.01, 67.23, 67.20, 54.21, 54.06, 53.43, 53.13, 38.26, 38.13, 37.84, 37.79, 36.30, 36.27, 29.17, 29.13, 23.02, 23.00, 18.00, 1.20, 1.11, 1.10.

Phe$_2$-Si$_8$-Phe$_2$ (14)

13 (0.195 g, 0.13 mmol) was dissolved in 15 mL of methanol in a 50 mL round-bottom flask. The solution was bubbled with nitrogen gas for 15 minutes. Pd/C (10 wt%) (0.015 g) was added to the solution. The flask was equipped with a septum and a balloon containing hydrogen gas. The mixture was brought to a H_2 atmosphere and was stirred overnight. The mixture was filtered over celite, and the filtrate was concentrated under reduced pressure until a colorless oil was obtained. Further purification was done using automated column chromatography using ethyl acetate as eluent. The obtained suspension in the first fraction of the column was decanted 3 times with 20 mL ethyl acetate. The pure product was obtained after drying as a white solid (0.040 g, 23%). ^{1}H NMR (400 MHz, Methanol-d_4 + drop of TFA added) δ = 7.29 – 7.05 (m, 20H), 4.70 – 4.60 (m, 4H), 3.23 – 2.62 (m, 8H), 2.15 – 2.04 (m, 4H), 1.46 (p, *J* = 7.6 Hz, 4H), 1.26 (p, *J* = 7.6, 7.2 Hz, 4H), 0.60 – 0.44 (m, 4H) 0.16 – 0.02 (m, 48H). ^{13}C NMR (100 MHz, Methanol-d_4 + drop of TFA added) δ = 160.59, 134.52, 129.02, 128.93, 127.69, 116.21, 115.35, 113.40, 56.44, 53.49, 37.03, 35.49, 22.84, 21.92, 17.73, 1.09, 1.04, 0.97, 0.86, 0.63, 0.03.

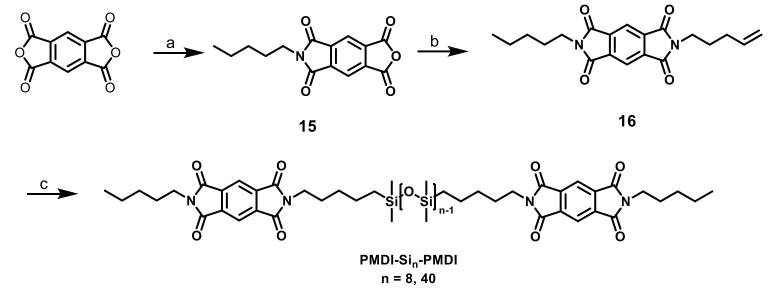

Scheme S5: *Synthesis of* ***PMDI-Si$_n$-PMDI****. (a) 1. Amylamine, DMF, 110 °C, O/N, 2. AcOH, 80 C, 72 h (27%). (b) 1-amino-pent-4-ene, DMF, Microwave, 110 °C, 30 min (69%). (c) oDMS$_8$ or oDMS$_{40}$, DCM, Karstedt's catalyst, RT, O/N (27 - 78%).*

6-pentyl-1H-furo[3,4-f]isoindole-1,3,5,7(6H)-tetraone ***(C$_5$-PMMI) (15)***

PMDA (1.063 g, 4.87 mmol) was dissolved in 20 mL of DMF in a 100 mL round-bottom flask. The solution was heated to 80 °C, after which amylamine (0.6 mL, 5.20 mmol, 1.1 eq) dissolved in 15 mL of DMF was added dropwise to the mixture. The mixture was left to stir overnight at 110 °C while stirring and under argon atmosphere. The mixture was cooled down to 0 °C after which a white precipitate was formed, which was filtered off and kept separately, as this was pure **C$_5$-PMDI-C$_5$**. The mixture was concentrated *in vacuo*. The mixture was dissolved in DCM, resulting in the formation of white precipitate which was filtered off. Further purification was performed by automated flash column chromatography using DCM/EtOAc (gradient 100/0 to 0/100) as eluent, yielding a slightly yellow oil. The oil was dissolved in 40 mL acetic anhydride and stirred at 80 °C for 72 hours. The product was obtained after evaporation of the solvent as a brown solid (0.373 g, 27%). ^{1}H NMR (400 MHz, $CDCl_3$ + several drops of TFA) δ = 8.50 – 8.28 (m, 2H), 3.77 (dt, J = 12.1, 7.3 Hz, 2H), 1.79 – 1.63 (m, 2H), 1.44 – 1.24 (m, 4H), 0.91 (t, J = 7.0 Hz, 3H). ^{13}C NMR (100 MHz, $CDCl_3$ + several drops of TFA) δ = 170.79, 166.88, 166.77, 165.75, 138.72, 137.25, 136.30, 134.83, 124.58, 120.79, 118.58, 115.77, 39.29, 39.04, 28.91, 28.08, 28.03, 22.20, 22.18, 13.87. **C$_5$-PMDI-C$_5$** characterization: ^{1}H NMR (400 MHz, $CDCl_3$) δ = 8.24 (s, 2H), 3.71 (t, J = 7.4 Hz, 4H), 1.69 (q, J = 7.6 Hz, 4H), 1.40 – 1.24 (m, 8H), 0.87 (t, J = 6.9 Hz, 6H). ^{13}C NMR (100 MHz, $CDCl_3$) δ = 166.31, 137.25, 118.09, 38.72, 28.94, 28.12, 22.22, 13.92.

2-(pent-4-en-1-yl)-6-pentylpyrrolo[3,4-f]isoindole-1,3,5,7(2H,6H)-tetraone ***(16)***

C$_5$-PMMI (0.220 g, 0.77 mmol) was dissolved in 4 mL of DMF in a 5 mL microwave reaction vessel. 4-Pentene-1-amine (0.123 mL, 1.15 mmol, 1.5 eq) was added to the vessel. The mixture was reacted in the microwave for 30 minutes at 110 °C while stirring. The mixture was concentrated *in vacuo*. Further purification was performed by automated flash column chromatography using DCM as eluent, yielding the product after evaporation of the solvent as a white solid (0.188 g, 69%). ^{1}H NMR (400 MHz, $CDCl_3$) δ = 8.27 (s, 2H), 5.88 – 5.73 (m, 1H), 5.12 – 4.94 (m, 2H), 3.79 – 3.71 (m, 4H), 2.21 – 2.07 (m, 2H), 1.82 (p, J = 7.4 Hz, 2H), 1.71 (p, J = 7.3 Hz, 2H), 1.43 – 1.27 (m, 4H), 0.90 (t, J = 7.0 Hz, 3H). ^{13}C NMR (100 MHz, $CDCl_3$) δ = 166.32, 166.30, 166.27, 137.27, 137.24, 137.21, 136.97, 118.18, 118.15, 118.13, 115.59, 38.75, 38.26, 30.94, 28.93, 28.12, 27.42, 22.21, 13.91.

PMDI-Si$_8$-PMDI (17)

Starting from compound **16** (0.100 g, 0.28 mmol, 2.2 eq) and *o*DMS$_8$ (0.073 g, 0.13 mmol), **PMDI-Si$_8$-PMDI** was obtained using general method A. The product was purified using automated column chromatography using DCM/heptane (gradient 80/20 to 100/0). The pure product was obtained after evaporation of the solvent as a white solid (0.126 g, 78%). ^{1}H NMR (400 MHz, $CDCl_3$) δ = 8.20 (s, 4H), 3.73 – 3.61 (m, 8H), 1.73 – 1.57 (m, 8H), 1.40 – 1.19 (m, 16H), 0.84 (t, J = 6.9 Hz, 6H), 0.53 – 0.40 (m, 4H). ^{13}C NMR (100 MHz, $CDCl_3$) δ = 166.31, 166.27, 137.25, 137.23, 118.09, 38.72, 30.53, 28.93, 28.17, 28.12, 22.81, 22.21, 18.12, 13.91, 1.18, 1.08.

PMDI-Si$_{40}$-PMDI (18)

Starting from compound **17** (0.060 g, 0.17 mmol, 2.3 eq) and *o*DMS$_{40}$ (0.216 g, 0.07 mmol), **PMDI-Si$_{40}$-PMDI** was obtained using general method A. The product was purified using

automated column chromatography using DCM/heptane (gradient 80/20 to 100/0). The pure product was obtained after evaporation of the solvent as a waxy white solid (0.069 g, 27%). ^{1}H NMR (400 MHz, $CDCl_3$) δ = 8.26 (s, 4H), 3.73 (td, J = 7.4, 2.8 Hz, 8H), 1.69 (p, J = 7.7 Hz, 8H), 1.45 – 1.25 (m, 16H), 0.90 (t, J = 6.9 Hz, 6H), 0.53 (t, J = 7.8 Hz, 4H), 0.07 (s, 240H). ^{13}C NMR (100 MHz, $CDCl_3$) δ = 166.31, 166.27, 137.24, 118.09, 38.72, 30.53, 28.93, 28.12, 22.81, 22.21, 18.12, 13.91, 1.17, 1.04, 0.14.

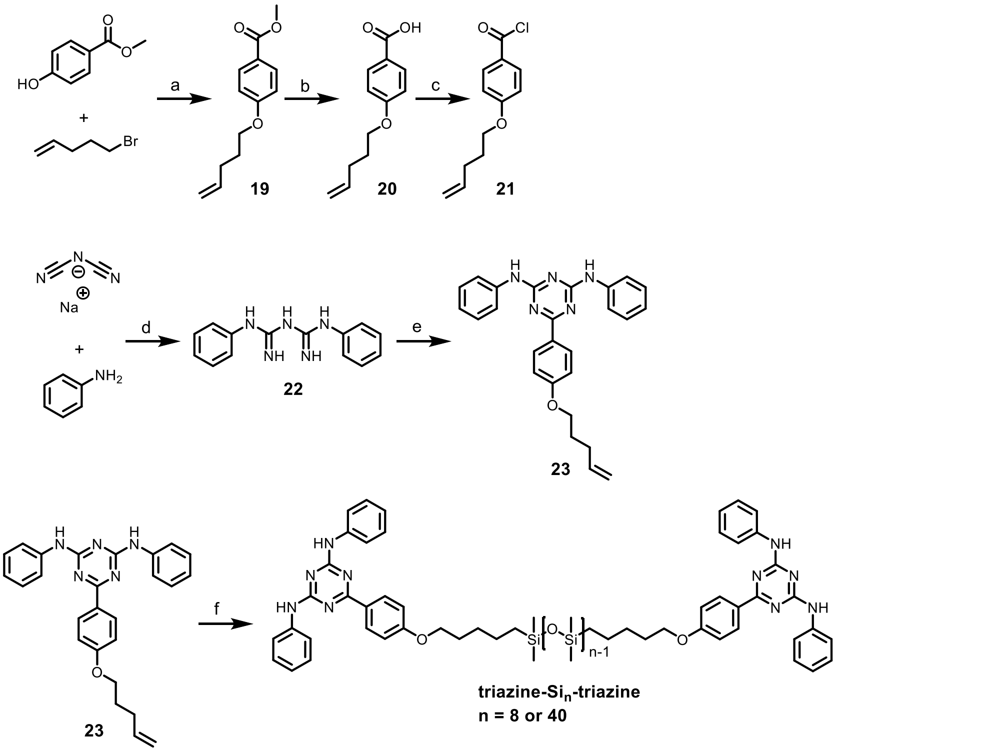

Scheme S6: *Synthesis of* ***triazine-Si$_n$-triazine****. Reaction conditions: (a) K_2CO_3, KI, acetone, 65 °C, O/N (57%); (b) KOH, ethanol, reflux, O/N (87%); (c) $SOCl_2$, dry $CHCl_3$, argon, reflux, 3 h (90%); (d) 1) 1M HCl, RT, 15 min; 2) sodium dicyanamide (**6**), reflux, O/N (44%); (e) Et_3N, **5**, DMAc, $CHCl_3$, 50 °C, O/N (33%); (f) oDMS$_8$ or oDMS$_{40}$, DCM, Karstedt's catalyst, RT, 2–3 h (40–46%).*

*Methyl 4-(pent-4-en-1-yloxy)benzoate (**19**)*

Methyl 4-hydroxybenzoate (1.73 g, 11.4 mmol), K_2CO_3 (3.14 g, 22.8 mmol, 2 eq) and KI (0.19 g, 1.14 mmol, 0.1 eq) were dissolved in acetone. 5-bromo-1-pentene (2.53 g, 17.0 mmol, 1.5 eq) was added and the mixture was stirred overnight at 60 °C. The crude product was transferred to a separation funnel and extracted with pentane (4 × 100 mL). The organic layers were collected and washed with 50 mL HCl (1M) and 100 mL brine. The organic layer was collected and dried using $MgSO_4$. The $MgSO_4$ was filtered off and the filtrate was concentrated in vacuo to a volume of ~10 mL. The product was purified using automated column chromatography with heptane/EtOAc (gradient 100/0 to 85/15) as eluent. The product was dried in vacuo, yielding pure methyl 4-(pent-4-en-1-yloxy)benzoate **20** (1.42 g, 57%). ^{1}H NMR (400 MHz, $CDCl_3$) δ = 7.97 (d, J = 8.4 Hz, 2H), 6.91 (d, J = 8.4 Hz, 2H), 5.91 – 5.79 (m, 1H), 5.19 – 4.93 (m, 2H), 4.02 (t, J = 6.4 Hz, 2H), 3.88 (s, 3H), 2.24 (q, J = 6.6 Hz, 2H), 1.91 (dt, J = 8.1, 6.5 Hz, 2H); ^{13}C NMR (100 MHz, $CDCl_3$) δ = 166.88, 162.84, 137.56, 131.56, 122.42, 115.38, 114.06, 67.32, 51.82, 30.02, 28.26 ppm.

*4-(Pent-4-en-1-yloxy)benzoic acid (**20**)*

Methyl 4-(pent-4-en-1-yloxy)benzoate (**19**) (1.37 g, 6.23 mmol) and KOH (0.70 g, 12.5, 2 eq) were dissolved in 30 mL ethanol and stirred at 80 °C overnight. Concentrated HCl (37 %) was added dropwise to the hot solution until pH 7 was reached. Subsequently, the mixture was poured in 100 mL water and extracted with diethyl ether (3 × 100 mL). Separation of the aqueous and organic layer was improved by addition of NaCl. The organic layers were collected, combined and washed with water. The organic layer was dried using $MgSO_4$, filtered and the filtrate was dried

in vacuo, yielding the pure product **21** as a light-yellow solid (1.12 g, 87%). ^1H NMR (400 MHz, $CDCl_3$) δ = 12.12 (s, 1H), 8.06 (d, J = 8.4 Hz, 2H), 6.94 (d, J = 8.4 Hz, 2H), 5.86 (ddt, J = 16.9, 10.2, 6.6 Hz, 1H), 5.20 – 4.94 (m, 2H), 4.04 (t, J = 6.4 Hz, 2H), 2.25 (q, J = 6.6, 3.4 Hz, 2H), 1.93 (dt, J = 8.1, 6.5 Hz, 2H); ^{13}C NMR (100 MHz, $CDCl_3$) δ = 172.23, 163.60, 137.52, 132.36, 121.54, 115.42, 114.20, 67.40, 30.01, 28.22 ppm.

*4-(Pent-4-en-1-yloxy)benzoyl chloride (**21**)*

4-(Pent-4-en-1-yloxy)benzoic acid (**20**) (0.37 g, 1.77 mmol) was dissolved in 10 mL chloroform and while stirring under argon, thionyl chloride (0.3 mL, 3.54 mmol, 2 eq) was added dropwise. The mixture was stirred for 3 h under reflux conditions. After full conversion, the chloroform and thionyl chloride were distilled off by vacuum distillation. The resulting brown oil was dissolved in heptane, forming a precipitate. The precipitate was filtered and the filtrate was dried in vacuo, yielding the benzoyl chloride product **22** as light-yellow solid (0.36 g, 90%). ^1H NMR (400 MHz, $CDCl_3$) δ = 8.08 (d, J = 8.4 Hz, 2H), 6.97 (d, J = 8.4 Hz, 2H), 5.94 – 5.76 (m, 1H), 5.15 – 4.98 (m, 2H), 4.06 (t, J = 6.4 Hz, 2H), 2.25 (q, J = 7.1 Hz, 2H), 1.93 (p, J = 6.8 Hz, 2H); ^{13}C NMR (100 MHz, $CDCl_3$) δ = 172.03, 163.58, 137.51, 132.33, 121.50, 115.57, 114.64, 67.41, 30.00, 28.21 ppm.

*1,5-Diphenylbiguanide (**22**)*

Aniline (0.93 g, 10 mmol, 2 eq) was added to a 1M HCl solution and stirred until the mixture was homogeneous. Sodium dicyanamide (0.44 g, 5 mmol) was added dropwise and the mixture was stirred under reflux overnight. The mixture was allowed to cool to room temperature and precipitate was filtered and washed with water. The precipitate was collected and dried in vacuo. The crude product was dissolved in 3 mL hot DMF after which 15 mL toluene was added, forming a gel-like precipitate. The mixture was filtered and the precipitate with was washed with toluene (20 mL) and water (20 mL). The residue was dried in vacuo. The crude product was dissolved in methanol to a concentration of 0.5 M after which 1.2 eq of methanolic $NaOCH_3$ was added. The mixture was stirred for 1 hour. Water (50 mL) was added, causing precipitation of the product. The mixture was filtered and the precipitate on the filter was washed with water (20 mL). The product was dried in vacuo. Additionally, precipitation from the aqueous filtrate was observed after 10 weeks. The filtrate was filtered again, collecting the precipitate on the filter paper which was dried in vacuo. The products were combined to obtain diphenylbiguanide **23** as a white solid (0.56 g, 44%). ^1H NMR (400 MHz, DMF-d_7) δ = 10.49 (s, 2H), 7.84 (s, 3H), 7.45 (d, J = 8.0 Hz, 4H), 7.33 (t, J = 7.7 Hz, 4H), 7.11 (t, J = 7.4 Hz, 2H); ^{13}C NMR (100 MHz, DMF-d_7) δ = 172.02, 156.70, 143.62, 129.54, 123.92 ppm. LC-MS: *m/z* calc. for $C_{14}H_{15}N_5^+$ 254.13 Da [M+H]$^+$; found 254.25 Da.

*6-(4-(Pent-4-en-1-yloxy)phenyl)-N^2,N^4-diphenyl-1,3,5-triazine-2,4-diamine (**23**)*

1,5-Diphenylbiguanide (**22**) (0.15 g, 0.59 mmol) and triethylamine (0.18 g, 1.78 mmol, 3 eq) were dissolved in dimethylacetamide (DMAc) and stirred under argon atmosphere. Benzoyl chloride **21** (0.24 g, 1.07 mmol, 1.8 eq) was dissolved in dry $CHCl_3$ and added dropwise to the reaction mixture. The mixture was stirred at 50 °C overnight. The mixture was allowed to cool to room temperature and transferred to a separation funnel. The mixture was washed with water (100 mL), saturated $NaHCO_3$ (100 mL) and saturated NaCl (100 mL). The organic layer was collected and dried using $MgSO_4$, followed by filtration and evaporation of the $CHCl_3$ in vacuo. DMAc had remained in the organic phase and was removed using vacuum distillation. The product was obtained via recrystallization from hot $CHCl_3$ and subsequently cooled in an ice bath. The recrystallization was repeated four times and product **24** was obtained as a white solid (83 mg, 33%). ^1H NMR (400 MHz, DMF-d_7) δ = 9.87 (s, 2H), 8.50 – 8.32 (m, 2H), 8.03 – 7.84 (m, 4H), 7.39 (t, J = 7.7 Hz, 4H), 7.24 – 7.00 (m, 4H), 5.94 – 5.76 (m, 1H), 5.23 – 4.93 (m, 2H), 4.15 (t, J = 6.4 Hz, 2H), 2.33 (q, J = 7.1 Hz, 2H), 1.96 (p, J = 6.8 Hz, 2H); ^{13}C NMR (100 MHz, DMF-d_7) δ = 170.71, 165.09, 140.48, 138.47, 130.27, 129.43, 128.84, 122.72, 120.62, 115.02 114.77, 67.64, 29.75, 28.60 ppm. LC-MS: *m/z* calc. for $C_{26}H_{25}N_5O^+$ 424.52 [M+H]$^+$; found 424.50 Da.

Triazine-Si₈-triazine (24)

Starting from olefin-terminated triazine **23** (24 mg, 0.057 mmol, 3 eq) and *o*DMS$_8$ (11 mg, 0.019 mmol), **triazine-Si$_8$-triazine** was obtained in 3 h using general method A. The crude product was purified by automated flash column chromatography using heptane/EtOAc (gradient 95/5 to 85/15) as eluent. The solvents were removed in vacuo. Additional purification using recycling GPC in $CHCl_3$ was necessary. The pure fractions were collected, and the product was dried in vacuo, yielding **triazine-Si$_8$-triazine** as a white solid (13 mg, 46%). ^{1}H NMR (400 MHz, $CDCl_3$) δ = 8.37 (d, J = 8.4 Hz, 4H), 7.66 (dd, J = 8.2, 4.3 Hz, 8H), 7.41 – 7.32 (m, 8H), 7.23 – 7.17 (m, 4H), 7.14 – 7.07 (m, 4H), 7.01 – 6.94 (m, 4H), 4.03 (t, J = 6.5, 4H), 1.87 – 1.78 (m, 4H), 1.57 – 1.37 (m, 8H), 0.63 – 0.58 (m, 4H), 0.16 – 0.03 (m, 48H) ppm. ^{13}C NMR (100 MHz, $CDCl_3$) δ = 171.11, 164.35, 162.21, 138.29, 130.04, 128.64, 128.36, 123.20, 120.34, 114.01, 67.92, 29.51, 28.75, 22.88, 18.00, 0.99, 0.89, 0.87, 0.85 ppm. MS (MALDI-TOF): *m/z* calc. for $C_{68}H_{100}N_{10}O_9Si_8^+$ 1425.58; found 1426.6 Da [M+H]$^+$.

Triazine-Si₄₀-triazine (25)

Starting from olefin-terminated triazine **23** (20 mg, 0.047 mmol, 2.5 eq) and *o*DMS$_{40}$ (55 mg, 0.019 mmol), **triazine-Si$_{40}$-triazine** was obtained in 2 h using general method A. The crude product was purified by automated flash column chromatography using heptane/EtOAc (75/25) as eluent. The solvents were removed in vacuo. Additional purification using recycling GPC in $CHCl_3$ was necessary. The pure fractions were collected, and the product was dried, yielding **triazine-Si$_{40}$-triazine** as a white solid (28 mg, 40%). ^{1}H NMR (400 MHz, $CDCl_3$) δ = 8.42 (s, 4H), 7.61 (s, 8H), 7.34 (s, 8H), 7.18 (s, 4H), 6.99 (s, 4H), 3.98 (s, 4H), 1.76 (s, 4H), 1.40 (s, 8H), 0.62 – 0.50 (m, 4H), 0.12 – 0.02 (m, 248H); ^{13}C NMR (100 MHz, $CDCl_3$) δ = 163.93, 162.41, 128.12, 124.45, 122.46, 122.03, 67.08, 28.67, 27.79, 22.09, 17.19, 8.44, 0.37, 0.13, -0.37, -0.71, -0.80, -1.04, -1.60. MS (MALDI-TOF): *m/z* calc. for $C_{132}H_{292}N_{10}O_{41}Si_{40}^+$ 3793.18; found 3794.39 Da [M+H]$^+$.

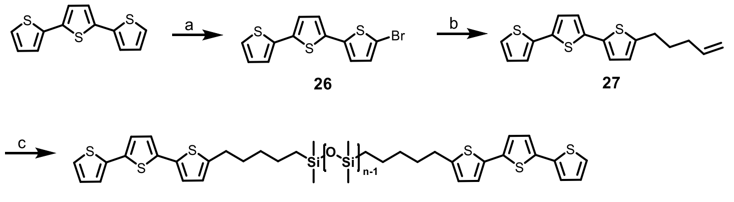

Thioph-Si$_n$-Thioph
n = 8, 16

***Scheme S7**: Synthesis of **Thioph-Si$_n$-Thioph**. Reaction conditions: (a) NBS, DMF, RT, O/N (7%); (b) 1) Mg, 1-bromo-pent-4-ene, Et_2O, reflux, 1 h; 2) Ni(dppp)Cl_2, RT, O/N (33%); (c) oDMS$_8$ or oDMS$_{16}$, DCM, Karstedt's catalyst, RT, 1–1.5 h (12–36%).*

*5-Bromo-2,2': 5',2''-terthiophene (**26**)*

Terthiophene (5 g, 20.1 mmol, 1 eq) and *N*-bromosuccinimide (NBS) (3.6 g, 20.1 mmol) were dissolved in DMF (50 mL) in an aluminum covered round-bottom flask to protect the mixture from light. The reaction was stirred overnight in the dark and at room temperature. The crude mixture was filtered and the filter paper was rinsed with DCM (150 mL). The filtrate was transferred to a separation funnel and washed with saturated $NaHCO_3$ (100 mL) and the organic layer was collected. The aqueous layer was extracted with EtOAc (3 × 100 mL). The organic layers were collected, combined and washed with brine (100 mL). The crude product was dried using $MgSO_4$, filtered and the filtrate was dried in vacuo. The crude product was impregnated on 30 g silica using DCM. The dry product was loaded onto a silica column and eluted with heptane. The mono-brominated product was collected and dried in vacuo, yielding pure bromo-terthiophene **11** as a green solid (0.54 g, 7%). ^{1}H NMR (400 MHz, $CDCl_3$) δ = 7.20 (dd, J = 5.1, 1.2 Hz, 1H), 7.16 (dd, J = 3.6, 1.2 Hz, 1H), 7.04 (d, J = 3.7 Hz, 1H), 7.02 – 6.98 (m, 2H), 6.96 (d, J = 3.9 Hz, 1H), 6.89 (d, J = 3.9 Hz, 1H) ppm.

*5-(Pent-4-en-1-yl)-2,2': 5',2''-terthiophene (**27**)*

A Schlenk tube was equipped with Mg turnings (58 mg, 2.38 mmol, 1.5 eq) which were dissolved in Et2O (3 mL) under argon atmosphere. The mixture was heated to reflux and 5-bromo-

1-pentene (**2**) (0.24 mL, 1.98 mmol, 1.3 eq) was added to the solution. In a sperate flask, mono-brominated terthiophene (**26)** (500 mg, 1.53 mmol, 1 eq) and Ni(dppp)Cl_2 (41 mg, 0.08mmol, 0.05 eq) were dissolved in Et2O (5 mL). Using a cannula transfer tube, the fresh Grignard reagent was added dropwise to the terthiophene solution while stirring. The reaction was stirred overnight. After full conversion of the terthiophene-bromide, the mixture was quenched with a 1M HCl solution (5 mL). The mixture was transferred to a separation funnel and the layers were allowed to separate. The organic layer was collected and washed with water (2 × 10 mL). The organic layer was dried using $MgSO_4$, filtered and concentrated in vacuo to a volume of ~1 mL. The crude product was purified by automated flash column chromatography using heptane/pentene (gradient 100/0 to 95/5) as eluent. The product was dried in vacuo, yielding the pure olefin-functionalized terthiophene **27** as a green solid (160 mg, 33%). ^{1}H NMR (400 MHz, $CDCl_3$) δ = 7.18 (dd, J = 5.1, 1.2 Hz, 1H), 7.14 (dd, J = 3.7, 1.2 Hz, 1H), 7.04 (d, J = 3.8 Hz, 1H), 7.00 – 6.94 (m, 3H), 6.67 (d, J = 5.2 Hz, 1H), 5.82 (ddt, J = 16.9, 10.2, 6.6 Hz, 1H), 5.09 – 4.96 (m, 2H), 2.80 (t, J = 6.8 Hz, 2H), 2.14 (q, J = 6.3 Hz, 2H), 1.78 (p, J = 7.5 Hz, 2H); ^{13}C NMR (100 MHz, $CDCl_3$) δ = 145.12, 138.16, 137.41, 136.88, 135.70, 134.77, 127.95, 125.15, 124.59, 124.41, 123.69, 123.64, 123.52, 115.28, 33.13, 30.79, 29.61 ppm.

Thioph-Si$_8$-Thioph (28)

Starting from olefin-terminated terthiophene **27** (40 mg, 0.13 mmol, 2.2 eq) and *o*DMS_8 (30 mg, 0.06 mmol), **Thioph-Si$_8$-Thioph** was obtained in 1.5 h using general method A. The crude product was purified by automated flash column chromatography using heptane/$CHCl_3$ (gradient 85/15 to 60/40) as eluent. The solvents were removed in vacuo. Additional purification using recycling GPC in $CHCl_3$ was necessary. The pure fractions were collected, and the product was dried in vacuo and obtained as a green solid (25 mg, 36%). ^{1}H NMR (400 MHz, $CDCl_3$) δ = 7.20 (dd, J = 5.1, 1.2 Hz, 2H), 7.16 (dd, J = 3.6, 1.2 Hz, 2H), 7.06 (d, J = 3.7 Hz, 2H), 7.01 (dd, J = 5.1, 3.6 Hz, 2H), 6.99 (dd, J = 4.6, 3.7 Hz, 4H), 6.68 (d, J = 3.4, 2H), 2.79 (d, J = 7.4 Hz, 4H), 1.69 (p, J = 7.2 Hz, 4H), 1.48 – 1.32 (m, 8H), 0.60 – 0.51 (m, 4H), 0.14 – 0.03 (m, 48H); ^{13}C NMR (100 MHz, $CDCl_3$) δ = 145.74, 137.46, 137.01, 135.65, 134.62, 127.96, 124.94, 124.61, 124.40, 123.71, 123.63, 123.52, 32.97, 31.47, 30.30, 23.13, 18.33, 1.73, 1.62, 1.36, 1.26, 1.25, 0.88, 0.35, 0.15, 0.06 ppm. MS (MALDI-TOF): *m/z* calc. for $C_{50}H_{82}O_7S_6Si_8^+$ 1210.25; found 1210.27 Da [M+H]$^+$.

Thioph-Si$_{16}$-Thioph (29)

Starting from olefin-terminated terthiophene **28** (100 mg, 0.32 mmol, 2.2 eq) and *o*DMS_{16} (170 mg, 0.14 mmol), **Thioph-Si$_{16}$-Thioph$_3$** was obtained in 1 h using general method A. The crude product was purified by automated flash column chromatography using heptane/$CHCl_3$ (gradient 85/15 to 50/50) as eluent. The solvents were removed in vacuo. Additional purification using recycling GPC in $CHCl_3$ was necessary. The pure fractions were collected, and the product was dried and obtained as a green solid (30 mg, 12%). ^{1}H NMR (400 MHz, $CDCl_3$) δ = 7.23 (dd, J = 5.1, 1.2 Hz, 2H), 7.18 (dd, J = 5.1, 1.2 Hz, 2H), 7.08 (d, J = 3.7 Hz, 2H), 7.04 (dd, J = 5.1, 3.6 Hz, 2H), 7.01 (dd, J = 4.9, 3.7 Hz, 4H), 6.71 (d, J = 4.6 Hz, 4H), 2.82 (t, J = 7.8 Hz, 4H), 1.72 (p, J = 7.4 Hz, 4H), 1.49 – 1.35 (m, 8H), 0.63 – 0.51 (m, 4H), 0.09 – 0.01 (m, J = 10.6 Hz, 96H); ^{13}C NMR (100 MHz, $CDCl_3$) δ = 145.74, 137.46, 136.99, 135.64, 134.61, 127.97, 124.94, 124.40, 123.63, 123.51, 32.97, 31.48, 30.29, 23.13, 18.32, 14.27, 1.58, 1.35, 1.24, 1.21, 0.84, 0.34, 0.15 ppm. MS (MALDI-TOF): *m/z* calc. for $C_{66}H_{130}O_{15}S_6Si_{16}^+$ 1802.40; found 1802.42 Da [M+H]$^+$.

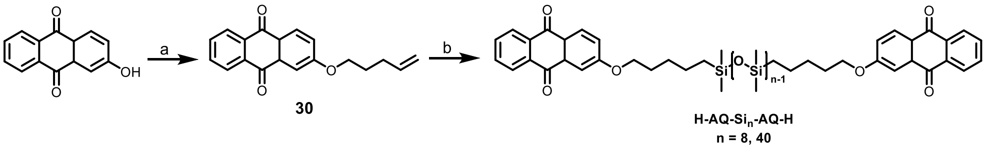

Scheme 8: *Synthesis of **H-AQ-Si$_n$-AQ-H**. Reaction conditions: (a) 1-bromo-pent-4-ene, K_2CO_3, KI, dry DMF, 80 °C, O/N (75%); (b) oDMS$_8$ or oDMS$_{40}$, DCM, Karstedt's catalyst, RT, 2 h (55–81%).*

*2-(Pent-4-en-1-yloxy)anthraquinone (**30**)*

2-Hydroxyanthraquinone (0.58 g, 2.57 mmol), K_2CO_3 (0.71 g, 5.13 mmol, 2 eq) and KI (43 mg, 0.26 mmol, 0.1 eq) were dissolved in acetone. The mixture was stirred and 5-bromo-1-pentene (**2**) (0.77 g, 5.13 mmol, 2 eq) was added. After full conversion, the mixture was precipitated in water and filtered. The residue was dried in vacuo, yielding the olefin-functionalized anthraquinone **30** as a dark-green solid (0.57 g, 75%). ^{1}H NMR (400 MHz, $CDCl_3$) δ = 8.36 – 8.19 (m, 3H, Ant-H), 7.84 – 7.65 (m, 3H, Ant-H), 7.31 – 7.18 (m, 1H, Ant-H), 5.88 (m, 1H, CH=CH_2), 5.06 (m, 2H, CH=CH_2), 4.17 (t, J = 6.4 Hz, 2H, O-CH_2), 2.28 (q, J = 7.1 Hz, 2H, CH_2-CH_2-CH), 1.97 (p, J = 6.8 Hz, 2H, CH_2-CH_2-CH); ^{13}C NMR (100 MHz, $CDCl_3$) δ = 183.35, 182.18, 163.94, 137.48, 135.64, 134.21, 133.77, 133.71, 133.67, 129.82, 127.21, 127.05, 121.51, 115.70, 110.62, 68.05, 30.08, 28.28 ppm.

H-AQ-Si$_8$-AQ-H (31)

Starting from olefin-terminated anthraquinone **30** (0.22 g, 0.75 mmol, 2.5 eq) and *o*DMS$_8$ (0.174 g, 0.30 mmol), **H-AQ-Si$_8$-AQ-H** was obtained in 2 h using general method A. The crude product was purified using automated column chromatography using heptane/EtOAc (95/5) as eluent. The product was dried in vacuo and obtained as a yellow wax (0.28 g, 81%). ^{1}H NMR (400 MHz, $CDCl_3$) δ = 8.36 – 8.19 (m, 6H, Ant-H), 7.84 – 7.65 (m, 6H, Ant-H), 7.31 – 7.18 (m, 2H, Ant-H), 4.13 (t, J = 6.5 Hz, 4H, O-CH_2), 1.85 (p, J = 6.8 Hz, 4H, O-CH_2-CH_2-), 1.56 – 1.52 (m, 4H, O-CH_2-CH_2-CH_2-CH_2), 1.52 – 1.43 (m, 4H, O-CH_2-CH_2-CH_2-CH_2), 0.65 – 0.54 (m, 4H, CH_2-Si(CH_3)$_2$), 0.11 – 0.04 (m, 48H, Si(CH_3)$_2$); ^{13}C NMR (100 MHz, $CDCl_3$) δ = 183.42, 182.25, 164.11, 135.69, 134.23, 133.84, 133.72, 129.84, 127.24, 127.02, 121.57, 110.64, 68.98, 29.79, 28.94, 23.21, 18.34, 1.35, 1.24 ppm. MS (MALDI-TOF): *m/z* calc. for $C_{54}H_{86}O_{13}Si_8^+$: 1166.42 [M+H]$^+$; found: 1186.17 Da.

H-AQ-Si$_{40}$-AQ-H (32)

Starting from olefin-terminated anthraquinone **30** (62 mg, 0.21 mmol, 2.5 eq) and *o*DMS$_{40}$ (0.25 g, 0.085 mmol), **H-AQ-Si$_{40}$-AQ-H** was obtained in 2 h using general method A. The crude product was purified using automated column chromatography using heptane/EtOAc (95/5) as eluent. The product was dried in vacuo and obtained as a yellow oil (0.17 g, 55%). ^{1}H NMR (400 MHz, $CDCl_3$) δ = 8.36 – 8.19 (m, 6H, Ant-H), 7.84 – 7.65 (m, 6H, Ant-H), 7.31 – 7.18 (m, 2H, Ant-H), 4.15 (t, J = 6.5 Hz, 4H, O-CH_2), 1.93 – 1.77 (m, 4H, O-CH_2-CH_2-), 1.52 (t, J = 7.9 Hz, 4H, O-CH_2-CH_2-CH_2-CH_2), 1.47 – 1.39 (m, 4H, , O-CH_2-CH_2-CH_2-CH_2), 0.73 – 0.51 (m, 4H, CH_2-Si(CH_3)$_2$), 0.11 – 0.04 (m, 240H, Si(CH_3)$_2$); ^{13}C NMR (100 MHz, $CDCl_3$) δ = 183.48, 182.31, 164.16, 135.74, 134.26, 133.89, 133.75, 129.88, 127.28, 127.07, 121.62, 110.67, 69.00, 29.80, 28.96, 23.22, 18.36, 1.34, 1.24, 1.19 ppm. MS (MALDI-TOF): *m/z* calc. for $C_{118}H_{278}O_{45}Si_{40}Na^+$: 3557.05 [M+Na]$^+$; found: 3559.08 Da.

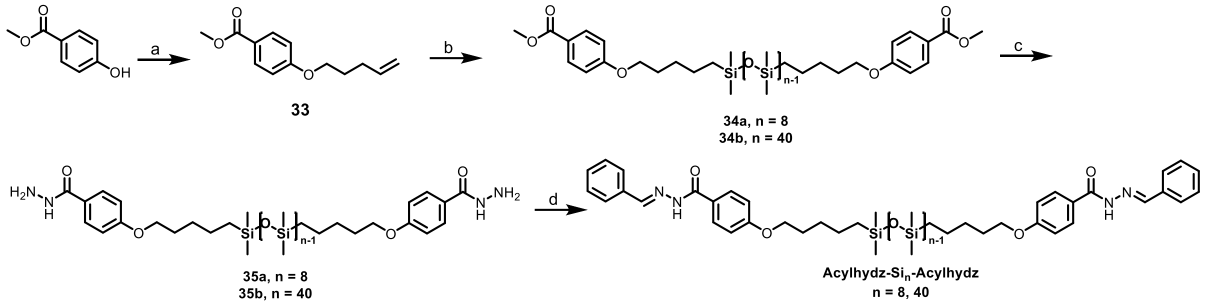

Scheme S9: *Synthesis of **Acylhydz-Si$_n$-Acylhydz**. Reaction conditions: (a) 1-bromo-pent-4-ene, K_2CO_3, acetone, 65 °C, O/N (92%); (b) oDMS$_8$ or oDMS$_{40}$, DCM, Karstedt's catalyst, RT, 1–1.5 h (52–83%); (c) $N_2H_4{\cdot}H_2O$, THF, 70 °C, O/N – 3 days (75%); (d) benzaldehyde, $MgSO_4$, DCM, RT, O/N (66–86%).*

*Methyl 4-(pent-4-en-1-yloxy)benzoate (**33**)*

Methyl-4-hydroxybenzoate (3 g, 19.7 mmol) and K_2CO_3 (4.08 g, 29.6 mmol, 1.5 eq) were dissolved in acetone (40 mL). The mixture was stirred and 5-bromo-1-pentene (2.6 mL, 21.7 mmol, 1.1 eq) was added. The mixture was heated to 65 °C and stirred overnight. The crude mixture was cooled to room temperature and poured into water (50 mL). The mixture was transferred to a separation funnel and the product was extracted with diethylether (3 × 40 mL). The organic layers were collected, combined and washed with brine (50 mL). The organic layer was collected and dried using $MgSO_4$. The solution was filtered, and the crude product was concentrated in vacuo to a volume of ~10 mL. The crude product was purified by automated flash column chromatography using heptane/EtOAc (gradient 95/5 to 40/60) as eluent. The product was dried in vacuo, yielding the pure olefin-functionalized methylbenzoate **33** (3.98 g, 92%). ^1H NMR (400 MHz, $CDCl_3$) δ = 7.98 (d, J = 8.8 Hz, 2H), 6.90 (d, J = 8.8 Hz, 2H), 5.85 (ddt, J = 17.0, 10.2, 6.7 Hz, 1H), 5.11 – 4.93 (m, 2H), 4.02 (t, J = 6.4 Hz, 2H), 3.88 (s, 3H), 2.23 (d, J = 6.9 Hz, 2H), 1.91 (p, J = 6.9 Hz, 2H); ^{13}C NMR (100 MHz, $CDCl_3$) δ = 167.04, 162.99, 137.71, 131.71, 122.56, 115.53, 114.21, 67.47, 51.98, 30.16 ppm.

*Methylbenzoate-Si$_8$-methylbenzoate (**34a**)*

Starting from olefin-terminated methyl benzoate **33** (0.80 g, 2.1 mmol, 2.2 eq) and *o*DMS$_8$ (0.55 g, 0.95 mmol), methylbenzoate-Si$_8$-methylbenzoate (**34a**) was obtained in 1.5 h using general method A. The crude product was purified by automated flash column chromatography using heptane/DCM (gradient 40/60 to 0/100) as eluent. The solvents were removed in vacuo, yielding pure product **17a** as an oil (0.80 g, 83%). ^1H NMR (400 MHz, $CDCl_3$) δ = 7.89 (d, J = 8.6 Hz, 4H), 6.89 (d, J = 8.6 Hz, 4H), 3.99 (t, J = 6.6 Hz, 4H), 3.88 (s, 6H), 1.80 (p, J = 6.9 Hz, 4H), 1.59 – 1.31 (m, 8H), 0.64 – 0.47 (m, 4H), 0.09 (s, 48H); ^{13}C NMR (100 MHz, $CDCl_3$) δ = 167.04, 163.09, 131.69, 122.45, 114.18, 68.30, 51.94, 29.81, 29.01, 23.20, 18.33, 1.60, 1.33, 1.23 ppm. MS (MALDI-TOF): *m/z* calc. for $C_{42}H_{82}O_{13}Si_8Na^+$ 1041.39; found 1041.39 Da $[M+Na]^+$.

*Methylbenzoate-Si$_8$-methylbenzoate (**34b**)*

Starting from olefin-terminated methyl benzoate **33** (0.25 g, 1.1 mmol, 2.2 eq) and *o*DMS$_{40}$ (1.5 g, 0.95 mmol), methylbenzoate-Si$_{40}$-methylbenzoate (**34b**) was obtained in 1 h using general method A. The crude product was purified by automated flash column chromatography using heptane/DCM (gradient 80/20 to 0/100) as eluent. The solvents were removed in vacuo, yielding pure product **17b** as an oil (0.89 g, 52%). ^1H NMR (400 MHz, $CDCl_3$) δ = 7.98 (d, J = 8.8 Hz, 4H), 6.90 (d, J = 8.7 Hz, 4H), 4.00 (t, J = 6.5 Hz, 4H), 3.88 (s, 6H), 1.80 (p, J = 6.8 Hz, 4H), 1.56 – 1.32 (m, 8H), 0.65 – 0.51 (m, 4H), 0.08 (s, 240H); ^{13}C NMR (100 MHz, $CDCl_3$) δ = 167.06, 163.12, 131.71, 122.49, 114.21, 68.33, 51.95, 29.84, 29.04, 23.22, 18.36, 1.57, 1.34, 1.24, 1.20 ppm. MS (MALDI-TOF): *m/z* calc. for $C_{106}H_{274}O_{46}Si_{40}Na^+$ 3409.99; found 3410.93 Da $[M+Na]^+$.

*Benzohydrazide-Si$_8$-benzohydrazide (**35a**)*

Hydrazine monohydrate (0.48 mL, 9.8 mmol, 20 eq) was added to a solution of methylbenzoate-Si$_8$-methylbenzoate (**34a**) (0.5 g, 0.49 mmol) in THF (5mL). The mixture was heated to reflux and stirred overnight. The crude product was concentrated in vacuo to a volume

of ~2 mL, and purified by automated flash column chromatography using DCM/MeOH (gradient 100/0 to 90/100) as eluent. The product was dried in vacuo, yielding the pure benzohydrazide-Si_8-benzohydrazide (**35a**) as a yellow oil (0.38 g, 75%). ^{1}H NMR (400 MHz, $CDCl_3$) δ = 7.62 (d, J = 8.7 Hz, 4H), 7.34 (s, 2H), 6.83 (d, J = 8.8 Hz, 5H), 4.02 (s, 4H), 3.91 (t, J = 6.5 Hz, 4H), 1.72 (p, J = 6.8 Hz, 4H), 1.45 – 1.21 (m, 8H), 0.57 – 0.40 (m, 4H), 0.06 – 0.01 (m, J = 11.5 Hz, 48H); ^{13}C NMR (100 MHz, $CDCl_3$) δ = 168.54, 162.25, 128.74, 124.72, 114.55, 68.33, 29.82, 29.02, 23.20, 18.33, 1.61, 1.34, 1.24 ppm. MS (MALDI-TOF): *m/z* calc. for $C_{40}H_{82}N_4O_{11}Si_{40}Na^+$ 1041.41; found 1041.39 Da $[M+Na]^+$.

*Benzohydrazide-Si_{40}-benzohydrazide (**35b**)*

Hydrazine monohydrate (0.48 mL, 9.8 mmol, 20 eq) was added to a solution of methylbenzoate-Si_{40}-mehtlbenzoate (**34b**) (0.5 g, 0.49 mmol) in THF (5mL). The mixture was heated to reflux and stirred for 3 days. The product was dried in vacuo, yielding benzohydrazide-Si_8-benzohydrazide (**35b**) as a yellow oil (product was used without further purification). ^{1}H NMR (400 MHz, $CDCl_3$) δ = 7.62 (d, J = 8.4 Hz, 4H), 7.26 (s, 2H), 6.84 (d, J = 8.5 Hz, 4H), 3.91 (t, J = 6.5 Hz, 4H), 1.73 (p, J = 6.9 Hz, 4H), 1.50 – 1.26 (m, 8H), 0.58 – 0.44 (m, 4H), 0.08 – 0.00 (m, 240H); ^{13}C NMR (100 MHz, $CDCl_3$) δ = 168.55, 162.27, 128.73, 124.73, 114.57, 68.34, 29.82, 29.02, 26.20, 23.21, 18.34, 1.56, 1.33, 1.22, 1.19 ppm. MS (MALDI-TOF): *m/z* calc. for $C_{104}H_{274}N_4O_{43}Si_{40}Na^+$ 3410.01; found 3410.11 Da $[M+Na]^+$.

Acylhydz-Si_8-Acylhydz (36)

Benzohydrazide-Si_8-benzohydrazide (**35a**) (0.36 g, 0.35 mmol) and benzaldehyde (0.08 mL, 0.78 mmol, 2.2 eq) were dissolved in DCM (5 mL). $MgSO_4$ (0.85g, 7.1 mmol, 20 eq) was added and the suspension was stirred overnight. After full conversion of the hydrazide, the mixture was filtered to remove the $MgSO_4$. The filtrate was concentrated in vacuo to a volume of ~2 mL, and purified by automated flash column chromatography using DCM/EtOAc (gradient 100/0 to 0/100) as eluent. The product was dried in vacuo and obtained as a white solid (0.37 g, 86%). ^{1}H NMR (400 MHz, $CDCl_3$) δ = 10.29 (s, 2H), 8.34 (s, 2H), 7.84 (s, 4H), 7.57 (d, J = 7.0 Hz, 4H), 7.22 (s, 5H), 6.79 (s, 4H), 3.85 (s, 4H), 1.70 (d, J = 6.1 Hz, 4H), 1.45 – 1.26 (m, 8H), 0.54 – 0.46 (m, 4H), 0.10 – 0.01 (m, 48H); ^{13}C NMR (100 MHz, $CDCl_3$) δ = 171.31, 162.30, 148.54, 133.99, 130.30, 129.56, 128.71, 127.73, 125.14, 114.38, 68.29, 29.82, 29.04, 23.22, 18.33, 1.71, 1.61, 1.35, 1.24 ppm. MS (MALDI-TOF): *m/z* calc. for $C_{54}H_{90}N_4O_{11}Si_8Na^+$ 1217.48; found 1217.49 Da $[M+Na]^+$.

Acylhydz-Si_{40}-Acylhydz (37)

Benzohydrazide-Si_{40}-benzohydrazide (**35a**) (0.79 g, 0.23 mmol) and benzaldehyde (0.09 mL, 0.92 mmol, 4 eq) were dissolved in DCM (5 mL). $MgSO_4$ (0.55 g, 4.6 mmol, 20 eq) was added and the suspension was stirred overnight. After full conversion of the hydrazide, the mixture was filtered to remove the $MgSO_4$. The filtrate was concentrated in vacuo to a volume of ~2 mL, and purified by automated flash column chromatography using DCM/EtOAc (gradient 100/0 to 0/100) as eluent. The product was dried in vacuo and obtained as a white solid (0.54 g, 66%). ^{1}H NMR (400 MHz, $CDCl_3$) δ = 9.13 (s, 2H), 8.29 (s, 2H), 7.86 (s, 4H), 7.74 (s, 4H), 7.40 (s, 5H), 6.95 (d, J = 8.6 Hz, 4H), 4.01 (t, J = 6.5 Hz, 4H), 1.81 (p, J = 6.7 Hz, 4H), 1.52 – 1.39 (m, 8H), 0.64 – 0.50 (m, 4H), 0.13 – -0.02 (m, 240H); ^{13}C NMR (100 MHz, $CDCl_3$) δ = 171.28, 162.23, 148.44, 133.83, 130.21, 129.46, 128.86, 127.74, 125.16, 114.36, 68.38, 29.84, 29.05, 23.23, 18.35, 1.56, 1.34, 1.23, 1.19 ppm. MS (MALDI-TOF): *m/z* calc. for $C_{118}H_{282}N_4O_{43}Si_{40}Na^+$ 3586.08; found 3586.05 Da $[M+Na]^+$.

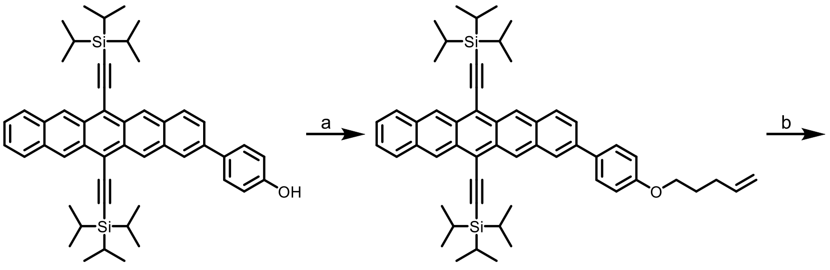

38

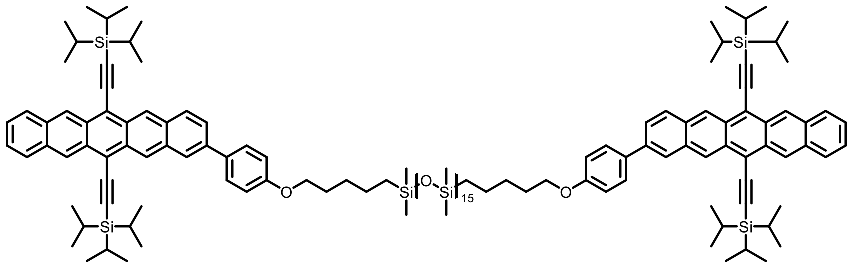

TIPSPent-Si$_{16}$-TIPSPent

Scheme S10: *Synthesis of* ***TIPSPent-Si$_{16}$-TIPSPent****. (a) 1-bromo-pent-4-ene, K_2CO_3, dry DMF, 80 °C, 48 h, 63 %. (b) Karstedt's catalyst, dry DCM, RT, 3h, 23 %.*

Pentacene-pentene **(38)**

Phenol-decorated TIPS-pentacene (308 mg, 0.42 mmol, 1 eq.), K_2CO_3 (127 mg, 0.92 mmol, 2.18 eq.) and 5-bromopent-1-ene (0.5 mL, 4.09 mmol, 9,7 eq.) were dissolved in 10 mL dry DMF and heated to 80 °C. After 48 hours, the mixture was cooled to room temperature before adding DI water. The target compound was extracted with EtOAc until the aqueous phase was colorless. The organic phase was dried with $MgSO_4$ and reduced in vacuo to 370 mg crude product as dark green solid that was impregnated on celite and purified by column chromatography (Solid loading, eluent heptane/EtOAc 100/0 – 95/5). The target product was obtained as dark green solid (210 mg, 63% yield).

^{1}H NMR (400 MHz, $CDCl_3$): δ 9.30 (s, 4H), 8.07 (s, 1H), 8.03 (d, *J* = 9.0 Hz, 1H), 8.00 – 7.95 (m, 2H), 7.76 – 7.67 (m, 3H), 7.43- 7.39 (m, 2H), 7.07 (d, *J* = 8.7 Hz, 2H), 5.96 – 5.83 (m, 1H), 5.15 – 5.00 (m, 2H), 4.05 (t, *J* = 6.4 Hz, 2H), 2.33 – 2.26 (m, 2H), 2.00 – 1.91 (m, 2H), 1.45 – 1.30 (m, 42H) ppm. ^{13}C NMR (100 MHz, $CDCl_3$): δ 159.14, 138.04, 137.94, 133.29, 132.72, 132.44, 132.38, 131.50, 131.12, 130.88, 130.81, 130.70, 129.37, 128.82, 128.43, 126.59, 126.46, 126.44, 126.31, 126.20, 126.15, 126.13, 124.89, 118.56, 118.27, 115.43, 115.18, 107.29, 107.21, 104.91, 104.85, 67.48, 30.30, 28.61, 19.18, 19.17, 11.85 ppm. MS (MALDI-TOF): *m/z* calc. for $C_{55}H_{66}OSi_2$ 799.30 Da; found 798.51 Da [M^+].

Pent-Si$_{16}$-Pent (39)

Starting from olefin-terminated pentacene **38** (53 mg, 0.007 mmol, 2.36 eq) and *o*DMS$_{16}$ (33 mg, 0.03 mmol), **Pent-Si$_{16}$-Pent** was obtained in 3 h using general method A. The crude product was purified using automated column chromatography using heptane/DCM (80/20) as eluent. The product was dried in vacuo and obtained as a tough dark green wax (18 mg, 23%).

^{1}H NMR (400 MHz, $CDCl_3$): δ 9.30 (s, 8H), 8.08 (s, 2H), 8.03 (d, *J* = 9.0 Hz, 2H), 8.00 – 7.95 (m, 4H), 7.76 – 7.67 (m, 6H), 7.44 – 7.40 (m, 4H), 7.07 (d, *J* = 8.4 Hz, 4H), 4.04 (t, *J* = 6.5 Hz, 4H), 1.89 – 1.80 (m, 4H), 1.59 – 1.49 (m, 4H), 1.48 – 1.40 (m, 4H), 1.39 (s, 48H), 1.37 (s, 24H), 0.66 – 0.58 (m, 4H), 0.11 – 0.04 (m, 96H) ppm. ^{13}C NMR (100 MHz, $CDCl_3$) δ 159.27, 138.10, 133.16, 132.74, 132.44, 132.37, 131.51, 131.12, 130.81, 130.70, 129.35, 128.82, 128.41, 126.62, 126.45, 126.30, 126.19, 126.15, 126.12, 124.85, 118.56, 118.26, 115.14, 107.28, 107.20, 104.85, 77.48, 77.16, 76.84, 68.33,

32.04, 29.84, 29.28, 23.06, 22.85, 19.17, 17.63, 14.28, 11.85, 1.94, 1.30, 1.30, 1.26, 1.24, 1.23, 0.16, -0.29 ppm. MS (MALDI-TOF): *m/z* calc. for $C_{141}H_{230}O_{17}Si_{20}$ 2771.09 Da, found 2770.22 Da [M^+].

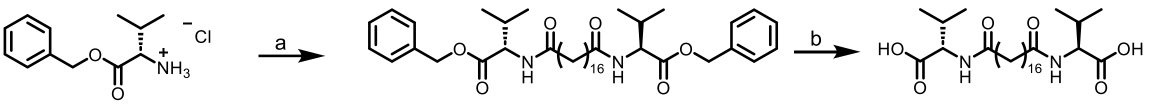

BnO-Val-C18-Val-OBn **Val-C18-Val**

Scheme 11: *Synthesis of* ***Val-C18-Val****. (a) DIPEA, DCM, Octadecanoic acid, HATU, RT, O/N (79%). (b) Pd/C, H2, MeOH, O/N (quant.).*

BnO-Val-C$_{18}$-Val-OBn (40)

BnO-*L*-Valine (298.8 mg, 1.23 mmol, 3 eq) and DIPEA (0.21 mL, 1.20 mmol, 3 eq) were dissolved in dry DCM (10 mL). Octadecanedioic acid (128.5 mg, 0.41 mmol), HATU (466.1 mg, 1.23 mmol) and DIPEA (0.14 mL, 0.80 mmol, 2 eq) were dissolved in dry DCM (5 mL) and to this, the mixture of BnO-*L*-Valine and DIPEA was added dropwise. The mixture was left to stir overnight at room temperature. The mixture was diluted with DCM (35 mL) and extracted with H_2O (3×50 mL). The organic phase was dried using Na_2SO_4, filtered and concentrated in vacuo. The crude material was purified by automated flash column chromatography using $CHCl_3/Et_2O$ (80/20 vv) as eluent. The product was dried in vacuo and obtained as a white solid (224.6 mg, 79%).
^{1}H NMR (400 MHz, $CDCl_3$): δ 7.41 – 7.30 (m, 10H), 5.89 (d, J = 8.8 Hz, 2H), 5.17 (q, J = 12.2 Hz, 4H), 4.64 (dd, J = 8.9, 4.7 Hz, 2H), 2.30 – 2.10 (m, 6H), 1.63 (p, J = 7.6 Hz, 4H), 1.26 (d, J = 14.6 Hz, 24H), 0.91 (d, J = 6.9 Hz, 6H), 0.86 (d, J = 6.9 Hz, 6H). ^{13}C NMR (100 MHz, $CDCl_3$) δ 173.05, 172.15, 135.33, 128.62, 128.47, 128.37, 67.04, 56.77, 36.80, 31.39, 29.67, 29.64, 29.61, 29.49, 29.34, 29.27, 25.72, 18.98, 17.68. MS (MALDI-TOF): *m/z* calc. for $C_{42}H_{64}N_2O_6$ 692.49 Da, Found: 693.49 Da $[M+H]^+$, 715.49 Da $[M+Na]^+$, 735.45 Da $[M+K]^+$

Val-C$_{18}$-Val (41)

BnO-Val-C$_{18}$-Val-OBn (**40**) was dissolved in dry MeOH (5 mL) and bubbled through with N_2 for 15 min. To the solution, Pd/C (6.7 mg) was added and a balloon filled with H_2 was connected through a septum. The mixture was saturated with H_2 and left to stir overnight at room temperature. Subsequently, the mixture was bubbled through with N_2 and filtered over celite. The filtrate was concentrated in vacuo to yield the material as a white solid (66.6 mg, quant.).
^{1}H NMR (400 MHz, Methanol-*d$_4$*): δ 4.31 (d, J = 5.7 Hz, 2H), 2.31 – 2.08 (m, 6H), 1.61 (p, J = 7.2 Hz, 4H), 1.30 (d, J = 17.0 Hz, 24H), 0.96 (m, J = 6.9, 3.9 Hz, 12H). ^{13}C NMR (100 MHz, $CDCl_3$) 50.29, 50.07, 49.86, 49.64, 49.43, 36.71, 29.55, 25.64, 18.90. MS (MALDI-TOF): *m/z* calc. for $C_{28}H_{52}N_2O_6$ 512.38 Da, Found: 513.38 Da $[M+H]^+$, 535.36 Da $[M+Na]^+$, 551.33 Da $[M+K]^+$, 557.34 Da $[M-H+2Na]^+$, 573.31 Da $[M-H+Na+K]^+$

3. SAXS and DSC characterization

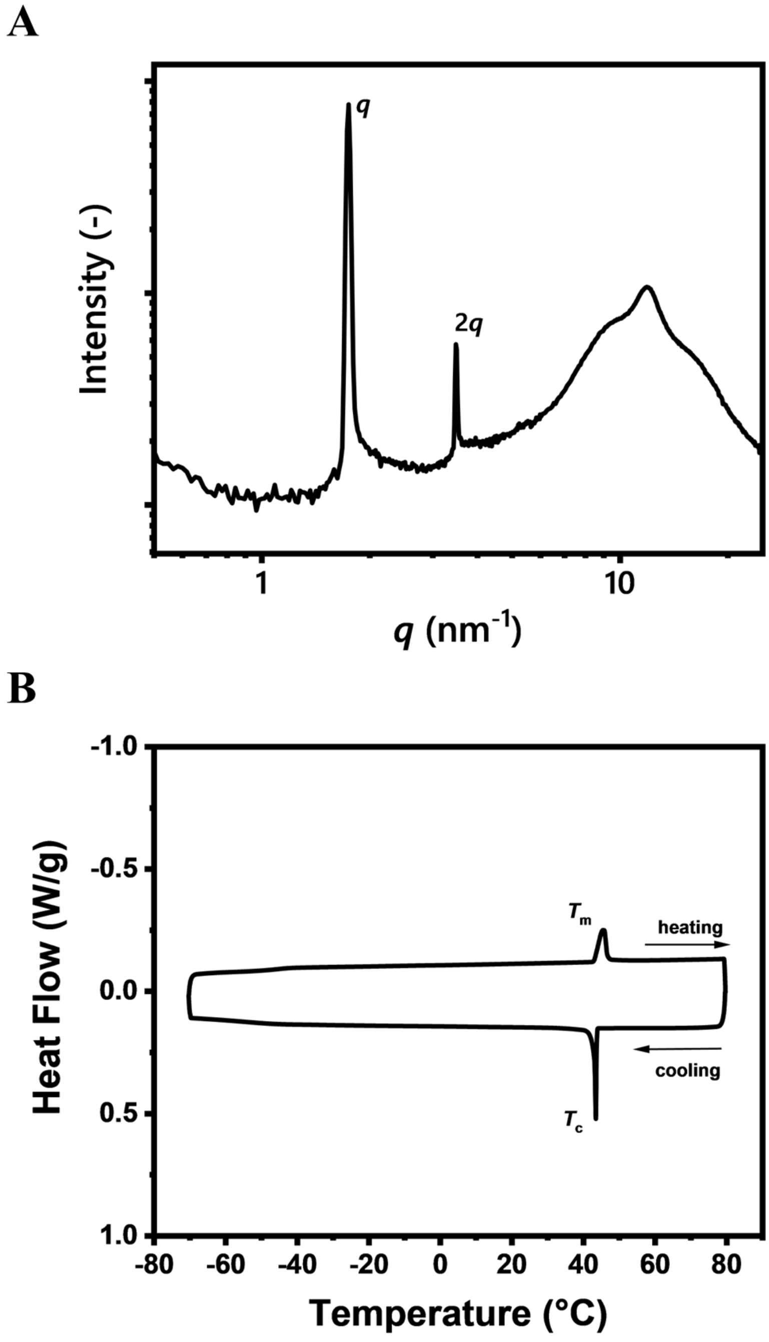

***Figure S2**: SAXS and DSC characterization of **Chol-Si$_8$-Chol**. (A) 1D transmission scattering profile at room temperature showing lamellar order (d_{lam} = 3.6 nm). (B) Differential scanning calorimetry trace of the second cycle with 10 K min^{-1} showing the melting and crystallization peaks (exothermic down).*

A

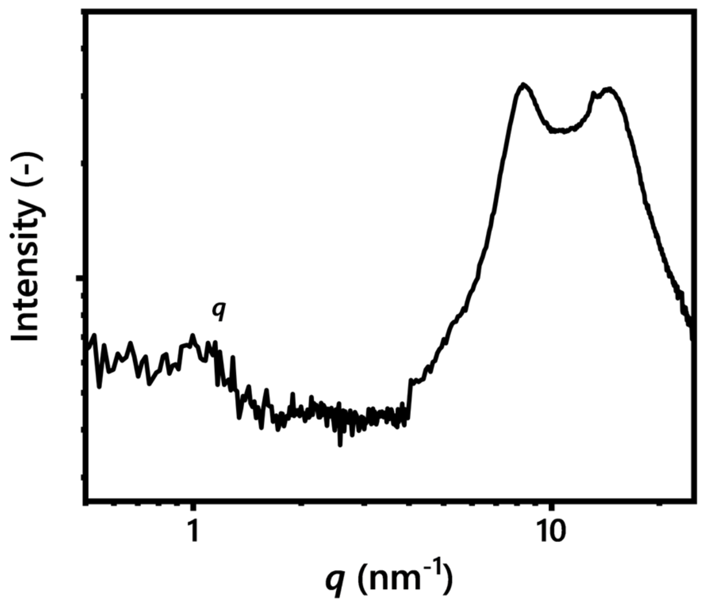

B

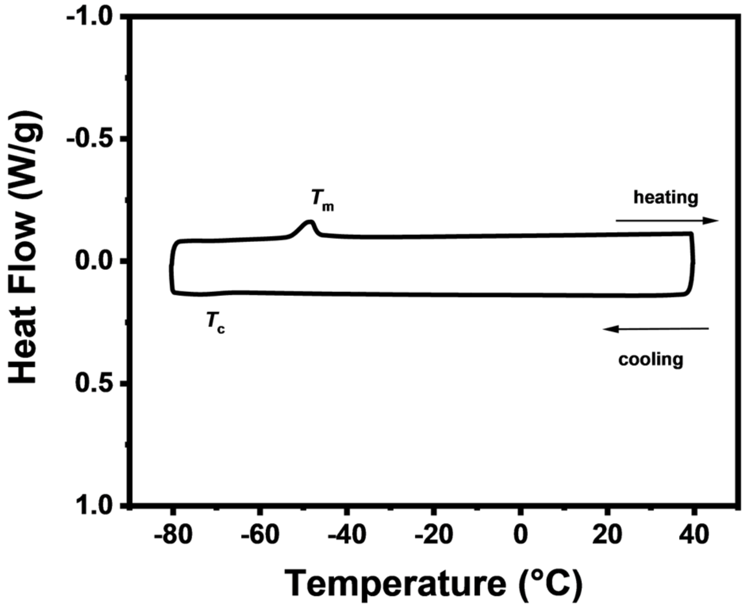

Figure S3*: SAXS and DSC characterization of* ***Chol-Si$_{40}$-Chol****. (A) 1D transmission scattering profile at −70 °C showing a disordered morphology. (B) Differential scanning calorimetry trace of the second cycle with 10 K min^{-1} showing the melting and crystallization peaks (exothermic down).*

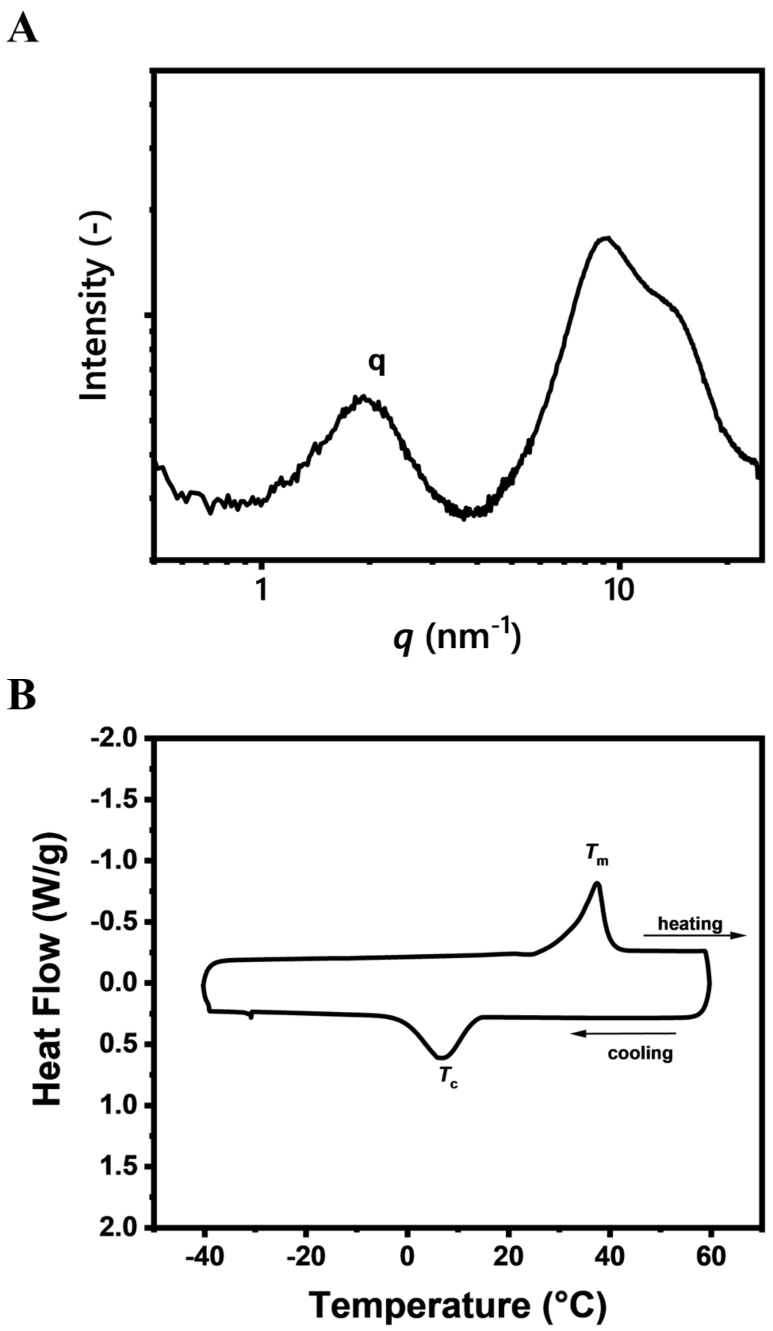

Figure S4: *SAXS and DSC characterization of* ***Est-Si$_8$-Est****. (A) 1D transmission scattering profile at −20 °C showing a disordered morphology. (B) Differential scanning calorimetry trace of the second cycle with 10 K min^{-1} showing the melting and crystallization peaks (exothermic down).*

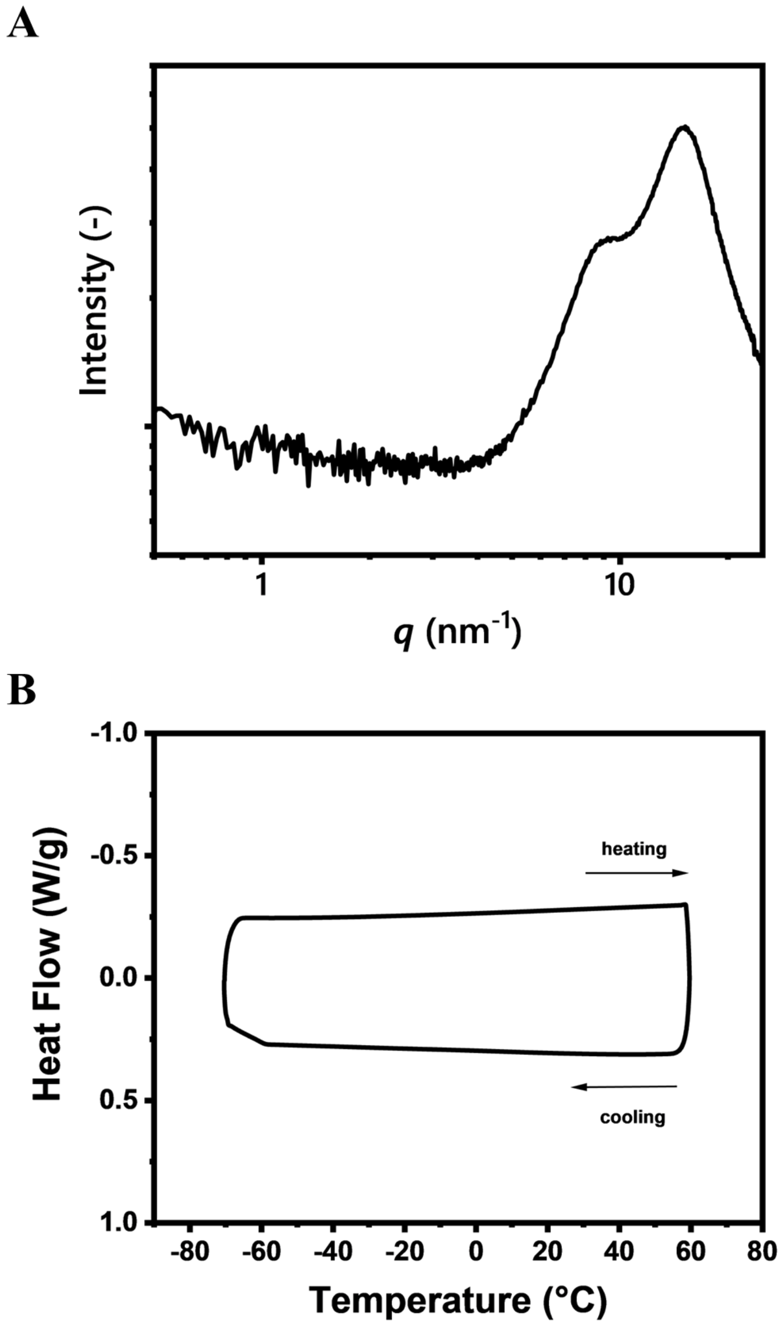

Figure S5: *SAXS and DSC characterization of* ***BnO-Val-Si$_8$-Val-OBn****. (A) 1D transmission scattering profile at room temperature showing a disordered morphology. (B) Differential scanning calorimetry trace of the second cycle with 10 K min^{-1} showing the melting and crystallization peaks (exothermic down).*

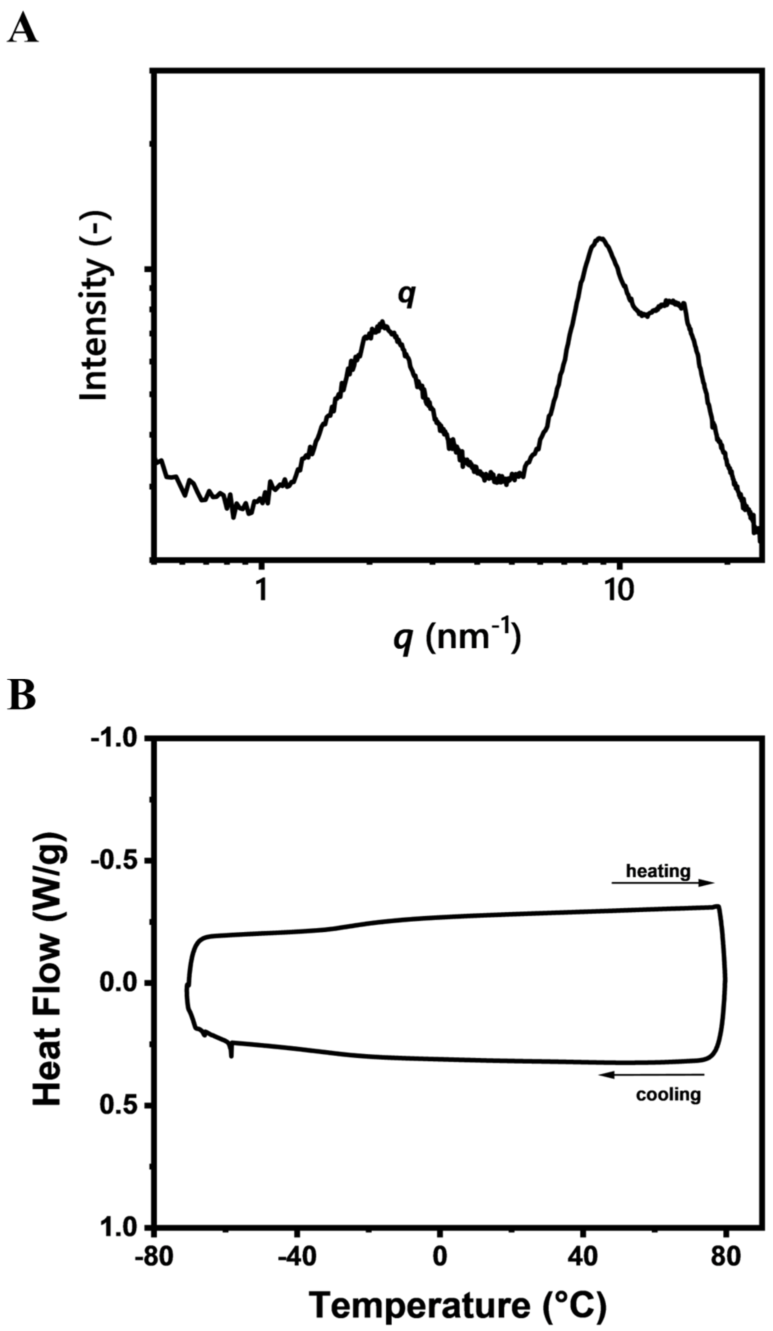

Figure S6: *SAXS and DSC characterization of* ***Val-Si$_8$-Val****. (A) 1D transmission scattering profile at −65 °C showing a disordered morphology. (B) Differential scanning calorimetry trace of the second cycle with 10 K min^{-1} showing the melting and crystallization peaks (exothermic down).*

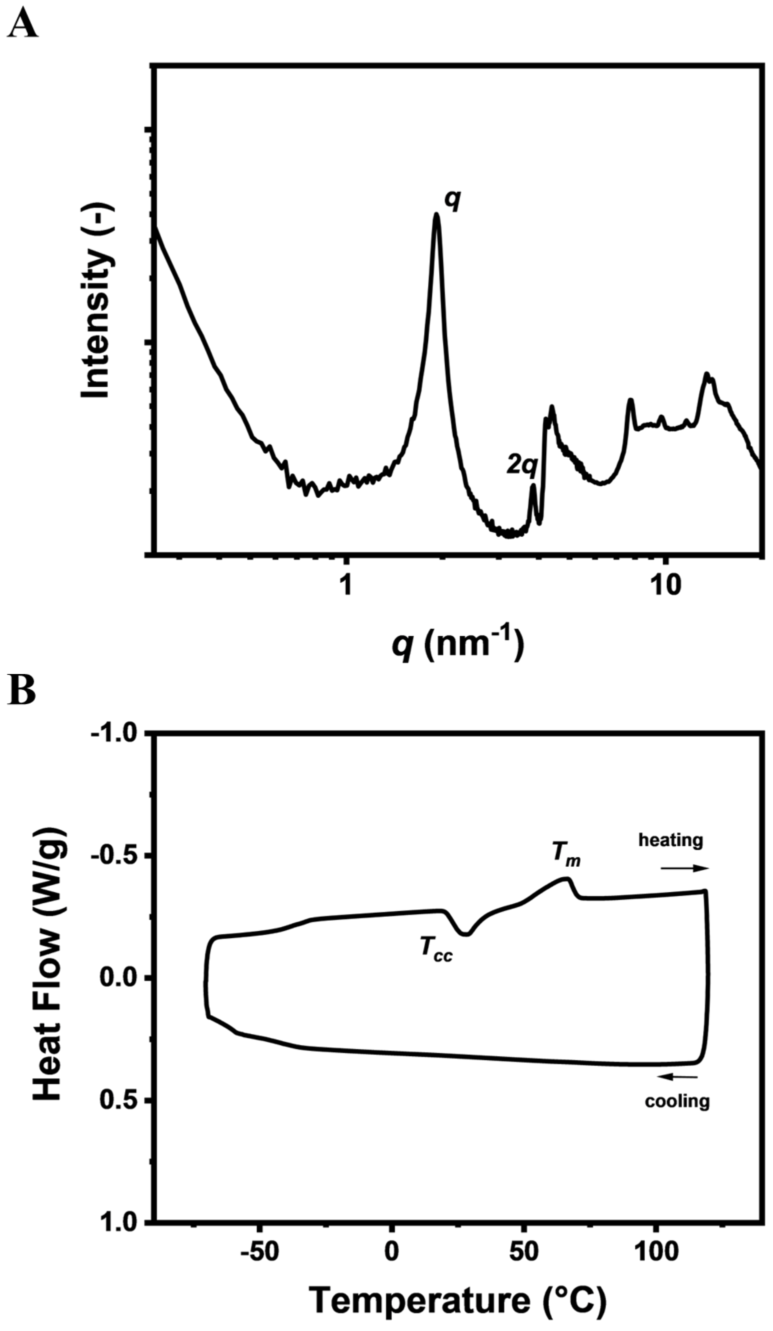

Figure S7: *SAXS and DSC characterization of* ***BnPhePhe-Si$_8$-BnPhePhe****. (A) 1D transmission scattering profile at room temperature showing a lamellar morphology (d_{lam} = 3.3 nm). (B) Differential scanning calorimetry trace of the second cycle with 10 K min^{-1} showing the melting and crystallization peaks (exothermic down).*

A

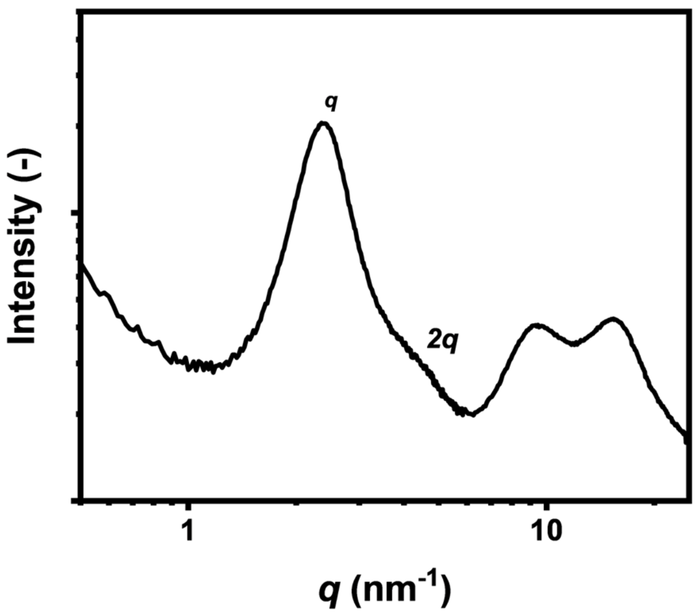

B

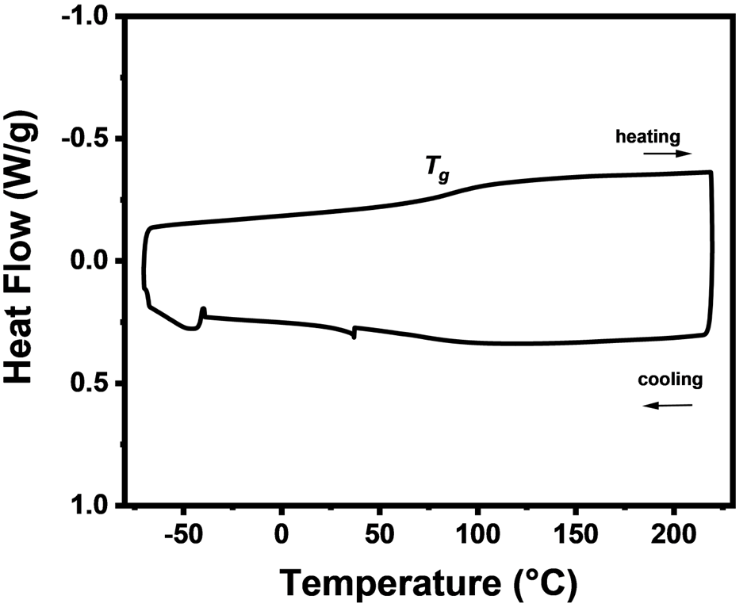

Figure S8: *SAXS and DSC characterization of* ***PhePhe-Si$_8$-PhePhe****. (A) 1D transmission scattering profile at room temperature showing a lamellar morphology (d_{lam} = 2.6 nm). (B) Differential scanning calorimetry trace of the second cycle with 10 K min^{-1} showing the melting and crystallization peaks (exothermic down).*

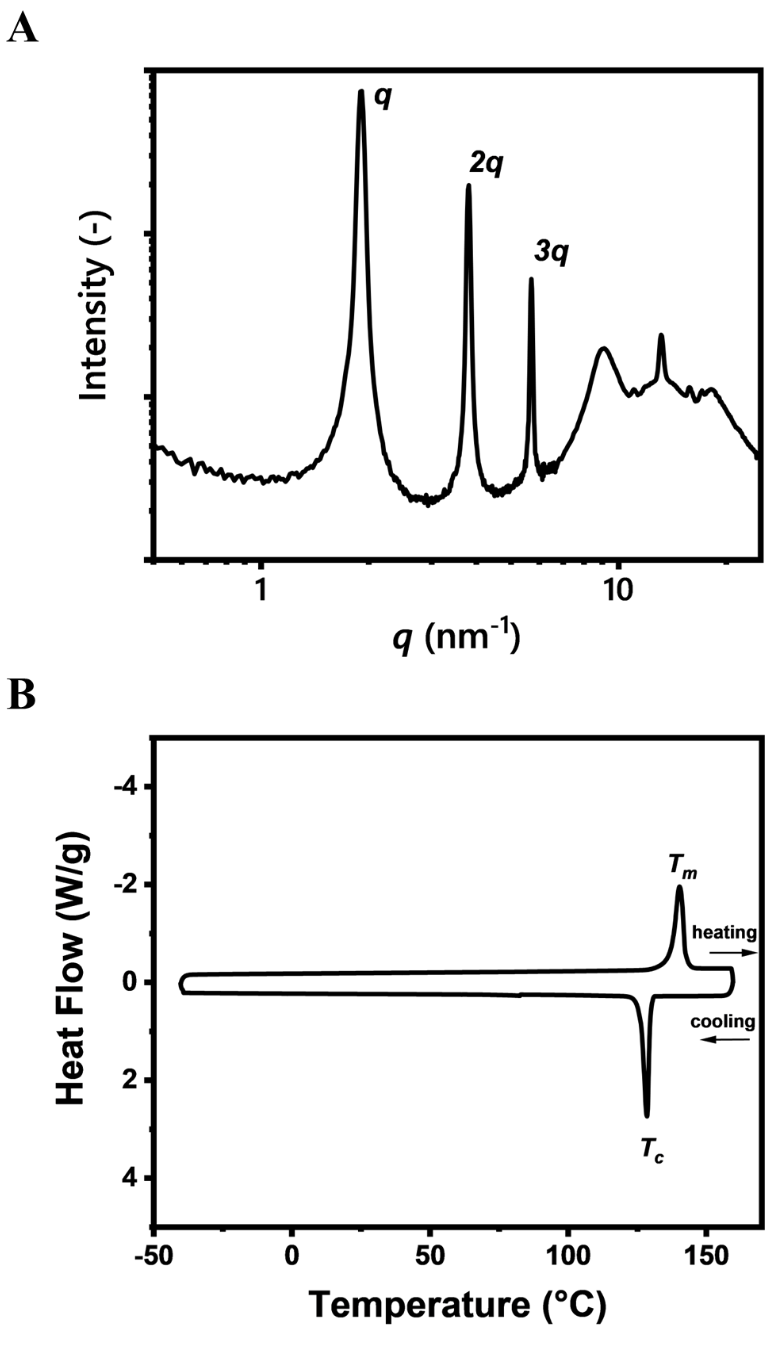

***Figure S9**: SAXS and DSC characterization of **PMDI-Si$_8$-PMDI**. (A) 1D transmission scattering profile at room temperature showing a lamellar morphology (d_{lam} = 3.3 nm). (B) Differential scanning calorimetry trace of the second cycle with 10 K min^{-1} showing the melting and crystallization peaks (exothermic down).*

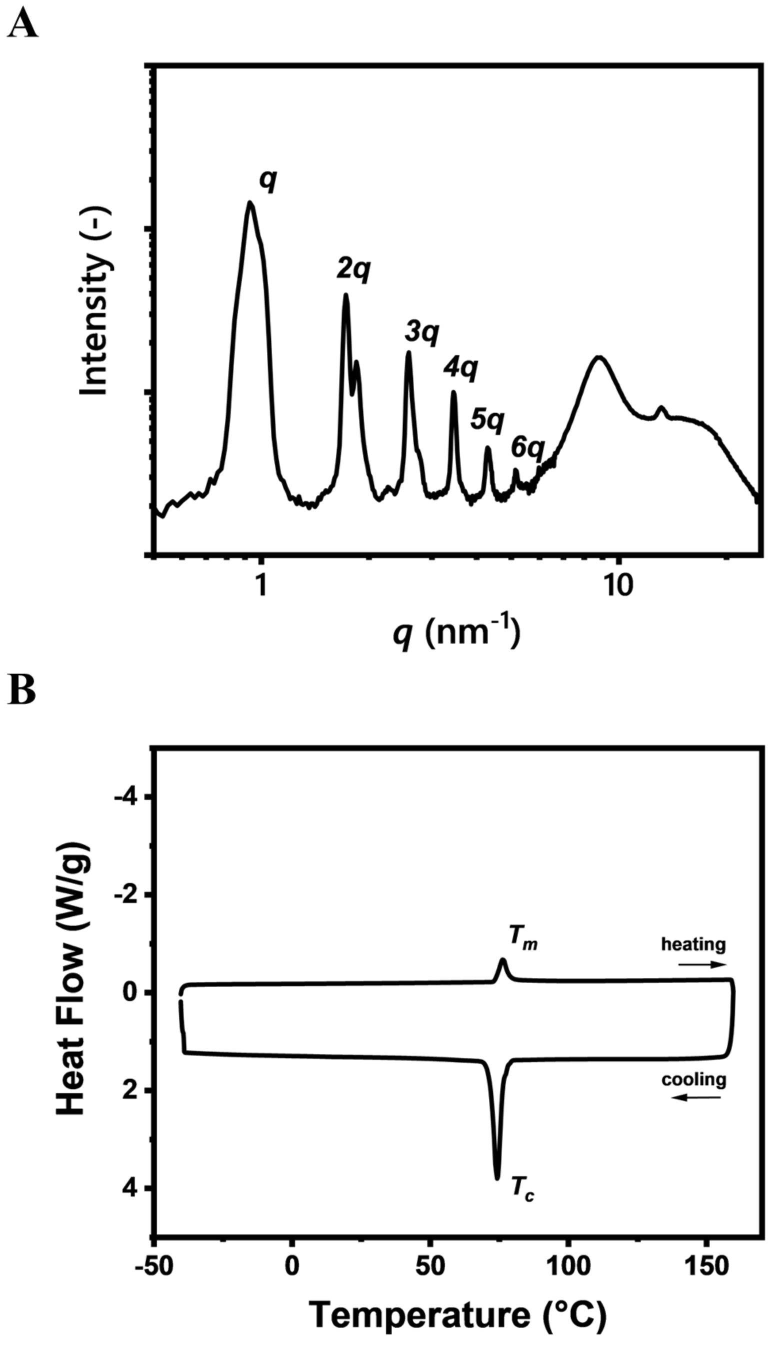

Figure S10: *SAXS and DSC characterization of **PMDI-Si$_{40}$-PMDI**. (A) 1D transmission scattering profile at room temperature showing a lamellar morphology (d_{lam} = 7.3 nm). (B) Differential scanning calorimetry trace of the second cycle with 10 K min^{-1} showing the melting and crystallization peaks (exothermic down).*

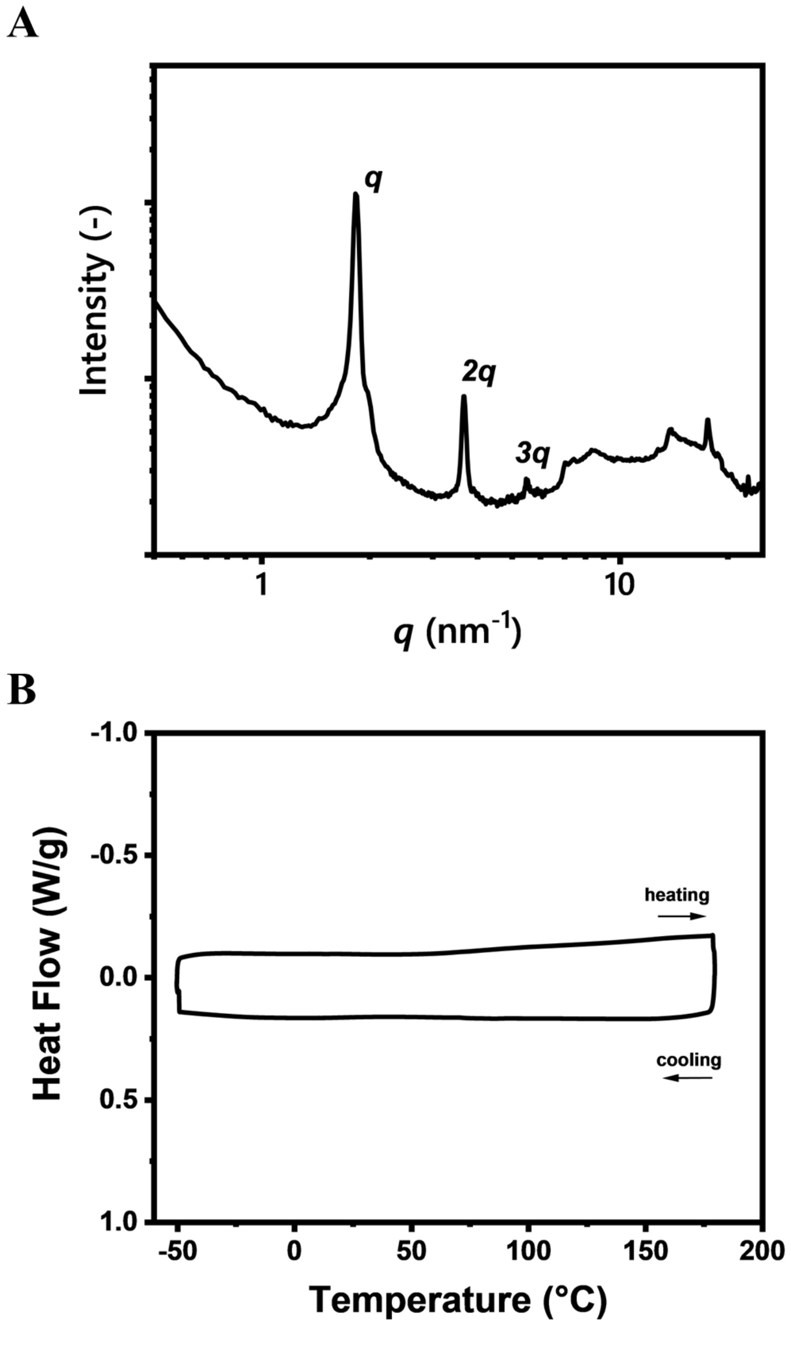

Figure S11: *SAXS and DSC characterization of* ***Triazine-Si$_8$-Triazine****. (A) 1D transmission scattering profile at room temperature showing a lamellar morphology (d_{lam} = 3.4 nm). (B) Differential scanning calorimetry trace of the second cycle with 10 K min^{-1} showing the melting and crystallization peaks (exothermic down).*

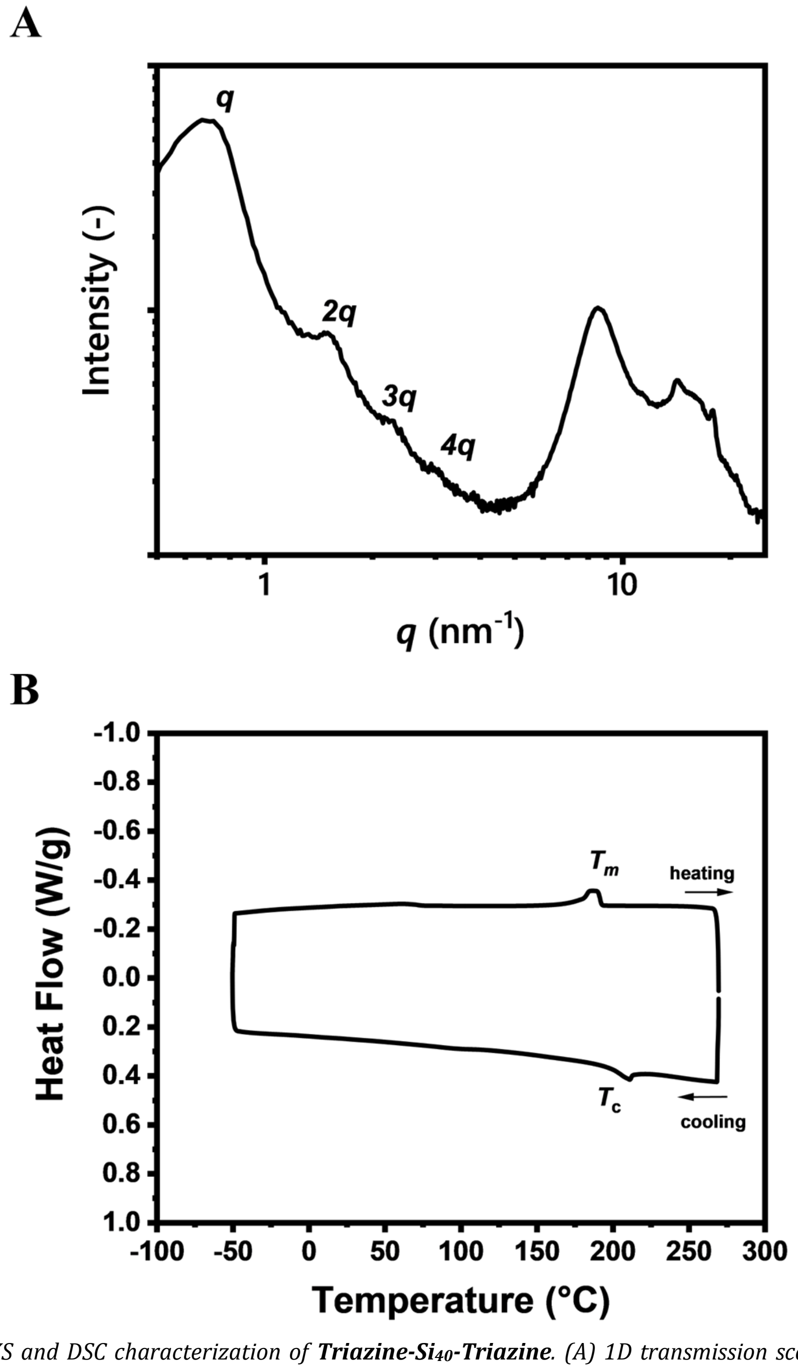

***Figure S12**: SAXS and DSC characterization of **Triazine-Si$_{40}$-Triazine**. (A) 1D transmission scattering profile at room temperature showing a lamellar morphology (d_{lam} = 9.1 nm). (B) Differential scanning calorimetry trace of the second cycle with 10 K min^{-1} showing the melting and crystallization peaks (exothermic down).*

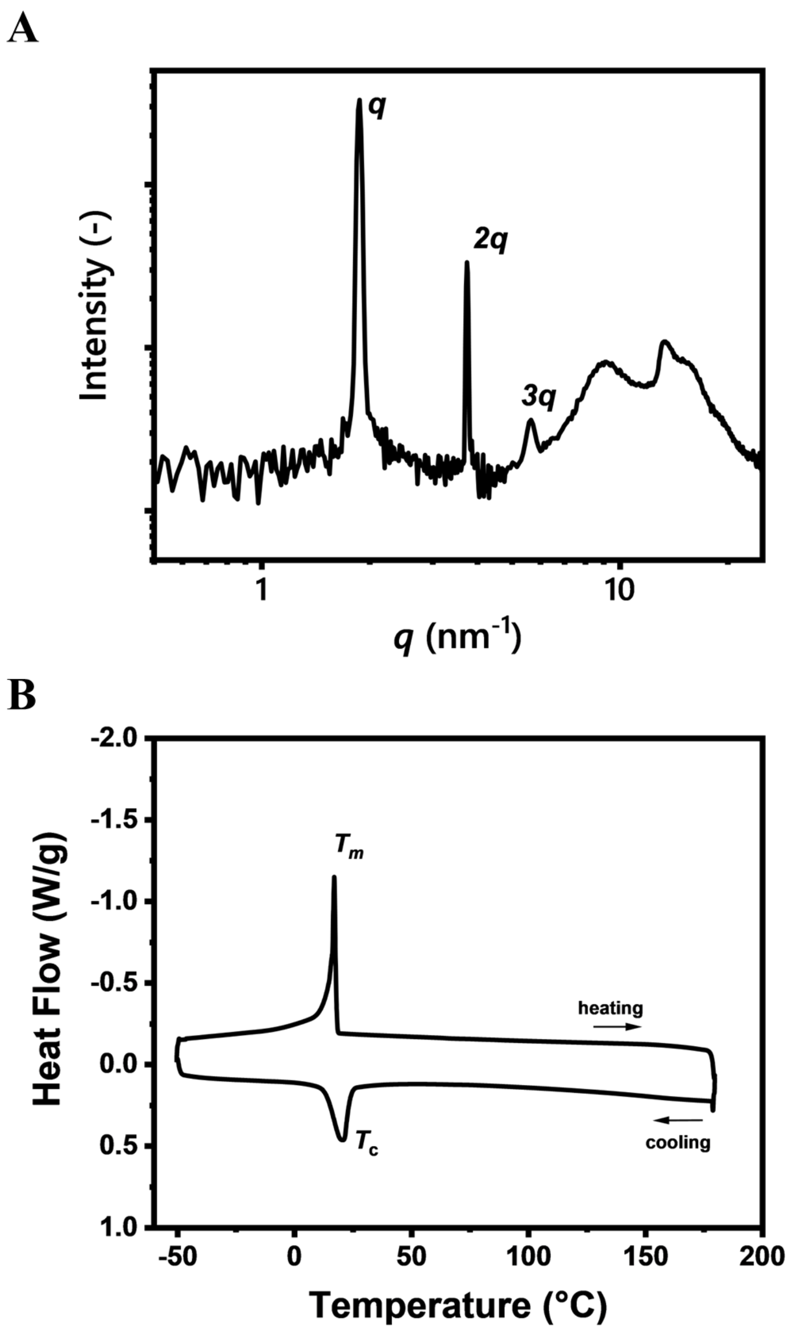

***Figure S13**: SAXS and DSC characterization of **Thiophene-Si$_8$-Thiophene**. (A) 1D transmission scattering profile at −20 °C showing a lamellar morphology (d_{lam} = 3.4 nm). (B) Differential scanning calorimetry trace of the second cycle with 10 K min^{-1} showing the melting and crystallization peaks (exothermic down).*

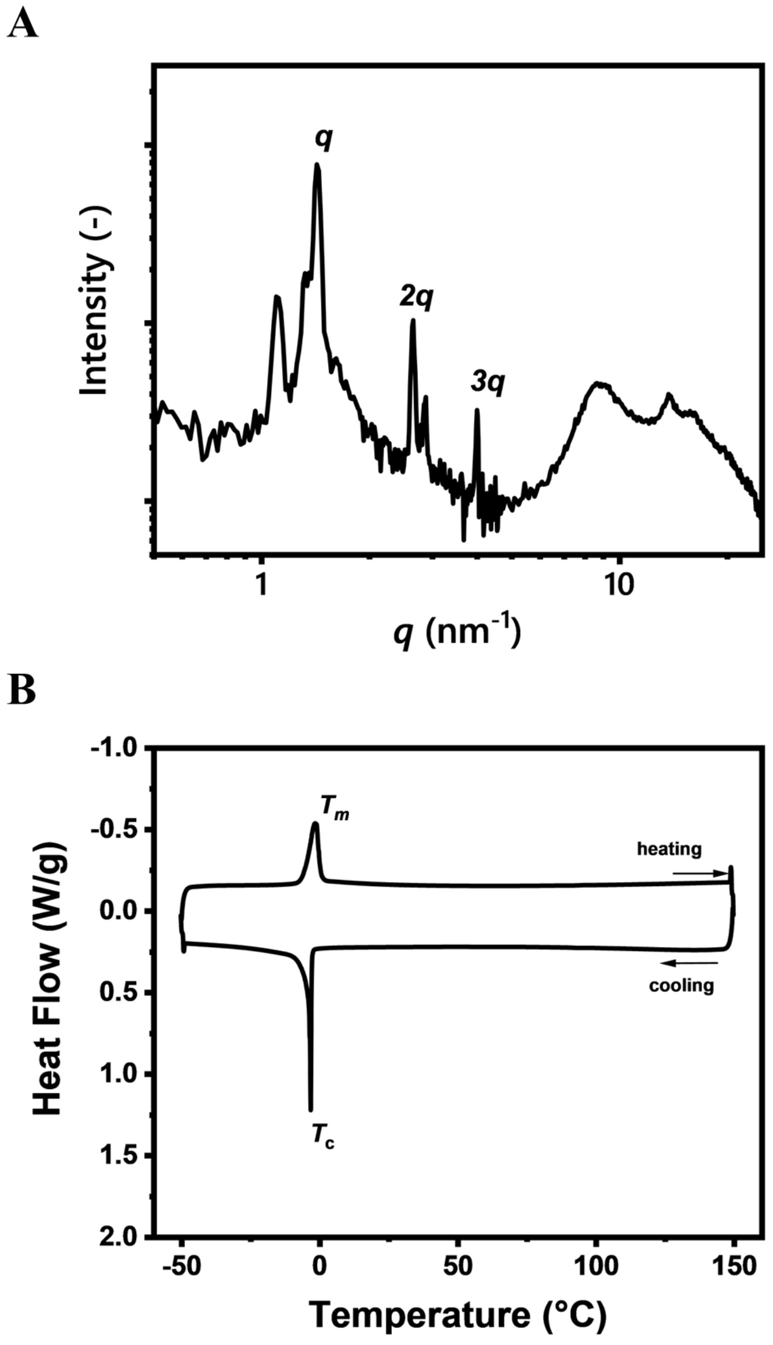

***Figure S14**: SAXS and DSC characterization of **Thiophene-Si$_{16}$-Thiophene**. (A) 1D transmission scattering profile at −20 °C showing a lamellar morphology (d_{lam} = 4.4 nm). (B) Differential scanning calorimetry trace of the second cycle with 10 K min^{-1} showing the melting and crystallization peaks (exothermic down).*

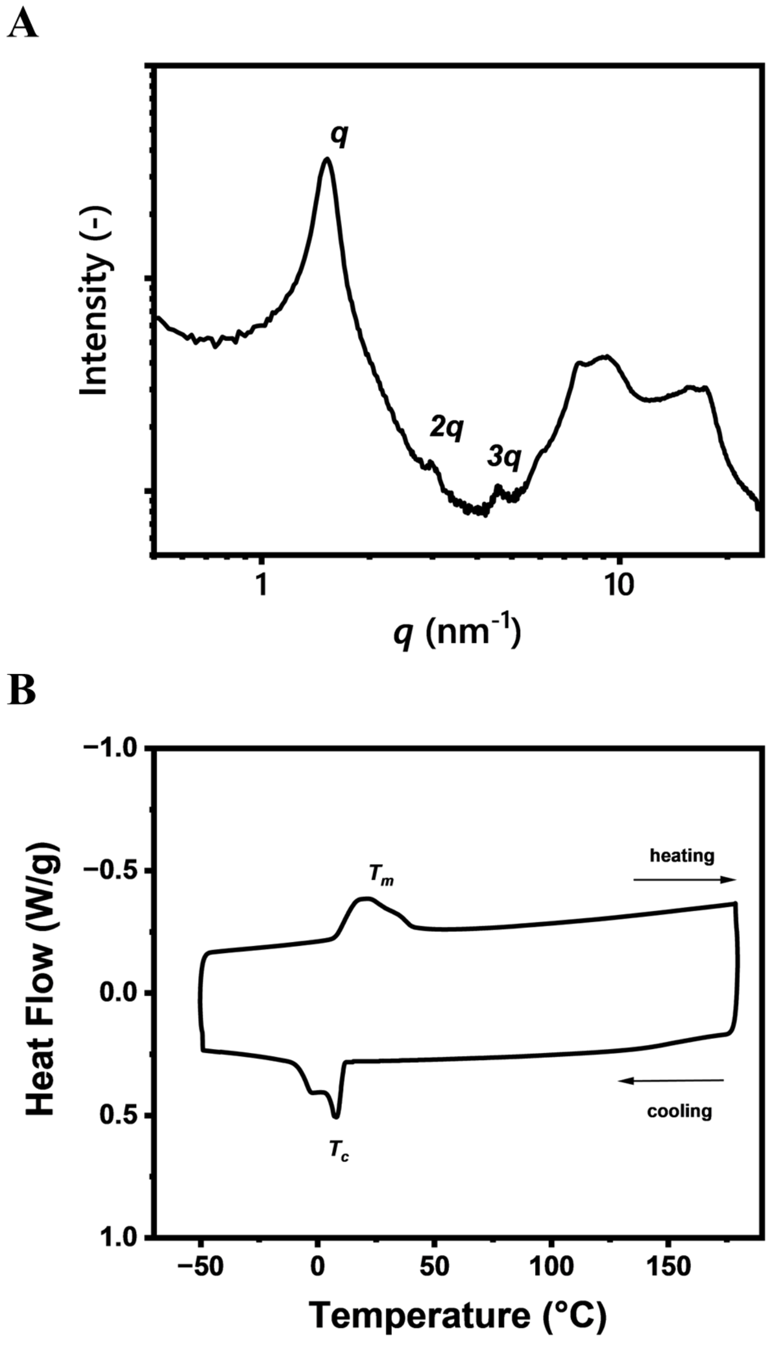

Figure S15: *SAXS and DSC characterization of **H-AQ-Si$_8$-AQ-H**. (A) 1D transmission scattering profile at −20 °C showing a lamellar morphology (d_{lam} = 4.1 nm). (B) Differential scanning calorimetry trace of the second cycle with 10 K min^{-1} showing the melting and crystallization peaks (exothermic down).*

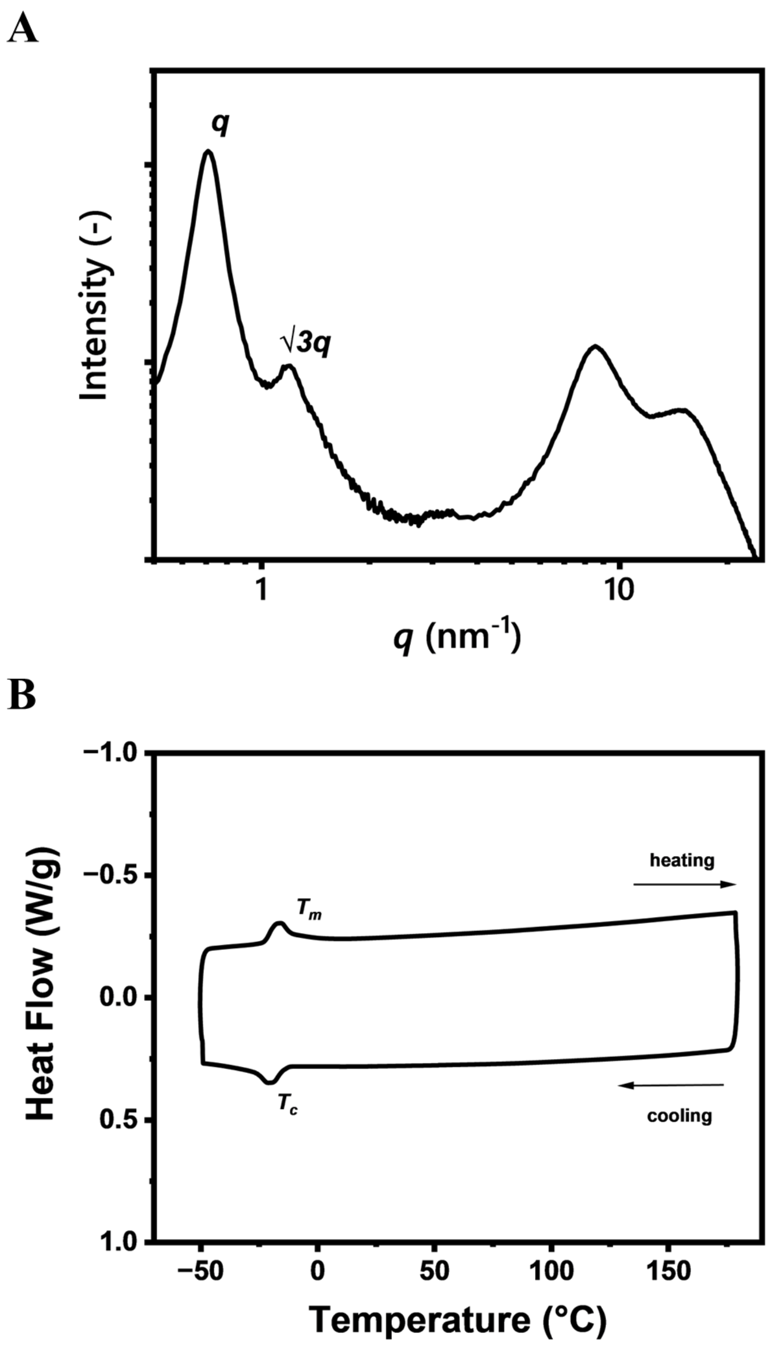

***Figure S16**: SAXS and DSC characterization of **H-AQ-Si$_{40}$-AQ-H**. (A) 1D transmission scattering profile at −50 °C showing a columnar hexagonal morphology (d_{col} = 8.8 nm). (B) Differential scanning calorimetry trace of the second cycle with 10 K min^{-1} showing the melting and crystallization peaks (exothermic down).*

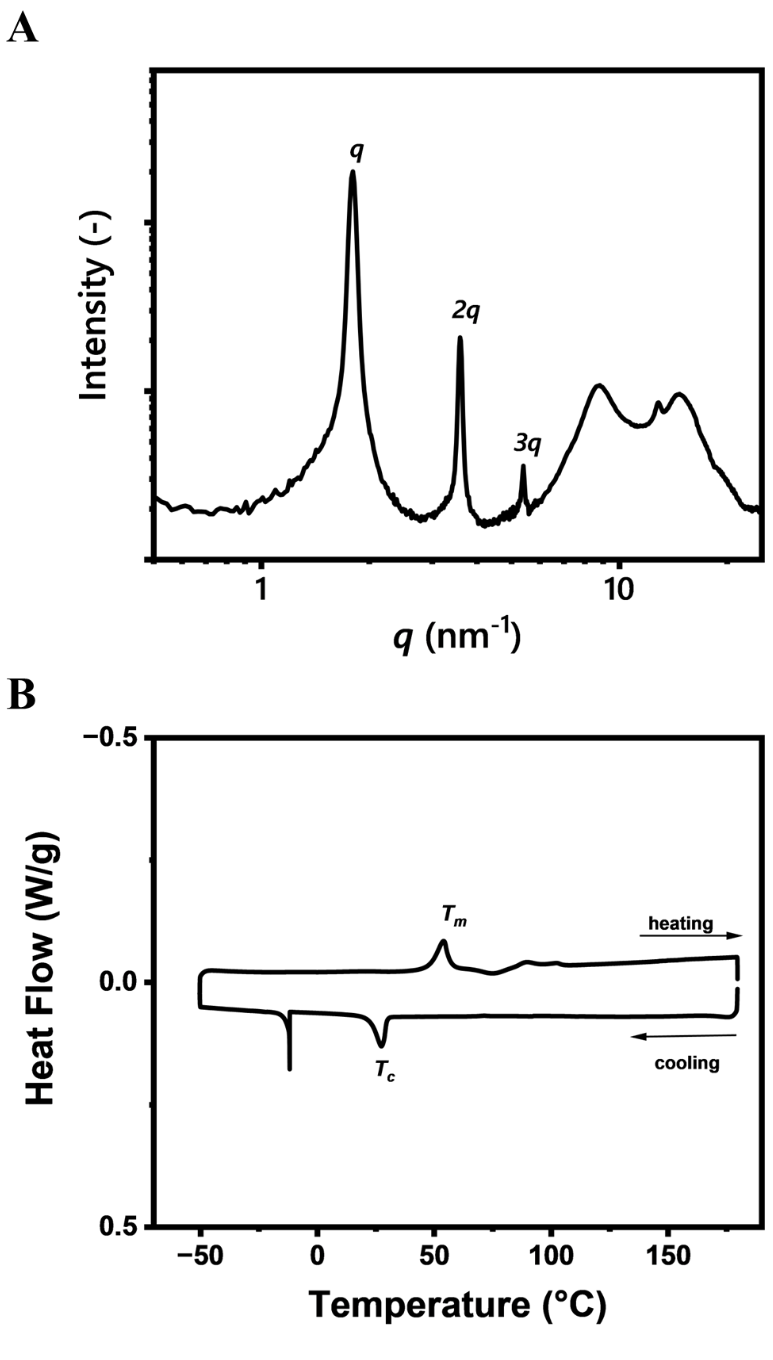

Figure S17: *SAXS and DSC characterization of* ***Acylhydz-Si$_8$-Acylhydz****. (A) 1D transmission scattering profile at 0 °C showing a lamellar morphology (d_{lam} = 3.5 nm). (B) Differential scanning calorimetry trace of the second cycle with 10 K min^{-1} showing the melting and crystallization peaks (exothermic down).*

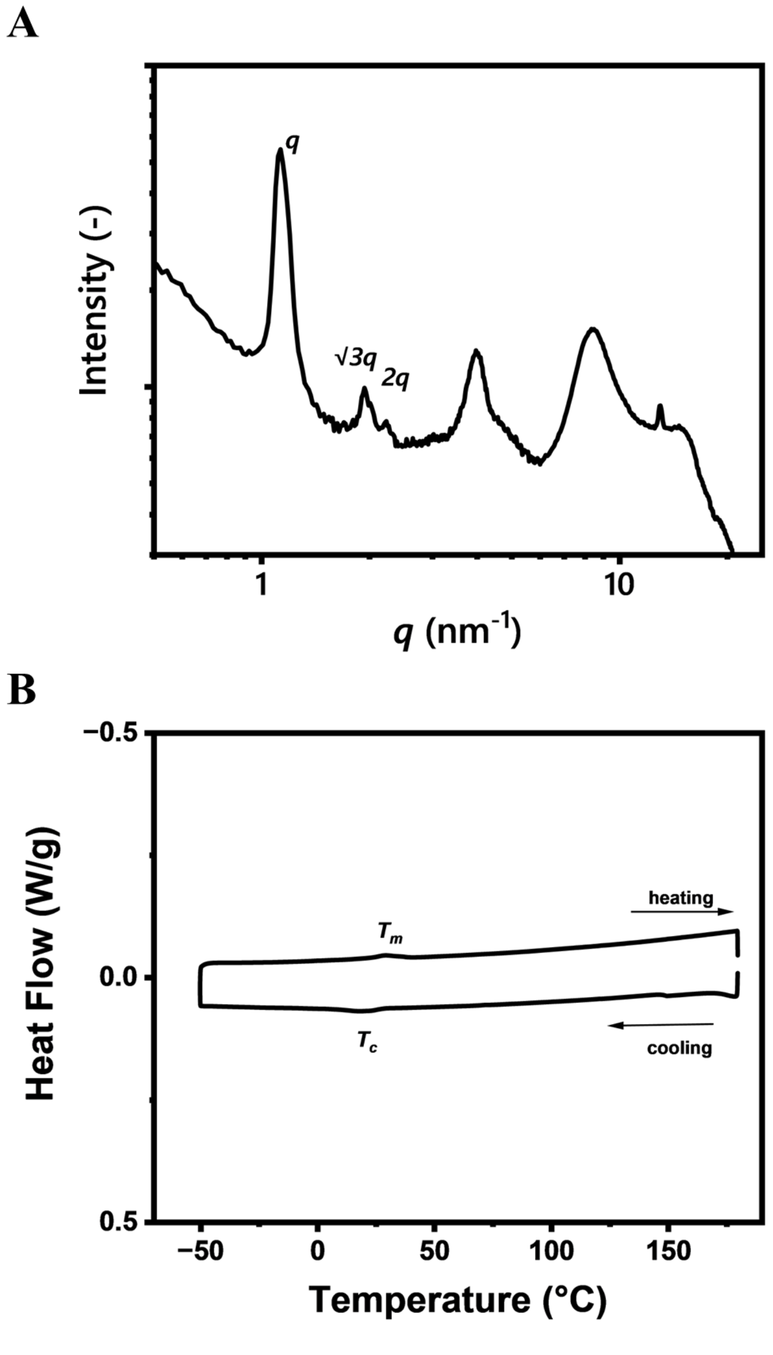

Figure S18: *SAXS and DSC characterization of* ***Acylhydz-Si$_{40}$-Acylhydz****. (A) 1D transmission scattering profile at −20 °C showing a columnar hexagonal morphology (d_{col} = 5.6 nm). (B) Differential scanning calorimetry trace of the second cycle with 10 K min^{-1} showing the melting and crystallization peaks (exothermic down).*

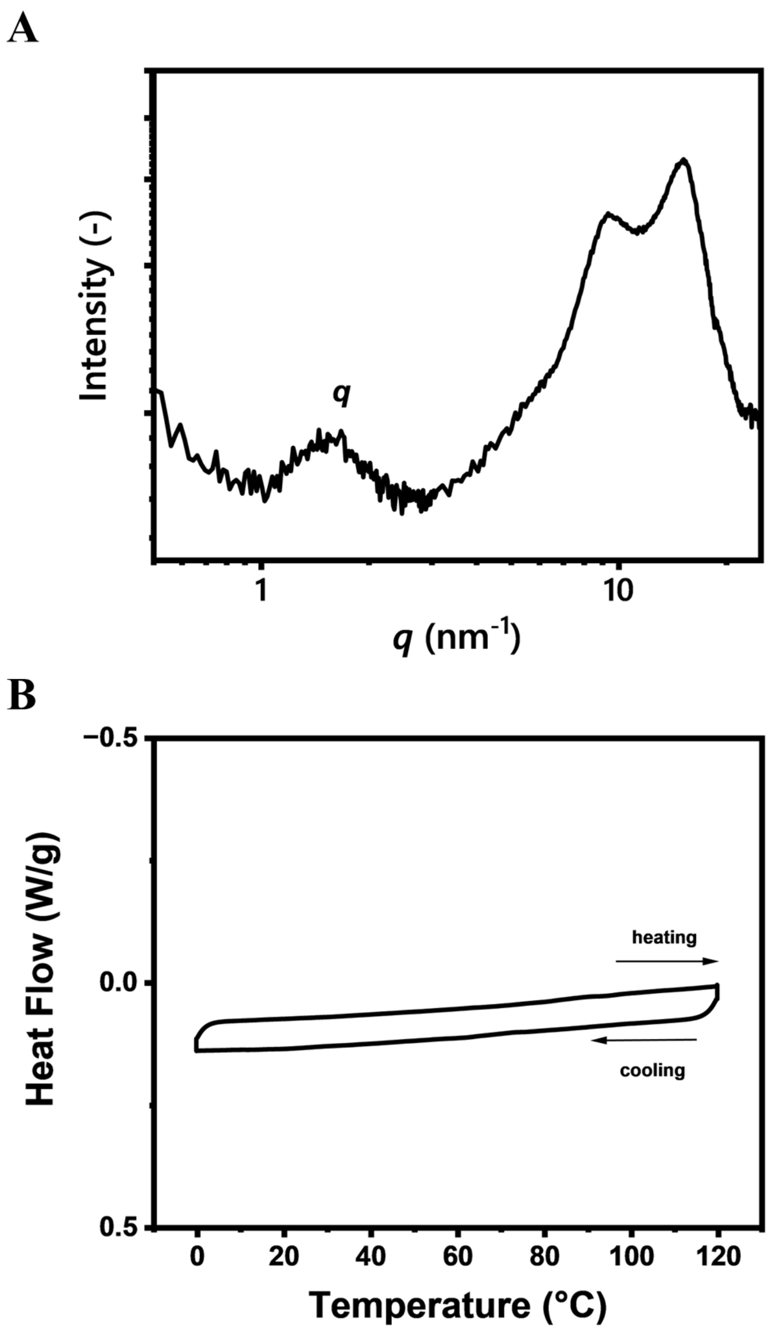

Figure S19: *SAXS and DSC characterization of* ***TIPSPent-Si$_{16}$-TIPSPent****. (A) 1D transmission scattering profile at room temperature showing a disordered morphology. (B) Differential scanning calorimetry trace of the second cycle with 10 K min^{-1} showing the melting and crystallization peaks (exothermic down).*

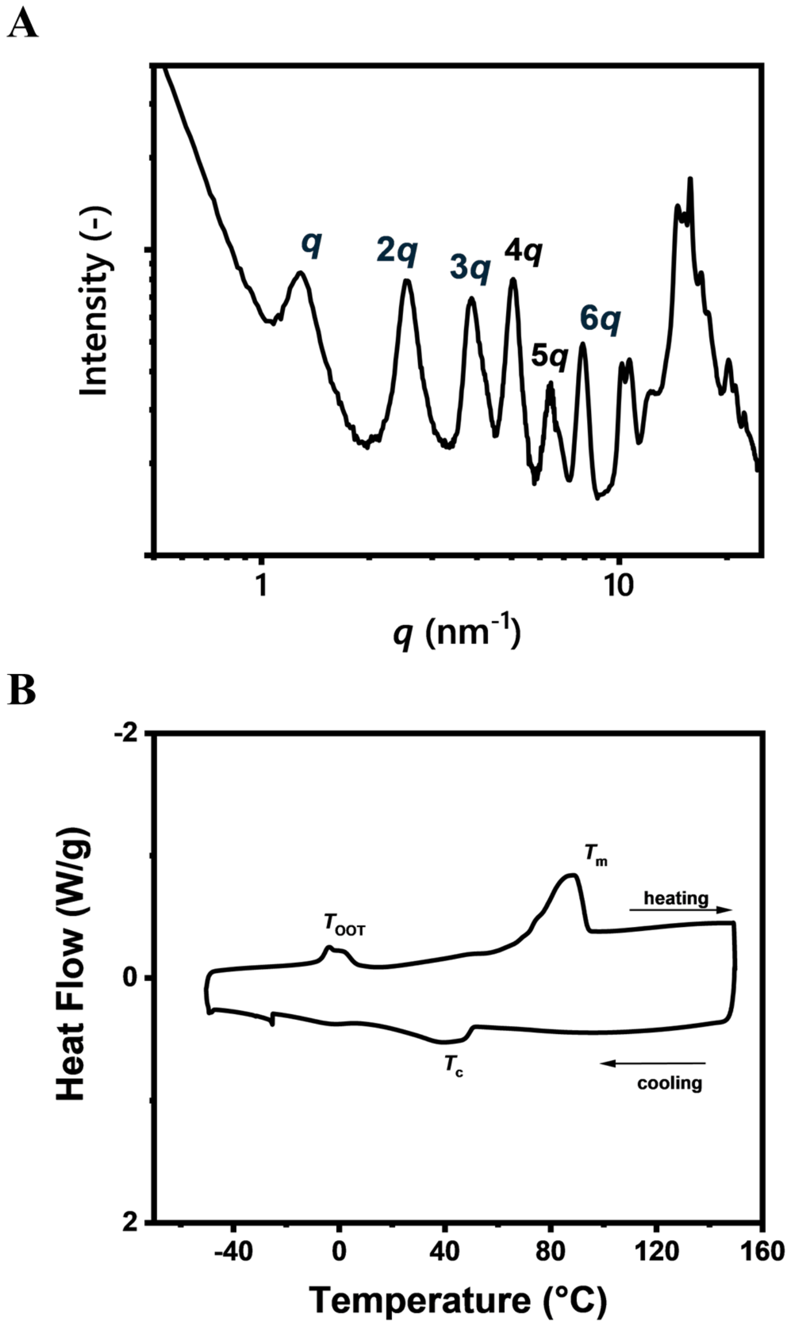

Figure S20: *SAXS and DSC characterization of* ***Val-C*** $_{18}$ ***-Val****. (A) 1D transmission scattering profile at −20 °C showing a lamellar morphology (*d_{lam} *= 4.8 nm). (B) Differential scanning calorimetry trace of the second cycle with 10 K min*$^{-1}$ *showing the melting and crystallization peaks (exothermic down).*

Table S1: Characteristics of Head-Tail block molecules. *[a]Volume fraction of the non-oDMS part of the molecule. [b]Identifiers of the crystal structures retrieved from the CCDC. [c]Distance between the connection points of the oDMS chains. [d]Predicted from the width of the crystalline layer and length of the spacer (d_{CH2} = 0.12 nm, d_{oDMS} = 0.155 nm). [e]Morphology determined from the pattern of the Bragg's reflection peaks in SAXS spectra (LAM = lamellar, CYL = cylindrical, CYL,OBL = cylindrical oblique, Dis = disordered). [f]Calculated using the formula $d_{exp} = 2\pi/q$. [g]Measured distance contradicts the observed morphology. [h]Crystal structure was determined in this work and shown in SI 5.*

**Crystalline unit**	**# *o*DMS**	**$\varphi_{\text{non-}o\text{DMS}}$[a] (-)**	**1. Crystal Structure?[b]**	**2. Ordering in layer?**	**3. Overlap Energy Framework**	**4. Intramol. distance[c] (Å)**	**5. Pred. d_{lam}[d] (nm)**	**Morph.[e]**	**d_{exp}[f] (Å)**
BnoLLA11	23	0.36	-	-	-	-	-	CYL	65
BnoLLA13	23	0.24	-	-	-	-	-	CYL	71
BnoLLA15	23	0.23	-	-	-	-	-	GYR	74
BnoLLA15	27	0.5	-	-	-	-	-	CYL	80
BnoLLA17	15	0.2	-	-	-	-	-	LAM	71
BnoLLA17	23	0.12	-	-	-	-	-	GYR	78
BnoLLA25	23	0.21	-	-	-	-	-	LAM	86
BnoLLA25	27	0.52	-	-	-	-	-	LAM	93
BnoLLA33	59	0.67	-	-	-	-	-	CYL	137
BnoLLA9	15	0.15	-	-	-	-	-	Dis	-
BnoLLA9	23	0.1	-	-	-	-	-	Dis	-
DPA	15	0.23	**DPANTR**	LAM	YES (−192.2 kJ/mol)	9.4	44	LAM	58
HAQ	15	0.2	**ANTQUO**	LAM	YES (−134.8 kJ/mol)	8.7 [g]	35	CYL	57
HOPV	15	0.23	**REDHIR02**	LAM	YES (−288.4 kJ/mol)	6.8	-	CYL,OBL	55x61
mCP	4	0.51	**IFOREC**	NO	-	-	-	LAM	32
MeAQ	15	0.22	**ANTQUO**	LAM	YES (−134.8 kJ/mol)	8.7 [g]	37	CYL	56
MeOoPro6	15	0.18	-	-	-	-	-	LAM	72
MeOoPro9	15	0.31	-	-	-	-	-	LAM	82
MeOPV	15	0.21	**REDHIR02**	LAM	YES (−288.4 kJ/mol)	6.8	-	CYL,OBL	57x61
NH2oPro3	15	0.51	-	-	-	-	-	CYL	48
NH2oPro6	15	0.13	-	-	-	-	-	LAM	68
NitroHydr	15	0.19	**YEFFAR**	LAM	YES (−288.4 kJ/mol)	6.2	-	CYL	49
oMe33	7	0.2	-	-	-	-	-	LAM	56
oMe33	11	0.21	-	-	-	-	-	CYL	70
oMe33	15	0.15	-	-	-	-	-	CYL	78
oMe69	15	0.23	-	-	-	-	-	LAM	112
oMe69	23	0.31	-	-	-	-	-	LAM	125
PentAQ	15	0.28	**S.I. 5 [h]**	LAM	YES (−282.4 kJ/mol)	8.7	41	LAM	59
PentAzo	15	0.22	**AzPhen10**	LAM	YES (−204.0 kJ/mol)	7.6	41	LAM	63
PentOPV	15	0.25	**REDHIR02**	LAM	YES (−288.4 kJ/mol)	6.8	-	CYL,OBL	58x65
TIPSPent	7	0.54	-	-	-	-	-	CYL	2.7
	15	0.36		-	-	-	-	LAM	4.5

Table S2: Characteristics of Center-functionalized block molecules. *[a]Volume fraction of the non-oDMS part of the molecule. [b]Identifiers of the crystal structures retrieved from the CCDC. [c]Distance between the connection points of the oDMS chains. [d]Predicted from the width of the crystalline layer and length of the spacer (d_{CH2} = 0.12 nm, d_{oDMS} = 0.155 nm). [e]Morphology determined from the pattern of the Bragg's reflection peaks in SAXS spectra (LAM = lamellar, CYL = cylindrical, CYL,OBL = cylindrical oblique, Dis = disordered). [f]Calculated using the formula $d_{exp} = 2\pi/q$.*

Crystalline unit	# *o*DMS	$\varphi_{non\text{-}oDMS}$[a] (-)	1. Crystal Structure?[b]	2. Ordering in layer?	3. Overlap Energy Framework	4. Intramol. distance[c] (Å)	5. Pred. d_{lam}[d] (Å)	Morph.[e]	d_{exp}[f] (Å)
Br-PDPP	7	0.26	**XATJAE**	LAM	NO	-	-	Dis	-
	3	0.45		LAM	NO	-	-	LAM	27
DiPhePent	7	0.34	**WUPJOI**	NO	-	-	-	Dis	-
	11	0.25		NO	-	-	-	LAM	47
	15	0.19		NO	-	-	-	LAM	58
DPA	7	0.28	**DPANTR**	LAM	YES (−192.2 kJ/mol)	9.4	36	LAM	32
	11	0.19		LAM	YES (−192.2 kJ/mol)	9.4	49	LAM	43
	15	0.15		LAM	YES (−192.2 kJ/mol)	9.4	61	LAM	57
DPND	7	0.4	-	-	-	-	-	Dis	-
NDI	7	0.26	**DAHMUX**	LAM	YES (−213.2 kJ/mol)	4.6	-	CYL,OBL	93x29
	7	0.27		LAM	YES (−213.2 kJ/mol)	4.6	-	CYL	61
	15	0.14		LAM	YES (−213.2 kJ/mol)	4.6	-	CYL	65
	15	0.15		LAM	YES (−213.2 kJ/mol)	4.6	-	CYL	74
	23	0.1		LAM	YES (−213.2 kJ/mol)	4.6	-	CYL,OBL	77x68
	23	0.1		LAM	YES (−213.2 kJ/mol)	4.6	-	CYL	88
oMe66	7	0.51	-	-	-	-	-	LAM	59
PentAQ	7	0.22	**S.I. 5** [h]	LAM	YES (−282.4 kJ/mol)	4.4	-	CYL	37
PentAzo	7	0.23	**AzPhen10**	LAM	YES (−204.0 kJ/mol)	3.8	-	CYL,OBL	34x41
PentOPV	7	0.24	**REDHIR02**	LAM	YES (−288.4 kJ/mol)	6.8	-	CYL,OBL	35x40
TTF	15	0.11	**HITTOU**	LAM	YES (−187.4 kJ/mol)	7.4	69	LAM	49
TDPP	7	0.25	**OVOVUS01**	LAM	YES (−191.2 kJ/mol)	4.6	-	Dis	-

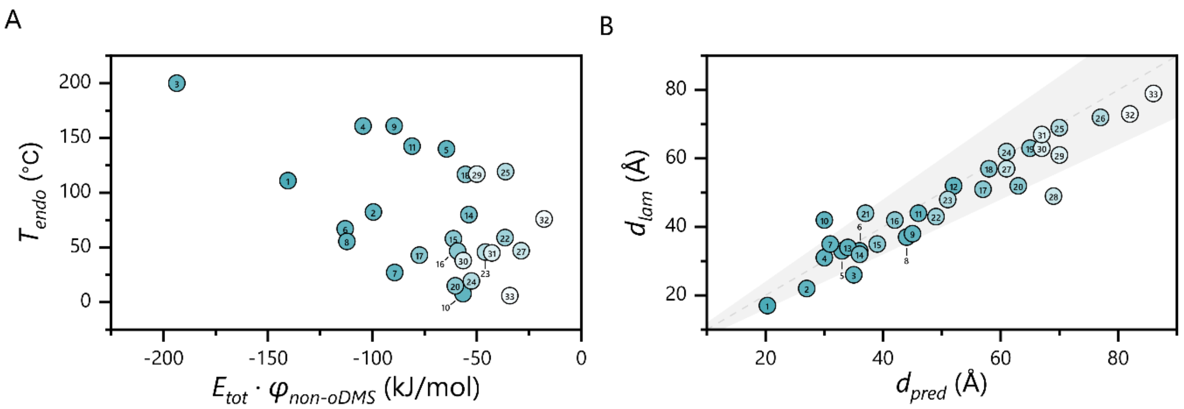

Figure S21: *Enlarged version of Figure 4, showing indexes for all datapoints (Table S3). The dashed line in B indicates the perfect correlation of predicted vs observed lamellar spacings, where the gray area displays ± 10% error.*

Table S3: *Table of entries displayed in Figure S20.*

Entry	Crystalline moiety	# *o*DMS	$\varphi_{non\text{-}oDMS}$ (-)	$E_{tot}\cdot\varphi_{non\text{-}oDMS}$ (kJ/mol)	T_{endo} (°C)	Pred. d_{lam} (Å)	Obs. d_{lam} (Å)
1	BnUPy	4	0.55	−140.4	111	20	17
2	BnUPy	8	0.39	−99.5	82.4	27	22
3	PhePhe	8	0.53	−193.6	200	35	26
4	NDI	8	0.49	−104.5	160.9	30	31
5	PMDI	8	0.33	−64.5	140	33	33
6	Pyrene	8	0.43	−113	67	36	33
7	AcylHydr	8	0.47	−89.3	27.1	31	35
8	MeAzo	8	0.55	−112.2	55.5	44	37
9	NDI	8	0.42	−89.5	161	45	38
10	HAQ	8	0.42	−56.6	7.8	30	42
11	NDI	8	0.38	−81	142.5	46	44
12	NDI-NDI	8	0.57	n.o.	n.o.	52	52
13	Thiophene	8	0.44	n.o.	50.6	34	34
14	DPA	14	0.28	−53.8	80	36	32
15	BnUPy	16	0.24	−61.2	58	39	35
16	MeAzo	16	0.29	−59.2	46.7	42	42
17	MeAzo	16	0.38	−77.5	43	57	51
18	NDI	16	0.26	−55.4	116.7	58	57
19	NDI-NDI	16	0.42	n.o.	n.o.	65	63
20	2-HOMeAzo	16	0.38	−60.3	15	63	52
21	Thiophene	16	0.28	n.o.	19.4	37	44
22	DPA	22	0.19	−36.5	59	49	43
23	BnUPy	24	0.18	−45.9	45.8	51	48
24	Pyrene	24	0.2	−52.6	19.4	61	62
25	NDI	24	0.17	−36.2	119.3	70	69
26	NDI-NDI	24	0.3	n.o.	n.o.	77	72
27	DPA	30	0.15	−28.8	47	61	57
28	TTF	30	0.11	n.o.	n.o.	69	49
29	DPA	32	0.26	−76	117	70	61
30	Pent-AQ	32	0.2	−56.5	38	67	63
31	Pent-Azo	32	0.21	−42.8	45	67	67
32	PMDI	40	0.09	−17.8	76	82	73
33	Pyrene	40	0.13	−34.2	6	86	79

4. NMR spectra

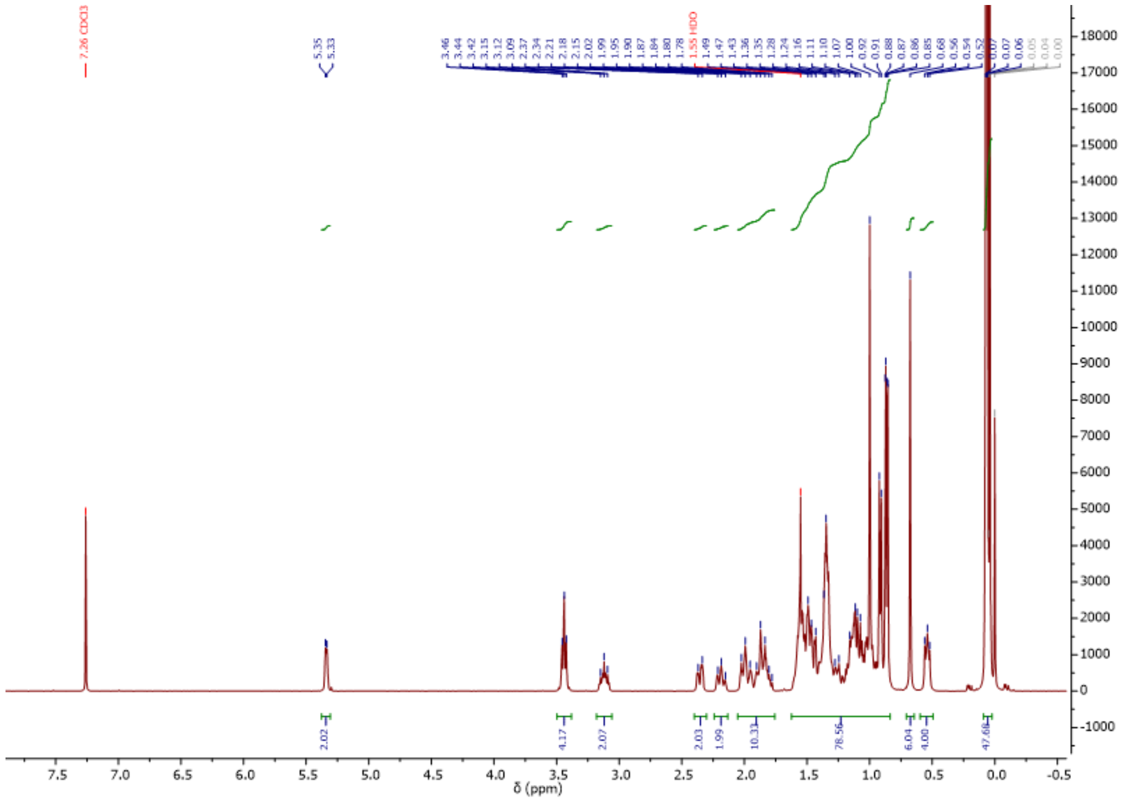

Figure S22: *^{1}H NMR (400 MHz, $CDCl_3$) spectrum of* ***Chol-Si$_8$-Chol***.

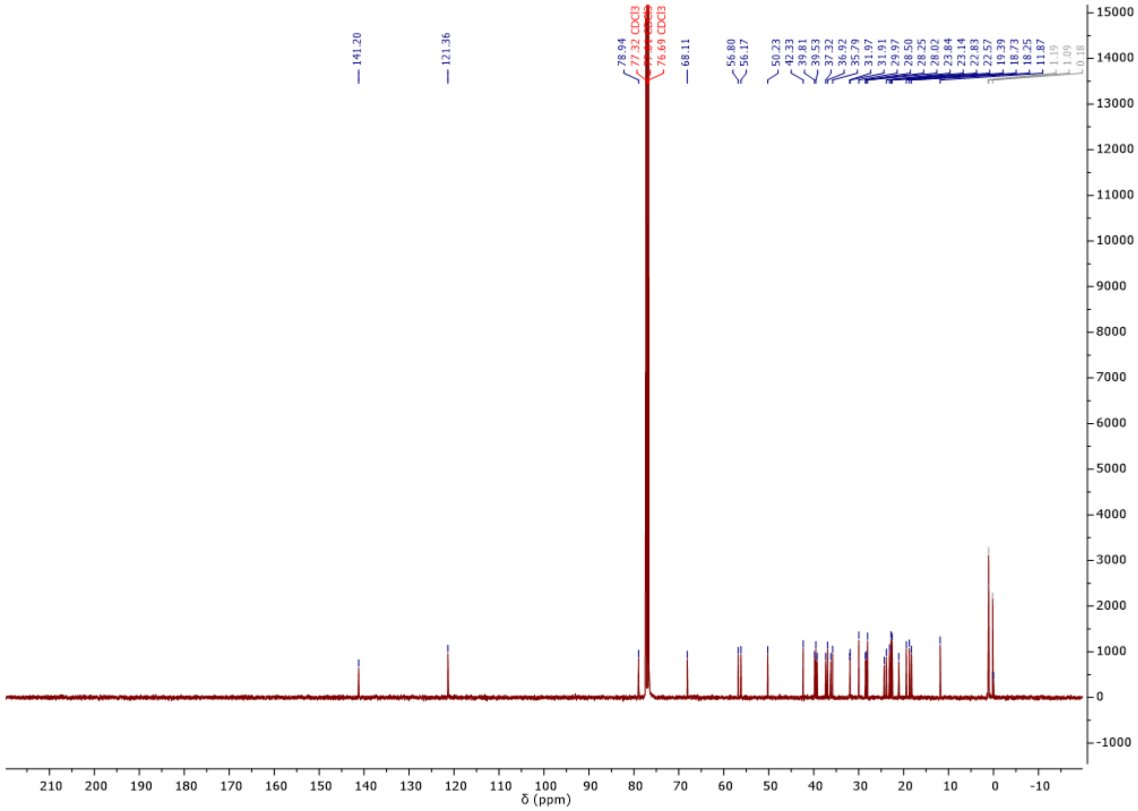

Figure S23: *^{13}C NMR (100 MHz, $CDCl_3$) spectrum of* ***Chol-Si$_8$-Chol***.

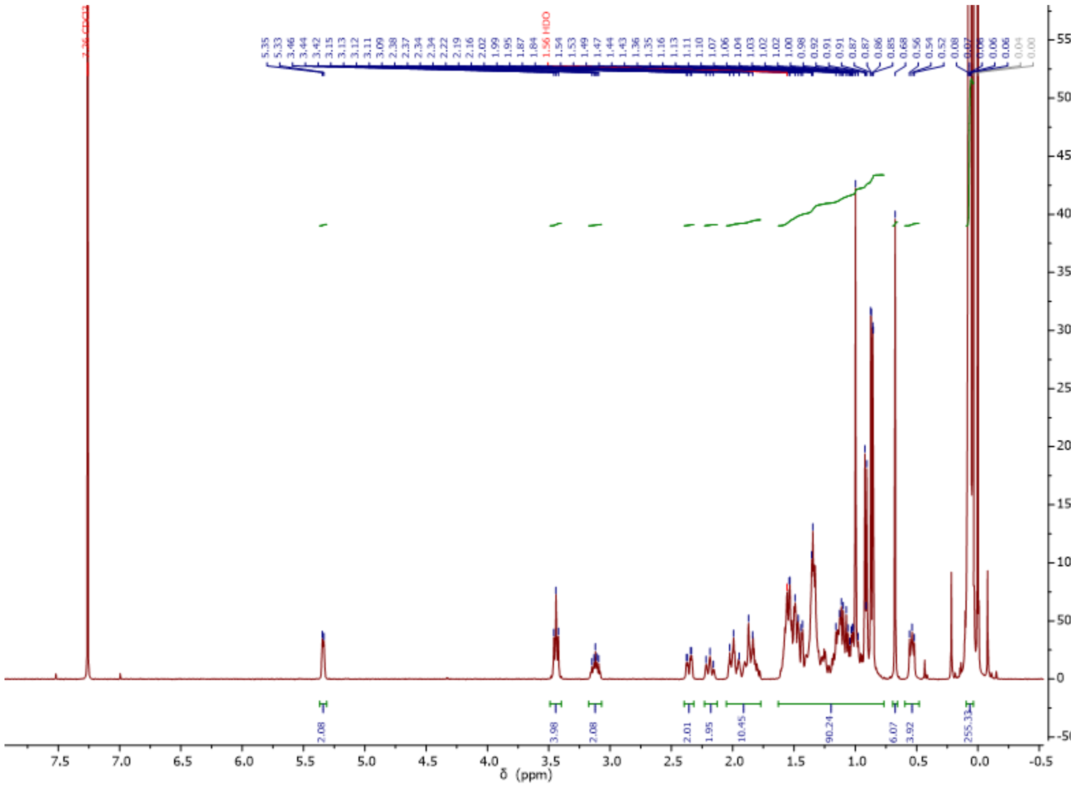

Figure S24: *^{1}H NMR (400 MHz, CDCl$_3$) spectrum of **Chol-Si$_{40}$-Chol**.*

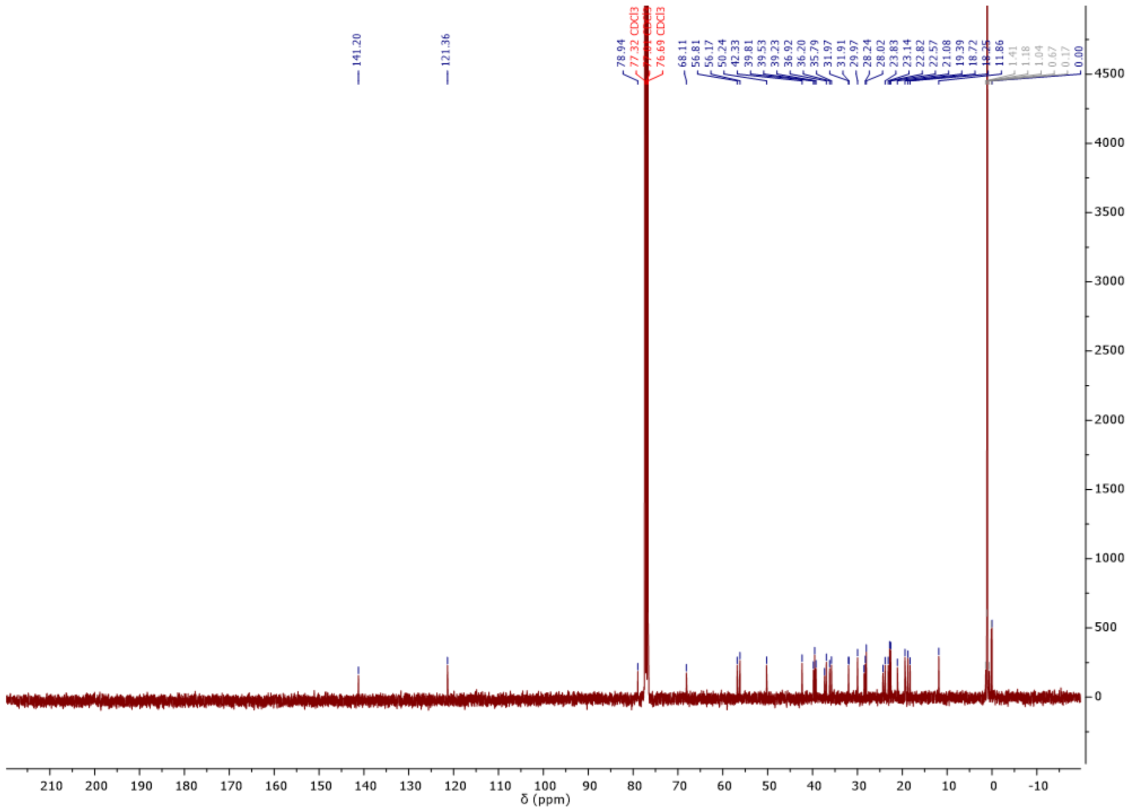

Figure S25: *^{13}C NMR (100 MHz, CDCl$_3$) spectrum of **Chol-Si$_{40}$-Chol**.*

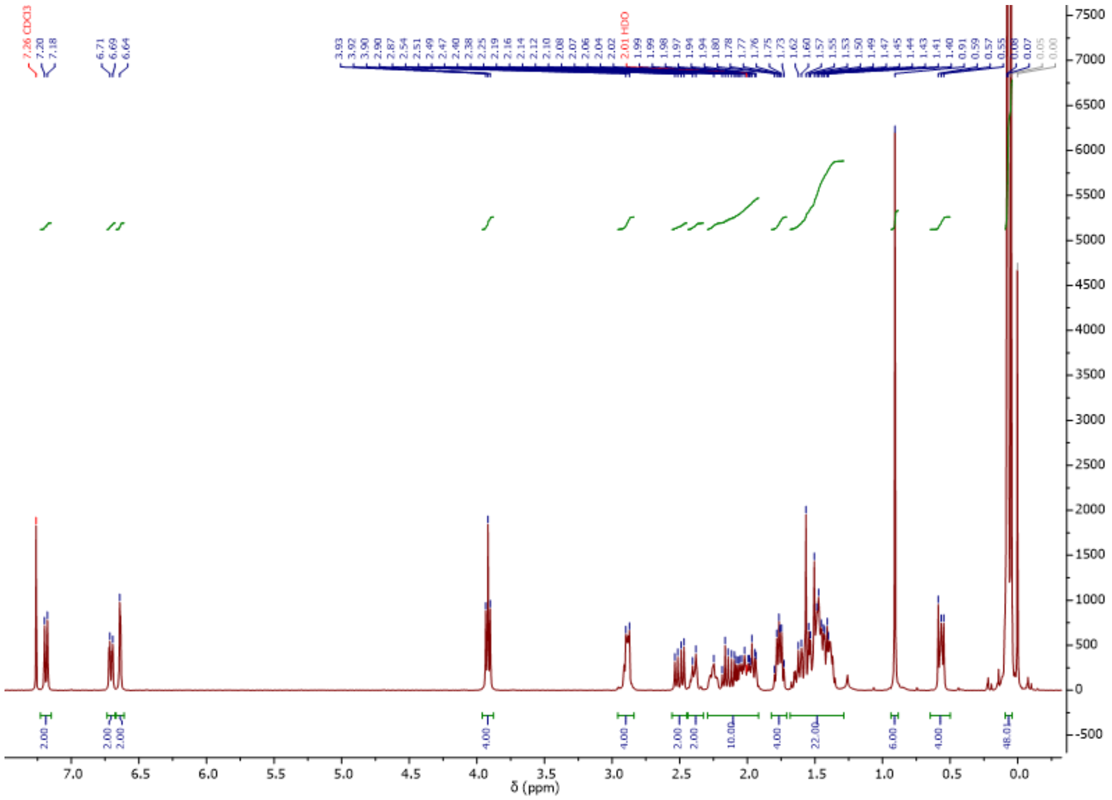

Figure S26: *^{1}H NMR (400 MHz, CDCl$_3$) spectrum of* ***Est-Si$_8$-Est***.

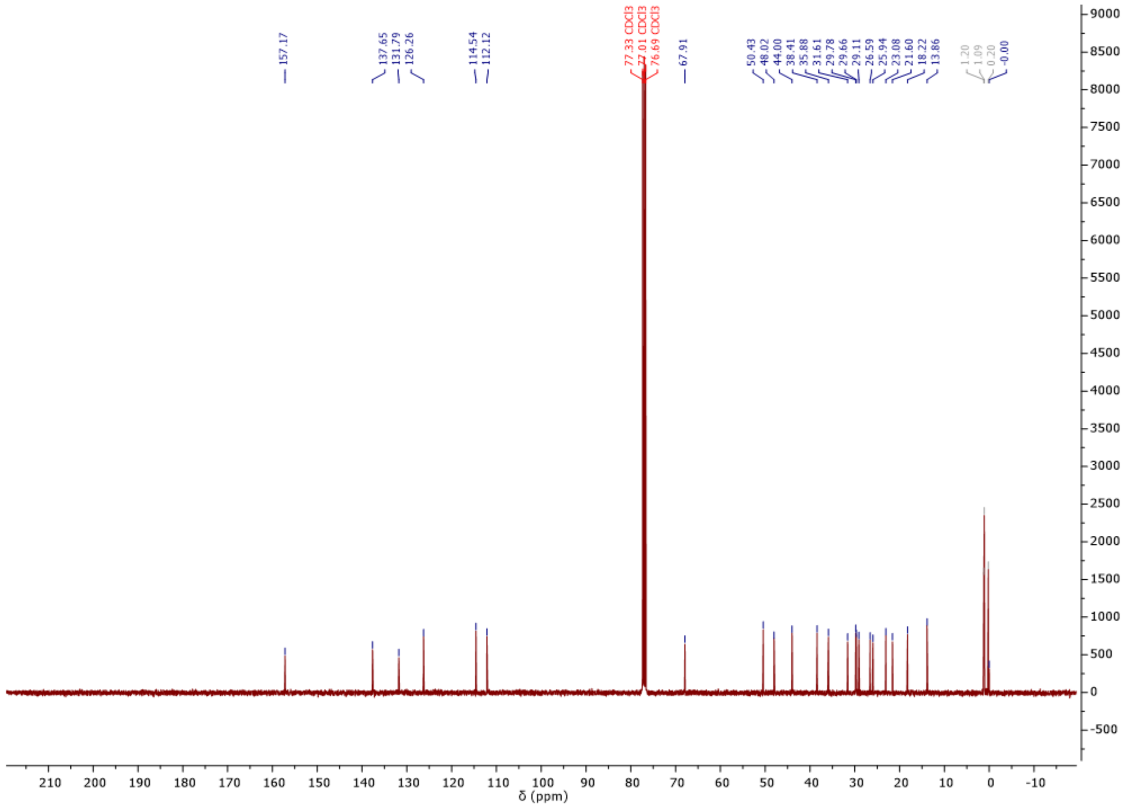

Figure S27: *^{13}C NMR (100 MHz, CDCl$_3$) spectrum of* ***Est-Si$_8$-Est***.

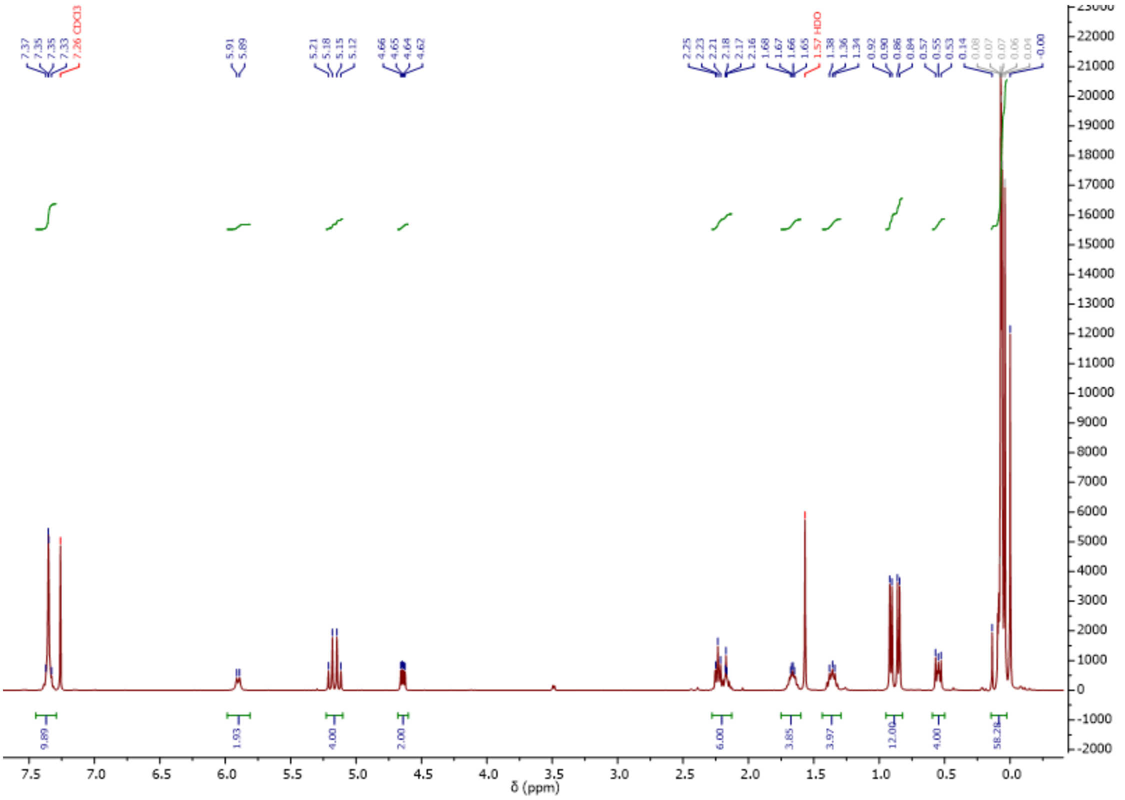

Figure S28: *^{1}H NMR (400 MHz, CDCl$_3$) spectrum of* ***BnO-Val-Si$_8$-Val-OBn****.*

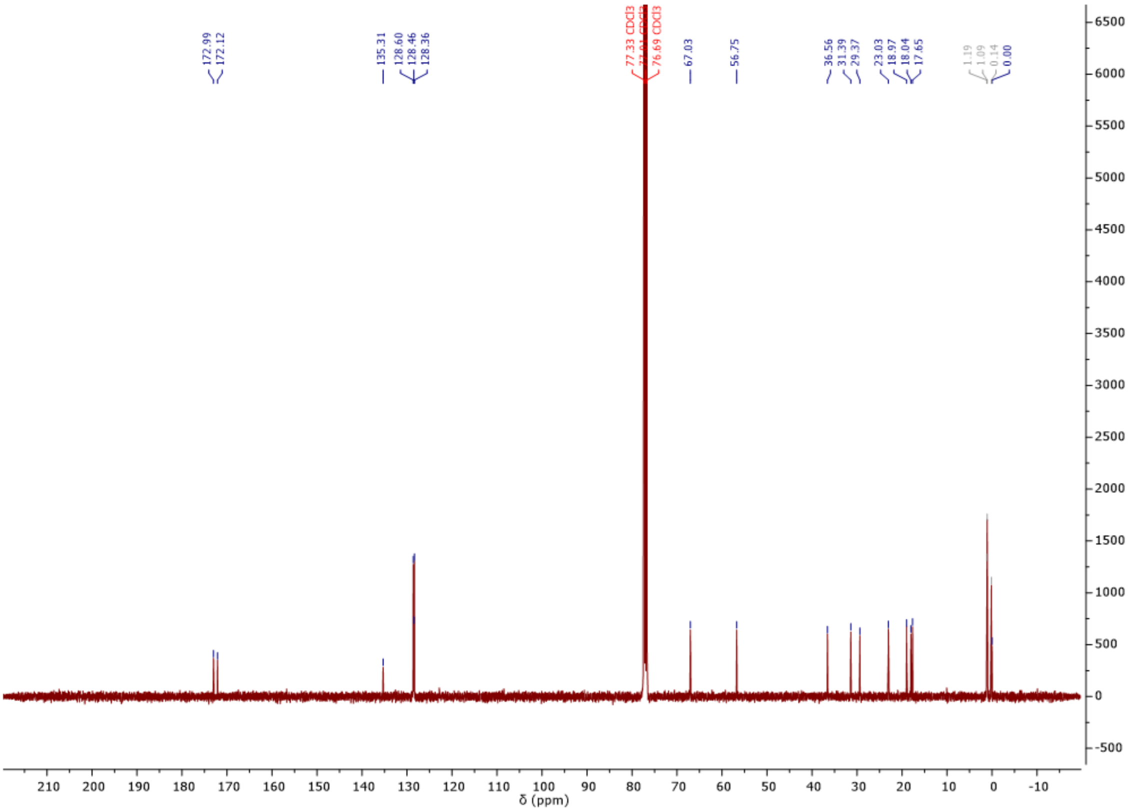

Figure S29: *^{13}C NMR (100 MHz, CDCl$_3$) spectrum of* ***BnO-Val-Si$_8$-Val-OBn****.*

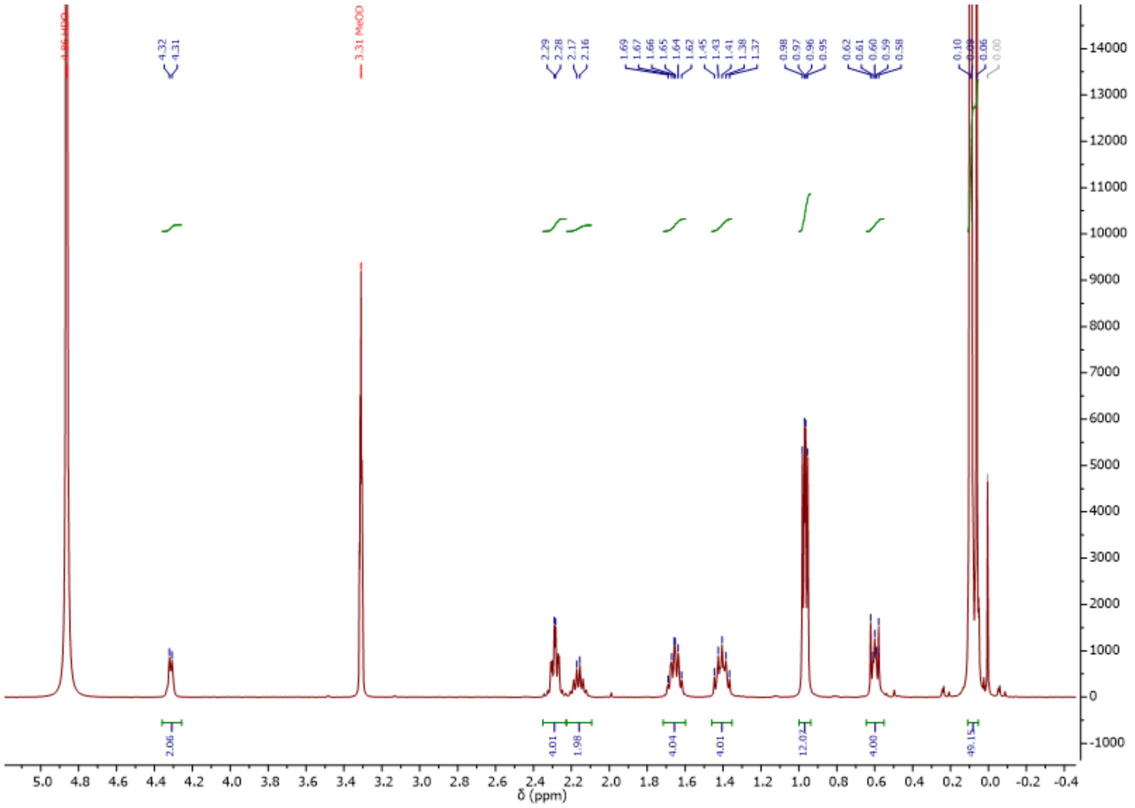

***Figure S30**: ^{1}H NMR (400 MHz, Methanol-d4) spectrum of **Val-Si8-Val**.*

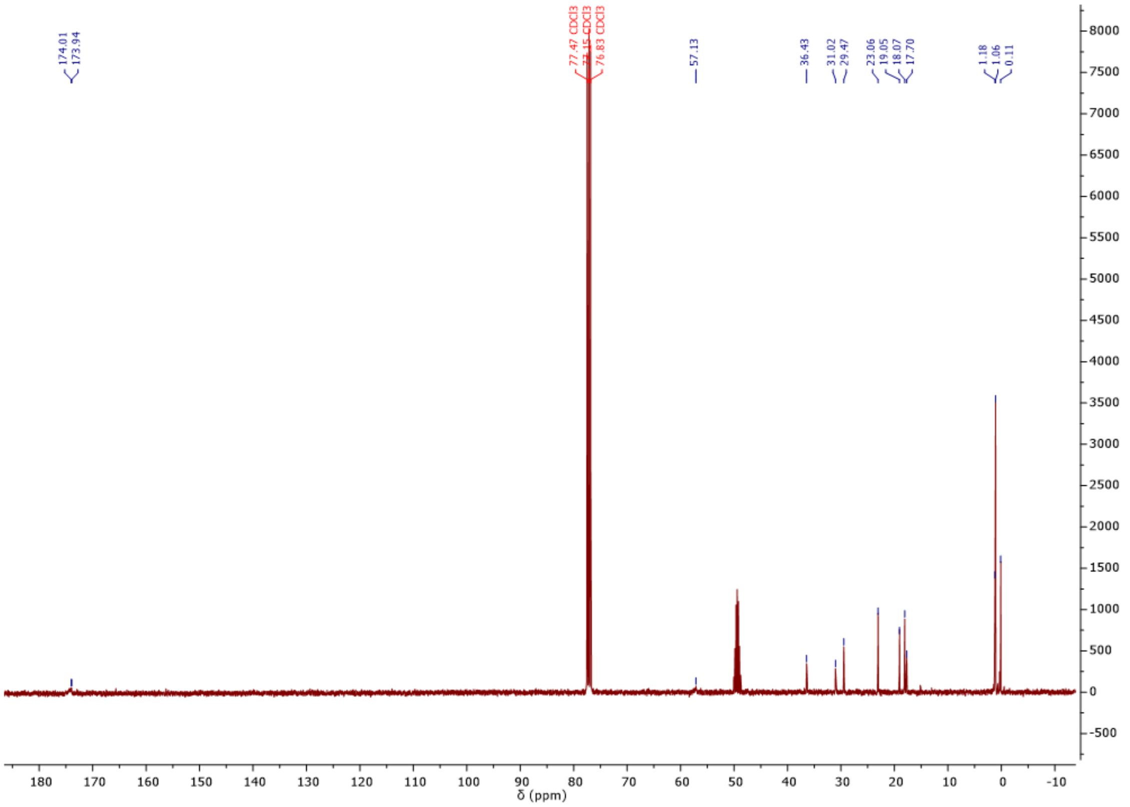

***Figure S31**: ^{13}C NMR (100 MHz, CDCl3) spectrum of **Val-Si8-Val**.*

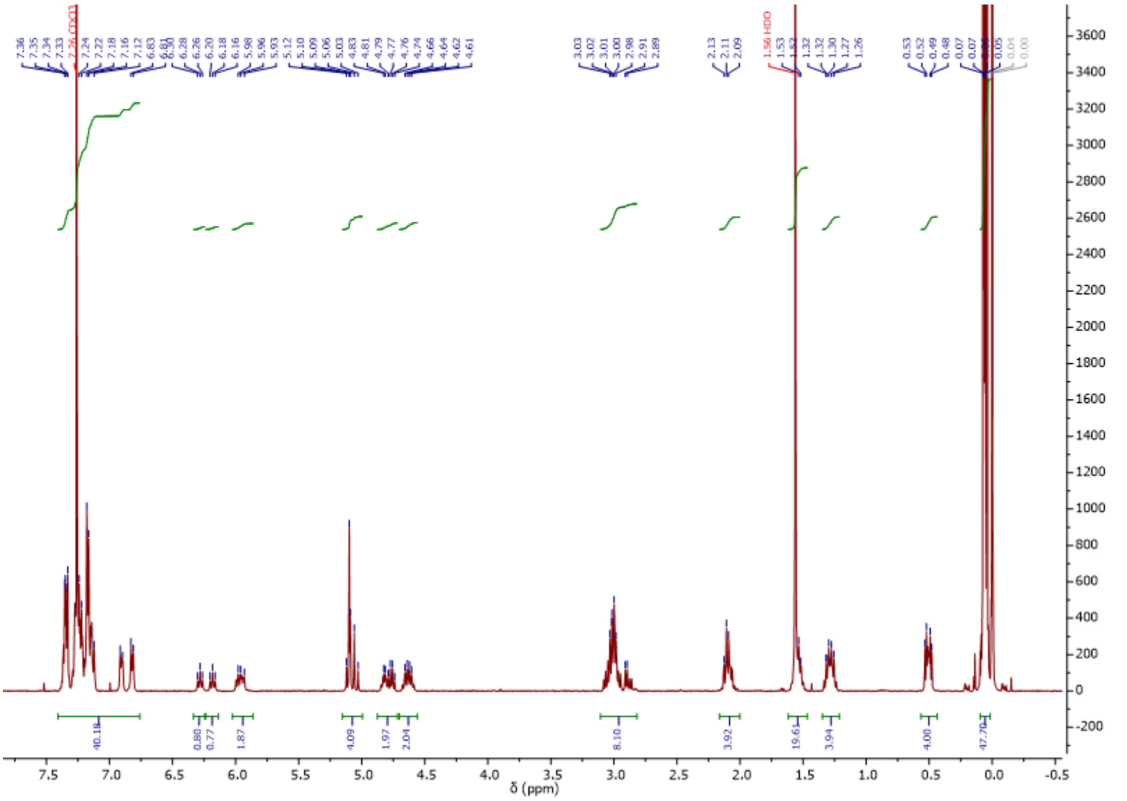

Figure S32: *^{1}H NMR (400 MHz, CDCl₃) spectrum of* ***BnO-Phe₂-Si₈-Phe₂-OBn***.

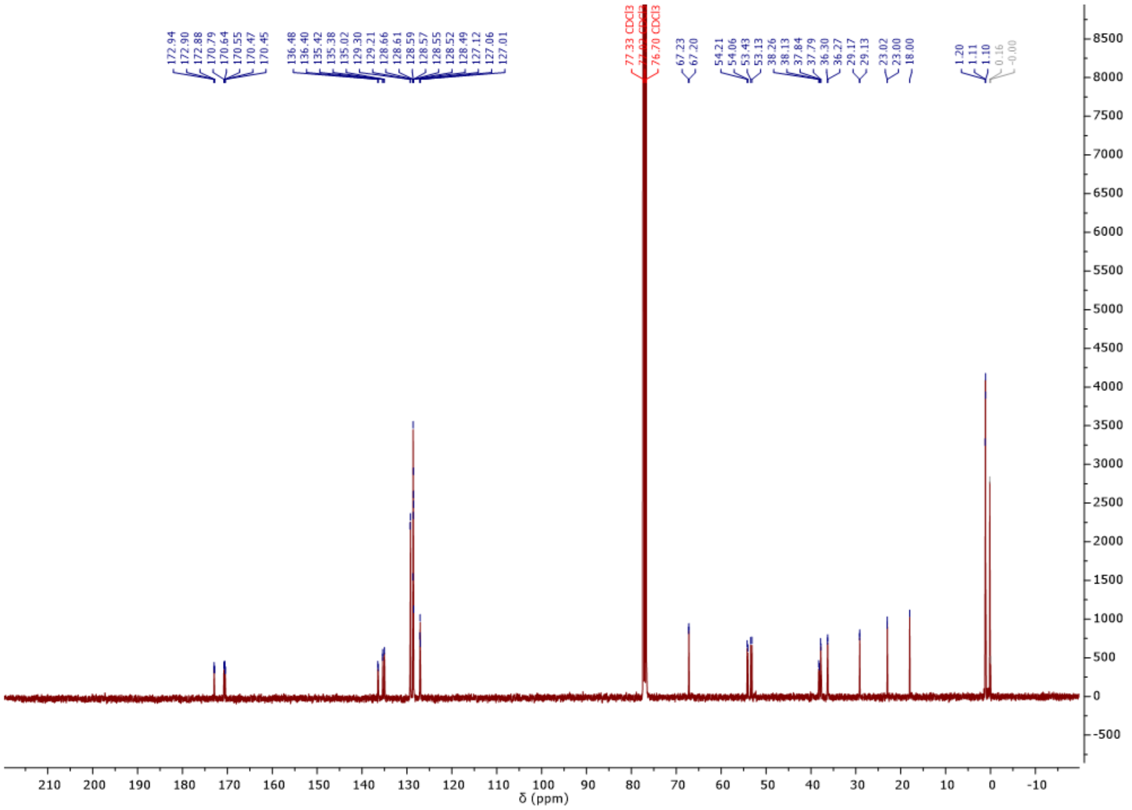

Figure S33: *^{13}C NMR (100 MHz, CDCl₃) spectrum of* ***BnO-Phe₂-Si₈-Phe₂-OBn***.

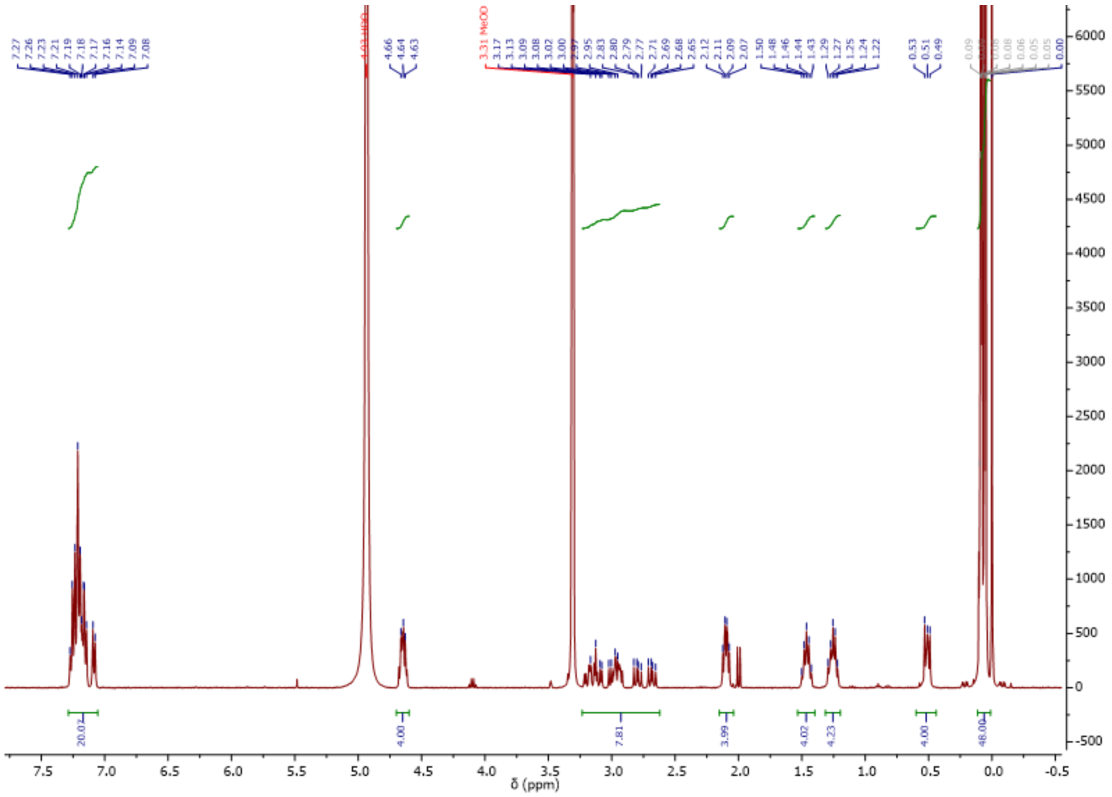

Figure S34: *^{1}H NMR (400 MHz, Methanol-d₄ + TFA) spectrum of* ***Phe₂-Si₈-Phe₂***.

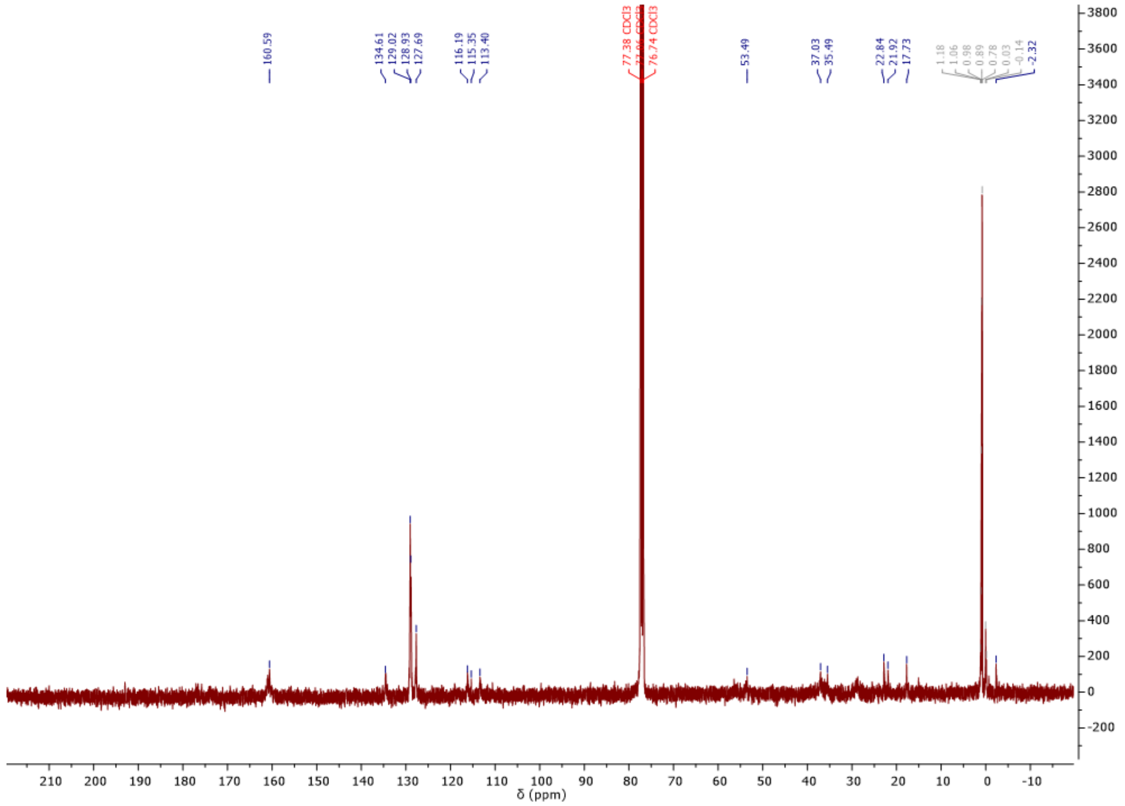

Figure S35: *^{13}C NMR (100 MHz, $CDCl_3$ + TFA) spectrum of* ***Phe₂-Si₈-Phe₂***.

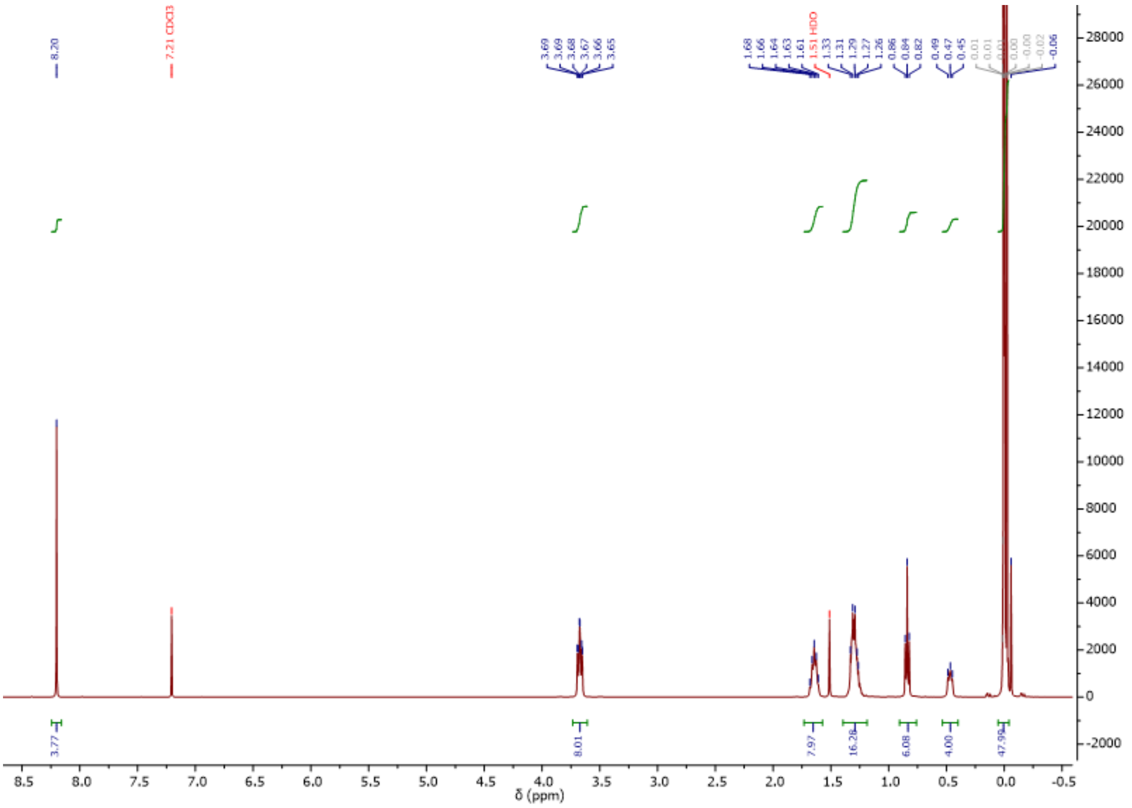

Figure S36: *^{1}H NMR (400 MHz, $CDCl_3$) spectrum of* ***PMDI-Si$_8$-PMDI***.

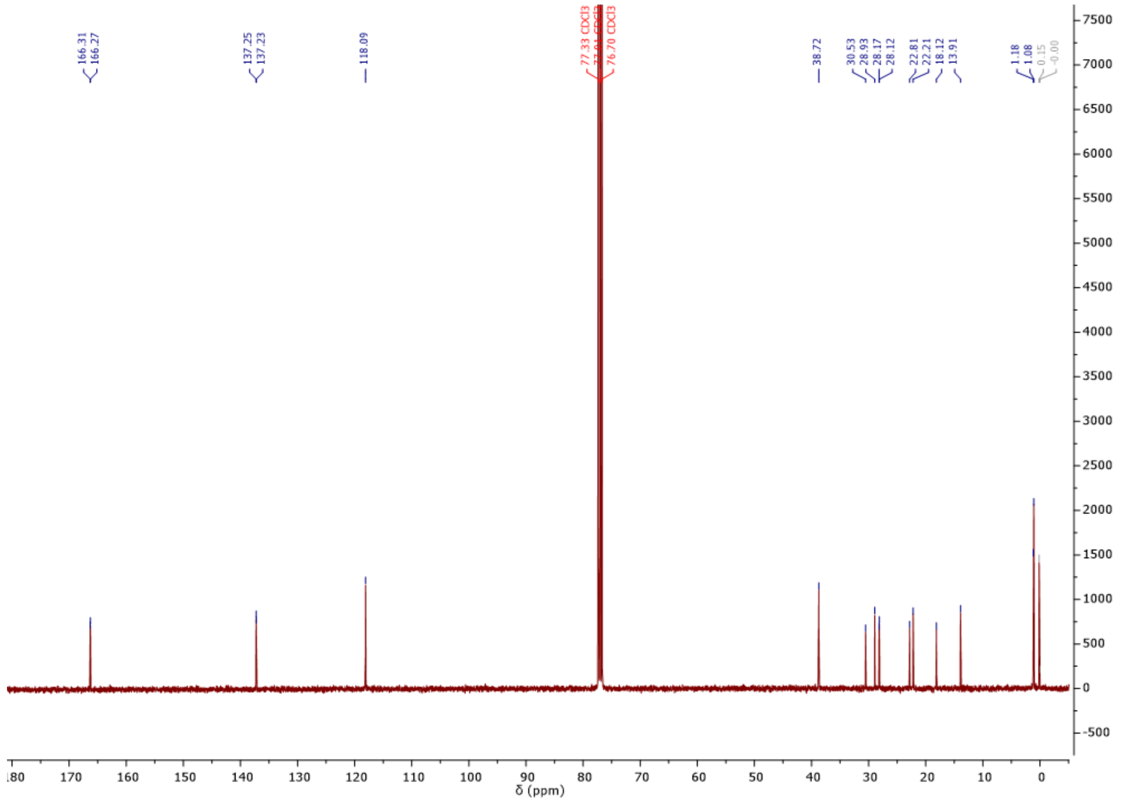

Figure S37: *^{13}C NMR (100 MHz, $CDCl_3$) spectrum of* ***PMDI-Si$_8$-PMDI***.

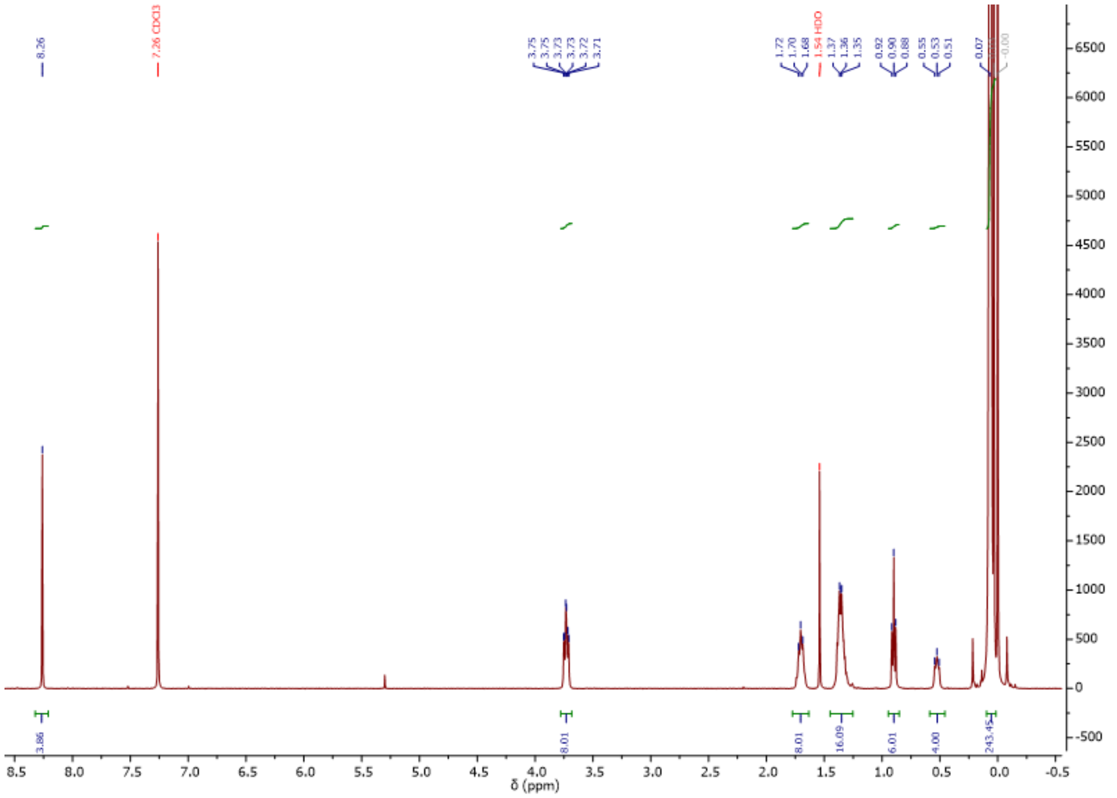

Figure S38: *^{1}H NMR (400 MHz, CDCl3) spectrum of* ***PMDI-Si40-PMDI****.*

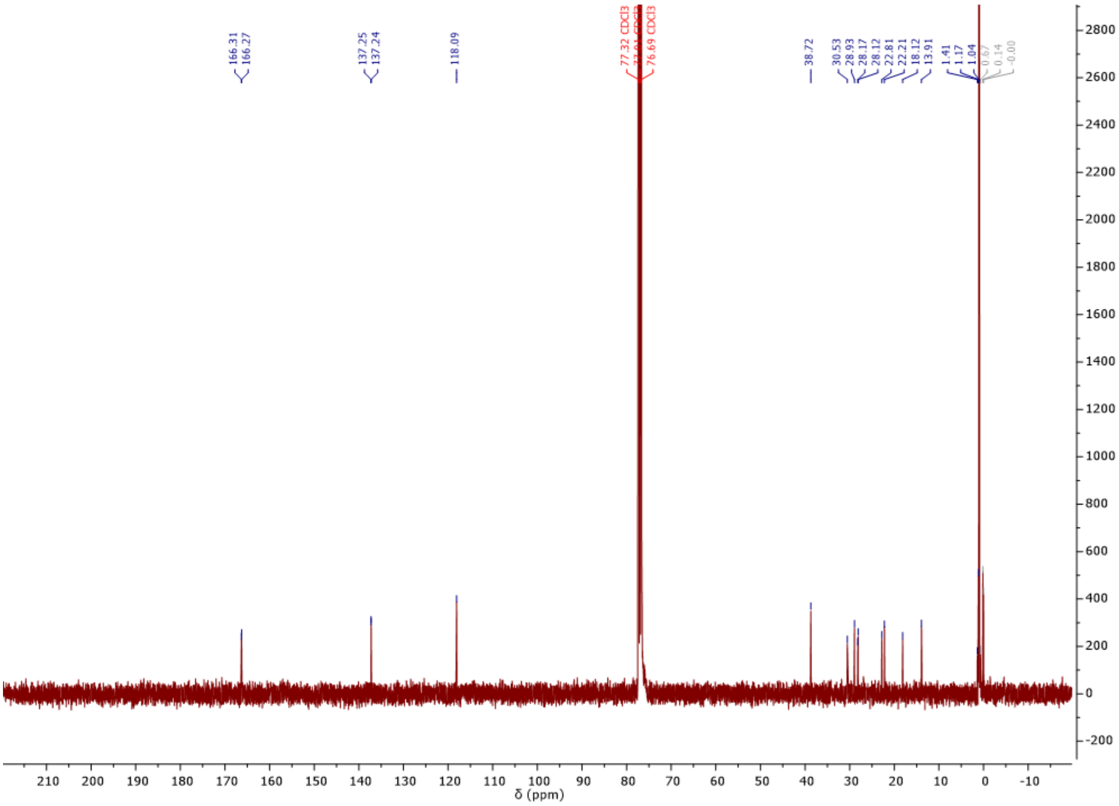

Figure S39: *^{13}C NMR (100 MHz, CDCl3) spectrum of* ***PMDI-Si40-PMDI****.*

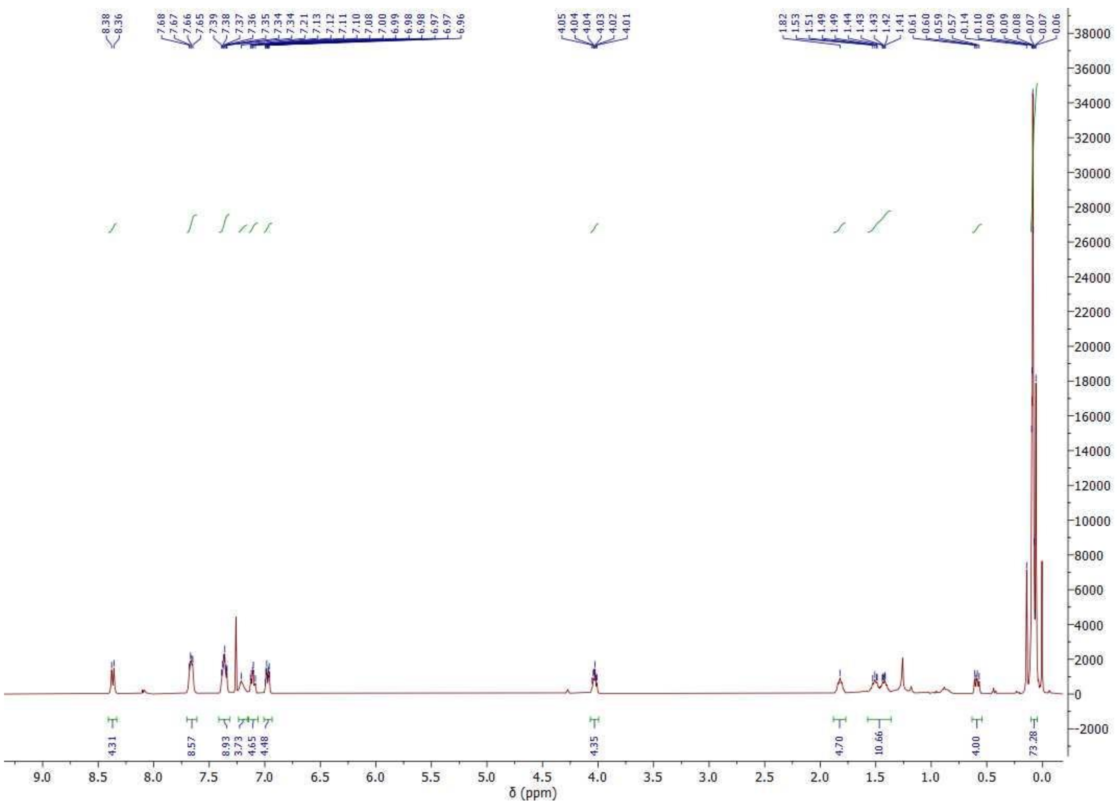

Figure S40: *^{1}H NMR (400 MHz, $CDCl_3$) spectrum of* ***Triazine-Si$_8$-Triazine****.*

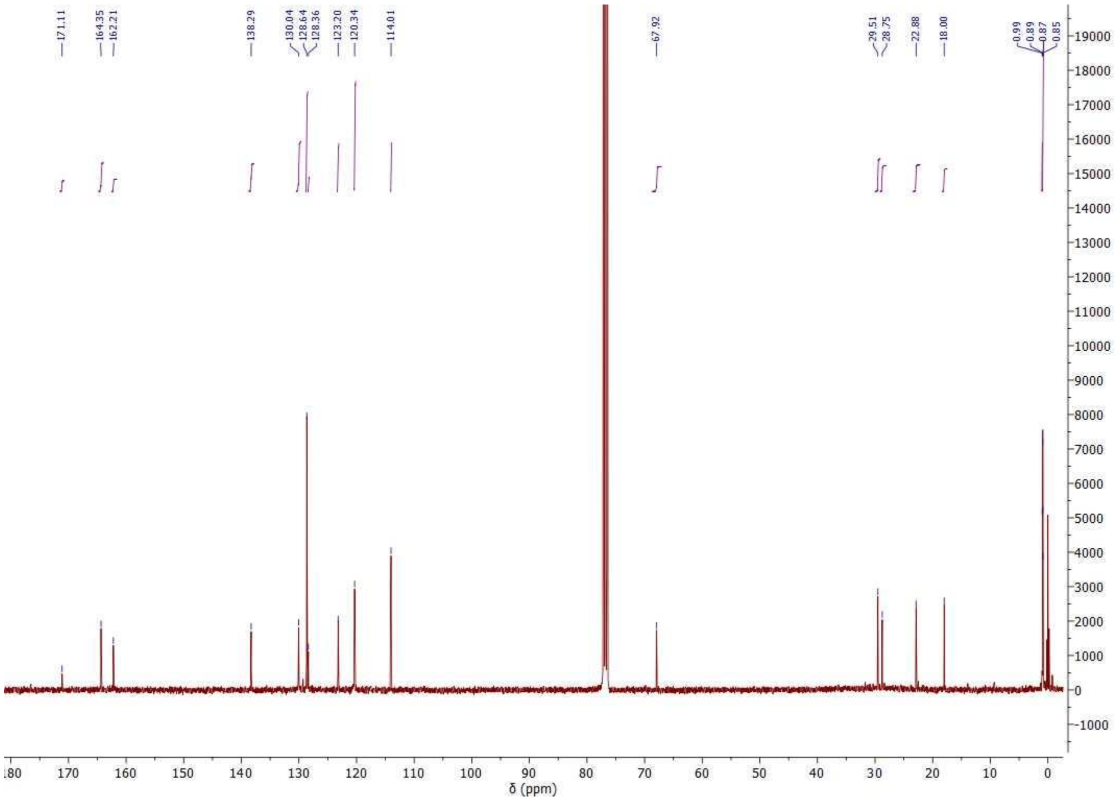

Figure S41: *^{13}C NMR (100 MHz, $CDCl_3$) spectrum of* ***Triazine-Si$_8$-Triazine****.*

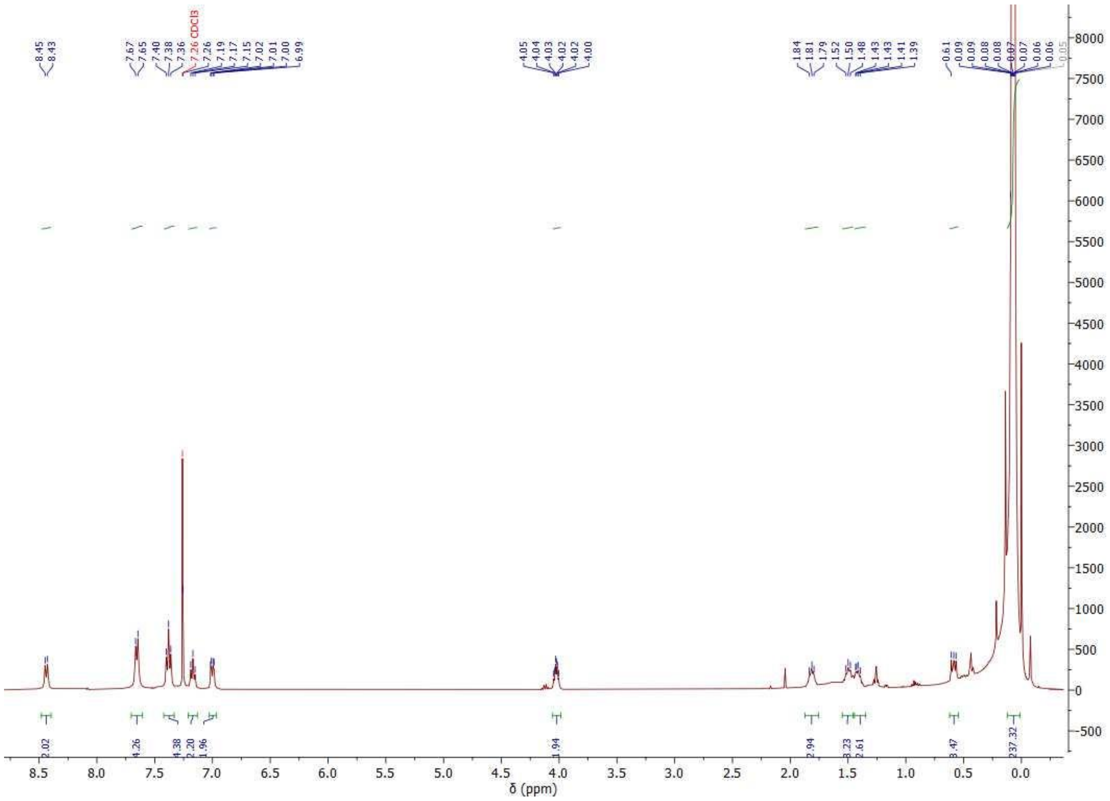

__Figure S42__: ^{1}H NMR (400 MHz, $CDCl_3$) spectrum of __Triazine-Si$_{40}$-Triazine__.

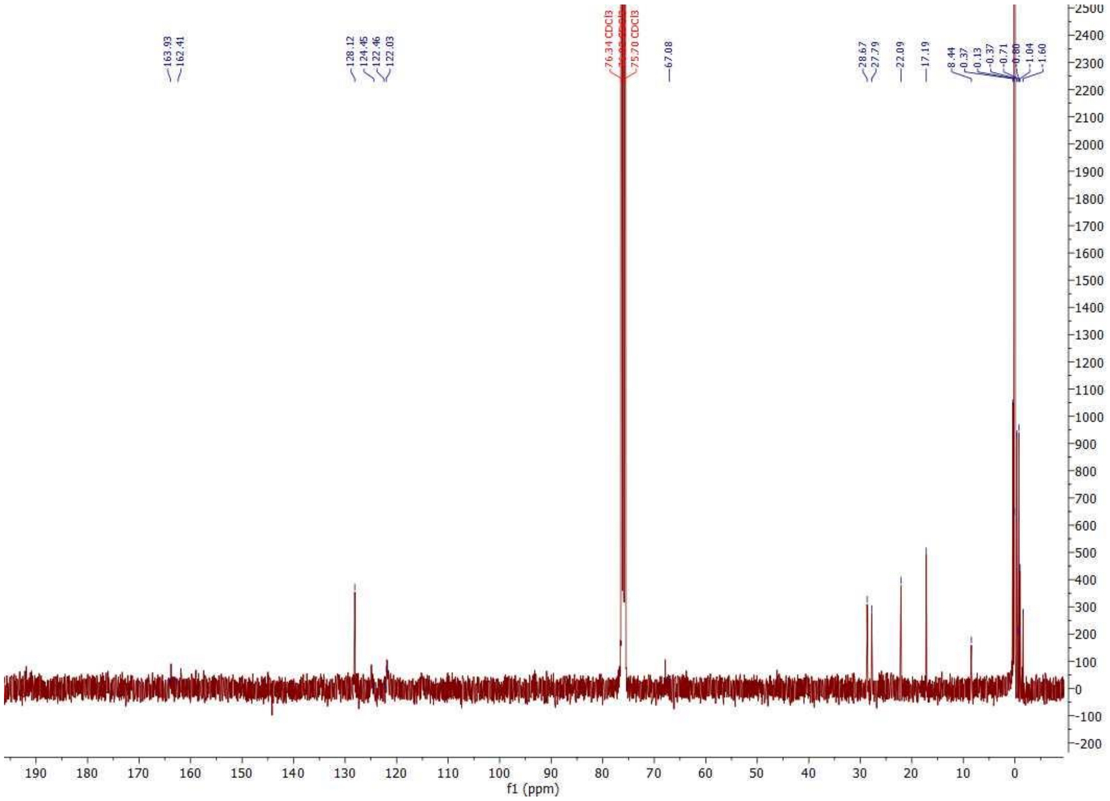

__Figure S43__: ^{13}C NMR (100 MHz, $CDCl_3$) spectrum of __Triazine-Si$_{40}$-Triazine__.

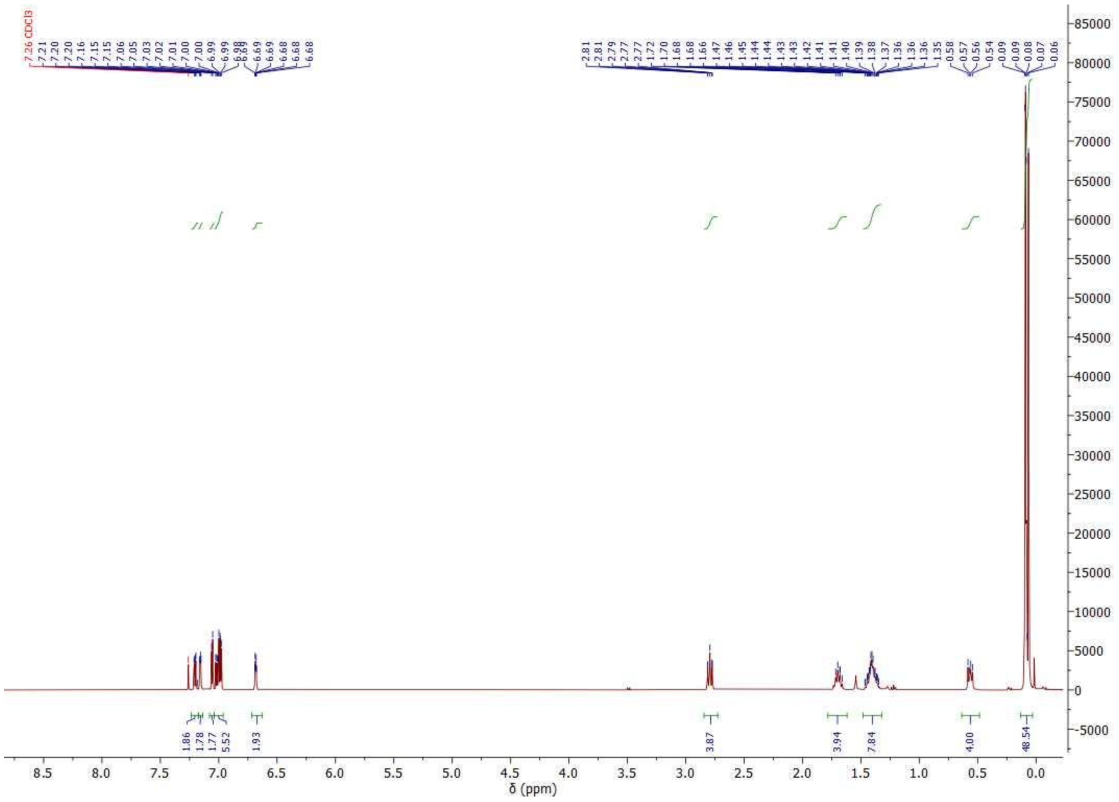

Figure S44: *^{1}H NMR (400 MHz, $CDCl_3$) spectrum of* ***Thiophene-Si₈-Thiophene****.*

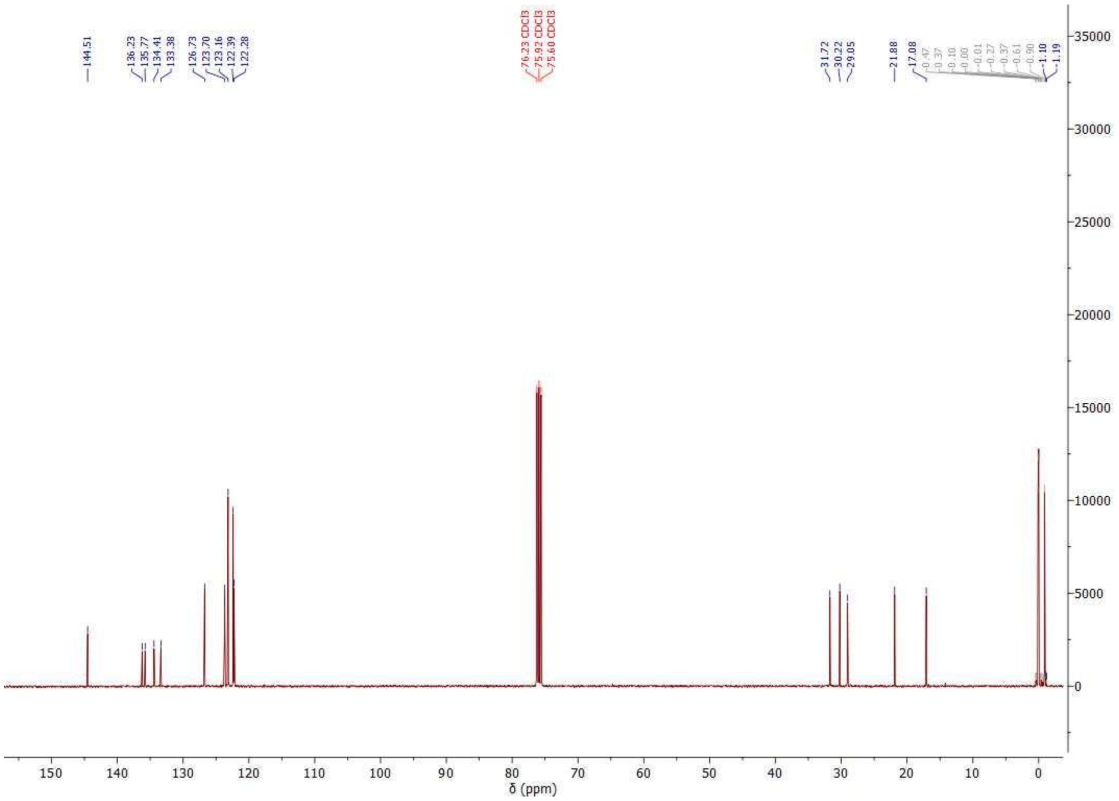

Figure S45: *^{13}C NMR (100 MHz, $CDCl_3$) spectrum of* ***Thiophene-Si₈-Thiophene****.*

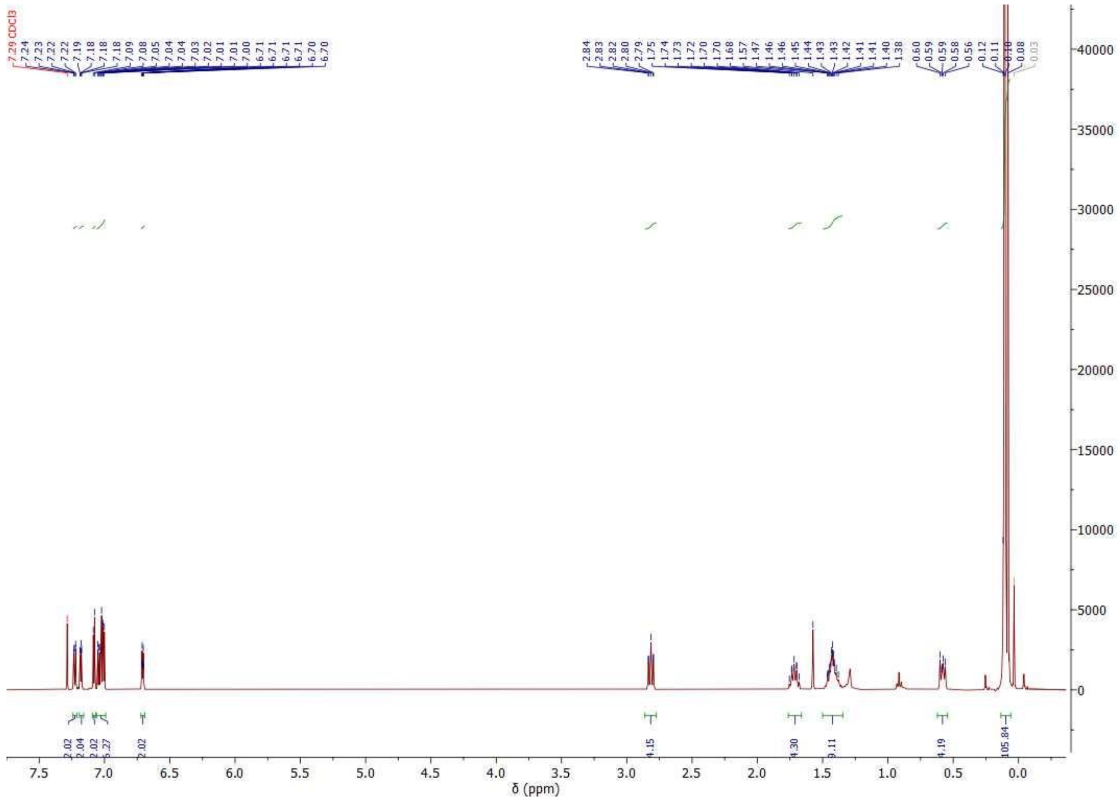

***Figure S46**: ^{1}H NMR (400 MHz, $CDCl_3$) spectrum of **Thiophene-Si_{16}-Thiophene**.*

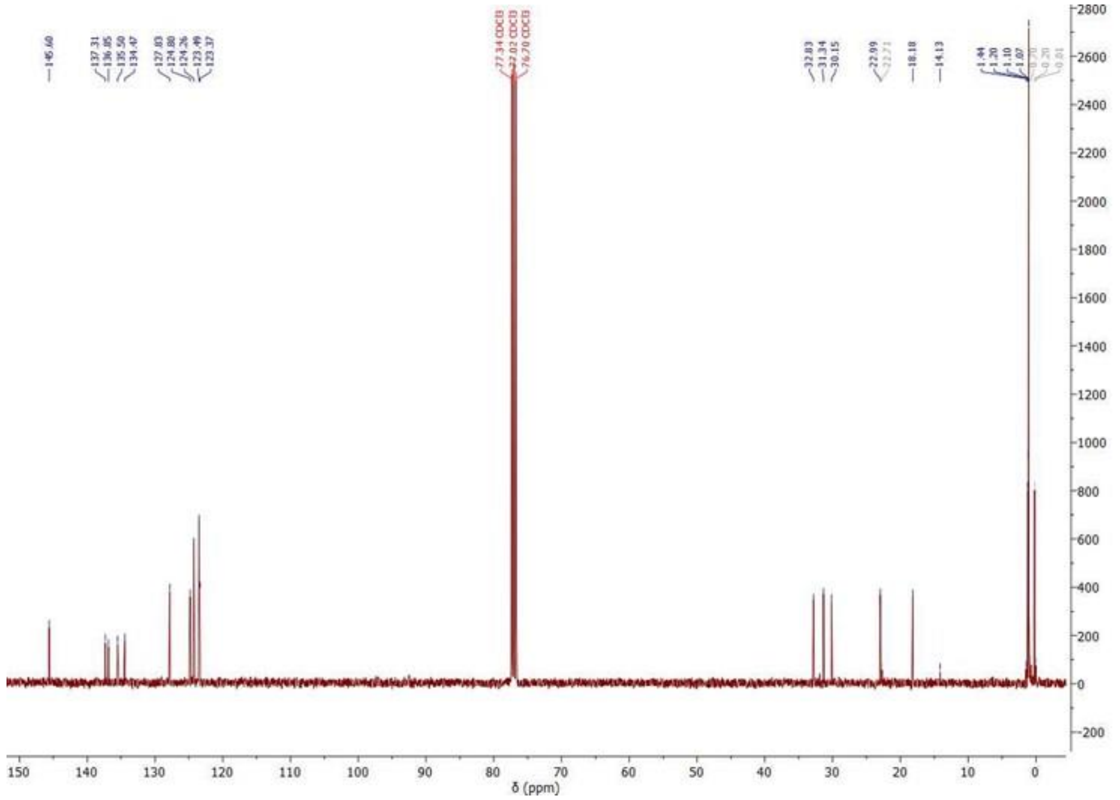

***Figure S47**: ^{13}C NMR (100 MHz, $CDCl_3$) spectrum of **Thiophene-Si_{16}-Thiophene**.*

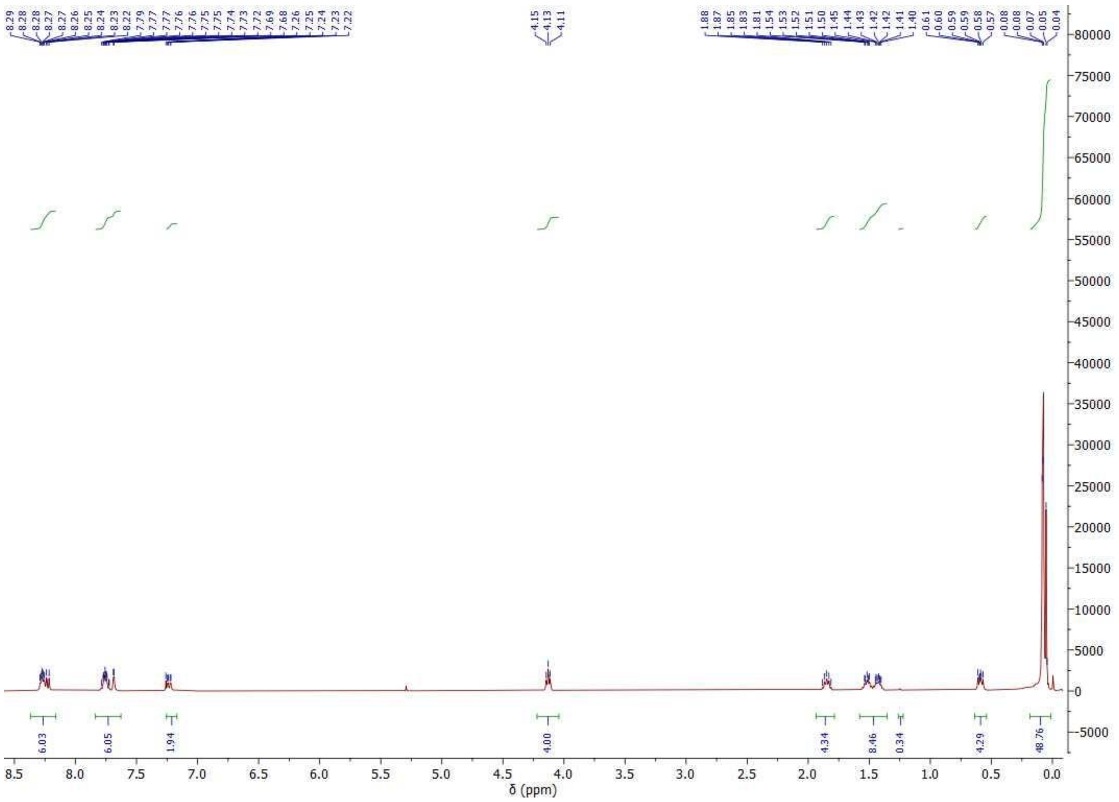

***Figure S48**: ^{1}H NMR (400 MHz, $CDCl_3$) spectrum of **H-AQ-Si$_8$-AQ-H**.*

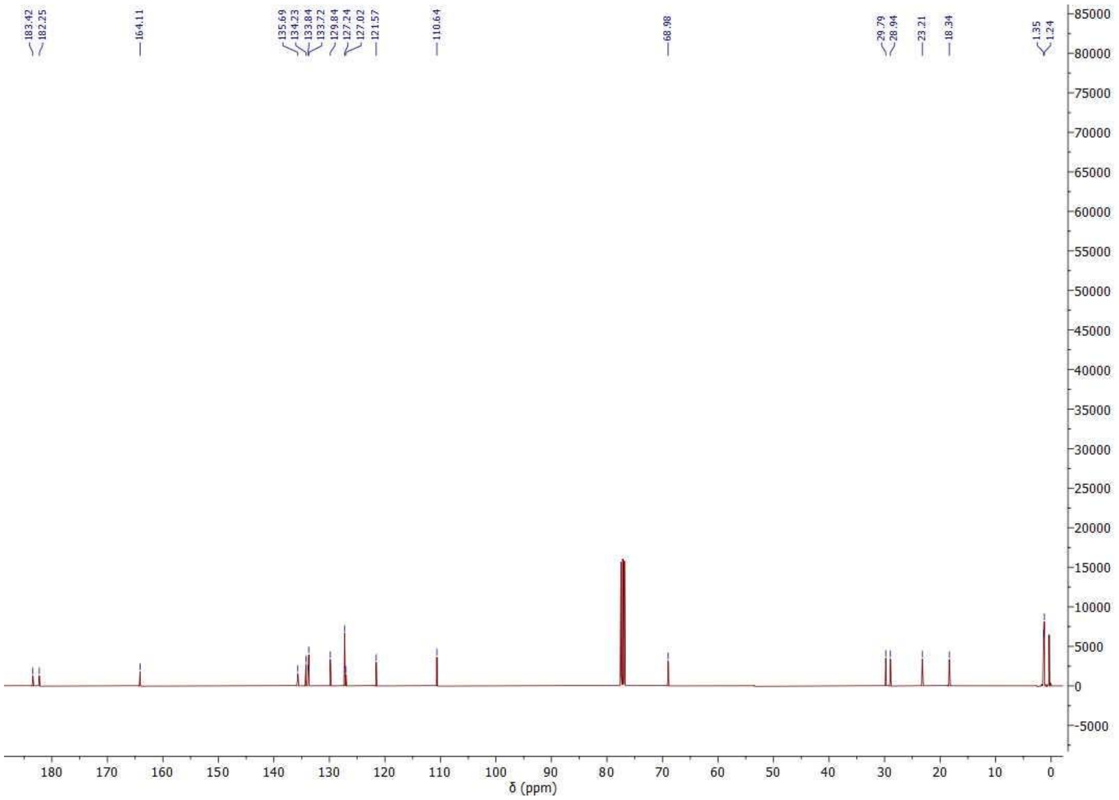

***Figure S49**: ^{13}C NMR (100 MHz, $CDCl_3$) spectrum of **H-AQ-Si$_8$-AQ-H**.*

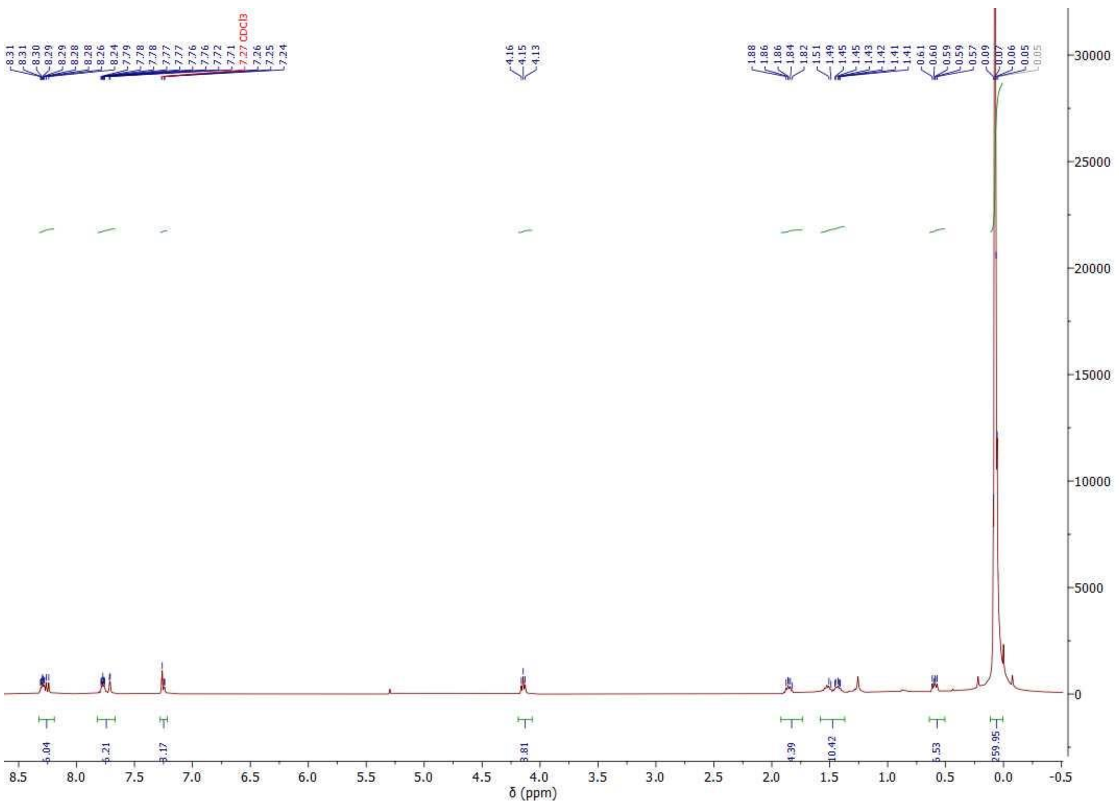

Figure S50: *^{1}H NMR (400 MHz, CDCl$_3$) spectrum of* ***H-AQ-Si$_{40}$-AQ-H***.

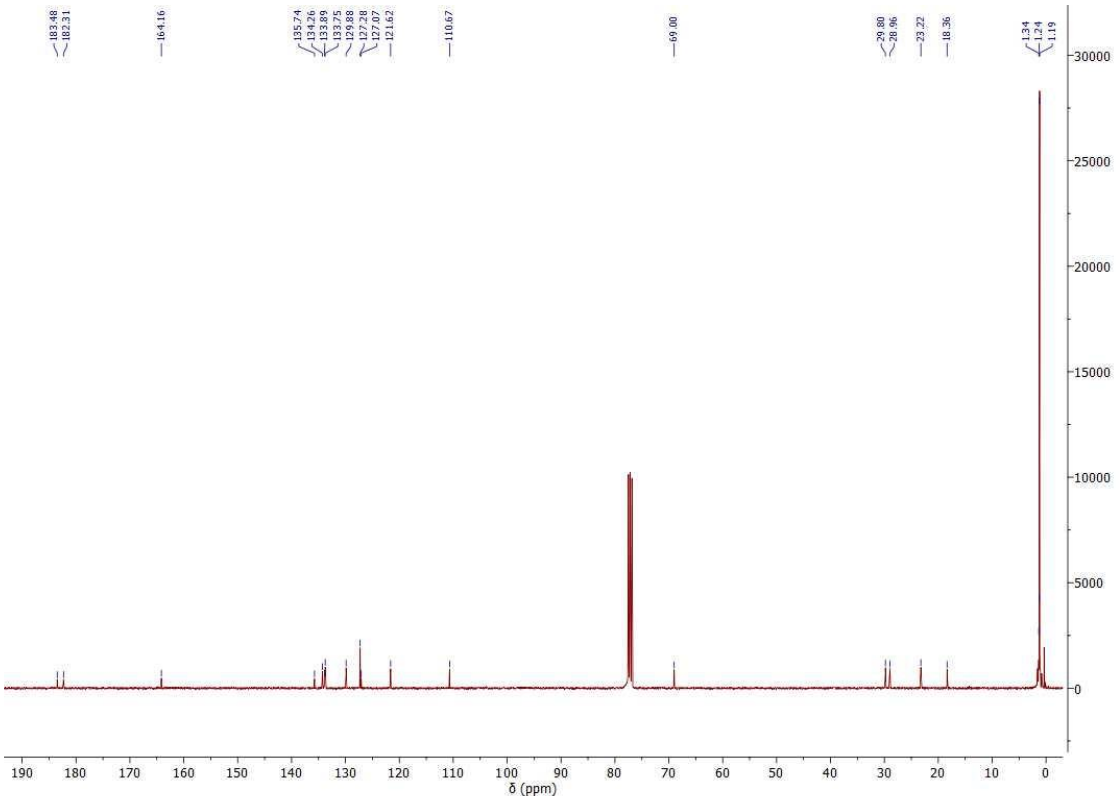

Figure S51: *^{13}C NMR (100 MHz, CDCl$_3$) spectrum of* ***H-AQ-Si$_{40}$-AQ-H***.

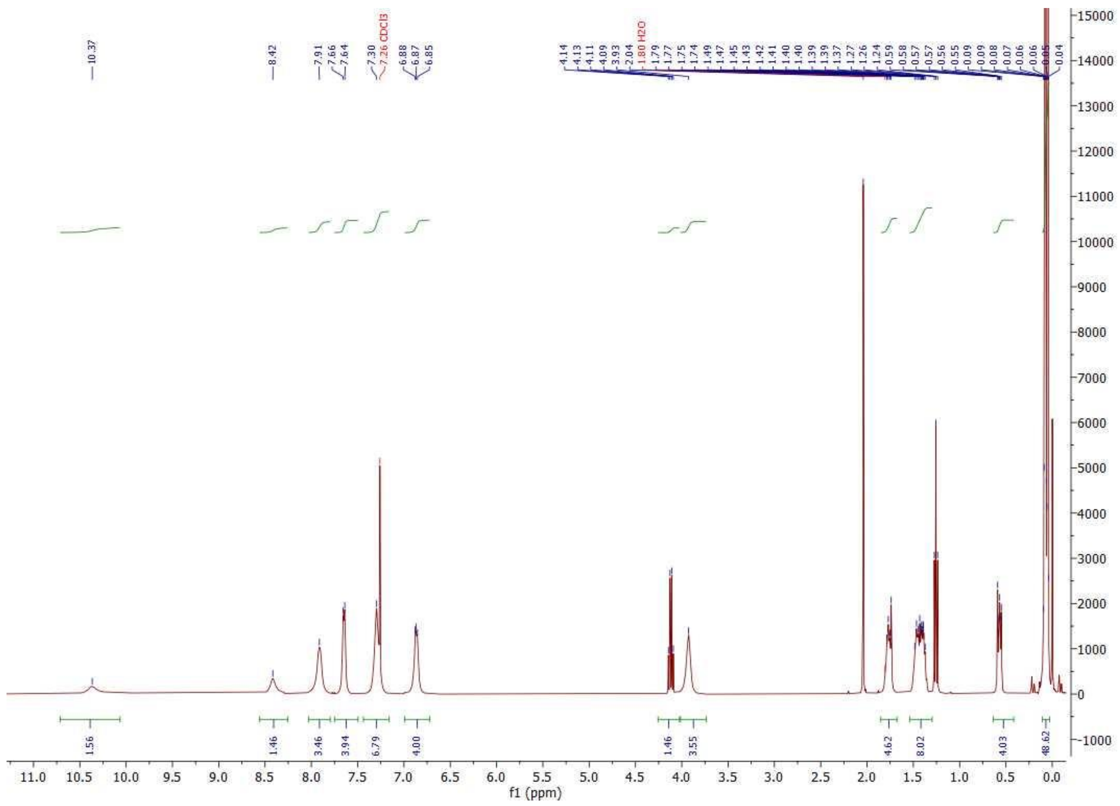

Figure S52: *^{1}H NMR (400 MHz, $CDCl_3$) spectrum of* ***Acylhydr-Si$_8$-Acylhydr***.

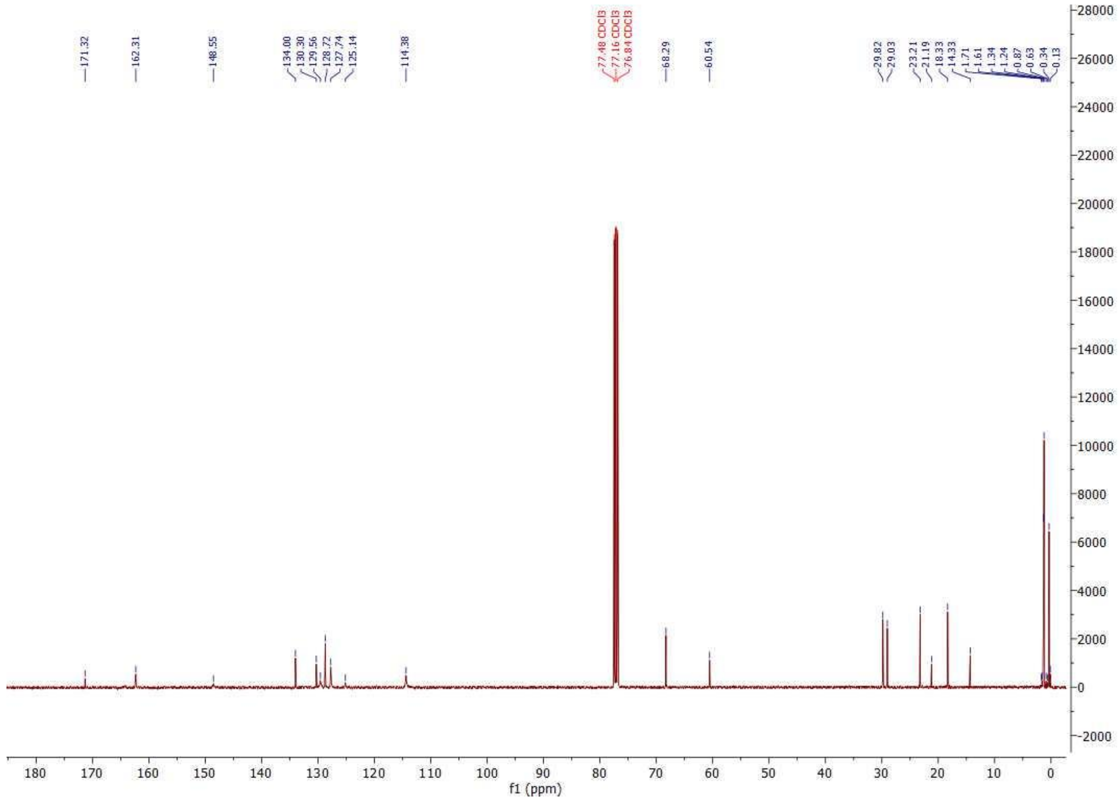

Figure S53: *^{13}C NMR (100 MHz, $CDCl_3$) spectrum of* ***Acylhydr-Si$_8$-Acylhydr***.

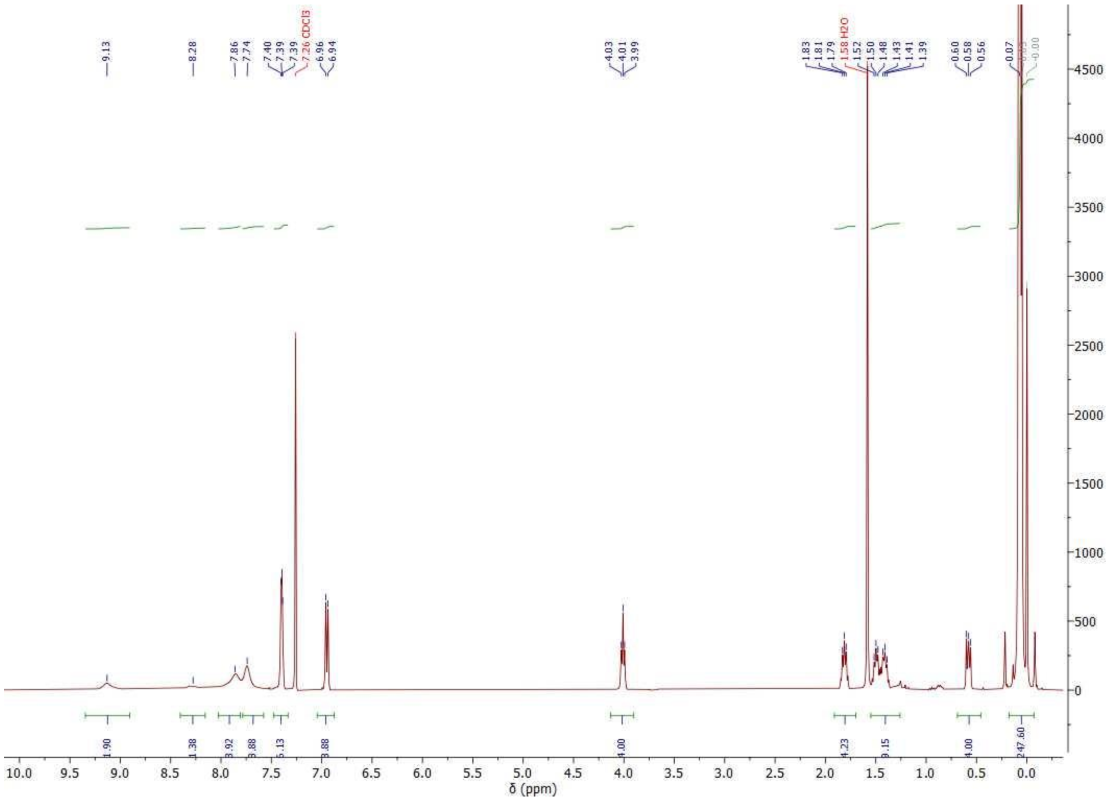

Figure S54: *^{1}H NMR (400 MHz, CDCl$_3$) spectrum of* ***Acylhydr-Si$_{40}$-Acylhydr***.

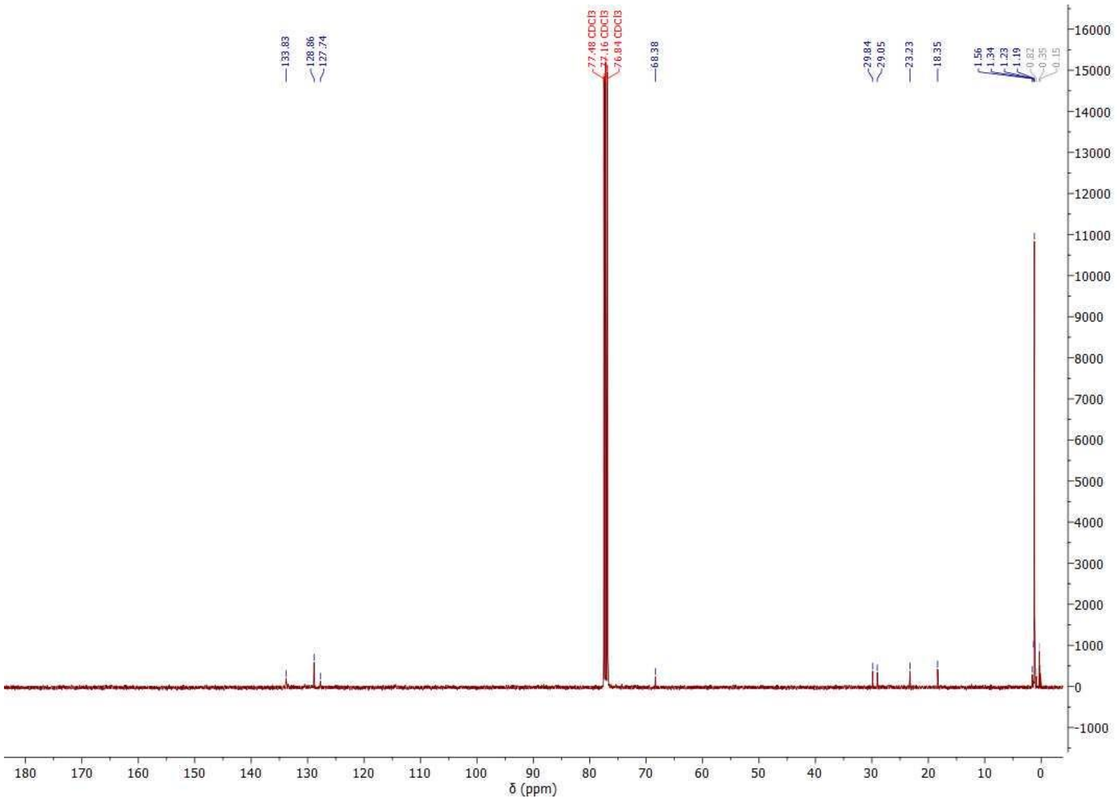

Figure S55: *^{13}C NMR (100 MHz, CDCl$_3$) spectrum of* ***Acylhydr-Si$_{40}$-Acylhydr***.

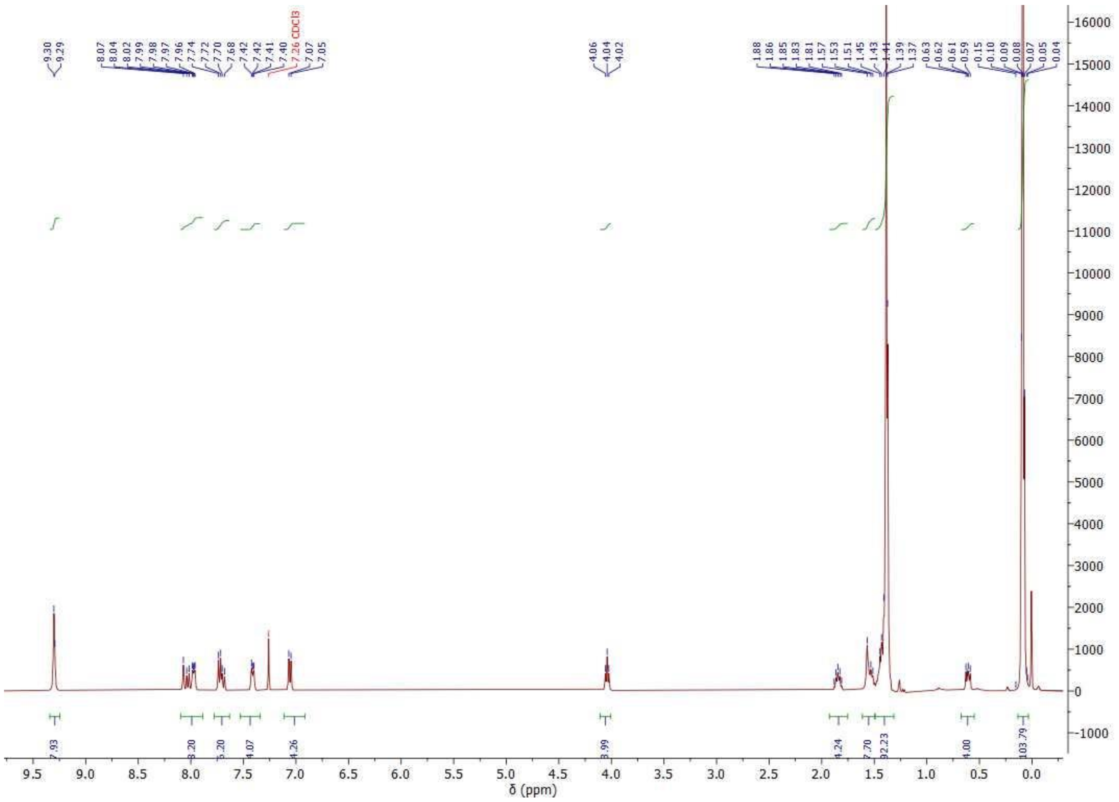

Figure S56: *^{1}H NMR (400 MHz, CDCl₃) spectrum of* ***TIPSPent-Si₁₆-TIPSPent***.

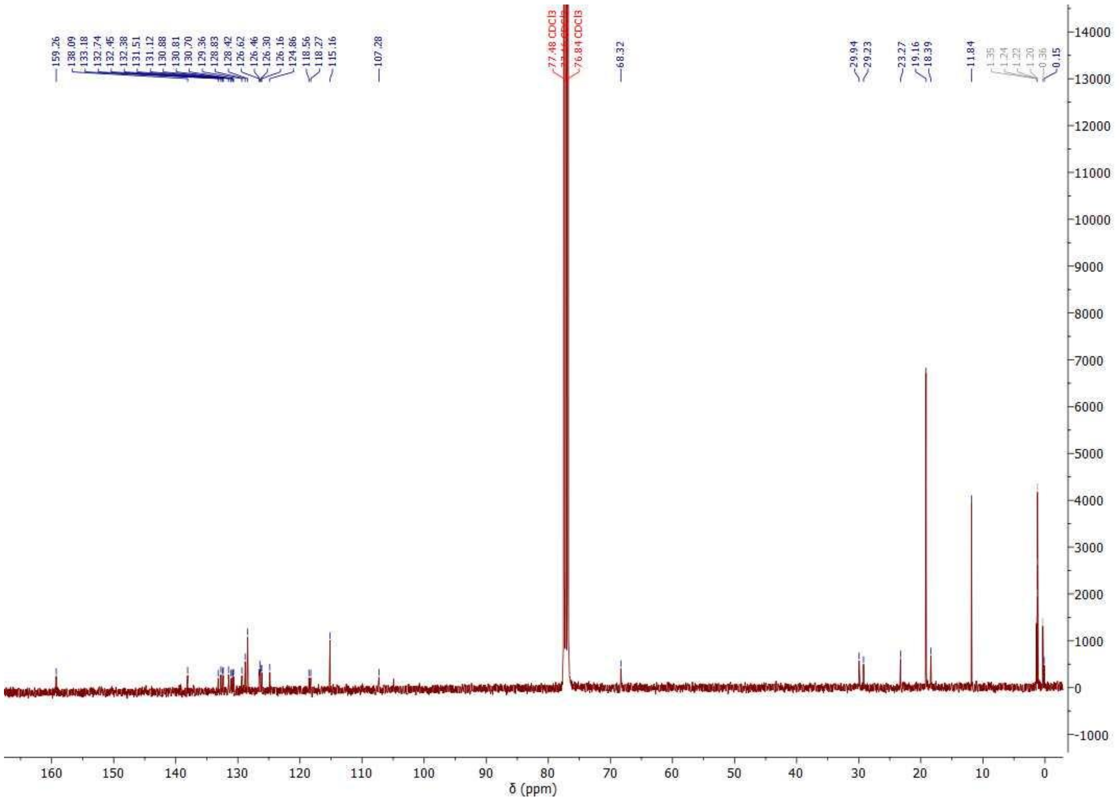

Figure S57: *^{13}C NMR (100 MHz, CDCl₃) spectrum of* ***TIPSPent-Si₁₆-TIPSPent***.

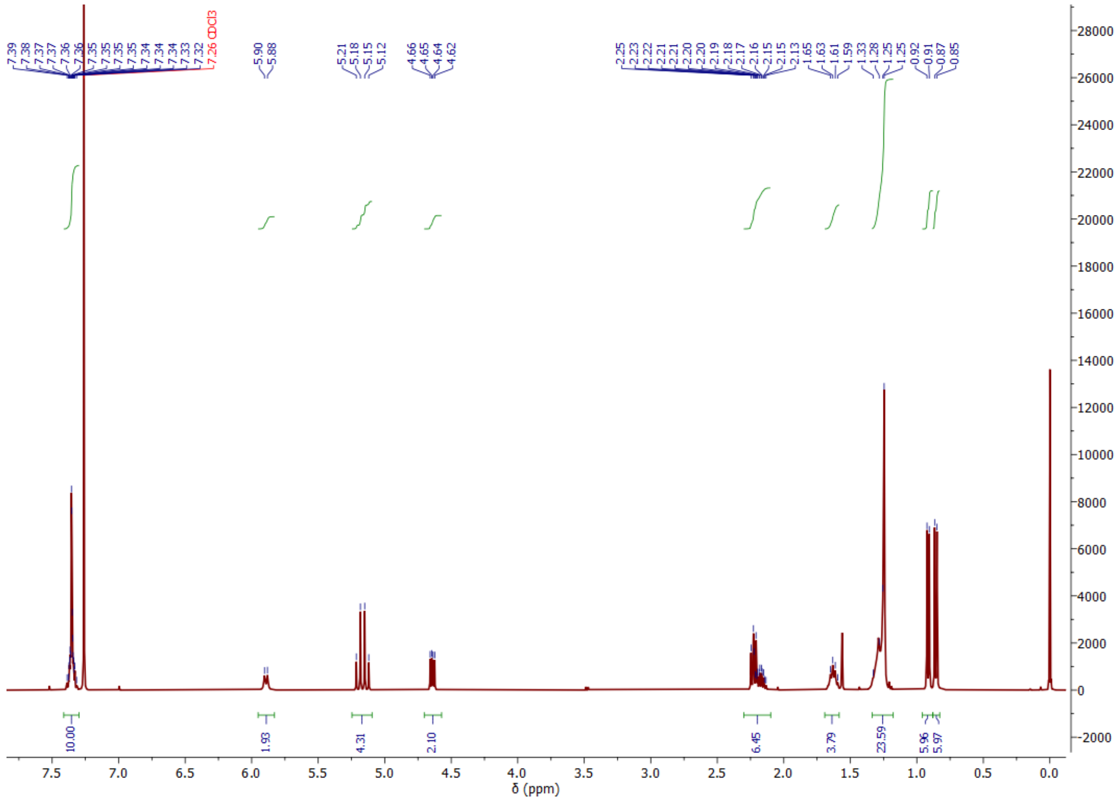

Figure S58: ^{1}H NMR (400 MHz, $CDCl_3$) spectrum of ***BnO-Val-C$_{18}$-Val-OBn***.

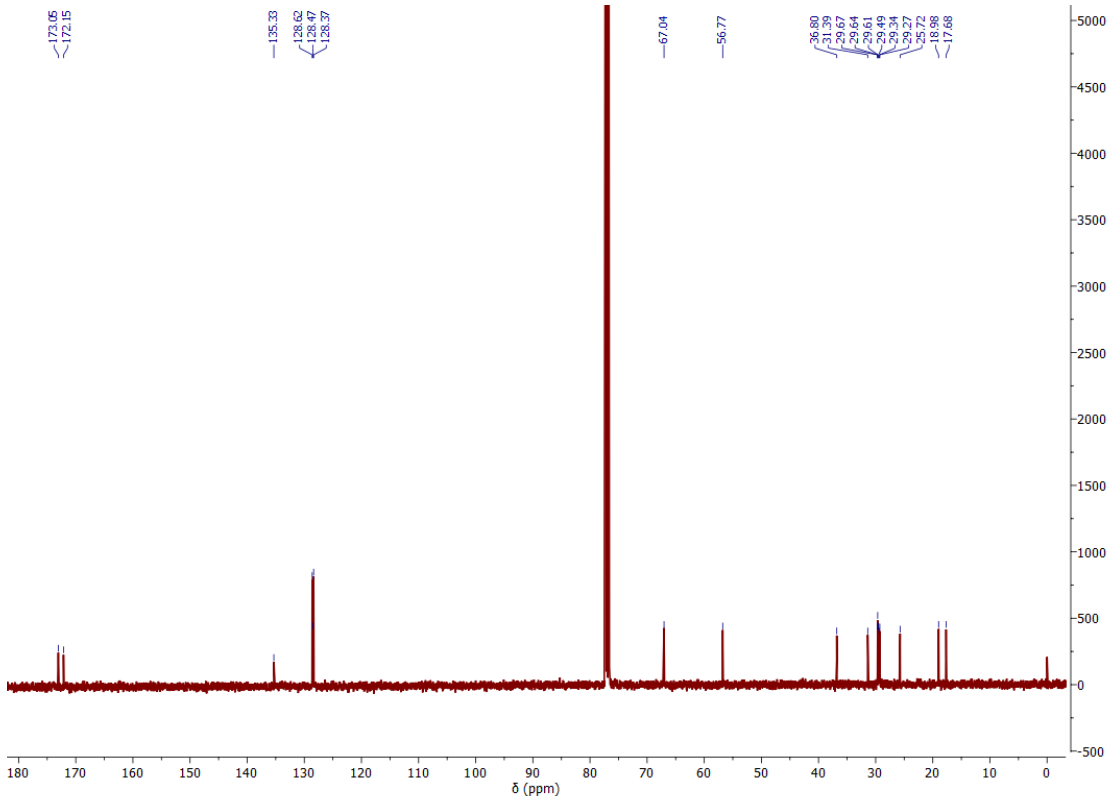

Figure S59: ^{13}C NMR (100 MHz, $CDCl_3$) spectrum of ***BnO-Val-C$_{18}$-Val-OBn***.

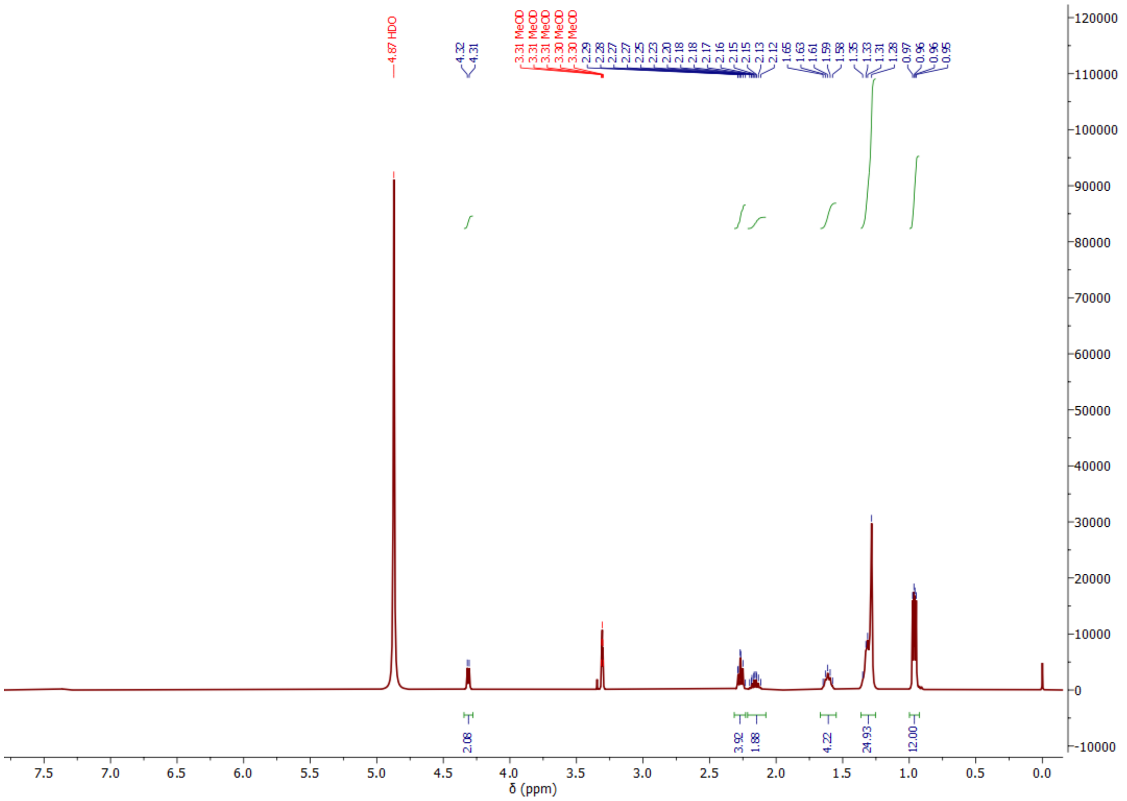

Figure S60: *^{1}H NMR (400 MHz, Methanol-d4) spectrum of* ***Val-C18-Val****.*

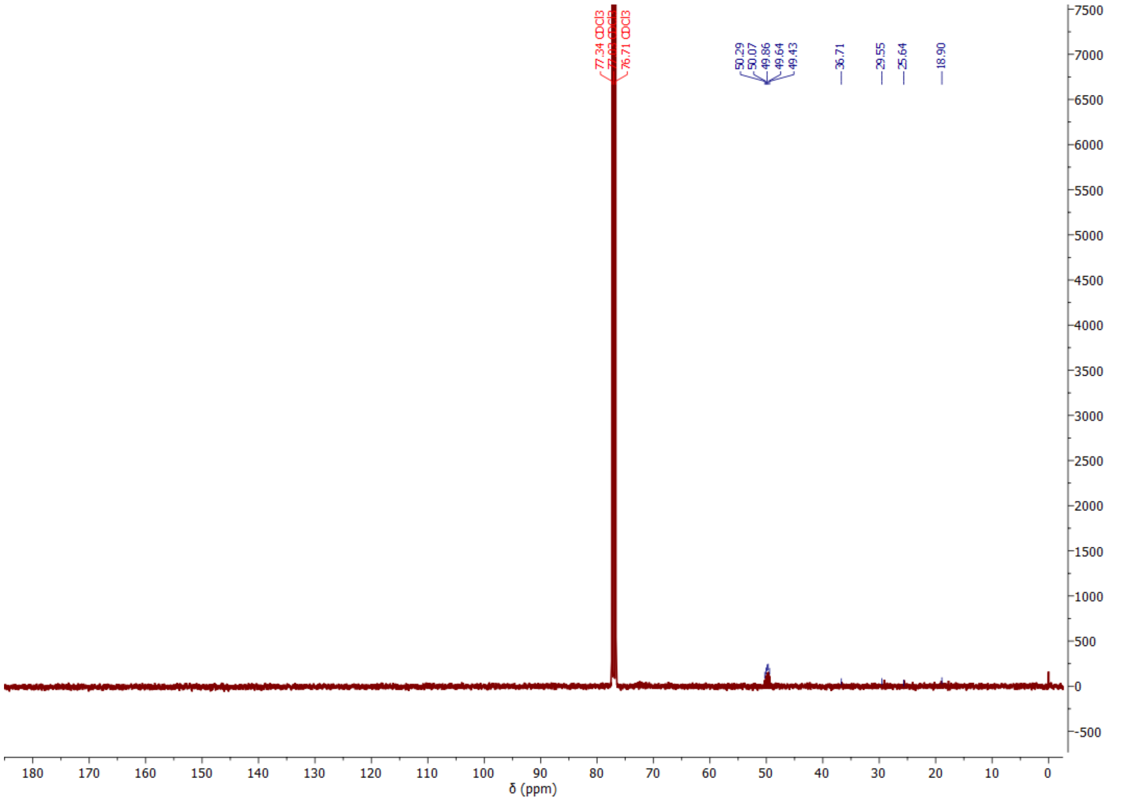

Figure S61: *^{13}C NMR (100 MHz, $CDCl_3$) spectrum of* ***Val-C18-Val****.*

5. Crystal Lattice Analysis

General Procedure & Remarks

Crystal structures were used without further refinement from the CCDC. The respective codes are given in Table 1, Table S1 and Table S2. Structures that are not reported in the CCDC were obtained by crystallization of the respective crystalline unit followed by X-ray Crystal Lattice Analysis.

Layer spacings (d_{layer}) were determined using the program Mercury 2023.1.0. First, a representative unit of the crystal lattice was calculated using the "Packing/Slicing" function (typically 3x3x3). If a layer could be identified, two planes were calculated on each site of the crystalline unit. The planes were calculated using the "Planes…" function based on a set of three atoms at the crystalline unit periphery. The distance of the two parallel planes (= d_{layer}) was determined using the "Measure distance" function.

Energy networks were calculated using the program CrystalExplorer 21.3. Molecules within the lattice were generated around the central molecule using the "Generate Atoms Within Radius" followed by the "Complete Fragments" function. Selection of the central molecule followed by "Calculate Energies" (Accurate, B3LYP/6-31G(d,p)) afforded the energy network. The network was visualized. The thickness of the interaction columns is proportional to the respective attractive energy. This allows straight forward comparison with the layer obtained in the program Mercury and judgement if the strongest interactions in the lattice match the previously identified layer. The thermal stability is determined via E_{tot}, which is the sum of interaction energies between the central molecule and its neighbors within the layer. Interactions to molecules outside the layer are no considered.

Distances between connecting points are determined using the program Mercury 2023.1.0. and the "Measure distance" function. The connection points are selected such that the siloxane chain proceeds alternatingly at both sides of the layer.

2-HOMeAzo (EXEZUC)

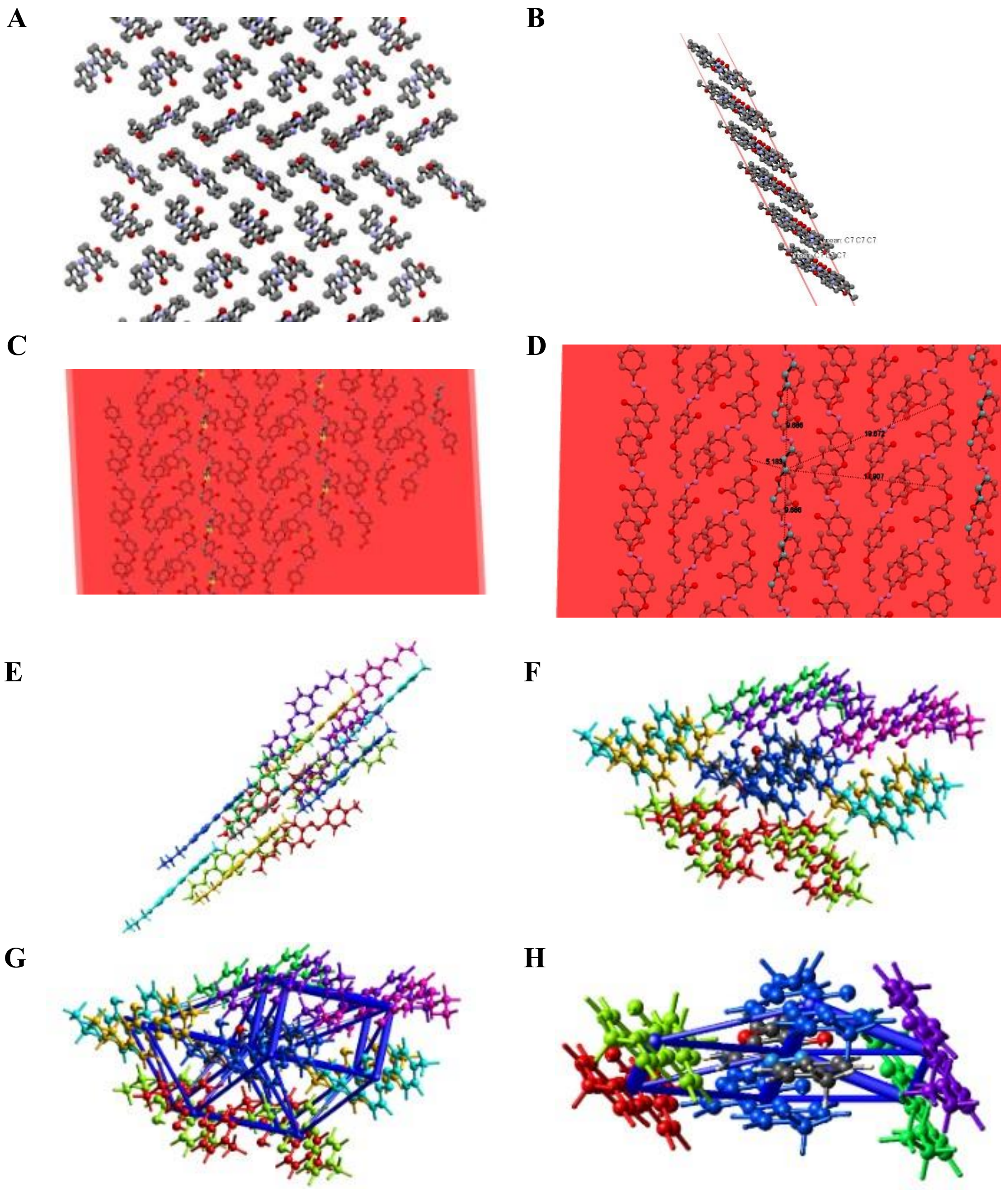

I

	N	Symop	R	Electron Density	E_ele	E_pol	E_dis	E_rep	E_tot
	2	-x+1/2, y, z+1/2	6.45	B3LYP/6-31G(d,p)	-2.7	-0.6	-21.9	11.7	-15.1
	2	x, y+1/2, z+1/2	9.69	B3LYP/6-31G(d,p)	-9.0	-1.7	-27.0	19.1	-22.5
	2	-x+1/2, y+1/2, z	10.39	B3LYP/6-31G(d,p)	-6.7	-1.0	-24.3	18.0	-17.9
	1	-x, -y, z	5.31	B3LYP/6-31G(d,p)	-11.2	-4.3	-44.7	33.2	-33.5
	2	x, y, z	17.90	B3LYP/6-31G(d,p)	0.1	-0.1	-4.4	1.8	-2.7
	2	x, y+1/2, z+1/2	9.69	B3LYP/6-31G(d,p)	-10.9	-1.5	-52.5	33.6	-37.6
	2	-x, -y+1/2, z+1/2	10.75	B3LYP/6-31G(d,p)	-4.0	-0.8	-21.7	10.5	-17.2
	1	-x, -y, z	18.32	B3LYP/6-31G(d,p)	-0.2	-0.0	-5.2	2.7	-3.1

Figure S62*: Crystal Lattice Analysis of the crystalline unit 2-OHMeAzo (CCDC identifier: EXEZUC). (A,B) Identification of the layer. (C,D) Looking for ordering along two axes and measuring distance between connection points which was found to be 9.7 Å and thus sufficiently large. (E-I) Energy calculations using B3LYP/6-31G(d,p) showing the overlap with the layers and the total attractive energy between neighbors which was found to be −158.9 kJ/mol.*

AcylHydr (RUJQOD)

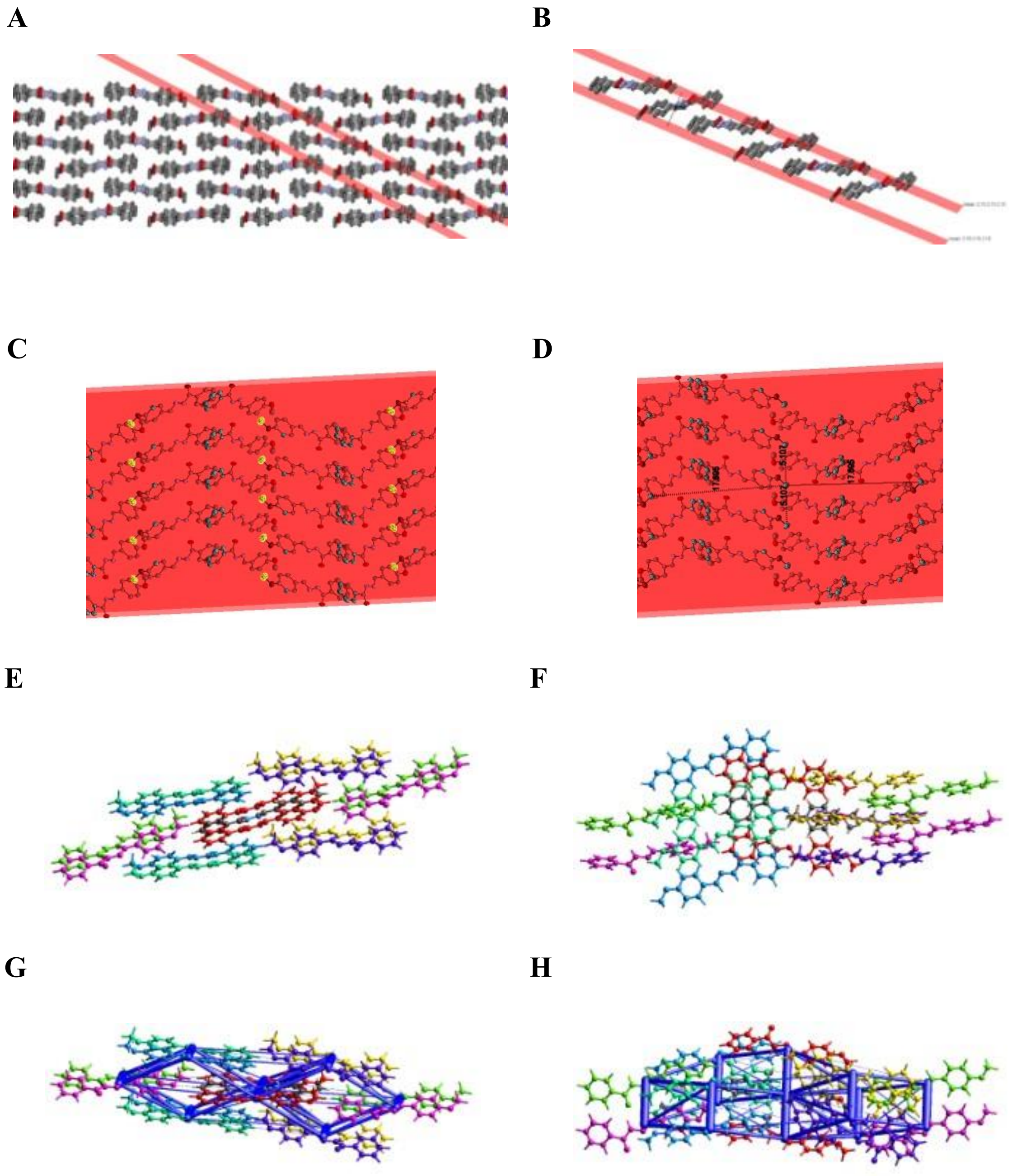

I

	N	Symop	R	Electron Density	E_ele	E_pol	E_dis	E_rep	E_tot
	2	x, y, z	5.11	B3LYP/6-31G(d,p)	-38.8	-11.3	-42.1	49.8	-55.3
	2	-x, -y, z+1/2	8.93	B3LYP/6-31G(d,p)	-11.3	-3.1	-24.9	15.1	-26.6
	2	x+1/2, -y, z	15.73	B3LYP/6-31G(d,p)	-5.5	-0.7	-9.0	6.5	-10.2
	2	-x+1/2, y, z+1/2	8.80	B3LYP/6-31G(d,p)	-3.6	-2.4	-34.0	18.0	-24.1
	2	-x+1/2, y, z+1/2	10.17	B3LYP/6-31G(d,p)	-0.3	-1.8	-10.3	4.3	-7.9
	2	-x, -y, z+1/2	9.83	B3LYP/6-31G(d,p)	-6.8	-1.5	-25.5	15.8	-20.8
	2	x+1/2, -y, z	16.26	B3LYP/6-31G(d,p)	0.8	-0.5	-6.8	5.7	-2.0

Figure S63: *Crystal Lattice Analysis of the crystalline unit AcylHydr (CCDC identifier: RUJQOD). (A,B) Identification of the layer. (C,D) Looking for ordering along two axes and measuring distance between connection points which was found to be 5.1 Å and thus too small. (E-I) Energy calculations using B3LYP/6-31G(d,p) showing the overlap with the layers and the total attractive energy between neighbors which was found to be −190.0 kJ/mol.*

BnUPy (OLICOD)

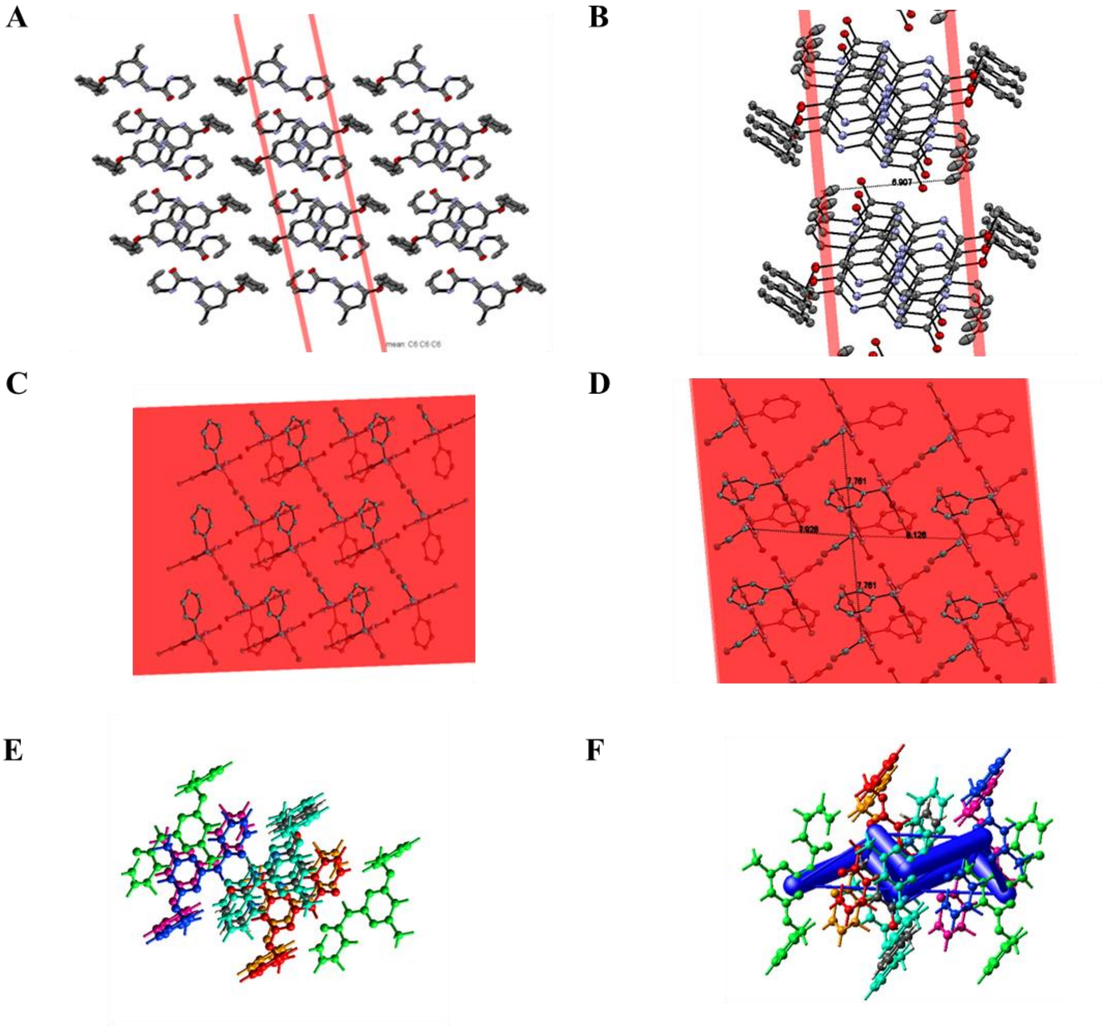

G

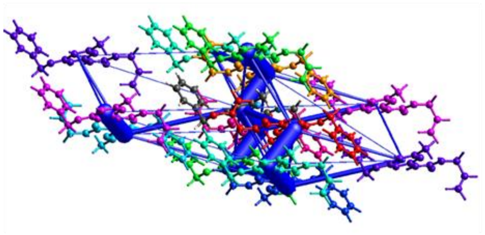

H

[B3LYP/6-31G(d,p)]

	N	Symop	R	E_ele	E_pol	E_dis	E_rep	E_tot
	1	-x, -y, -z	7.70	-13.3	-2.2	-43.0	35.0	-31.5
	1	-x, -y, -z	4.09	-27.0	-4.5	-107.2	72.5	-80.4
	0	-x, -y, -z	10.94	-3.0	-0.5	-15.1	5.2	-13.5
	0	-x, -y, -z	14.07	-1.2	-0.2	-8.2	5.6	-5.2
	2	x, y, z	7.76	-3.4	-2.0	-13.1	5.7	-13.0
	2	x, y, z	7.93	-1.6	-0.7	-14.2	5.6	-11.1
	1	-x, -y, -z	10.21	-8.8	-1.5	-31.6	18.5	-26.4
	1	-x, -y, -z	8.18	-3.1	-0.6	-19.2	10.0	-14.2
	2	x, y, z	15.07	-0.9	-0.1	-7.2	4.6	-4.5
	2	x, y, z	13.00	-1.2	-0.5	-9.6	6.4	-6.0
	1	-x, -y, -z	6.61	-98.9	-29.2	-43.4	121.9	-88.7

Figure S64: *Crystal Lattice Analysis of the crystalline unit BnUPy (CCDC identifier: OLICOD). (A,B) Identification of the layer. (C,D) Looking for ordering along two axes and measuring distance between connection points which was found to be 7.8 Å and thus sufficiently large . (E-H) Energy calculations using B3LYP/6-31G(d,p) showing the overlap with the layers and the total attractive energy between neighbors which was found to be −255.2 kJ/mol.*

Cholesterol (BUGLEU)

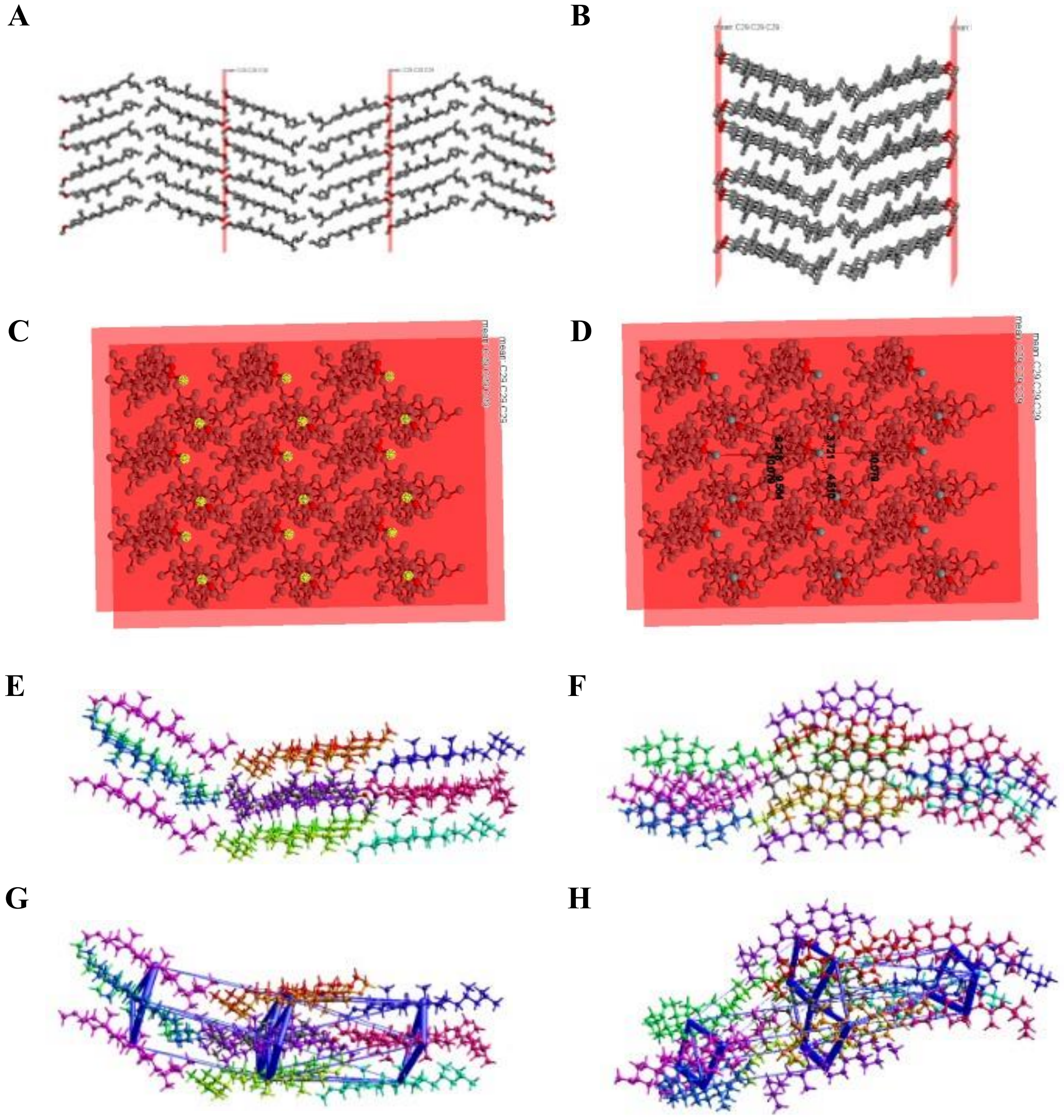

I

[B3LYP/6-31G(d,p)]

	N	Symop	R	E_ele	E_pol	E_dis	E_rep	E_tot
	0	-	6.46	-6.5	-0.4	-68.8	28.0	-49.8
	0	-	7.01	-3.0	-0.2	-47.0	16.0	-34.3
	0	x, y, z	7.65	-3.5	-0.1	-33.7	14.6	-24.1
	0	-	6.34	-6.2	-0.3	-60.8	27.7	-42.5
	0	-	5.73	-8.9	-0.8	-75.4	32.0	-55.9
	0	-	18.43	-1.2	-0.1	-13.6	5.3	-9.9
	0	-	16.01	-1.8	-0.2	-10.8	6.3	-7.5
	0	-x+1/2, -y, z+1/2	16.10	-1.5	-0.8	-16.2	5.8	-12.8
	0	-	20.31	-1.2	-0.1	-6.6	4.3	-4.3
	0	-	18.36	-3.3	-1.1	-11.1	5.9	-10.3
	2	x, y, z	7.65	-3.9	-0.1	-35.1	15.5	-25.1
	2	x+1/2, -y+1/2, -z	19.10	-0.9	-0.0	-7.2	2.9	-5.5
	2	-x+1/2, -y, z+1/2	17.71	-3.9	-1.2	-13.3	8.8	-11.2

Figure S65: *Crystal Lattice Analysis of the crystalline unit Cholesterol (CCDC identifier: BUGLEU). (A,B) Identification of the layer. (C,D) Looking for ordering along two axes and measuring distance between connection points which was found to be 3.7 Å and thus too small. (E-I) Energy calculations using B3LYP/6-31G(d,p) showing the overlap with the layers and the total attractive energy between neighbors which was found to be −232.7 kJ/mol.*

DPA (DPANTR)

A

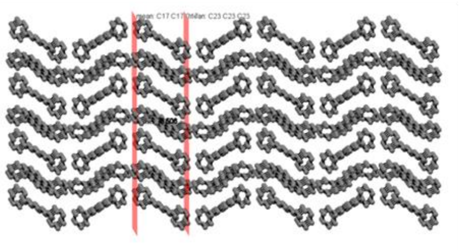

B

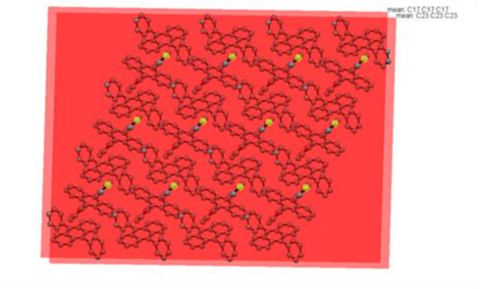

C

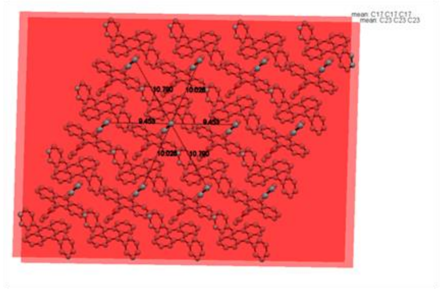

D

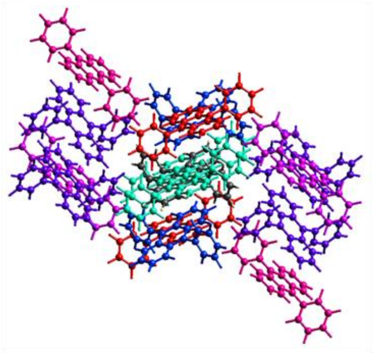

E

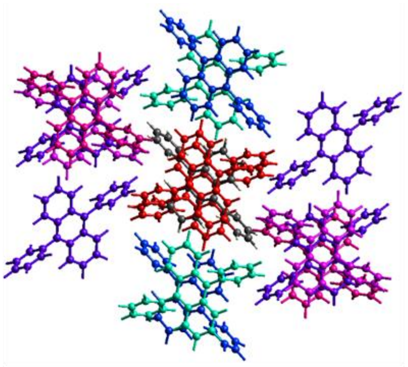

F

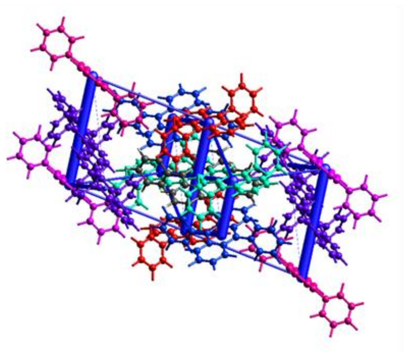

G

[B3LYP/6-31G(d,p)]

	N	Symop	R	E_ele	E_pol	E_dis	E_rep	E_tot
(red)	0	-	5.01	-11.8	-2.6	-85.1	53.4	-55.6
(cyan)	0	-	8.82	-4.2	-0.7	-30.3	15.5	-21.7
(blue)	2	x, y, z	9.45	-5.4	-0.7	-29.0	20.5	-18.8
(violet)	4	-x+1/2, y+1/2, -z	11.21	-1.4	-0.2	-11.6	3.7	-9.4
(magenta)	2	-	11.51	-1.0	-0.1	-8.6	2.3	-7.1
(pink)	2	-	13.01	-0.8	-0.1	-6.9	2.8	-5.3

Figure S66 *: Crystal Lattice Analysis of the crystalline unit DPA (CCDC identifier: DPANTR). (A) Identification of the layer. (B,C) Looking for ordering along two axes and measuring distance between connection points which was found to be 9.5 Å and thus sufficiently large. (D-G) Energy calculations using B3LYP/6-31G(d,p) showing the overlap with the layers and the total attractive energy between neighbors which was found to be −192.2 kJ/mol.*

HAQ and MeAQ (ANTQUO)

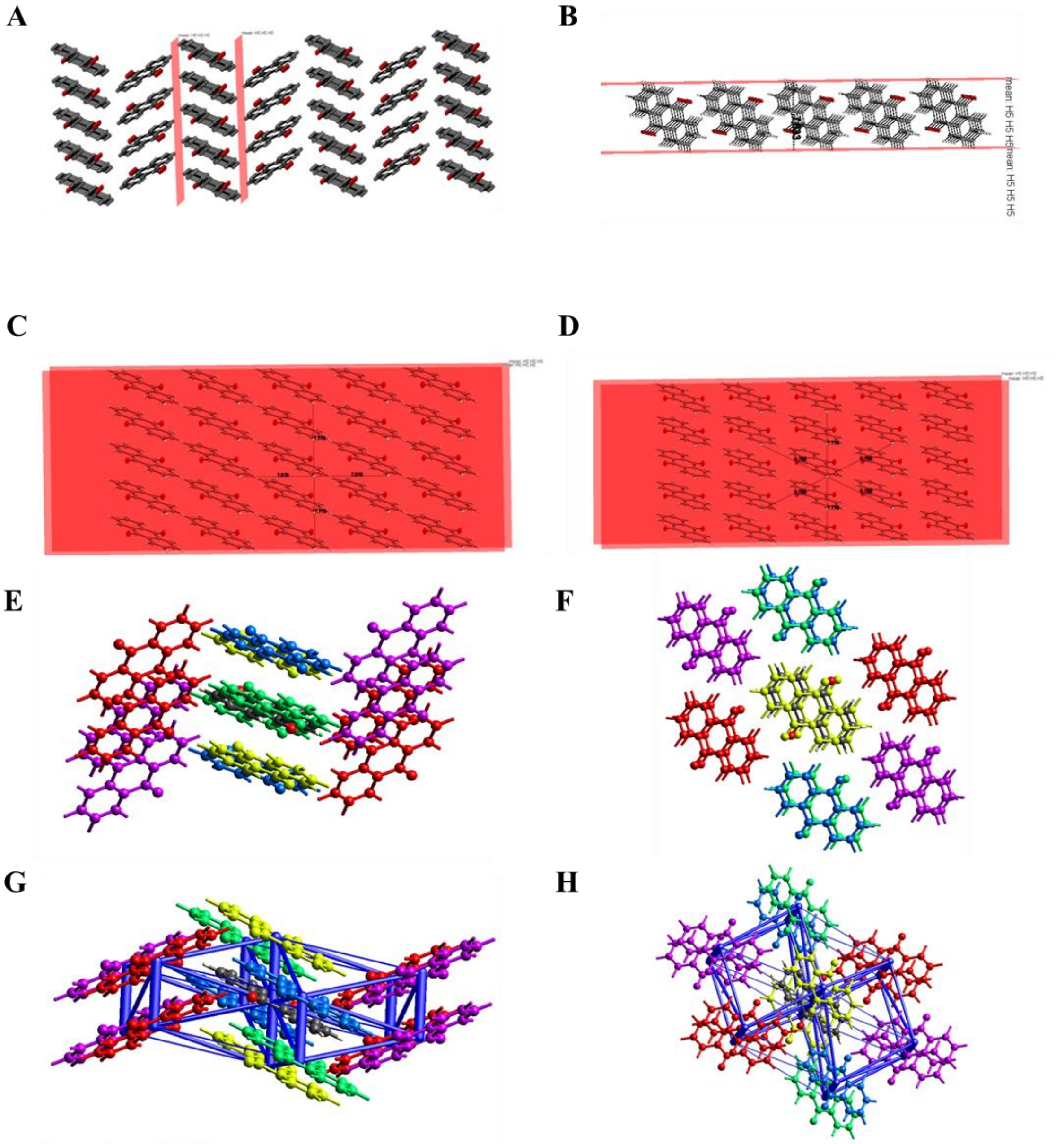

I [B3LYP/6-31G(d,p)]

	N	Symop	R	E_ele	E_pol	E_dis	E_rep	E_tot
	0	-x, y+1/2, -z+1/2	8.06	-7.7	-2.1	-11.4	9.2	-14.0
	0	x, y, z	3.89	-3.5	-0.7	-66.2	38.7	-37.9
	0	x, y, z	8.79	-8.2	-1.5	-14.5	12.2	-14.8
	0	x, y, z	7.88	-5.8	-1.9	-14.6	9.1	-14.7
	0	-x, y+1/2, -z+1/2	10.01	-0.7	-0.6	-10.2	7.7	-5.3

Figure S67*: Crystal Lattice Analysis of the crystalline unit HAQ, MeAQ (CCDC identifier: ANTQUO). (A,B) Identification of the layer. (C,D) Looking for ordering along two axes and measuring distance between connection points which was found to be 8.7 Å and thus sufficiently large. (E-I) Energy calculations using B3LYP/6-31G(d,p) showing the overlap with the layers and the total attractive energy between neighbors which was found to be −134.8 kJ/mol.*

HOPV, MeOPV and PentOPV (REDHIR02)

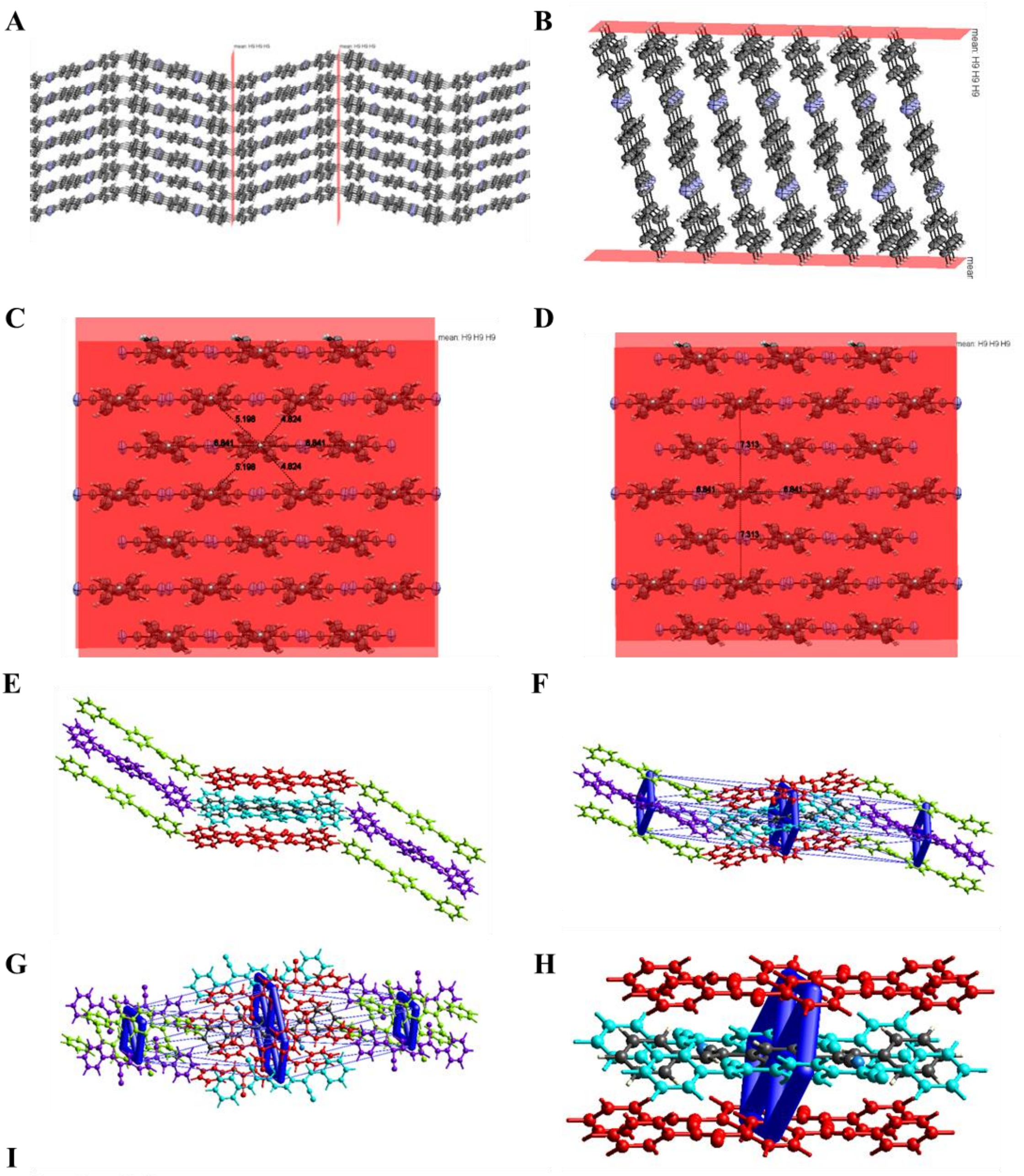

I

[B3LYP/6-31G(d,p)]

	N	Symop	R	E_ele	E_pol	E_dis	E_rep	E_tot
(red)	0	x+1/2, -y+1/2, -z	5.01	-10.7	-3.9	-65.0	31.8	-51.2
(green)	0	-x, y+1/2, -z+1/2	18.14	-0.3	-0.2	-5.5	1.3	-4.4
(cyan)	0	x, y, z	6.84	-31.8	-9.4	-24.4	32.5	-41.8
(purple)	0	-x+1/2, -y, z+1/2	18.09	-1.1	-0.4	-8.9	6.1	-5.4

Figure S68*: Crystal Lattice Analysis of the crystalline unit HOPV, MeOPV and PentOPV (CCDC identifier: REDHIR02). (A,B) Identification of the layer. (C,D) Looking for ordering along two axes and measuring distance between connection points which was found to be 6.8 Å and thus slightly too small. (E-I) Energy calculations using B3LYP/6-31G(d,p) showing the overlap with the layers and the total attractive energy between neighbors which was found to be −288.4 kJ/mol.*

OPV Polymorph 2 (SAPNII01)

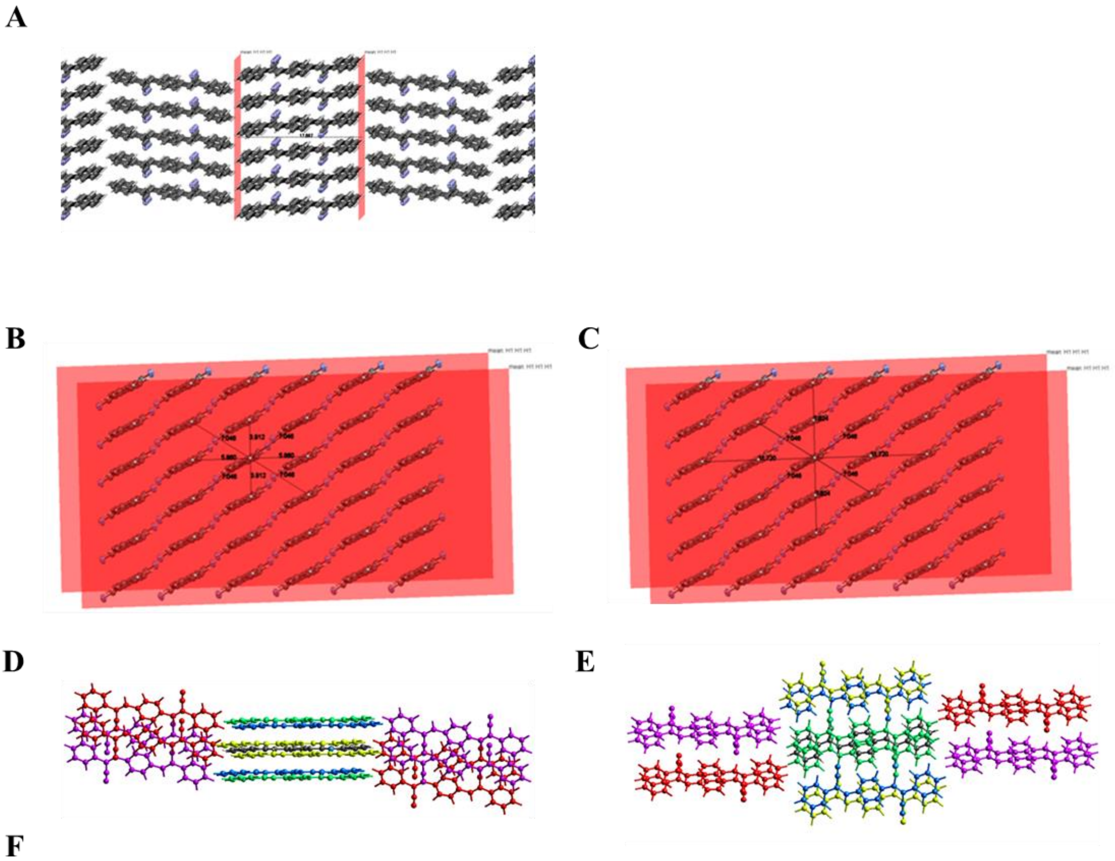

F

[B3LYP/6-31G(d,p)]

	N	Symop	R	E_ele	E_pol	E_dis	E_rep	E_tot
(red)	0	-x+1/2, y+1/2, -z+1/2	18.54	-0.5	-0.4	-8.1	4.8	-4.9
(yellow)	0	x, y, z	7.05	-25.7	-8.1	-27.4	26.1	-40.9
(green)	0	x, y, z	3.91	-3.0	-2.5	-102.3	54.9	-60.2
(blue)	0	x, y, z	5.86	-6.1	-4.1	-47.3	25.3	-35.1
(magenta)	4	-x+1/2, y+1/2, -z+1/2	18.99	-0.4	-0.3	-6.1	3.2	-4.0

Figure S69: *Crystal Lattice Analysis of an OPV polymorph (CCDC identifier: SAPNII01). (A) Identification of the layer. (B,C) Looking for ordering along two axes and measuring distance between connection points which was found to be 7.0 Å and thus exactly large enough. (D-F) Energy calculations using B3LYP/6-31G(d,p) showing the overlap with the layers and the total attractive energy between neighbors which was found to be −272.4 kJ/mol.*

OPV Polymorph 3 (SAPNII)

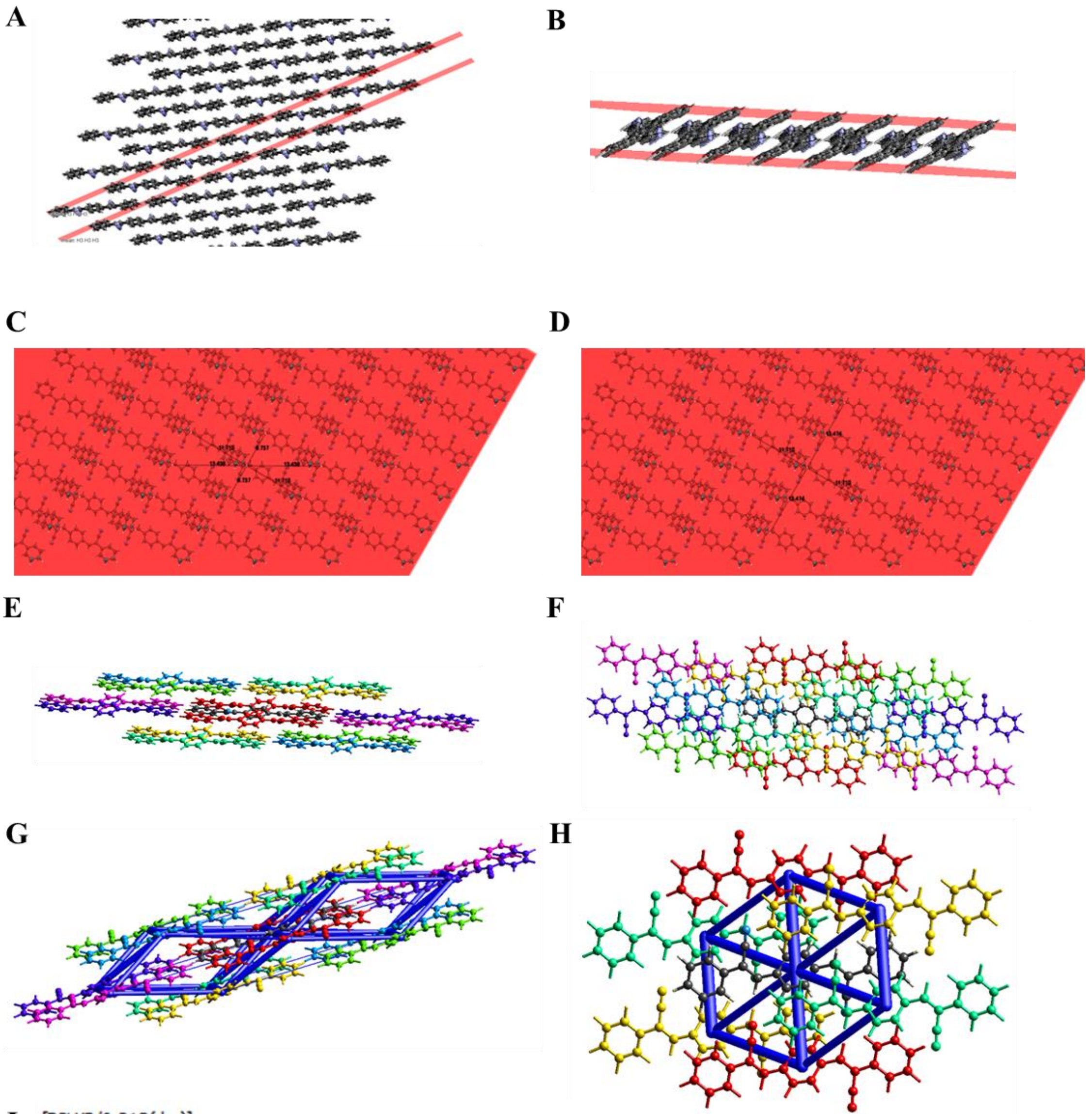

I

[B3LYP/6-31G(d,p)]

	N	Symop	R	E_ele	E_pol	E_dis	E_rep	E_tot
	0	x, y, z	6.74	-29.9	-9.1	-28.9	34.5	-42.2
	0	x, y, z	8.98	-4.4	-2.4	-39.3	19.1	-28.9
	0	x, y, z	13.44	-6.7	-2.8	-10.5	5.9	-14.6
	0	x, y, z	8.97	-6.8	-1.4	-44.1	18.8	-35.1
	0	x, y, z	11.71	-4.0	-0.9	-46.7	24.2	-30.7
	0	x, y, z	18.98	-1.1	-0.3	-8.3	5.1	-5.5
	0	x, y, z	19.04	0.9	-0.4	-6.6	3.3	-3.1

Figure S70*: Crystal Lattice Analysis of an OPV polymorph (CCDC identifier: SAPNII). (A,B) Identification of the layer. (C,D) Looking for ordering along two axes and measuring distance between connection points which was found to be 11.7 Å and thus sufficiently large. (E-I) Energy calculations using B3LYP/6-31G(d,p) showing the overlap with the layers and the total attractive energy between neighbors which was found to be −212.4 kJ/mol.*

MeAzo, PentAzo (AzPhen10)

Note, the crystal is disordered at the ethoxy group. Thus, not all hydrogen atoms are defined. The CrystalExplorer calculations will therefore have a larger error. Calculations were performed on HF/3-21G level.

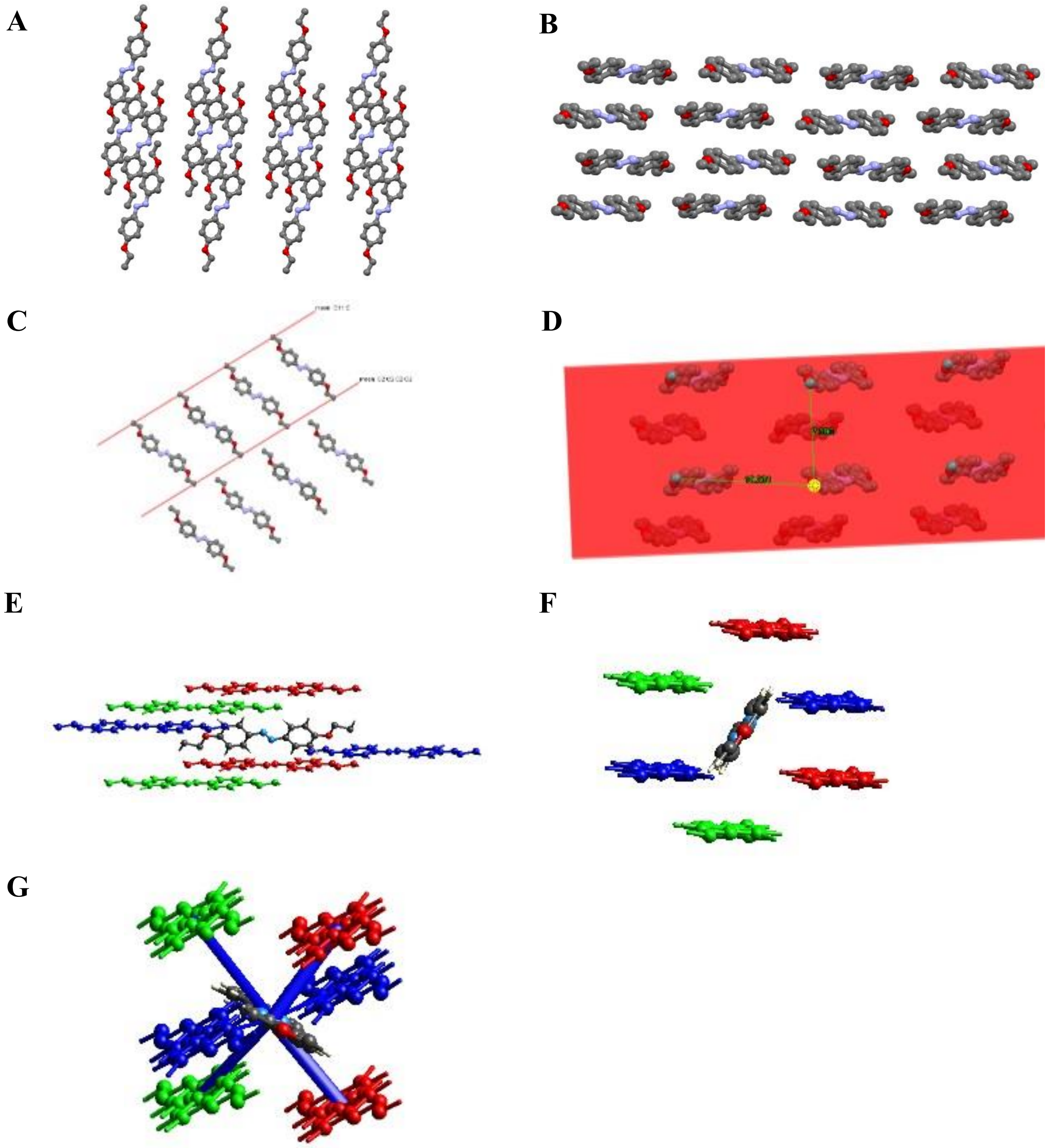

H

	N	Symop	R	Electron Density	E_ele	E_pol	E_dis	E_rep	E_tot
(red)	2	-x, y+1/2, -z+1/2	4.82	HF/3-21G	-8.7	-2.2	-41.9	18.1	-33.3
(green)	2	-x, y+1/2, -z+1/2	8.69	HF/3-21G	-5.7	-1.7	-30.0	12.2	-24.0
(blue)	2	x, -y+1/2, z+1/2	12.36	HF/3-21G	-0.3	-1.1	-12.4	3.6	-9.2

***Figure S71**: Crystal Lattice Analysis of the crystalline unit MeAzo and PentAzo (CCDC identifier: AzPhen10). (A,B) Identification of the layer. (C,D) Looking for ordering along two axes and measuring distance between connection points which was found to be 7.6 Å and thus sufficiently large. (E-H) Energy calculations using HF/3-21G showing the overlap with the layers and the total attractive energy between neighbors which was found to be −204.0 kJ/mol.*

NDI and NDI-NDI (DAHMUX)

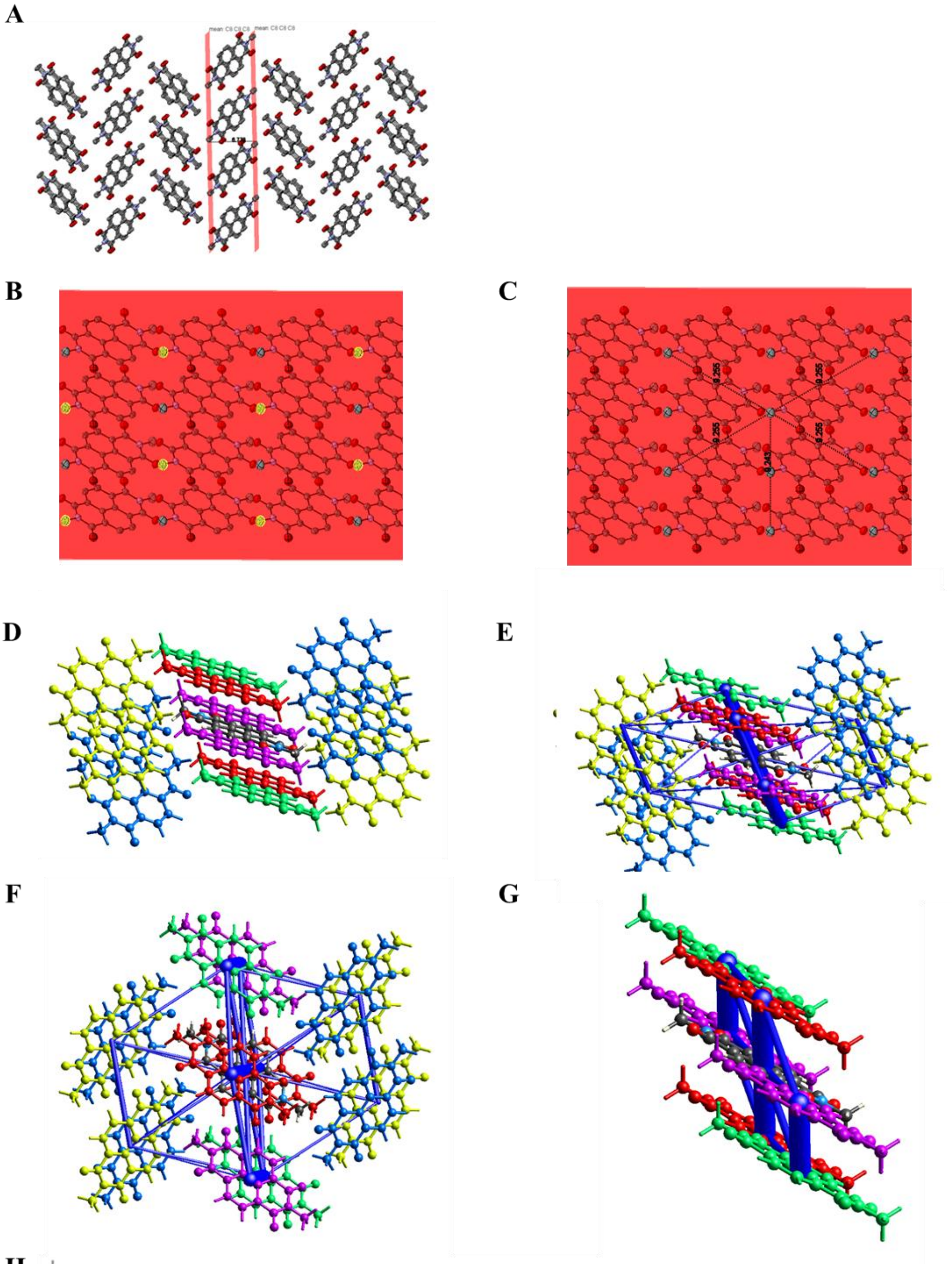

H

	N	Symop	R	Electron Density	E_ele	E_pol	E_dis	E_rep	E_tot
	2	x, y, z	4.62	B3LYP/6-31G(d,p)	-18.1	-3.8	-78.1	47.0	-60.9
	4	-x, y+1/2, -z+1/2	10.74	B3LYP/6-31G(d,p)	-6.8	-1.6	-8.3	6.4	-11.6
	2	x, y, z	8.02	B3LYP/6-31G(d,p)	-5.0	-1.8	-28.0	12.8	-23.2
	0	-x, y+1/2, -z+1/2	9.41	B3LYP/6-31G(d,p)	1.1	-0.6	-12.7	5.1	-7.2
	2	x, y, z	9.26	B3LYP/6-31G(d,p)	-21.2	-3.9	-12.2	21.8	-22.5

Figure S72*: Crystal Lattice Analysis of the crystalline unit NDI and NDI-NDI (CCDC identifier: DAHMUX). (A) Identification of the layer. (B,C) Looking for ordering along two axes and measuring distance between connection points which was found to be 9.2 Å and thus sufficiently large. (D-H) Energy calculations using B3LYP/6-31G(d,p) showing the overlap with the layers and the total attractive energy between neighbors which was found to be −213.2 kJ/mol.*

Ethyl-NDI (BIYRIM)

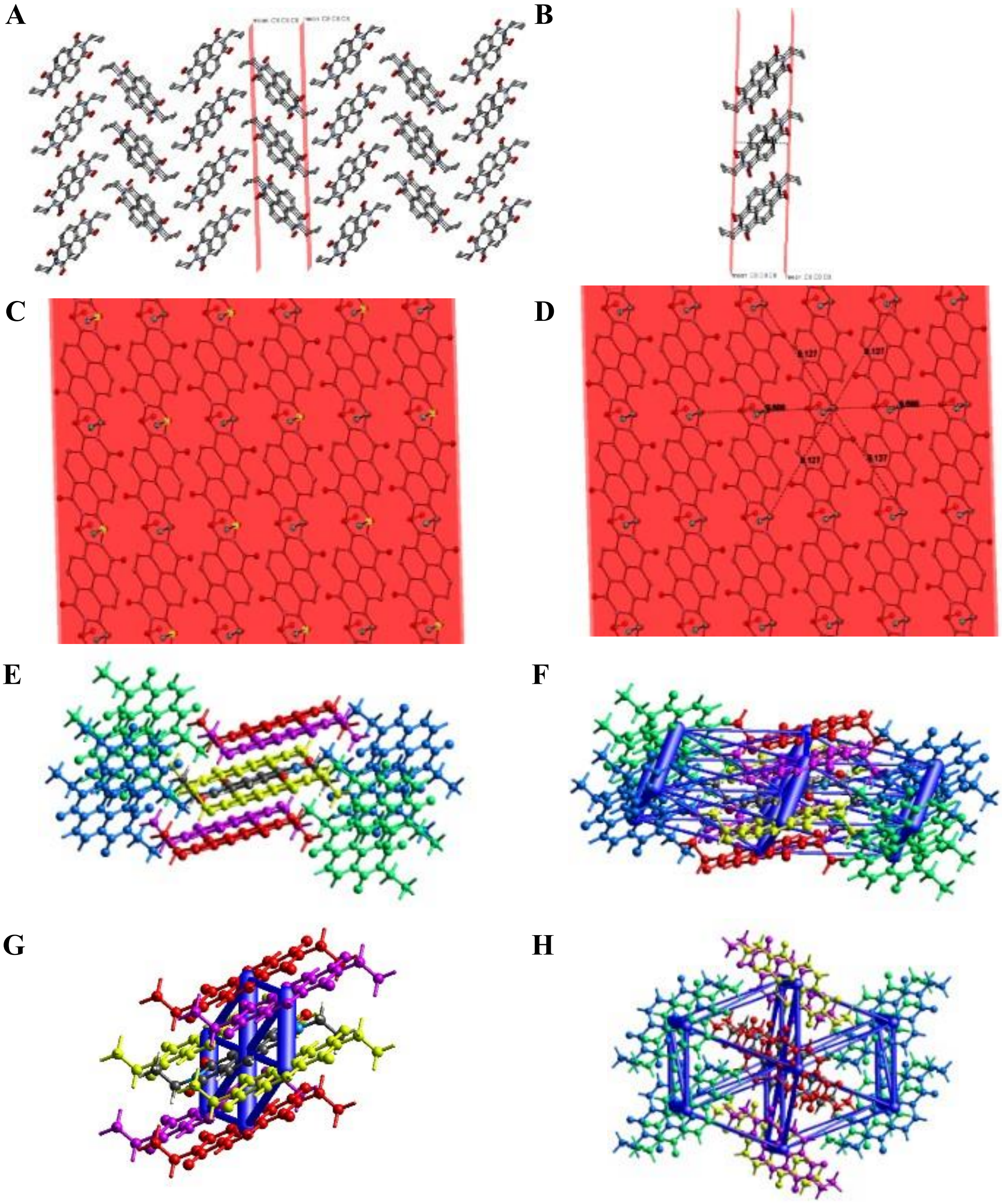

I

	N	Symop	R	Electron Density	E_ele	E_pol	E_dis	E_rep	E_tot
	2	x, y, z	4.84	B3LYP/6-31G(d,p)	-20.2	-4.9	-86.1	59.2	-63.4
	2	x, y, z	9.13	B3LYP/6-31G(d,p)	-23.1	-4.5	-14.2	24.5	-25.1
	4	-x, y+1/2, -z+1/2	9.94	B3LYP/6-31G(d,p)	-1.4	-0.6	-22.1	13.5	-12.8
	4	-x, y+1/2, -z+1/2	11.07	B3LYP/6-31G(d,p)	-6.8	-1.9	-11.5	10.7	-12.0
	2	x, y, z	7.74	B3LYP/6-31G(d,p)	-8.1	-1.7	-36.3	20.5	-28.7

***Figure S73**: Crystal Lattice Analysis of the crystalline unit Ethyl-NDI (CCDC identifier: BIYRIM). (A,B) Identification of the layer. (C,D) Looking for ordering along two axes and measuring distance between connection points which was found to be 9.1 Å and thus sufficiently large. (E-I) Energy calculations using B3LYP/6-31G(d,p) showing the overlap with the layers and the total attractive energy between neighbors which was found to be −234.4 kJ/mol.*

Propyl-NDI (DAHLOQ)

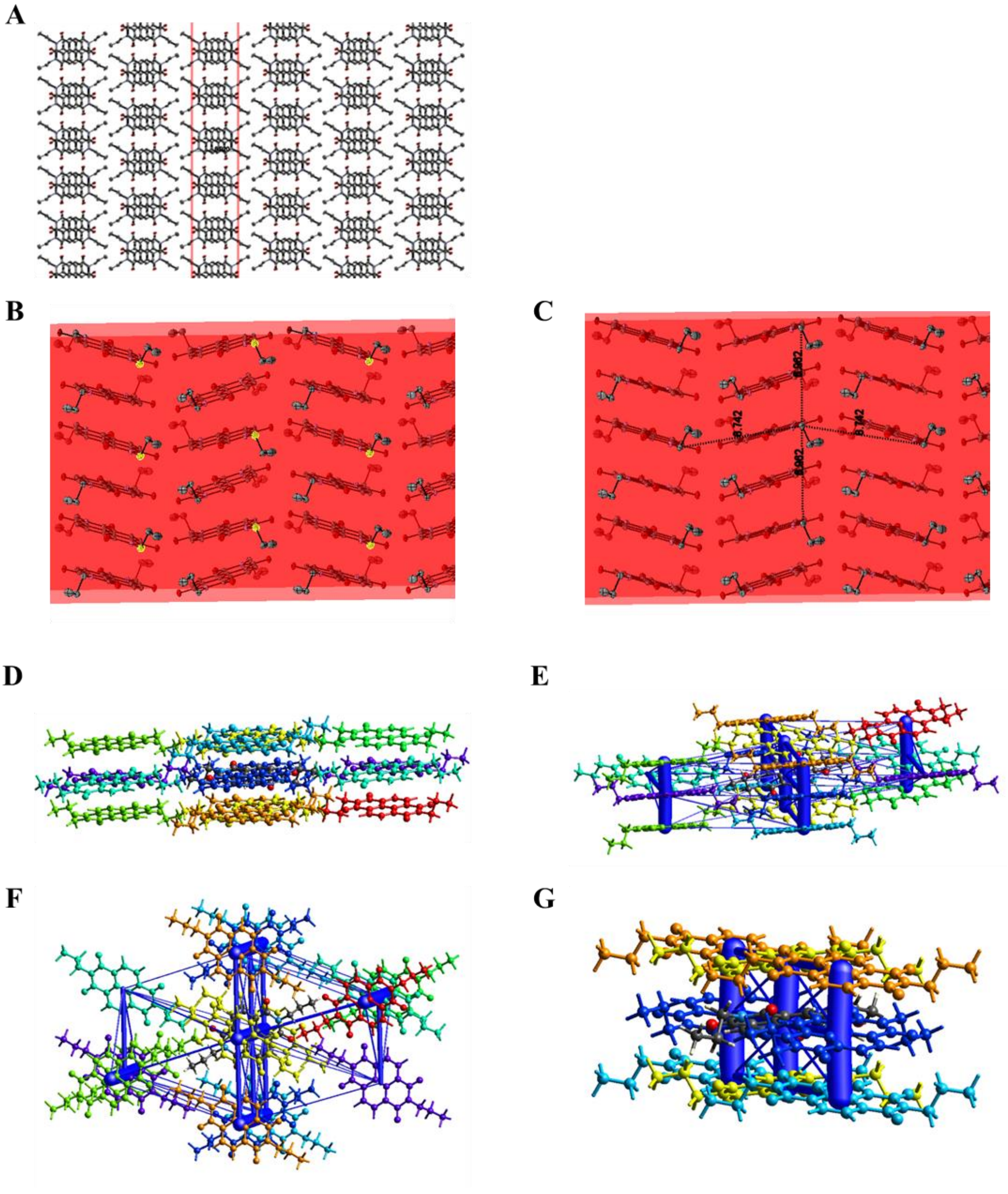

H

	N	Symop	R	Electron Density	E_ele	E_pol	E_dis	E_rep	E_tot
	1	-x, -y, -z	14.69	B3LYP/6-31G(d,p)	-0.2	-0.1	-7.1	1.8	-5.3
	2	-x, y+1/2, -z+1/2	9.28	B3LYP/6-31G(d,p)	-1.6	-0.8	-15.5	10.5	-9.4
	2	x+1/2, y, -z+1/2	3.48	B3LYP/6-31G(d,p)	-16.4	-4.3	-119.2	73.6	-78.9
	2	x+1/2, -y+1/2, -z	15.02	B3LYP/6-31G(d,p)	-0.9	-0.2	-8.9	4.5	-6.1
	1	-x, -y, -z	14.72	B3LYP/6-31G(d,p)	-2.2	-0.2	-12.1	6.3	-9.2
	2	-x+1/2, -y, z+1/2	14.39	B3LYP/6-31G(d,p)	-0.7	-1.0	-9.4	5.0	-6.5
	2	-x, y+1/2, -z+1/2	9.32	B3LYP/6-31G(d,p)	-1.5	-0.6	-14.5	9.0	-9.0
	2	-x+1/2, y+1/2, z	8.62	B3LYP/6-31G(d,p)	-21.0	-5.1	-20.4	25.9	-27.7
	2	x, -y+1/2, z+1/2	14.51	B3LYP/6-31G(d,p)	-0.4	-0.8	-7.4	3.0	-5.7
	0	x, y, z	6.96	B3LYP/6-31G(d,p)	0.6	-0.1	-6.2	0.0	-4.8
	0	-x+1/2, -y, z+1/2	16.01	B3LYP/6-31G(d,p)	-0.1	-0.0	-0.4	0.0	-0.5

***Figure S74**: Crystal Lattice Analysis of the crystalline unit Propyl-NDI (CCDC identifier: DAHLOQ). (A) Identification of the layer. (B,C) Looking for ordering along two axes and measuring distance between connection points which was found to be 8.7 Å and thus sufficiently large. (D-H) Energy calculations using B3LYP/6-31G(d,p) showing the overlap with the layers and the total attractive energy between neighbors which was found to be −250.0 kJ/mol.*

Butyl-NDI (UNANAZ)

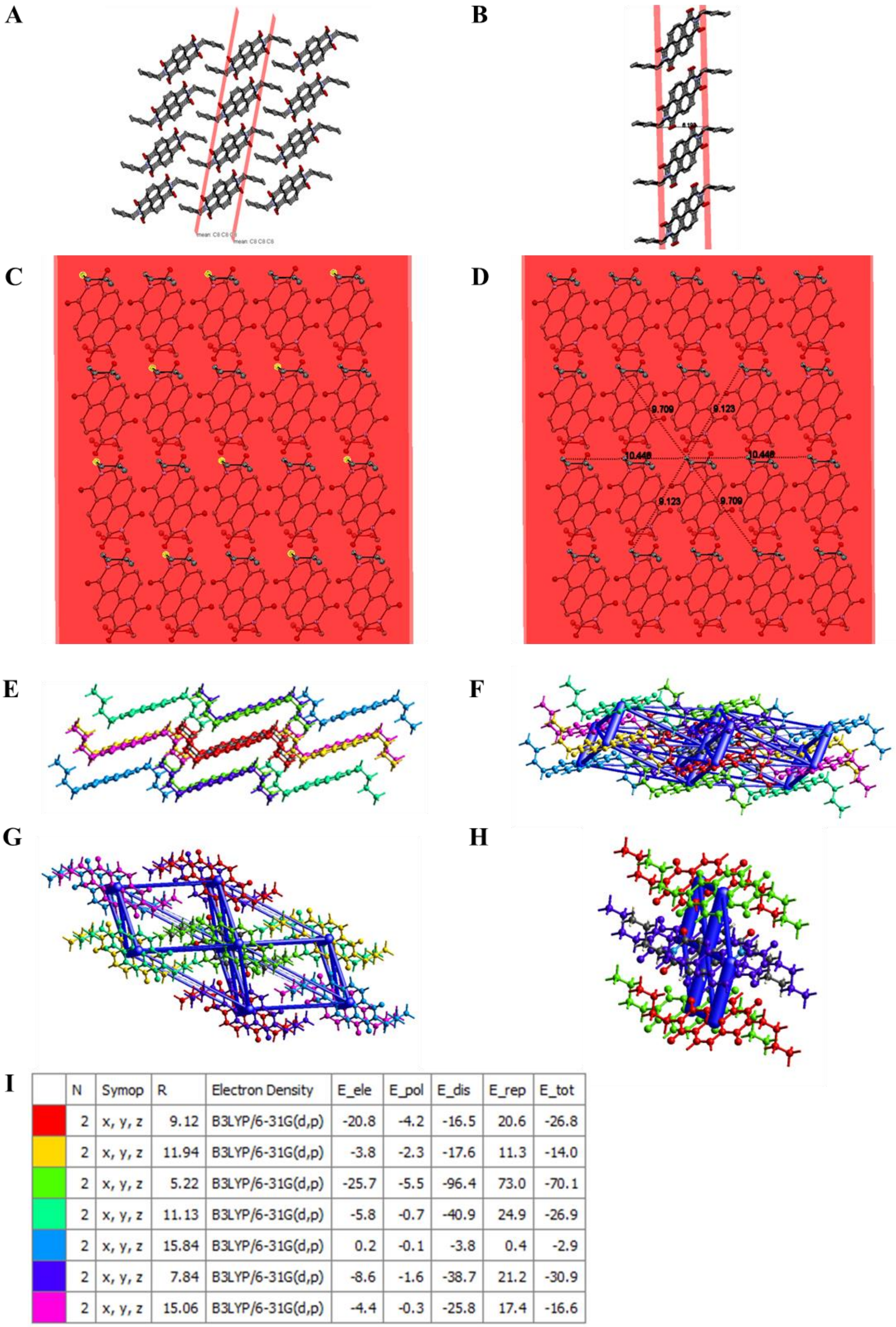

I

	N	Symop	R	Electron Density	E_ele	E_pol	E_dis	E_rep	E_tot
	2	x, y, z	9.12	B3LYP/6-31G(d,p)	-20.8	-4.2	-16.5	20.6	-26.8
	2	x, y, z	11.94	B3LYP/6-31G(d,p)	-3.8	-2.3	-17.6	11.3	-14.0
	2	x, y, z	5.22	B3LYP/6-31G(d,p)	-25.7	-5.5	-96.4	73.0	-70.1
	2	x, y, z	11.13	B3LYP/6-31G(d,p)	-5.8	-0.7	-40.9	24.9	-26.9
	2	x, y, z	15.84	B3LYP/6-31G(d,p)	0.2	-0.1	-3.8	0.4	-2.9
	2	x, y, z	7.84	B3LYP/6-31G(d,p)	-8.6	-1.6	-38.7	21.2	-30.9
	2	x, y, z	15.06	B3LYP/6-31G(d,p)	-4.4	-0.3	-25.8	17.4	-16.6

Figure S75*: Crystal Lattice Analysis of the crystalline unit Butyl-NDI (CCDC identifier: UNANAZ). (A,B) Identification of the layer. (C,D) Looking for ordering along two axes and measuring distance between connection points which was found to be 9.1 Å and thus sufficiently large. (E-I) Energy calculations using B3LYP/6-31G(d,p) showing the overlap with the layers and the total attractive energy between neighbors which was found to be −255.6 kJ/mol.*

Pentyl-NDI (RAGJIT)

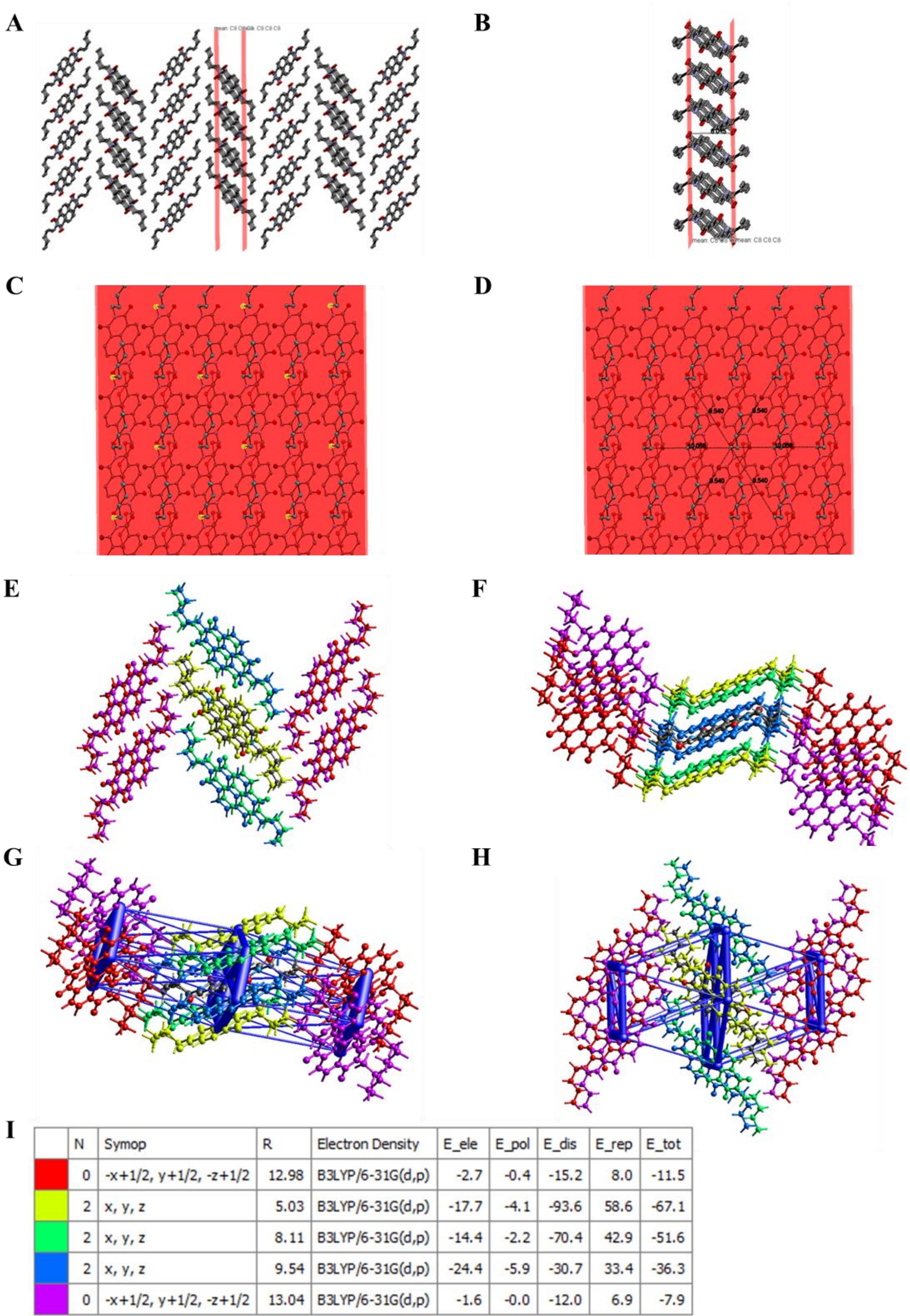

I

	N	Symop	R	Electron Density	E_ele	E_pol	E_dis	E_rep	E_tot
	0	-x+1/2, y+1/2, -z+1/2	12.98	B3LYP/6-31G(d,p)	-2.7	-0.4	-15.2	8.0	-11.5
	2	x, y, z	5.03	B3LYP/6-31G(d,p)	-17.7	-4.1	-93.6	58.6	-67.1
	2	x, y, z	8.11	B3LYP/6-31G(d,p)	-14.4	-2.2	-70.4	42.9	-51.6
	2	x, y, z	9.54	B3LYP/6-31G(d,p)	-24.4	-5.9	-30.7	33.4	-36.3
	0	-x+1/2, y+1/2, -z+1/2	13.04	B3LYP/6-31G(d,p)	-1.6	-0.0	-12.0	6.9	-7.9

Figure S76*: Crystal Lattice Analysis of the crystalline unit Pentyl-NDI (CCDC identifier: RAGJIT). (A,B) Identification of the layer. (C,D) Looking for ordering along two axes and measuring distance between connection points which was found to be 9.5 Å and thus sufficiently large. (E-I) Energy calculations using B3LYP/6-31G(d,p) showing the overlap with the layers and the total attractive energy between neighbors which was found to be −310.0 kJ/mol.*

Dodecyl-NDI (UNANED)

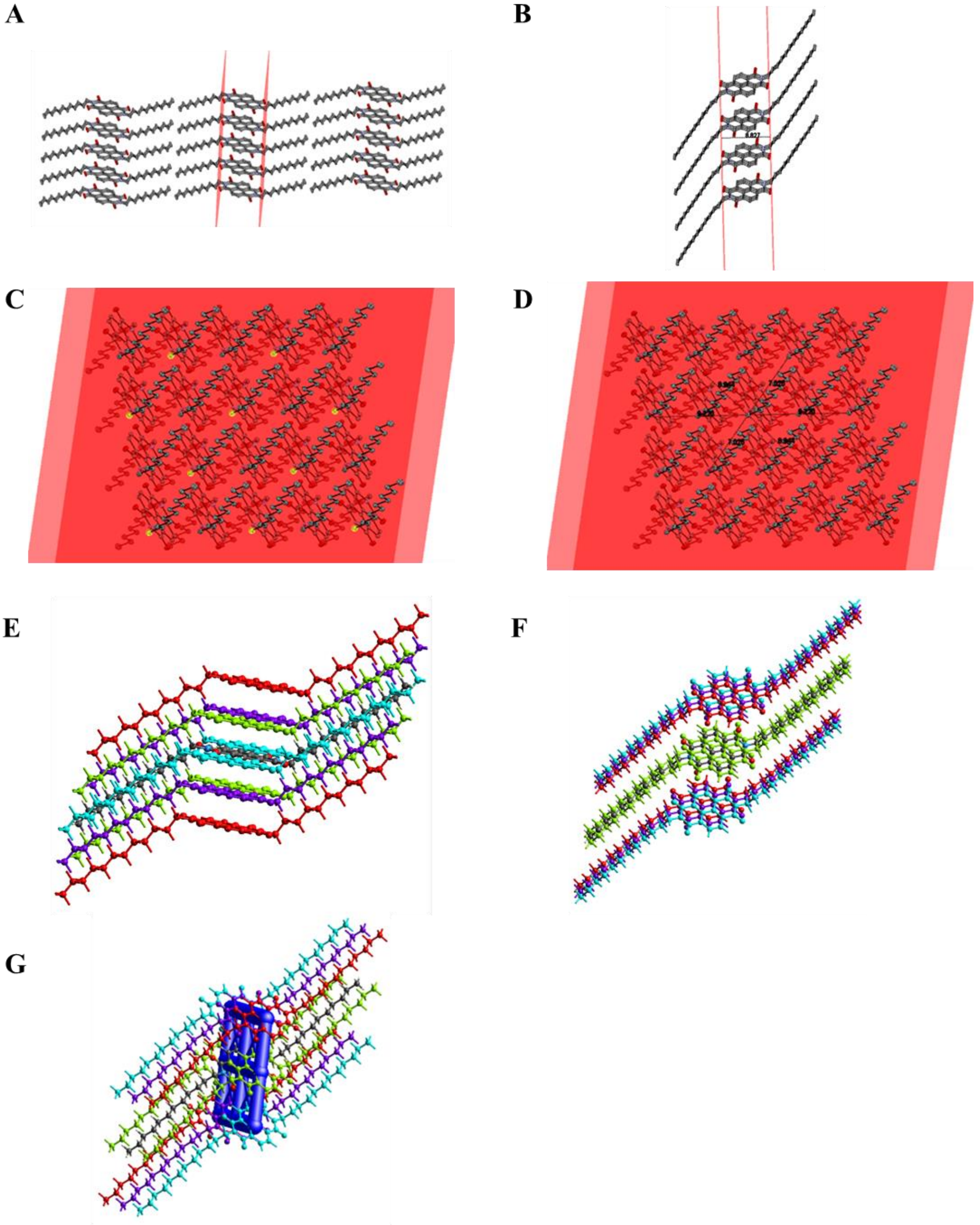

H

	N	Symop	R	Electron Density	E_ele	E_pol	E_dis	E_rep	E_tot
(red)	2	x, y, z	7.03	B3LYP/6-31G(d,p)	-5.1	-0.3	-56.6	27.2	-38.1
(green)	2	x, y, z	4.61	B3LYP/6-31G(d,p)	-23.2	-3.7	-173.0	98.3	-117.1
(cyan)	2	x, y, z	8.94	B3LYP/6-31G(d,p)	-22.3	-5.2	-22.7	24.5	-32.1
(purple)	2	x, y, z	6.59	B3LYP/6-31G(d,p)	-19.7	-6.6	-135.6	78.2	-95.5

Figure S77: *Crystal Lattice Analysis of the crystalline unit dodecyl-NDI (CCDC identifier: UNANED). (A,B) Identification of the layer. (C,D) Looking for ordering along two axes and measuring distance between connection points which was found to be 7.8 Å and thus sufficiently large. (E-H) Energy calculations using B3LYP/6-31G(d,p) showing the overlap with the layers and the total attractive energy between neighbors which was found to be −565.6 kJ/mol.*

NitroHydr (YEFFAR)

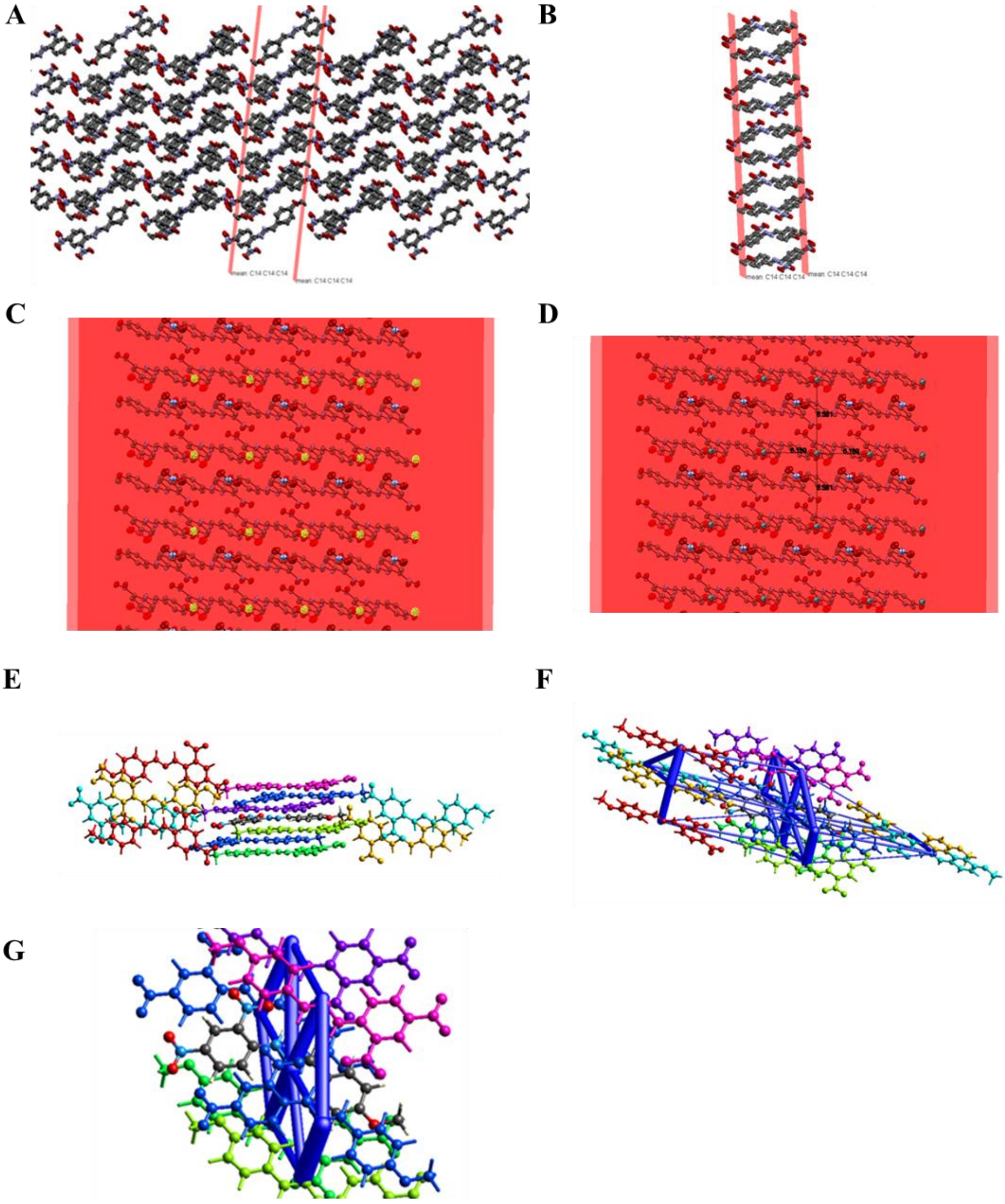

H

	N	Symop	R	Electron Density	E_ele	E_pol	E_dis	E_rep	E_tot
(red)	2	-x+1/2, y+1/2, -z+1/2	12.54	B3LYP/6-31G(d,p)	-3.2	-1.8	-6.5	2.1	-9.1
(orange)	2	x+1/2, -y+1/2, z+1/2	14.03	B3LYP/6-31G(d,p)	-7.0	-2.1	-12.5	7.9	-14.9
(yellow-green)	1	-x, -y, -z	8.29	B3LYP/6-31G(d,p)	-17.8	-3.6	-33.3	24.3	-35.5
(green)	1	-x, -y, -z	4.41	B3LYP/6-31G(d,p)	-19.0	-2.5	-80.2	46.1	-63.3
(cyan)	2	x+1/2, -y+1/2, z+1/2	17.08	B3LYP/6-31G(d,p)	-8.1	-1.3	-5.1	5.9	-10.3
(blue)	2	x, y, z	6.19	B3LYP/6-31G(d,p)	0.2	-2.6	-45.0	21.8	-27.5
(purple)	1	-x, -y, -z	7.44	B3LYP/6-31G(d,p)	-42.8	-7.9	-25.4	40.4	-48.2
(magenta)	1	-x, -y, -z	5.29	B3LYP/6-31G(d,p)	-12.2	-4.1	-62.8	35.7	-48.6

Figure S78*: Crystal Lattice Analysis of the crystalline unit NitroHydr (CCDC identifier: YEFFAR). (A,B) Identification of the layer. (C,D) Looking for ordering along two axes and measuring distance between connection points which was found to be 6.2 Å and thus too small. (E-H) Energy calculations using B3LYP/6-31G(d,p) showing the overlap with the layers and the total attractive energy between neighbors which was found to be −250.6 kJ/mol.*

PentAQ (Supporting information 5)

Crystal structure determined below.

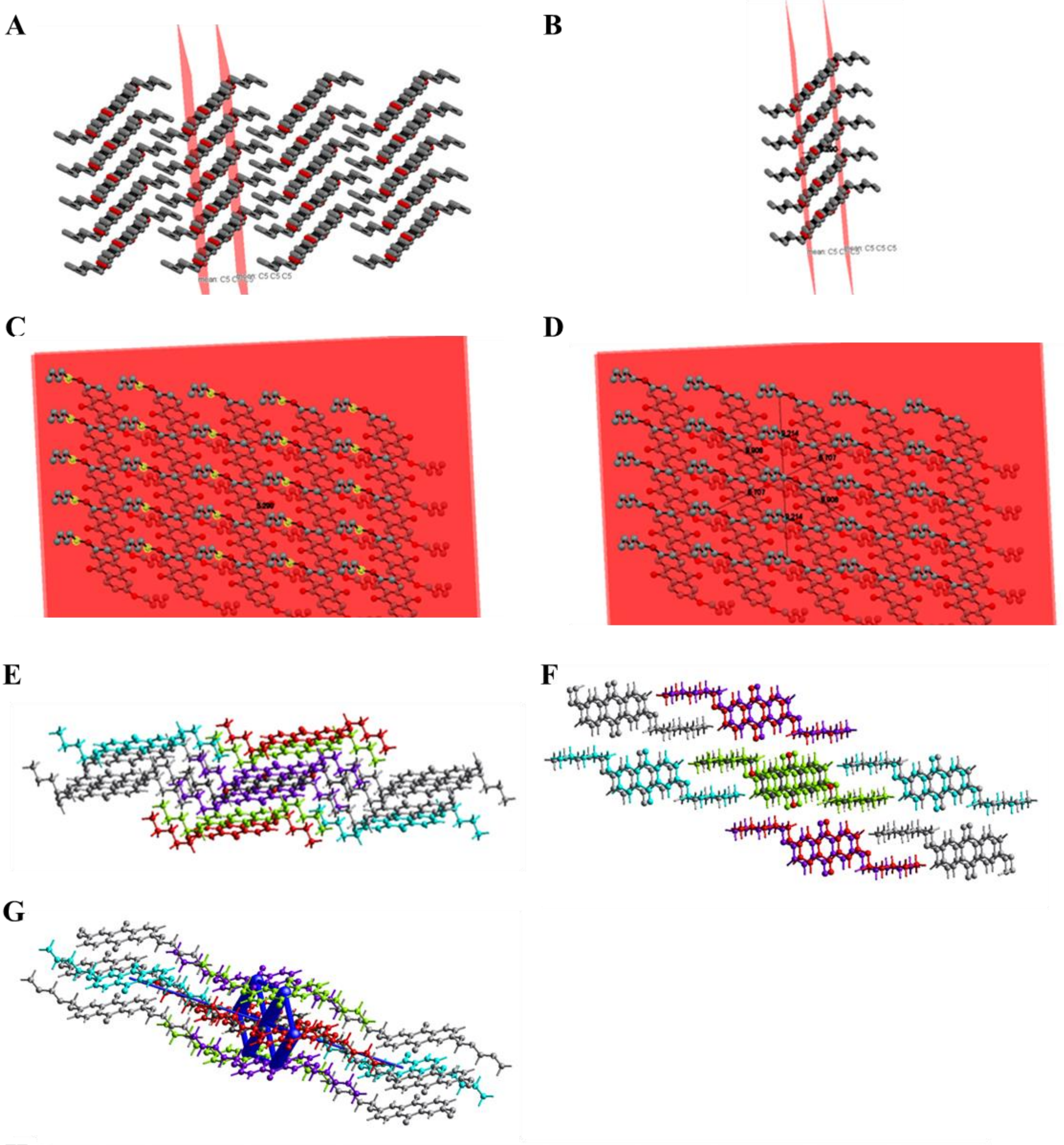

H

	N	Symop	R	Electron Density	E_ele	E_pol	E_dis	E_rep	E_tot
(red)	2	x, y, z	9.91	B3LYP/6-31G(d,p)	-13.4	-4.6	-24.7	18.1	-27.9
(green)	2	x, y, z	4.61	B3LYP/6-31G(d,p)	-15.1	-2.0	-119.1	69.3	-78.4
(cyan)	2	x, y, z	15.50	B3LYP/6-31G(d,p)	-2.5	-0.2	-23.4	12.8	-15.3
(purple)	2	x, y, z	8.11	B3LYP/6-31G(d,p)	-12.4	-5.8	-45.5	35.7	-34.9

Figure S79*: Crystal Lattice Analysis of the crystalline unit PentAQ (Supporting Information 5). (A,B) Identification of the layer. (C,D) Looking for ordering along two axes and measuring distance between connection points which was found to be 8.7 Å and thus sufficiently large. (E-H) Energy calculations using B3LYP/6-31G(d,p) showing the overlap with the layers and the total attractive energy between neighbors which was found to be −282.4 kJ/mol.*

PhePhe (METXEP)

A

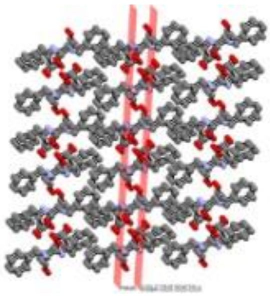

B

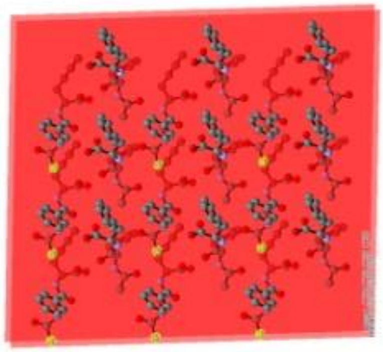

C

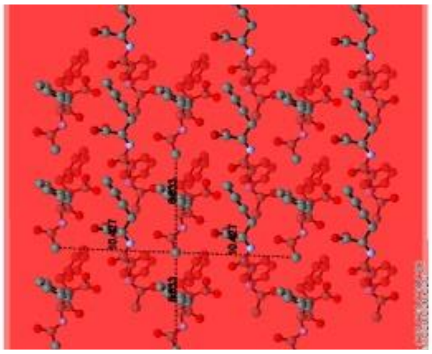

D

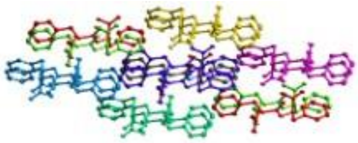

E

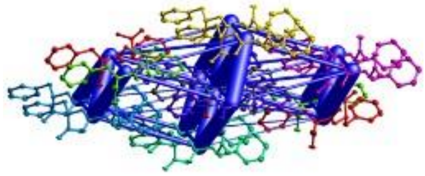

F

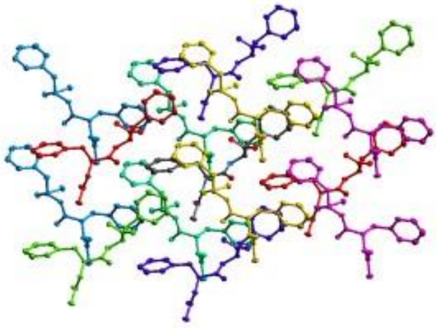

G

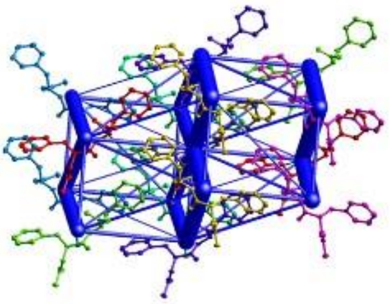

H

	N	Symop	R	Electron Density	E_ele	E_pol	E_dis	E_rep	E_tot
	2	x, y, z	10.19	B3LYP/6-31G(d,p)	-6.1	-1.8	-25.7	13.0	-22.1
	2	-x, y+1/2, -z	6.63	B3LYP/6-31G(d,p)	-105.6	-26.0	-40.0	126.8	-87.4
	2	x, y, z	13.35	B3LYP/6-31G(d,p)	-2.7	-0.6	-20.4	15.0	-11.8
	2	-x, y+1/2, -z	6.94	B3LYP/6-31G(d,p)	-62.2	-20.6	-46.5	73.8	-75.9
	2	-x, y+1/2, -z	13.13	B3LYP/6-31G(d,p)	-0.8	-0.5	-13.3	9.0	-7.1
	2	x, y, z	8.63	B3LYP/6-31G(d,p)	-3.6	-1.8	-19.6	4.7	-19.3
	2	-x, y+1/2, -z	12.27	B3LYP/6-31G(d,p)	-6.4	-2.9	-18.3	14.5	-15.9

Figure S80*: Crystal Lattice Analysis of the crystalline unit PhePhe (CCDC identifier: METXEP). (A) Identification of the layer. (B,C) Looking for ordering along two axes and measuring distance between connection points which was found to be 8.6 Å and thus sufficiently large. (D-H) Energy calculations using B3LYP/6-31G(d,p) showing the overlap with the layers and the total attractive energy between neighbors which was found to be −365.2 kJ/mol.*

PMDI (RAGTIF)

A

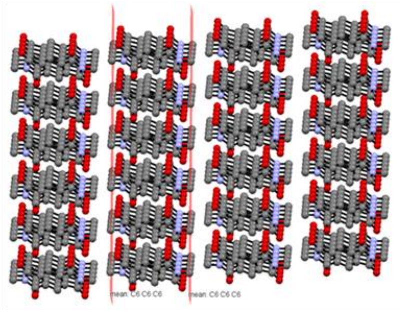

B

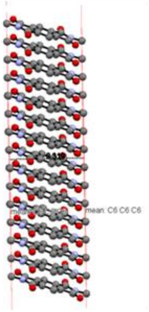

C

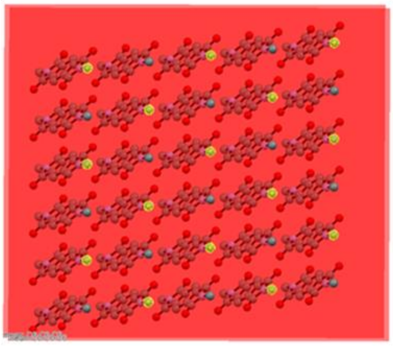

D

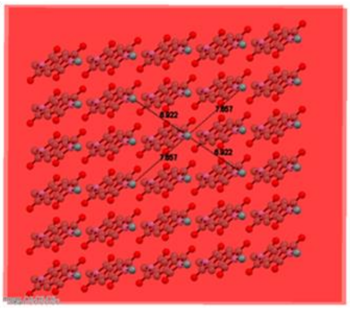

E

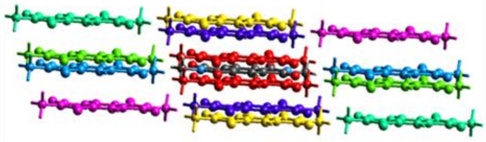

F

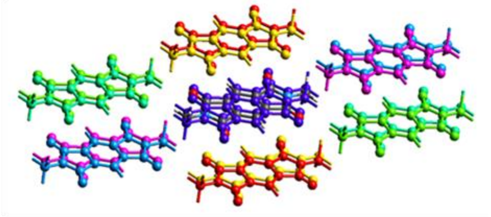

G

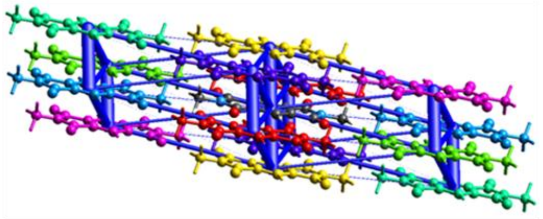

H

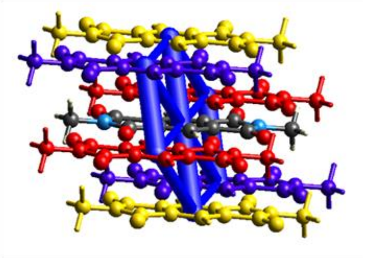

I

	N	Symop	R	Electron Density	E_ele	E_pol	E_dis	E_rep	E_tot
(red)	2	x, y, z	7.66	B3LYP/6-31G(d,p)	-19.5	-3.5	-10.8	20.7	-19.8
(yellow)	2	x, y, z	4.47	B3LYP/6-31G(d,p)	-19.0	-4.2	-57.5	40.7	-48.1
(green)	2	x, y, z	11.03	B3LYP/6-31G(d,p)	-14.1	-2.5	-8.0	12.6	-15.9
(light green)	2	x, y, z	13.14	B3LYP/6-31G(d,p)	1.3	-0.2	-2.0	0.1	-0.4
(light blue)	2	x, y, z	12.90	B3LYP/6-31G(d,p)	0.8	-0.5	-6.0	3.1	-2.7
(blue)	2	x, y, z	5.77	B3LYP/6-31G(d,p)	-11.3	-2.9	-33.6	22.0	-29.8
(magenta)	2	x, y, z	10.49	B3LYP/6-31G(d,p)	-2.0	-1.2	-13.2	6.5	-10.4

Figure S81*: Crystal Lattice Analysis of the crystalline unit PMDI (CCDC identifier: RAGTIF). (A,B) Identification of the layer. (C,D) Looking for ordering along two axes and measuring distance between connection points which was found to be 7.8 Å and thus sufficiently large. (E-I) Energy calculations using B3LYP/6-31G(d,p) showing the overlap with the layers and the total attractive energy between neighbors which was found to be −195.4 kJ/mol.*

Pyrene-OPent (Supporting Information 5)

Crystal structure determined below

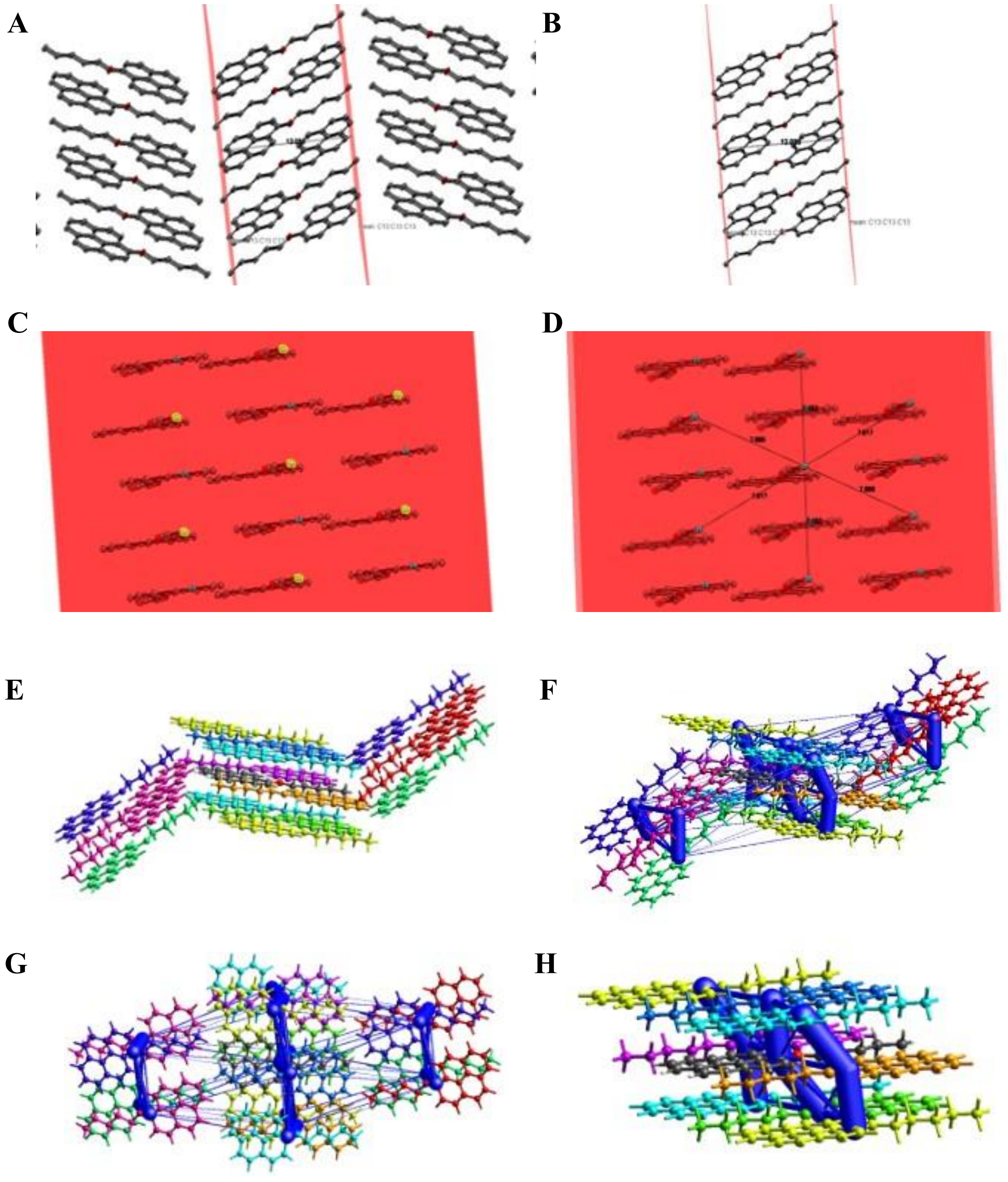

I

	N	Symop	R	Electron Density	E_ele	E_pol	E_dis	E_rep	E_tot
	0	x,-y+1/2,z+1/2	17.72	B3LYP/6-31G(d,p)	-0.0	-0.0	-3.5	1.2	-2.3
	1	-x,-y,-z	8.02	B3LYP/6-31G(d,p)	-4.8	-1.1	-36.6	25.9	-21.8
	2	x,y,z	7.92	B3LYP/6-31G(d,p)	-2.1	-0.6	-15.0	3.2	-13.9
	1	-x,-y,-z	5.48	B3LYP/6-31G(d,p)	-26.8	-3.8	-113.2	77.8	-81.7
	0	-x,y+1/2,-z+1/2	15.50	B3LYP/6-31G(d,p)	-1.2	-0.1	-7.5	5.5	-4.5
	2	x,y,z	7.67	B3LYP/6-31G(d,p)	-1.1	-0.5	-11.2	1.2	-10.6
	1	-x,-y,-z	3.82	B3LYP/6-31G(d,p)	-24.4	-4.9	-121.4	83.1	-83.8
	0	-x,y+1/2,-z+1/2	14.95	B3LYP/6-31G(d,p)	-1.3	-0.1	-8.1	4.8	-5.5
	1	-x,-y,-z	6.81	B3LYP/6-31G(d,p)	-9.1	-0.5	-42.5	33.2	-26.5
	0	x,-y+1/2,z+1/2	12.60	B3LYP/6-31G(d,p)	-2.3	-0.3	-10.7	7.7	-7.3

***Figure S82**: Crystal Lattice Analysis of the crystalline unit Pyrene (Supporting Information 5). (A,B) Identification of the layer. (C,D) Looking for ordering along two axes and measuring distance between connection points which was found to be 7.4 Å and thus sufficiently large. (E-I) Energy calculations using B3LYP/6-31G(d,p) showing the overlap with the layers and the total attractive energy between neighbors which was found to be −262.8 kJ/mol.*

Thiophene (EYUXEB)

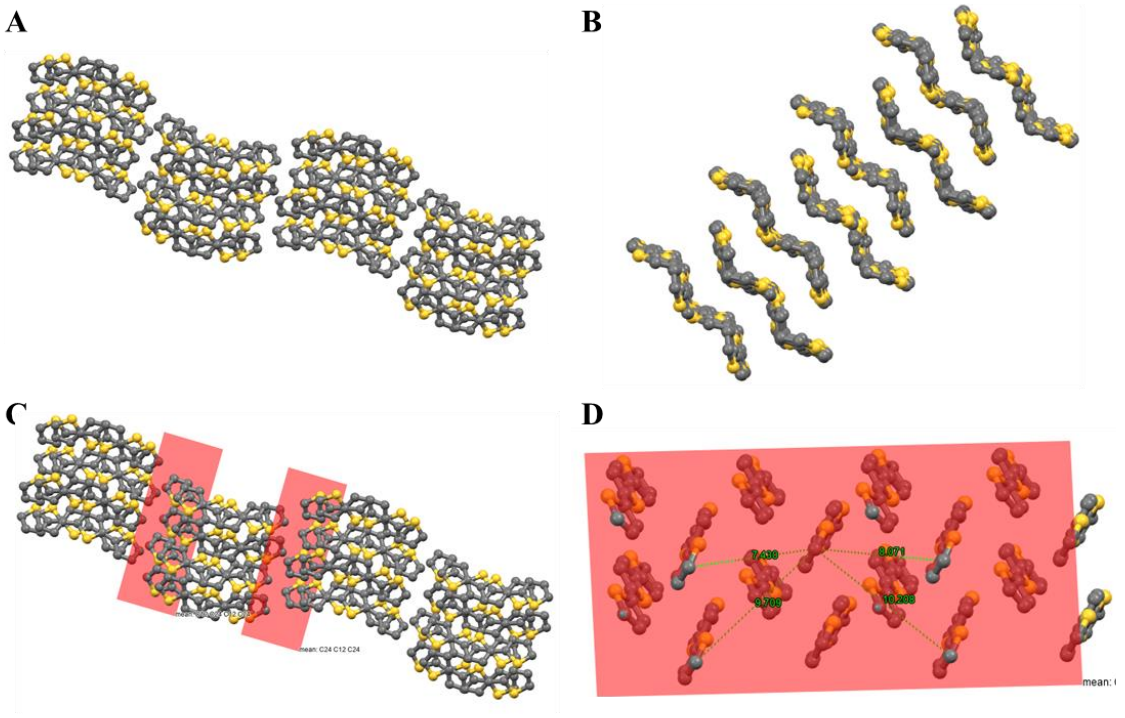

Figure S83*: Crystal Structure Analysis of the crystalline unit Thiophene (CCDC identifier: EYUXEB). (A,B) Identification of the layer. (C,D) Looking for ordering along two axes and measuring distance between connection points which was found to be 7.4 Å and thus sufficiently large. No energy calculation could be performed due to unresolved atoms in the crystal structure.*

UPy (SOBLUQ)

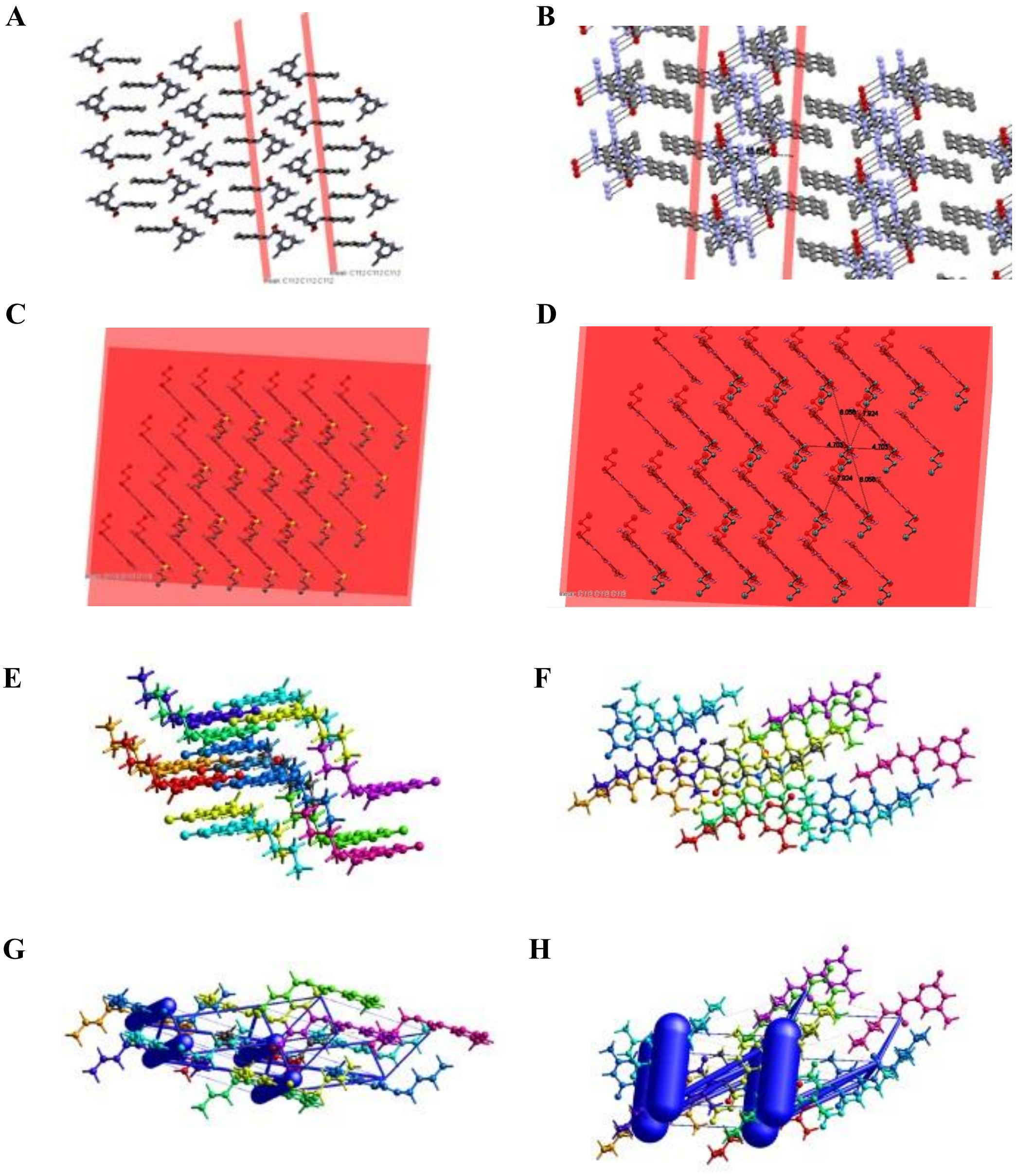

I

[B3LYP/6-31G(d,p)]

	N	Symop	R	E_ele	E_pol	E_dis	E_rep	E_tot
	0	x,y,z	7.92	1.5	-0.1	-1.9	0.0	-0.2
	0	-x,-y,-z	7.19	-158.3	-32.5	-30.2	161.7	-117.7
	0	x,y,z	4.70	-2.6	-2.9	-59.2	30.9	-37.3
	0	x,y,z	8.06	-7.6	-2.8	-8.9	11.6	-10.7
	0	-x,-y,-z	10.58	-74.4	-18.9	-22.9	88.8	-57.7
	0	-x,-y,-z	8.41	-0.0	-1.6	-28.9	13.9	-17.8
	0	-x,-y,-z	11.40	-1.0	-0.1	-0.8	0.0	-1.8
	0	-x,-y,-z	9.27	-4.2	-0.3	-30.4	18.6	-19.6
	0	x,y,z	10.24	0.9	-0.0	-0.4	0.0	0.7
	0	-x,-y,-z	10.64	-1.3	-0.1	-8.4	5.0	-5.7
	0	-x,-y,-z	8.29	-5.5	-1.2	-13.5	2.7	-16.8
	0	-x,-y,-z	12.46	-1.2	-0.0	-0.2	0.0	-1.5
	0	x,y,z	15.27	0.0	-0.0	-0.1	0.0	-0.0

***Figure S84**: Crystal Lattice Analysis of the crystalline unit UPy (CCDC identifier: SOBLUQ). (A,B) Identification of the layer. (C,D) Looking for ordering along two axes and measuring distance between connection points which was found to be 4.7 Å and thus too small. (E-I) Energy calculations using B3LYP/6-31G(d,p) showing the overlap with the layers and the total attractive energy between neighbors which was found to be −389.1 kJ/mol.*

Val (EWOTUF)

A

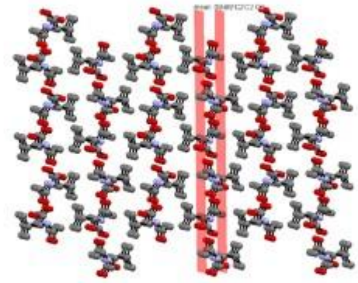

B

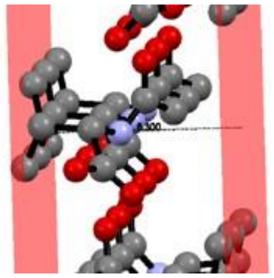

C

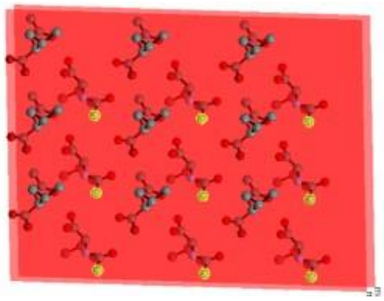

D

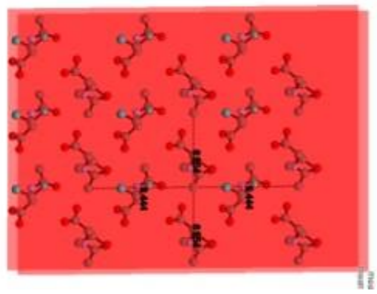

E

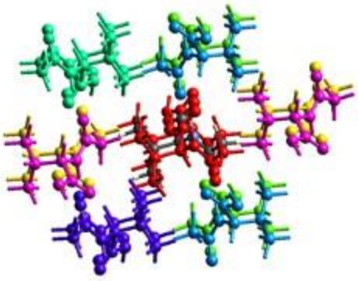

F

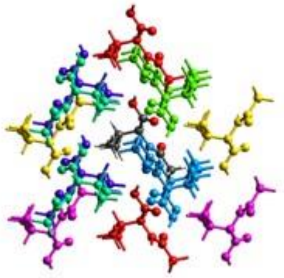

G

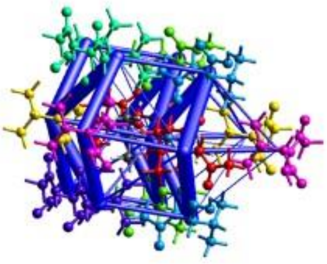

H

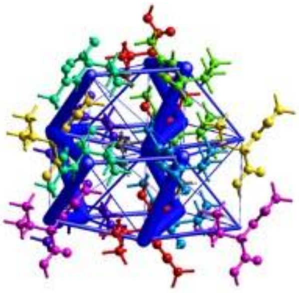

I

[HF/3-21G]

	N	Symop	R	E_ele	E_pol	E_dis	E_rep	E_tot
(red)	2	x,y,z	6.65	7.0	-1.8	-14.5	4.5	-3.4
(yellow)	2	-x+1/2,-y,z+1/2	6.61	-6.3	-1.8	-20.5	10.0	-18.0
(green)	2	-x,y+1/2,-z+1/2	6.21	-62.1	-17.6	-12.4	38.5	-54.7
(light green)	2	x+1/2,-y+1/2,-z	7.82	0.6	-0.6	-6.3	1.0	-4.6
(light blue)	2	-x,y+1/2,-z+1/2	5.88	-40.2	-13.4	-26.4	31.4	-48.0
(blue)	2	x+1/2,-y+1/2,-z	7.25	-2.7	-0.4	-12.1	4.6	-10.1
(magenta)	2	-x+1/2,-y,z+1/2	9.16	0.2	-0.1	-4.0	0.5	-3.0

Figure S85: *Crystal Lattice Analysis of the crystalline unit Val (CCDC identifier: EWOTUF). (A,B) Identification of the layer. (C,D) Looking for ordering along two axes and measuring distance between connection points which was found to be 6.7 Å and thus too small. (E-I) Energy calculations using HF/3-21G showing the overlap with the layers and the total attractive energy between neighbors which was found to be −212.2 kJ/mol.*

TTF (HITTOU)

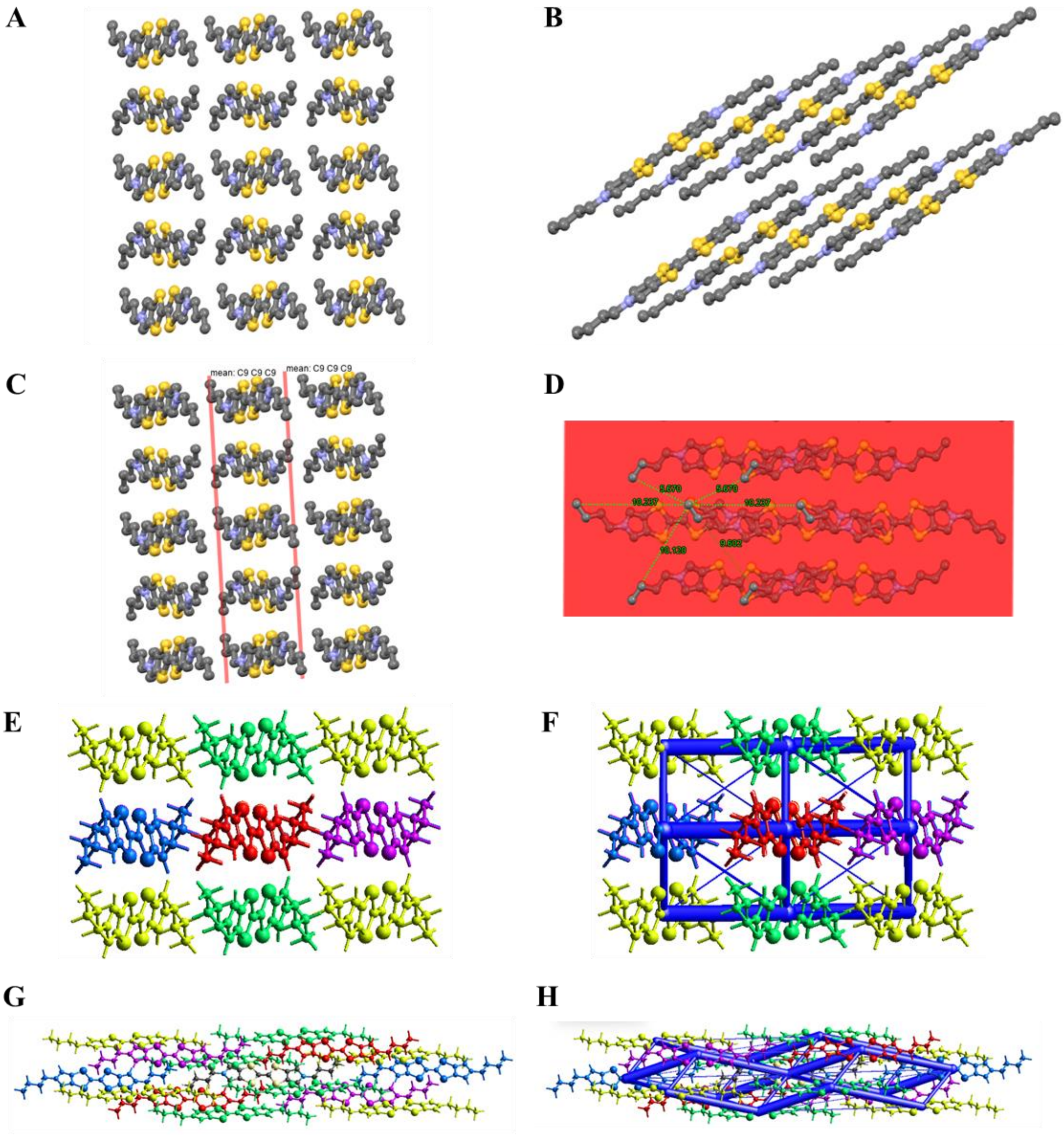

I

	N	Symop	R	Electron Density	E_ele	E_pol	E_dis	E_rep	E_tot
	2	x, y, z	10.24	B3LYP/6-31G(d,p)	-29.6	-6.9	-75.0	54.8	-67.9
	4	-x+1/2, y+1/2, -z	18.43	B3LYP/6-31G(d,p)	0.4	-0.3	-6.7	0.0	-5.6
	4	-x+1/2, y+1/2, -z	7.58	B3LYP/6-31G(d,p)	-12.7	-3.9	-37.3	37.3	-25.8
	2	x, y, z	22.23	B3LYP/6-31G(d,p)	1.4	-0.2	-9.5	0.0	-7.0
	2	x, y, z	13.25	B3LYP/6-31G(d,p)	-5.3	-4.8	-53.4	0.0	-55.6

Figure S86*: Crystal Lattice Analysis of the crystalline unit TTF (CCDC identifier: HITTOU). (A,B) Identification of the layer. (C,D) Looking for ordering along two axes and measuring distance between connection points which was found to be 7.4 Å and thus sufficiently large. (E-I) Energy calculations using B3LYP/6-31G(d,p) showing the overlap with the layers and the total attractive energy between neighbors which was found to be −187.4 kJ/mol.*

TDPP (OVOVUS01)

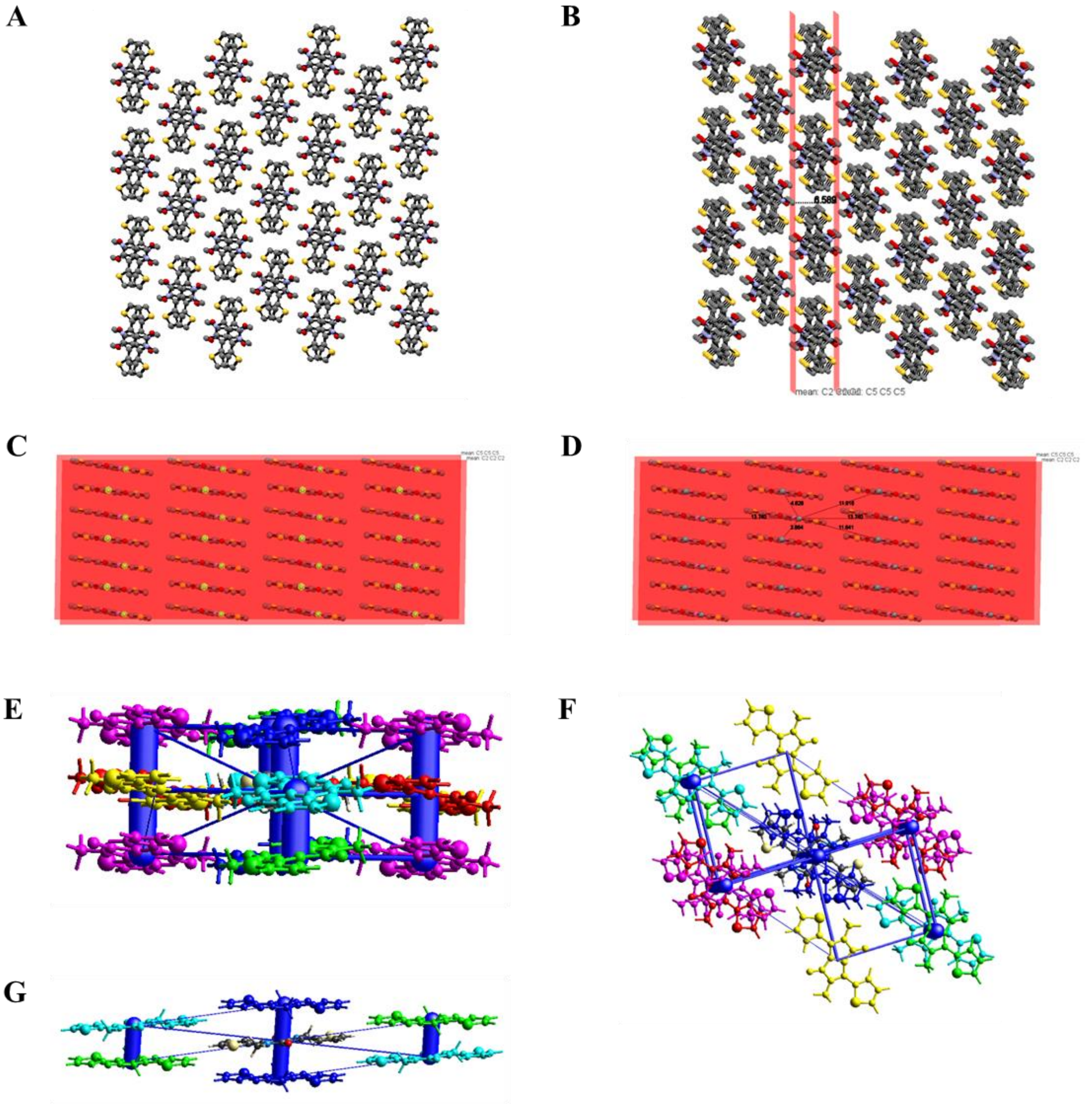

H

	N	Symop	R	Electron Density	E_ele	E_pol	E_dis	E_rep	E_tot
(red)	2	-	9.60	B3LYP/6-31G(d,p)	-4.5	-2.7	-17.0	15.9	-11.7
(yellow)	2	-	10.40	B3LYP/6-31G(d,p)	-11.2	-3.1	-20.2	23.3	-17.4
(green)	2	-	14.16	B3LYP/6-31G(d,p)	-0.3	-0.8	-9.3	7.8	-4.2
(cyan)	2	x, y, z	13.74	B3LYP/6-31G(d,p)	-3.7	-0.7	-11.3	10.2	-7.9
(blue)	2	-	3.42	B3LYP/6-31G(d,p)	-23.7	-5.3	-118.7	79.0	-83.5
(magenta)	4	-x+1/2, y+1/2, -z+1/2	10.20	B3LYP/6-31G(d,p)	-3.6	-0.4	-10.5	7.1	-8.8

Figure S87: *Crystal Lattice Analysis of the crystalline unit TDPP (CCDC identifier: OVOVUS01). (A,B) Identification of the layer. (C,D) Looking for ordering along two axes and measuring distance between connection points which was found to be 4.6 Å and thus too small. (E-H) Energy calculations using B3LYP/6-31G(d,p) showing the overlap with the layers and the total attractive energy between neighbors which was found to be −191.2 kJ/mol.*

6. Crystal Structure Determination

Pyrene-OPent:

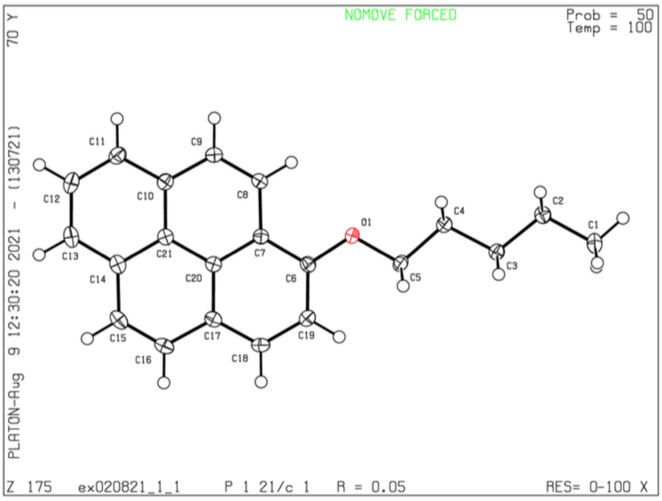

Identification code	ex020821_1_1
Empirical formula	$C_{21}H_{20}O$
Formula weight	288.37
Temperature/K	100.0(1)
Crystal system	monoclinic
Space group	$P2_1/c$
a/Å	7.4323(2)
b/Å	29.3078(6)
c/Å	7.6658(2)
α/°	90
β/°	116.771(4)
γ/°	90
Volume/Å^3	1490.82(8)
Z	4
ρ_{calc}g/cm^3	1.285
μ/mm^{-1}	0.592
F(000)	616.0
Crystal size/mm^3	0.19 × 0.065 × 0.032
Radiation	Cu Kα (λ = 1.54184)
2Θ range for data collection/°	6.032 to 160.574
Index ranges	$-9 \le h \le 7$, $-37 \le k \le 37$, $-8 \le l \le 9$
Reflections collected	17399
Independent reflections	3178 [R_{int} = 0.0422, R_{sigma} = 0.0289]
Data/restraints/parameters	3178/0/200
Goodness-of-fit on F^2	1.074
Final R indexes [I>=2σ (I)]	R_1 = 0.0488, wR_2 = 0.1339
Final R indexes [all data]	R_1 = 0.0553, wR_2 = 0.1388
Largest diff. peak/hole / e Å^{-3}	0.32/-0.25

Experimental

Single crystals of $C_{21}H_{20}O$ [ex020821_1_1] were obtained through solvent vapor diffusion using chloroform and diethyl ether. A suitable crystal was selected and measured on a XtaLAB Synergy, Dualflex, Pilatus 300K diffractometer. The crystal was kept at 100.0 K during data collection. Using Olex2,[3] the structure was solved with the SHELXT[4] structure solution program using Intrinsic Phasing and refined with the SHELXL[5] refinement package using Least Squares minimization.

PentAQ

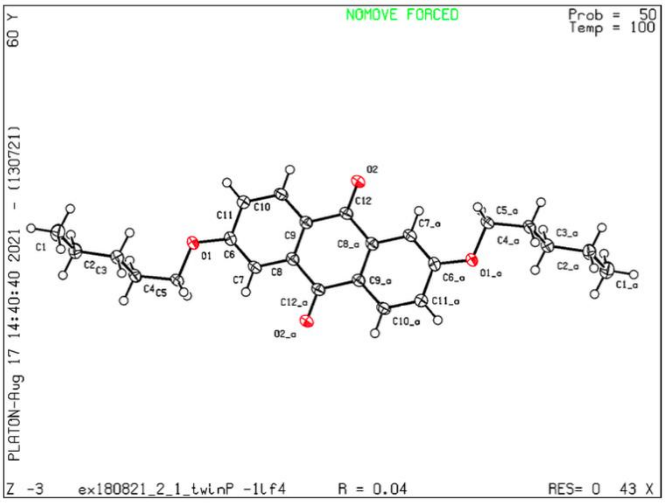

Identification code	ex180821_2_1
Empirical formula	$C_{24}H_{28}O_4$
Formula weight	380.46
Temperature/K	100.0(1)
Crystal system	triclinic
Space group	P-1
a/Å	4.60720(10)
b/Å	8.1094(2)
c/Å	13.4590(4)
α/°	82.457(2)
β/°	85.174(3)
γ/°	81.403(2)
Volume/Å^3	491.83(2)
Z	1
ρ_{calc}g/cm^3	1.285
μ/mm^{-1}	0.690
F(000)	204.0
Crystal size/mm^3	0.25 × 0.069 × 0.025
Radiation	Cu Kα (λ = 1.54184)
2Θ range for data collection/°	6.64 to 133.082
Index ranges	$-5 \le h \le 5, -9 \le k \le 9, -15 \le l \le 15$
Reflections collected	2979
Independent reflections	2979 [R_{int} = 0.0500, R_{sigma} = 0.0132]
Data/restraints/parameters	2979/0/129
Goodness-of-fit on F^2	1.066
Final R indexes [I>=2σ (I)]	R_1 = 0.0419, wR_2 = 0.1170
Final R indexes [all data]	R_1 = 0.0473, wR_2 = 0.1215
Largest diff. peak/hole / e Å^{-3}	0.19/-0.23

Experimental

Single crystals of $C_{24}H_{28}O_4$ [ex180821_2_1] were obtained through solvent vapor diffusion using chloroform and diethyl ether. A suitable crystal was selected and measured on a XtaLAB Synergy, Dualflex, Pilatus 300K diffractometer. The crystal was kept at 100.0 K during data collection. Using Olex2,[3] the structure was solved with the SHELXT[4] structure solution program using Intrinsic Phasing and refined with the SHELXL[5] refinement package using Least Squares minimization.

7. References

(1) Lamers, B. A. G.; Waal, B. F. M. de; Meijer, E. W. The Iterative Synthesis of Discrete Dimethylsiloxane Oligomers: A Practical Guide. *J. Polym. Sci.* **2021**, *59* (12), 1142–1150.

(2) Van Genabeek, B.; de Waal, B. F. M.; Gosens, M. M. J.; Pitet, L. M.; Palmans, A. R. A.; Meijer, E. W. Synthesis and Self-Assembly of Discrete Dimethylsiloxane-Lactic Acid Diblock Co-Oligomers; the Dononacontamer and Its Shorter Homologues. *J. Am. Chem. Soc.* **2016**, *138* (12), 4210.

(3) Dolomanov, O. V.; Bourhis, L. J.; Gildea, R. J.; Howard, J. A. K.; Puschmann, H. OLEX2: A Complete Structure Solution, Refinement and Analysis Program. *J. Appl. Crystallogr.* **2009**, *42* (2), 339–341.

(4) Sheldrick, G. M. SHELXT - Integrated Space-Group and Crystal-Structure Determination. *Acta Crystallogr. Sect. A Found. Crystallogr.* **2015**, *71* (1), 3–8.

(5) Sheldrick, G. M. Crystal Structure Refinement with SHELXL. *Acta Cryst. C.* **2015**, *71* (1), 3–8.
